# Taxonomic studies on the sac spider genus *Clubiona* (Araneae, Clubionidae) from Xishuangbanna Rainforest, China

**DOI:** 10.3897/zookeys.1034.59413

**Published:** 2021-04-26

**Authors:** Jianshuang Zhang, Hao Yu, Shuqiang Li

**Affiliations:** 1 School of Life Sciences; 2 Guizhou Normal University, Guiyang, Guizhou, China; 3 School of Biological Sciences, Guizhou Education University, Guiyang, Guizhou, China; 4 Institute of Zoology, Chinese Academy of Sciences, Beijing, China

**Keywords:** Checklist, DNA barcoding, new species, new synonymy, taxonomy, tropical rainforest

## Abstract

Spiders of the genus *Clubiona* Latreille, 1804 from Xishuangbanna, Yunnan Province, China are studied. A total of 47 species is reported and illustrated, including 14 new species and two new synonyms. Twelve of the new species belong to four species groups: *C. dengpao* Yu & Li, **sp. nov.**, *C. subdidentata* Yu & Li, **sp. nov.**, *C. tixing* Yu & Li, **sp. nov.**, *C. xiaoci* Yu & Li, **sp. nov.**, *C. xiaokong* Yu & Li, **sp. nov.**, *C. yejiei* Yu & Li, **sp. nov.**, *C. zhaoi* Yu & Li, **sp. nov.** and *C. zhigangi* Yu & Li, **sp. nov.** from the *C. corticalis* group; *C. mii* Yu & Li, **sp. nov.** and *C. subtongi* Yu & Li, **sp. nov.** from the *C. ternatensis* group; *C. banna* Yu & Li, **sp. nov.** from the *C. filicata* group; and *C. menglun* Yu & Li, **sp. nov.** from the *C. trivialis* group. The remaining two new species, *C. shuangsi* Yu & Li, **sp. nov.** and *C. wangchengi* Yu & Li, **sp. nov.**, are not readily assignable to any of the existing species groups. The female of *C. cochlearis* Yu & Li, 2019, the female of *C. tiane* Yu & Li, 2019, the female of *C. bicornis* Yu & Li, 2019, the male of *C. lala* Jäger & Dankittipakul, 2010 and the true female of *C. suthepica* Dankittipakul, 2008 are described for the first time. Two new synonyms are: *C. vukomi* Jäger & Dankittipakul, 2010 **syn. nov**. = *C. circulata* Zhang & Yin, 1998; *C. melanothele* Thorell, 1895 **syn. nov**. = *Clubiona melanosticta* Thorell, 1890. A checklist of *Clubiona* species from Xishuangbanna is provided. The DNA barcodes of almost all of the species were obtained for species delimitation, matching of sexes and future use.

## Introduction

*Clubiona* Latreille, 1804 is the type genus of the Clubionidae Wagner, 1887 and currently includes 506 extant species that are found worldwide except for the Polar Regions and South America (WSC 2021). This genus comprises 61% of the total number of species of the family (Marusik and Omelko 2018; Zhang and Yu 2020; WSC 2021). Despite its high species diversity, the genus *Clubiona* remains inadequately studied: almost half of the species are known from a single sex or juveniles (82 from males only, 133 from females only, two from juveniles only), and in some cases, the adults are apparently mismatched, or conspecific males and females have been described as separate species (Deeleman-Reinhold 2001; Jäger and Dankittipakul 2010; WSC 2021); the descriptions from early studies are rather brief, many species are not illustrated, or illustrations are inadequate; types of some species do not exist or are difficult to locate or access.

*Clubiona* are common spiders in China, with 152 species, of which 106 are known from both sexes (WSC 2021). Except for very few species, almost all of Chinese *Clubiona* were described or redescribed in the past 30 years (WSC 2021). *Clubiona* from Thailand and Laos have been well studied by Deeleman-Reinhold (2001), and Dankittipakul and co-authors (Dankittipakul and Singtripop 2008a, b; Dankittipakul et al. 2012; Jäger and Dankittipakul 2010). *Clubiona* from Myanmar are relatively poorly studied; half of the described species have not been illustrated or descriptions are accompanied by inadequate illustrations (WSC 2021).

Xishuangbanna is a key biogeographic area and a biodiversity hotspot in China (Myers 1988). It shares a border with Myanmar in the southwest and Laos in the southeast and harbours more species diversity than typical tropical rain forests of Southeast Asia (Zhu et al. 2006). Xishuangbanna spiders have received a lot of attention because of an “All Species Inventory” which has been conducted by SL and his team during the last 15 years (Li 2020). *Clubiona* from the region have been studied by Zhang and Yin (1998), Yin et al. (2012), Wu et al. (2015) and Yu and Li (2019a, b). These studies have described 25 new species and increased the total clubionid species number to 27 in the last 22 years (Yu and Li 2019b). However, based on samples from Xishuangbanna collected during 2007 to 2019, the diversity of the genus *Clubiona* is underestimated.

In the present paper, a checklist of Xishuangbanna *Clubiona* spiders is provided based on published literature and new collections. A total of 51 species are recorded, among them, 47 were collected and illustrated, including fourteen new species. The goal of this paper is to provide a detailed description and diagnosis of these new species, to provide the first description of the male or females of 4 known species, to synonymise *C. vukomi* Jäger & Dankittipakul, 2010 and *C. melanothele*, Thorell 1895 and to provide comparative illustrations of male palps and female epigynes for all Xishuangbanna *Clubiona* species.

## Materials and methods

Almost all of the species are leaf-dwellers. Most specimens were collected by canopy fogging, while a few were obtained by beating vegetation and pitfall trapping. Specimens were preserved in 75 or 95% ethanol. All type specimens are deposited in the Institute of Zoology, Chinese Academy of Sciences (**IZCAS**) in Beijing, China (curator Jun Chen).

Specimens were examined using a LEICA M205C and an Olympus SZX7 stereomicroscope. Further details were studied under a CX41 compound microscope. Male and female copulatory organs were examined and illustrated after dissection. Left male palps are illustrated unless otherwise indicated (photos of the right palp were horizontally mirrored in the figures to allow easier comparison with other species). Epigynes were removed and cleared in lactic acid or warm 10% potassium hydroxide (KOH) solution. Some vulvae were imaged after being embedded in Arabic gum. Images were captured with a Canon EOS 70D digital camera mounted on an Olympus CX41 compound microscope and assembled using Helicon Focus 6.80 image stacking software. All measurements were obtained using an Olympus SZX7 stereomicroscope and are given in millimetres. Eye diameters are taken from the widest distance. The total body length does not include chelicerae or spinnerets. Leg lengths are given as total length (femur, patella + tibia, metatarsus, tarsus). Terminology in the text and figure legends follows Yu and Li (2019a, b), Yu et al. (2017a), Zhang et al. (2018) and Dankittipakul and Singtripop (2008a, b).

A partial fragment (650 bp) of the mitochondrial gene cytochrome c oxidase subunit I (COI) was amplified and sequenced to obtain the genetic distances between morphologically similar species and to confirm identifications and sex pairing accuracy. However, we were unable to obtain good extractions from *C. kurosawai* Ono, 1986, *C. rama* Dankittipakul & Singtripop, 2008, *C. subyaginumai* Yu & Li, 2019, *C. tixing* sp. nov., *C. yejiei* sp. nov., and *C. zhanggureni* Yu & Li, 2019 and the male of *C. subquebecana* Yu & Li, 2019. Although recorded from Xishuangbanna, the following species were not collected by us and were unavailable for molecular work: *C. japonicola* Bösenberg & Strand, 1906, *C. filoramula* Zhang & Yin, 1998, *C. heteroducta* Zhang & Yin, 1998 and *C. zhangyongjingi* Li & Blick, 2019.

The primers used were: LCOI1490 (5’-GGTCAACAAATCATAAAGATATTG-3’) and HCOI2198 (5’-TAAACTTCAGGGTGACCAAAAAAT-3’). For additional information on extraction, amplification and sequencing procedures, see Malumbres-Olarte J and Vink (2012). Raw sequences were edited and assembled using BioEdit v.7.2.5 (Hall 1999), and uncorrected pairwise distances between sequences were calculated using MEGA v.10.0 (Tamura et al. 2013). All sequences were analysed using BLAST and are deposited in GenBank. The accession numbers are provided in Table 1.

**Table 1.** Checklist of *Clubiona* species from Xishuangbanna and voucher specimen information.

Species groups	Species	Sex	Figures in the present paper	Voucher code	GenBank accession number	References
*milingae group*	C. yaoi*	♂	62A, B, 72A, B,	YHCLU0002	MW731651	Yu and Li 2019a; present paper
		♀	80A, 88A, 96A	YHCLU0003	MW731650	
*corticalis* group	C. cochlearis*	♂	53A, 63A	YHCLU0068	MW731626	Yu and Li 2019b; female supplemented in present paper
		♀	1, 73A, 81A, 89A	YHCLU0079	MW731621	
	*C. dengpao* sp. nov.*	♀	2, 73B, 81B, 89B	YHCLU0080	MW731620	present paper
	C. didentata*	♂	54A, 64A	YHCLU0015	MW731648	Zhang and Yin 1998; female supplemented in Yu and Li 2019b; present paper
		♀	74E, 82E, 90E	YHCLU0016	MW731647	
	C. kai*	♂	54B, 64B	YHCLU0259	MW731584	female supplemented in Yu and Li 2019b; present paper
		♀	74C, 82C, 90C	YHCLU0052	MW731634	
	C. kurosawai*	♂	3, 4E, F, 56A, 66A	–		present paper
		♀	4A–D, G, H, 76A, 84A, 92A	–		
	C. moralis*	♂	5, 6E, F, 55A, 65A,	YHCLU0025	MW731643	present paper
		♀	6A–D, G, H, 75A, 83A, 91A	YHCLU0024	MW731644	
	C. multidentata*	♂	7, 8E, F, 56B, 66B,	YHCLU0076	MW731624	present paper
		♀	8A–D, G, H, 75D, 83D, 91D	YHCLU0077	MW731623	
	C. parconcinna*	♂	9, 10E, F, 55C, 65C	YHCLU0143	MW731590	present paper
		♀	10A–D, G, H, 75C, 83C, 91C	YHCLU0260	MW731583	
	C. pollicaris*	♂	11, 12E, F, 56C, 66C	YHCLU0020	MW731646	Wu et al. 2015; present paper
		♀	12A–D, G, H, 76B, 84B, 92B	YHCLU0021	MW731645	
	*C. rama* *	♂	13, 14, 53B, 63B	–		Yu et al. 2017b; present paper
*corticalis* group	*C. subdidentata* sp. nov.*	♀	15, 74F, 82F, 90F	YHCLU0073	MW731625	present paper
	C. submoralis*	♂	16, 17E, F, 55B, 65B	YHCLU0028	MW731642	Wu et al. 2015; present paper
		♀	17A–D, G, H, 75B, 83B, 91B	YHCLU0029	MW731641	
	C. subrama*	♂	53C, 63C	YHCLU0083	MW731619	Yu and Li 2019a; present paper
		♀	73E, 81E, 89E	YHCLU0084	MW731618	
	C. subyaginumai*	♂	54C, 64C	–		Yu and Li 2019a; present paper
		♀	75F, 83F, 91F	–		
	*C. tixing* sp. nov.*	♀	18, 73D, 81D, 89D	–		present paper
	C. tiane*	♂	54D, 64D	YHCLU0054	MW731632	Yu and Li 2019b; female supplemented in present paper
		♀	19, 74D, 82D, 90D	YHCLU0053	MW731633	
	*C. xiaoci* sp. nov.*	♂	20, 21E, F, 55D, 65D	YHCLU0088	MW731614	present paper
		♀	21A–D, G, H, 75E, 83E, 91E	YHCLU0089	MW731613	
	*C. xiaokong* sp. nov.*	♀	22, 74A, 82A, 90A	YHCLU0078	MW731622	present paper
	*C. yejiei* sp. nov..*	♀	23, 73C, 81C, 89C	–	–	present paper
	*C. zhaoi* sp. nov.*	♀	24, 74B, 82B, 90B	YHCLU0086	MW731616	present paper
	*C. zhigangi* sp. nov.*	♂	25, 26E, F, 53D, 63D	YHCLU0185	MW731586	present paper
		♀	26A–D, G, H, 73F, 81F, 89F	YHCLU0138	MW731592	
*ternatensis* group	*C. heteroducta*		–	–		Zhang and Yin 1998
	*C. mii* sp. nov.*	♀	27, 77A, 85A, 93A	YHCLU0065	MW731629	present paper
	C. subkuu*	♂	57C, 67C	YHCLU0039	MW731637	Yu and Li 2019a; present paper
		♀	77B, 85B, 93B	YHCLU0038	MW731638	
	*C. subtongi* sp. nov.*	♂	28, 29, 57D, 67D	YHCLU0056	MW731630	present paper
	C. theoblicki*	♂	57A, 67A	YHCLU0092	MW731612	Yu and Li 2019a; present paper
		♀	77C, 85C, 93C	YHCLU0093	MW731611	
	C. tongi*	♂	57B, 67B	YHCLU0055	MW731631	Yu and Li 2019b; present paper
		♀	77D, 85D, 93D	YHCLU0095	MW731610	
	C. zhengi*	♂	57E, 67E	YHCLU0042	MW731636	Yu and Li 2019a; present paper
		♀	77E, 85E, 93E	YHCLU0043	MW731635	
*japonicola* group	*C. japonicola*	♂♀	–	–	–	Yin et al. 2012
*filicata group*	C. abnormis*	♂	30, 31, 60C, 70C	YHCLU0113	MW731597	present paper
	*C. banan* sp. nov.*	♂	32, 33E, F, 58C, 68C	YHCLU0104	MW731604	present paper
		♀	33A–D, G, H, 78E, 86E, 94E	YHCLU0139	MW731591	
	C. circulata*	♂	34, 35E, F, 59C, 69C	YHCLU0108	MW731600	Zhang and Yin 1998; present paper
		♀	35A–D, 79A, 87A, 95A	YHCLU0156	MW731589	
	C. reichlini*	♂	36, 37E, F, 58A, 68A	YHCLU0263	MW731582	present paper
		♀	37A–D, G, H, 79B, 87B, 95B	YHCLU0264	MW731581	
	C. filicata*	♂	38, 39, 58B, 68B	YHCLU0107	MW731601	Zhang and Yin 1998; present paper
	*C. filoramula*	♂	–	–	–	Zhang and Yin 1998
	C. grucollaris*	♂	40, 41E, F, 60A, 70A	YHCLU0105	MW731603	present paper
		♀	41A–D, G, H, 79C, 87C, 95C	YHCLU0106	MW731602	
	C. lala*	♂	42, 43E, F, 60B, 70B	YHCLU0110	MW731599	male supplemented in present paper
		♀	43A–D, G, H, 79D, 87D, 95D	YHCLU0111	MW731598	
	C. melanosticta*	♂	44, 45E, F, 59A, 69A	YHCLU0011	MW731649	present paper
		♀	45A–D, G, H, 78F, 86F, 94F	YHCLU0164	MW731588	
	C. suthepica*	♂	46, 47E, F, 59D, 69D	YHCLU0114	MW731596	female supplemented in present paper
		♀	47A–D, G, H, 79E, 87E, 95E	YHCLU0209	MW731585	
	C. yueya*	♂	60D, 70D	YHCLU0116	MW731595	Yu and Li 2019b; present paper
		♀	79F, 87F, 95F	YHCLU0117	MW731594	
	C. zhanggureni*	♂	59B, 69B	–	–	Yu and Li 2019b; present paper
*filicata group*	*C. zhangyongjingi*		–	–	–	Zhang and Yin 1998; Li and Blick, 2019
*trivialis* group	C. bicornis*	♂	61A, 71A	YHCLU0180	MW731587	Yu and Li 2019b; female supplemented in present paper
		♀	48, 77F, 85F, 93F	YHCLU0099	MW731608	
	C. cheni*	♂	61B, 71B	YHCLU0033	MW731639	Yu and Li 2019a; present paper
		♀	78A, 86A, 94A	YHCLU0032	MW731640	
	*C. menglun* sp. nov.*	♀	49, 78B, 86B, 94B	YHCLU0097	MW731609	present paper
	C. subasrevida*	♂	61C, 71C	YHCLU0100	MW731607	Yu and Li 2019b; present paper
		♀	78C, 86C, 94C	YHCLU0101	MW731606	
	C. subquebecana*	♂	61D, 71D	–	–	Yu and Li 2019a; present paper
		♀	78D, 86D, 94D	YHCLU0103	MW731605	
Species group not assigned	C. jiandan*	♂	62C, 72C	YHCLU0066	MW731628	Yu and Li 2019b; present paper
		♀	80B, 88B, 96B	YHCLU0067	MW731627	Yu and Li 2019b; present paper
	*C. shuangsi* sp. nov.*	♂	50, 51E–G, 62D, 72D	YHCLU0135	MW731593	
		♀	51A–D, H, I, 80D, 88D, 96D	YHCLU0085	MW731617	present paper
	*C. wangchengi* sp. nov.*	♀	52, 80C, 88C, 96C	YHCLU0087	MW731615	

Note: A total of 51 *Clubiona* species is reported from Xishuangbanna, of which 47 (marked with asterisks) were collected by the authors. The table includes only references that explicitly documented that the species was found in Xishuangbanna. For a complete list of taxonomic references, see taxonomy below and WSC (2021).

Abbreviations used in the text or figures are given in Table 2. References to figures in the cited papers are listed in lowercase (fig. or figs); figures from this paper are noted with an initial capital (Fig. or Figs).

**Table 2.** List of abbreviations used in the text or figures.

**Male palp**	
	conductor
**C**	Note: two types of conductors are considered in the present paper, the first type of conductor is separate from the tegulum, and the second type of conductor is represented by a membranous area fused to the tegulum.
**DCA**	dorsal cymbial apophysis
**E**	embolus
**EB**	embolar base
**FA**	femoral apophysis
**PFR**	prolateral femoral ridge
**PPA**	prolateral patellar apophysis
**PTA**	prolateral tibial apophysis
**RPA**	retrolateral patellar apophysis
**RTA**	retrolateral tibial apophysis
**TA**	tegular apophysis
**TH**	tegular hump
**VTA**	ventral tibial apophysis
**Epigyne**	
**A**	atrium
**AAM**	atrial anterior margin
**AM**	atrial membrane
**APM**	atrial posterior margin
**BS**	bursa
**CD**	copulatory duct
**CO**	copulatory opening
**FD**	fertilisation duct
**R**	epigynal ridge
**SB**	spermathecal base
**SH**	spermathecal head
**SP**	spermatheca
**SS**	spermathecal stalk
**Ocular area**	
**AER**	anterior eye row
**ALE**	anterior lateral eyes
**AME**	anterior median eyes
**MOQ**	median ocular quadrangle
**MOQA**	MOQ anterior width
**MOQL**	length of MOQ
**MOQP**	MOQ posterior width
**PER**	posterior eye row
**PLE**	posterior lateral eyes
**PME**	posterior median eyes
**AME–AME**	distance between AMEs
**AME–ALE**	distance between AME and ALE
**PME–PME**	distance between PMEs
**PME–PLE**	distance between PME and PLE
**Institutions**	
**IZCAS**	Institute of Zoology, Chinese Academy of Sciences, Beijing, China
**XTBG**	Xishuangbanna Tropical Botanic Garden, Yunnan, China

## Taxonomy

### Family Clubionidae Wagner, 1887

#### Genus Clubiona

Taxon classificationAnimaliaAraneaeClubionidae

Latreille, 1804

96D05C06-E0E3-5A5D-B3CB-9B2608BC0B3B

Clubiona Latreille, 1804: 134 (type species Araneus pallidulus Clerck, 1757).
Hirtia Thorell, 1881: 222 (type species H. ternatensis Thorell, 1891).
Atalia Thorell, 1887: 54 (type species A. concinna Thorell, 1887).
Tolophus Thorell, 1891: 26 (type species T. submaculatus Thorell, 1891).
Paraclubiona Lohmander, 1944: 19 (type species Aranea corticalis Walckenaer, 1802).
Microclubiona Lohmander, 1944: 20 (type species C. trivialis C.L. Koch, 1834).
Hyloclubiona Lohmander, 1944: 20 (subgenus of Microclubiona, type species C. comta C.L. Koch, 1839).
Heteroclubiona Lohmander, 1944: 20 (subgenus of Clubiona, type species C. terrestris Westring, 1851).
Epiclubiona Lohmander, 1944: 20 (subgenus of Clubiona, type species C. neglecta O. Pickard-Cambridge, 1862, not C. similis L. Koch, 1866 as indicated by Wunderlich 2011).
Euryclubiona Lohmander, 1944: 21 (subgenus of Clubiona, type species C. subsultans Thorell, 1875).
Gauroclubiona Lohmander, 1944: 21 (subgenus of Clubiona, type species C. coerulescens L. Koch, 1867).
Bucliona Benoit, 1977: 68 (type species Clubiona dubia O. Pickard-Cambridge, 1869).
Japoniona Mikhailov, 1990: 143 (subgenus of Clubiona, type species C. japonica L. Koch, 1878).
Bicluona Mikhailov, 1994: 52 (subgenus of Clubiona, type species Liocranum jucundum Karsch, 1879).
Marmorclubiona Wunderlich, 2011: 136 (type species C. marmorata L. Koch, 1866).
Breviclubiona Wunderlich, 2011: 139 (type species C. brevipes Blackwall, 1841).
Anaclubiona Ono, 2010: 4 (type species C. zilla Dönitz & Strand, 1906).

##### Comments.

*Clubiona* sensu lato currently contains more than 500 nominal species and is one of the largest genera of Araneae (Marusik and Omelko 2018; WSC 2021). Several major taxonomic studies on a regional scale have been conducted, e.g., Simon (1932) for the French species, Lohmander (1944) for the Swedish species, Wiehle (1965) for the German species, Wunderlich (2011) for the European species, Edwards (1958) for the North American species, Dondale and Redner (1982) for the Canadian and Alaskan species, Mikhailov (1990, 1991, 1995, 2002, 2012) for the Palaearctic species, and Deeleman-Reinhold (2001) for the Southeast Asian species.

There are 14 generic names that are currently considered junior synonyms of *Clubiona* (see above list). Beyond that, at least ten subgeneric and 20 species group names have been recognised for subdivisions of the genus (Lohmander 1944; Dondale and Redner 1982; Mikhailov 1990, 1991, 1995, 2002; Deeleman-Reinhold 2001). However, almost all generic and subgeneric statuses were suppressed by Mikhailov (1990, 1991, 1995, 2002, 2012) and Deeleman-Reinhold (2001). At present, only a dozen species group names are used for the taxonomy of the genus. Although there is no agreement on the limits of most species groups, some groups present a distinct set of characters with relatively stable species composition and are currently accepted by most taxonomists. There are at least 16 species groups discussed or frequently used in recent publications: *C. apiculata* group, *C. corticalis* group (corresponds to *Atalia* and *Paraclubiona*), *C. hystrix* group (corresponds to *Hirtia*), *C. japonica* group (corresponds to *Tolophus* and *Japoniona*), *C. trivialis* group (corresponds to *Microclubiona*), *C. pallidula* group and *C. obesa* group (belongs to *Clubiona* s. str.), *C. abboi* group (mainly distributed in North America, may be elevated to genus level in the future), *C. similis* group (corresponds to *Epiclubiona*), *C. lutescens* group (corresponds to *Heteroclubiona*), *C. reclusa* group (corresponds to *Euryclubiona*), *C. caerulescens* group (corresponds to *Gauroclubiona*), *C. marmorata* group (corresponds to *Marmorclubiona*), *C. brevipes*s group (corresponds to *Breviclubiona*), *C. zilla* group (corresponds to *Anaclubiona*), *C. genevensis* group.

According to the quite diverse copulatory structures of both sexes, *Clubiona* sensu lato has been widely regarded as paraphyletic and will likely be split in the future (Wunderlich 2011; Marusik and Omelko 2018; Zhang and Yu 2020). However, we agree with Mikhailov (2012) regarding the need of an extensive, large-scale review of the genus. Consequently, the present study follows the WSC (2021) and Mikhailov (2012), and temporarily places all species in *Clubiona* sensu lato.

#### Key to species groups occurring in Xishuangbanna (males)

**Table:** 

1	Dorsum of abdomen/carapace/or legs with pattern (Figs 31A, C, 33E, F, 35E, F, 37E, F, 39A, C, 41E, F, 43E, F, 45E, F, 47E, F)	***C. filicata* group**
–	Legs and body dorsally without distinct pattern	**2**
2	Bulb enlarged or inflated, and protruded or prolapsed, with indistinct sperm duct (Figs 53A–D, 54A–D, 55A–D, 56A–C, 63A–D, 64A–D, 65A–D, 66A–C)	***C. corticalis* group**
–	Tegulum relatively flattened, sperm duct distinct	**3**
3	Sperm duct simple, U-shaped, or V-shaped in ventral view (Figs 57A–E, 62A, C)	**4**
–	Sperm duct sinuous (Figs 61A–D, 62D)	**6**
4	Conductor absent; embolus relatively long, oriented clockwise along the margin of the tegulum; tegulum distally with a tegular hump (Fig. 57A–E)	***C. ternatensis* group**
–	Conductor present; embolus very short (Figs 62A, C, 72A–C)	**5**
5	Male palp with a dorsal cymbial apophysis and three tibial apophyses, the retrolateral apophysis well-developed and distally forked (Figs 62B, 72B)	***C. milingae* group (*C. yaoi*)**
–	Male palp without dorsal cymbial apophysis, tibia only with a simple and not forked retrolateral apophysis (Figs 62C, 72C)	***C. jiandan***
6	Conductor filiform, separated from tegulum (Figs 50C–E, 62D)	***C. shuangsi* sp. nov.**
–	Conductor absent, or depressed and groovelike	**7**
7	Retrolateral tibial apophysis unbranched (Fig. 71B–D) or bifurcated and processes of different size (Fig. 71A); conductor groovelike, represented by membranous part of tegulum (Figs 61A–D, 71A–D); embolus arched around or angled across the distal end of tegulum, pointed proximally (Figs 61A–D, 71A–D)	***C. trivialis* group**
–	Retrolateral tibial apophysis bifurcated, both processes of similar size; conductor absent; embolus directed retrolaterad, then prolaterodistad	***C. japonicola* group (*C. japonicola*)**

#### Key to species groups occurring in Xishuangbanna (females)

**Table:** 

1	Dorsum of abdomen with pattern (Figs 33G, 35G, 37G, 41G, 43G, 45G, 47G); epigynal atrium broad (almost equal to epigyne width), located at anterior part of epigynal plate (78F, 79A–F, 86F, 87A–F) (atria is relatively small in *C. banna* sp. nov., Figs 78E, 86E)	***C. filicata* group**
–	Abdomen dorsally without distinct pattern; epigynal atrium absent, or located posteriorly, or located anteriorly but small	**2**
2	Copulatory openings located at anterior part of epigyne (Figs 81A–F, 82A–F, 83A–F, 84A, 88D) (located in the middle in *C. pollicaris*, Fig. 84B), well separated from epigastric furrow	**3**
–	Copulatory openings located posteriorly, close to epigastric furrow	**4**
3	Epigynal atrium shaped like an inverted triangle (Figs 80D, 88D)	***C. shaungsi* sp. nov.**
–	Epigynal atrium of variable shapes, but not inverted triangular	***C. corticalis* group**
4	Copulatory openings fused, or closely spaced, or separated by no more than one diameter (Figs 77A–F, 78A–D, 85A–F, 86A–D)	**5**
–	Copulatory openings separated by more than 1.5 × diameters (Figs 80A–C, 88A–C)	7
5	Copulatory openings hidden in ridges, folds, or under hood (Figs 77A–E, 85A–E)	***C. ternatensis* group**
–	Epigynal ridge or fold absent	**6**
6	Copulatory openings large, elongate, length more than 1/3 of epigyne length; copulatory ducts as broad as spermathecae	***C. japonicola* group (*C. japonicola*)**
–	Copulatory openings small, usually circular, diameter no more than 1/5 of epigyne length (Figs 77F, 78A–D, 85F, 86A–D); copulatory ducts slenderer than spermathecae (Figs 93F, 94A–D)	***C. trivialis* group**
7	Tibia I with three pairs of ventral spines; bursae oblong (Fig. 96A)	***C. milingae* group (*C. yaoi*)**
–	Tibia I with two pairs of ventral spines; bursae globular	**8**
8	Epigynal plate distinctly longer than wide, copulatory openings circular (Figs 80B, 88B), spermathecae oval (Fig. 96B)	***C. jiandan***
–	Epigynal plate distinctly wider than long, copulatory openings pocket-like (Figs 80C, 88C), spermathecae globular (Fig. 96C)	***C. wangchengi* sp. nov.**

#### Clubiona milingae

Taxon classificationAnimaliaAraneaeClubionidae

group

07247417-2447-5EC1-8931-3B303D986027

Clubiona apiculata group: Dankittipakul and Singtripop 2014: 1924.

##### Diagnosis.

See Dankittipakul and Singtripop (2014) and Yu and Li (2019a).

##### Description.

See Dankittipakul and Singtripop (2014).

##### Composition and distribution.

*Clubiona apiculata* Dankittipakul & Singtripop, 2014 (♂♀), *C. conica* Dankittipakul & Singtripop, 2014 (♂♀), *C. cylindriformis* Dankittipakul & Singtripop, 2014 (♂) and *C. cultrata* Dankittipakul & Singtripop, 2014 (♂) endemic to Borneo, *C. yaoi* Yu & Li, 2019 (♂♀) and *C. milingae* Barrion-Dupo, Barrion & Heong, 2013 (♂♀) from China.

##### Comments.

The *Clubiona milingae* group was established by Dankittipakul and Singtripop (2014) for four Borneo species. Yu and co-authors assigned two Chinese species to the species group (Yu and Li 2019; Zhang et al. 2020). The group presents a distinct set of characters, can be considered monophyletic, and may deserve the status of a separate genus in the future.

#### Clubiona yaoi

Taxon classificationAnimaliaAraneaeClubionidae

Yu & Li, 2019

0FA33ADC-8D7C-540F-BA42-050592D8C067

Figs 62A, B , 72A, B , 80A , 88A , 96A

Clubiona yaoi Yu & Li, 2019a: 152, figs 1A–E, 2A–H (♂♀).

##### Material examined.

***Types*.** Holotype ♂ (IZCAS Ar 34498), 1♀ (paratype, IZCAS Ar 34499), China: Yunnan Province: Xishuangbanna: Mengla County: Menglun Town: XTBG, secondary forest, 21°54.459'N, 101°16.755'E, ca. 644 m, 20.XI.2009, G. Tang and Z.Y. Yao leg. ***Other material examined.*** 1♂ (YHCLU0002), XTBG, leprosy village, 21°53.593'N, 101°17.329'E, ca. 559 m, 5.VIII.2018, H. Yu et al. leg.; 1♀ (YHCLU0003), XTBG, teak plantation, 21°54.117'N, 101°16.167'E, ca. 549 m, 8.VIII.2018, H. Yu et al. leg.

##### Diagnosis and description.

See Yu and Li (2019a). Male palp as in Figs 62A, B, 72A, B, epigyne as in Figs 80A, 88A, 96A.

##### Distribution.

Known only from Xishuangbanna.

##### Most similar species.

*Clubuiona milingae*.

#### Clubiona corticalis

Taxon classificationAnimaliaAraneaeClubionidae

group

A875642A-381D-58DF-8958-D1CBDDC6A0E7

Atalia Thorell, 1887: 54 (type species A. concinna Thorell, 1887).
Clubiona : Simon 1897: 76 (synonymised Atalia); Deeleman-Reinhold 2001: 90 (synonymised Paraclubiona).
Clubiona corticalis group: Simon 1932: 905; Mikhailov 1990: 142; Deeleman-Reinhold 2001: 90.
Paraclubiona Lohmander, 1944: 19 (type species Aranea corticalis Walckenaer, 1802).

##### Diagnosis.

See Mikhailov (1995), Deeleman-Reinhold (2001), and Yu and Li (2019a).

##### Description.

See Mikhailov (1995) and Deeleman-Reinhold (2001).

##### Composition and distribution.

Based on previous publications (Mikhailov 1995, 1998; Deeleman-Reinhold 2001; Ono and Hayashi 2009; Huang and Chen 2012; Wu et al. 2015; Yu and Li 2019a, b; Zhang and Yu 2020), at least 67 *Clubiona* species have been assigned to the *corticalis* group (from rows 1–67 in Table 3). A few other known species (from rows 68–73 in Table 3) resemble to some species in rows 1–67, but as no one indicated the group placement of these species, they are assigned tentatively to the *corticalis* group in the present paper for the lack of a better solution.

##### Comments.

At least two generic names are available for the *corticalis* group, *Atalia* Thorell, 1887 (type species *A. concinna*) and *Paraclubiona* Lohmander, 1944 (type species *C. corticalis*) (Zhang et al. 2018). The two taxa are currently considered junior synonyms of *Clubiona* (Wu et al. 2015; WSC 2021). The *corticalis* group is one of the most speciose clubionid groups and can be further divided into at least four or five subgroups based on morphological characters and molecular data (pers. obs.). We believe that the group deserves the status of a separate genus that can be further divided into several species groups in the future. A review of the genus *Clubiona* sensu lato and the *corticalis* group are not within the scope of this work.

Most species of the *C. corticalis* group are known from both sexes (Table 3); six species are known from males only: *C. fanjingshan*, *C. huiming*, and *C. subcylindrica* from Mt. Fanjing in Guizhou Province (1000 km from Xishuangbanna) and *C. lamina*, *C. aculeata*, and *C. tengchong* from northwest Yunnan (ca. 500 km from Xishuangbanna, Southeast Yunnan). We describe six new species known from females only in the present paper. In consideration of limited distribution ranges in almost all of the *corticalis* group species (Table 3), Xishuangbanna species are less likely to conspecific to the six species which are known from males only. None of our new species could be matched with *C. lamina*, *C. aculeata*, and *C. tengchong* due to their different habitus: Xishuangbanna species exhibit typical Southeast Asian *corticalis* group features, such as a lack of dark markings on the abdomen (Figs 2F, G, 15F, G, 18F, G, 22F, G, 23F, G, 24F, G) (vs. posteriorly with several chevron-shaped patterns dorsally on the abdomen of *C. lamina*, *C. aculeata*, and *C. tengchong*).

**Table 3.** *Clubiona corticalis* group species.

	Species name	Known sex3	Distribution
1	*C. aculeata* Zhang, Zhu & Song, 2007	♂	China (Yunnan)
2	*C. allotorta* Dankittipakul & Singtripop, 2008	♂♀	Thailand (Chiang Mai)
3	*C. alticola* Dankittipakul & Singtripop, 2008	♂♀	Thailand (Chiang Mai)
4	*C. altissimoides* Liu, Yan, Griswold & Ubick, 2007	♂♀	China (Yunnan)
5	*C. altissimus* Hu, 2001	♀	China (Xizang)
6	*C. applanata* Liu, Yan, Griswold & Ubick, 2007	♂♀	China (Yunnan)
7	*C. bandoi* Hayashi, 1995	♂♀	Japan (Shikoku)
8	*C. biforamina* Liu, Peng & Yan, 2016	♂♀	China (Yunnan)
9	*C. bifurcata* Zhang, Yu & Zhong, 2018	♂♀	China (Guizhou)
10	*C. bomiensis* Zhang & Zhu, 2009	♂♀	China (Xizang)
11	*C. boxaensis* Biswas & Biswas	♂♀	India (Jalpaiguri)
12	*C. brachyptera* Zhu & Chen, 2012	♂♀	China (Hainan)
13	*C. caohai* Zhang & Yu, 2020	♂♀	China (Guizhou)
14	*C. chakrabartei* Majumder & Tikader, 1991	♀	India (Uttarakhand)
15	*C. cirrosa* Ono, 1989	♂♀	Japan (Ryukyu Is.)
16	*C. cochlearis* Yu & Li, 2019	♂♀	China (Yunnan)
17	*C. cochleata* Wang, Wu & Zhang, 2015	♂♀	China (Yunnan)
18	*C. concinna* (Thorell, 1887)	♂♀	Myanmar (Tharrawaddy)
19	*C. cordata* Zhang & Zhu, 2009	♂♀	China (Sichuan, Xizang)
20	*C. corticalis* (Walckenaer, 1802)	♂♀	Europe, Turkey, Caucasus
21	*C. cylindrata* Liu, Yan, Griswold & Ubick, 2007	♂♀	China (Yunnan)
22	*C. dactylina* Liu, Peng & Yan, 2016	♂♀	China (Yunnan)
23	*C. dakong* Zhang & Yu, 2020	♀	China (Xiang)
24	*C. dichotoma* Wang, Chen & Z.S. Zhang, 2018	♂♀	China (Guizhou)
25	*C. didentata* Zhang & Yin, 1998	♂♀	China (Yunnan)
26	*C. falciforma* Liu, Peng & Yan, 2016	♂♀	China (Yunnan)
27	*C. fanjingshan* Wang, Chen & Z.S. Zhang, 2018	♂	China (Guizhou)
28	*C. femorocalcarata* Huang & Chen, 2012	♂♀	China (Taiwan)
29	*C. globosa* Wang, Chen & Z. S. Zhang, 2018	♂♀	China (Guizhou)
30	*C. gongshan* He, Liu & Zhang, 2016	♂♀	China (Yunnan)
31	*C. huiming* Wang, F. Zhang & Z. S. Zhang, 2018	♂	China (Guizhou)
32	*C. kai* Jäger & Dankittipakul, 2010	♂♀	Laos (Luang Prabang), China (Yunnan)
33	*C. kasanensis* Paik, 1990	♂♀	Korea (Gangwon, Gyeongsangbuk, Jeollabuk), Japan (Kojima)
34	*C. kayashimai* Ono, 1994	♀	China (Taiwan)
35	*C. kuanshanensis* Ono, 1994	♀	China (Taiwan)
36	*C. kurosawai* Ono, 1986	♀	China, Korea, Japan
37	*C. lamellaris* Zhang, Yu & Zhong, 2018	♂♀	China (Guizhou)
38	*C. lamina* Zhang, Zhu & Song, 2007	♂	China (Yunnan)
39	*C. lucida* He, Liu & Zhang, 2016	♂♀	China (Hunan)
40	*C. lyriformis* Song & Zhu, 1991	♀	China (Hubei)
41	*C. medog* Zhang, Zhu & Song, 2007	♀	China (Xizang)
42	*C. mikhailovi* Deeleman-Reinhold, 2001	♀	Indonesia (Java)
43	*C. moralis* Song & Zhu, 1991	♂♀	China (Yunnan, Hubei, Taiwan)
44	*C. multidentata* Liu, Peng & Yan, 2016	♂♀	China (Yunnan)
45	*C. parallela* Hu & Li, 1987	♂♀	China (Xizang)
46	*C. parconcinna* Deeleman-Reinhold, 2001	♂♀	Thailand (Nakhon Ratchasima), Indonesia (Borneo), China (Yunnan).
47	*C. pianmaensis* Wang, Wu & Zhang, 2015	♂♀	China (Yunnan)
48	*C. pollicaris* Wu, Zheng & Zhang, 2015	♂♀	China (Yunnan)
49	*C. pototanensis* Barrion & Litsinger, 1995	♀	Philippines (Panay Is.)
50	*C. pyrifera* Schenkel, 1936	♂♀	China (Gansu, Hubei)
51	*C. qiyunensis* Xu, Yang & Song, 2003	♂♀	China (Fujian, Anhui)
52	*C. rama* Dankittipakul & Singtripop, 2008	♂♀	India (Wes Bengal), Thailand (Phitsanulok), China (Yunnan)
53	*C. ryukyuensis* Ono, 1989	♂♀	Japan (Ryukyu Is.)
54	*C. stiligera* Deeleman-Reinhold, 2001	♂♀	Indonesia (Sumatra)
55	*C. subapplanata* Wang, Chen & Z.S. Zhang, 2018	♂♀	China (Guizhou)
56	*C. subcylindrica* Wang, Chen & Z.S. Zhang, 2018	♂	China (Guizhou)
57	*C. submoralis* Wu, Zheng & Zhang, 2015	♂♀	China (Yunnan)
58	*C. subrama* Yu & Li, 2019	♂♀	China (Yunnan)
59	*C. subyaginumai* Yu & Li, 2019	♂♀	China (Yunnan)
60	*C. taiwanica* Ono, 1994	♂♀	China (Taiwan)
61	*C. tangi* Liu, Peng & Yan, 2016	♂♀	China (Yunnan)
62	*C. tengchong* Zhang, Zhu & Song, 2007	♂	China (Yunnan)
63	*C. tiane* Yu & Li, 2019	♂♀	China (Yunnan)
64	*C. tortuosa* Zhang & Yin, 1998	♀	China (Yunnan)
65	*C. violaceovittata* Schenkel, 1936	♀	China (Gansu)
66	*C. yaginumai* Hayashi, 1989	♂♀	China (Taiwan), Japan (Honshu)
67	*C. yanzhii* Zhang & Yu, 2020	♀	China (Hunan)
68	*C. bucera* Yang, Ma & Zhang, 2011	♂♀	China (Yunnan)
69	*C. linzhiensis* Hu, 2001	♂♀	China (Xizang)
70	*C. ovalis* Zhang, 1991	♀	China (Fujian)
71	*C. pseudocordata* Dhali, Roy, Saha & Raychaudhuri, 2016	♀	India (West Bengal)
72	*C. wolongica* Zhu & An, 1999	♂♀	China (Anhui)
73	*C. zhangmuensis* Hu & Li, 1987	♂♀	China (Xizang)
74	*C. dengpao* Yu & Li, sp. nov.	♀	China (Yunnan)
75	*C. subdientata* Yu & Li, sp. nov.	♀	China (Yunnan)
76	*C. tixingi* Yu & Li, sp. nov.	♀	China (Yunnan)
77	*C. xiaoci* Yu & Li, sp. nov.	♂♀	China (Yunnan)
78	*C. xiaokong* Yu & Li, sp. nov.	♀	China (Yunnan)
79	*C. yejiei* Yu & Li, sp. nov.	♀	China (Yunnan)
80	*C. zhaoi* Yu & Li, sp. nov.	♀	China (Yunnan)
81	*C. zhigangi* Yu & Li, sp. nov.	♂♀	China (Yunnan)

#### Key to *C. corticalis* group species occurring in Xishuangbanna (males)

Males of *C. dengpao* sp. nov., *C. subdidentata* sp. nov., *C. tixing* sp. nov., *C. xiaokong* sp. nov., *C. yejiei* sp. nov. and *C. zhaoi* sp. nov. are excluded due to a lack of specimens.

**Table:** 

1	Palp with femoral apophysis (Figs 56C, 66C)	***C. pollicaris***
–	Palpal femur unmodified	**2**
2	Palp with patellar apophysis (Figs 65A–D, 66B)	**3**
–	Palpal patella unmodified	**7**
3	Palpal tibia retrolaterally with several short, modified spines near the base (Figs 55D, 65C, D)	**4**
–	Palpal tibia without spines	**5**
4	Conductor absent; retrolateral patellar apophysis with short, modified spines (Figs 55D, 65D)	***C. xiaoci* sp. nov.**
–	Conductor distinct; retrolateral patellar apophysis without short, modified spines (Figs 55C, 65C)	***C. parconcinna***
5	Patellar retrolateral apophysis represented by a small conoid, patella with a row of longitudinally arranged teeth in retrolateral view (Fig. 66B)	***C. multidentata***
–	Patellar retrolateral apophysis with an indented tip; patella retrolaterally without small tooth	**6**
6	Embolus wide and triangular (Fig. 55B)	***C. submoralis***
–	Embolus claw-like and curved (Fig. 55A)	***C. moralis***
7	Palpal tibia with single retrolateral apophysis (Figs 64A–D, 66A)	**8**
–	Palpal tibia with 2 retrolateral apophyses (Fig. 63A–D)	**12**
8	Bulb proximally with an apophysis (Fig. 56A); retrolateral tibial apophysis with thin distally and wide basally (Fig. 66A)	***C. kurosawai***
–	Bulb proximally without apophysis (Fig. 54A–D); retrolateral tibial apophysis not subdivided (Fig. 64A–D)	**9**
9	Both conductor and tegular apophysis present (Figs 54A, B, 64A, B)	**10**
–	Both conductor and tegular apophysis absent (Figs 54C, D, 64C, D)	**11**
10	Embolus twisted, distinctly longer than conductor; conductor papilliform with membranous tip; tegular apophysis distinctly smaller than embolus, tooth-shaped, directed prolatero-distally (Figs 54B, 64B)	***C. kai***
–	Embolus slightly curved, approximately as long as conductor; conductor linguiform and heavily sclerotised; tegular apophysis almost the same size as embolus, triangular, directed distally (Figs 54A, 64A)	***C. didentata***
11	Embolus curved and neck of a swan-shaped (Fig. 54D); retrolateral tibial apophysis shaped like dorsal fin of a fish (Fig. 64D)	***C. tiane***
–	Embolus straight and needle-shaped (Fig. 54C); retrolateral tibial apophysis thumb-like (Fig. 64C)	***C. subyaginumai***
12	Embolus strong, spoon-shaped, with expanded, torsional tip (Figs 53A, 63A)	***C. cochlearis***
–	Embolus slender and filiform (Figs 53B–D, 63B–D)	**13**
13	Embolar apex sinuate; conductor short, ca. 1/5 of tegulum length, with a blunt tip (Figs 53D, 63D)	***C. zhigangi* sp. nov.**
–	Embolar tip not curved; conductor long, not less than 1/3 of tegulum length, with a sharply pointed tip (Figs 53B, C, 63B, C)	**14**
14	Retrolateral tibial apophysis with sharp apex; ventral tibial apophysis trapezoidal, with blunt tip (Fig. 63C)	***C. subrama***
–	Retrolateral tibial apophysis apically indented; ventral tibial apophysis subtriangular, with sharp tip (Fig. 63B)	***C. rama***

#### Key to *C. corticalis* group species occurring in Xishuangbanna (females)

*C. rama* is excluded due to lack of specimens.

**Table:** 

1	Copulatory openings located in the centre of epigynal plate (Figs 76B, 84B)	***C. pollicaris***
–	Copulatory openings located at anterior part of epigynal plate	**2**
2	Spermathecae tubular and sinuous, spermatheca with head (Figs 89A–F, 90A, D, 92A)	**3**
–	Spermathecae not as above	**11**
3	Atrium not less than 1/3 of epigyne width (Figs 73A–D, 81A–D)	**4**
–	Atrium reduced, relatively small, less than 1/3 of epigyne width (Figs 73E, F, 74A, D, 76A, 81E, F, 82A, D, 84A)	**7**
4	Atrium with atrial membrane on anterior margin (Figs 73D, 81D)	***C. tixing* sp. nov.**
	Atrium without atrial membrane (Figs 73A–C, 81A–C)	**5**
5	Copulatory openings large, diameter as long as atrium length, situated laterally in atrium (Figs 73A, 81A); copulatory ducts and spermathecae thin and strongly twisted (Fig. 89A)	***C. cochlearis***
–	Copulatory openings small, diameter ca. 1/3 of atrium length, located posteriorly in atrium (Figs 73B, C, 81B, C); copulatory ducts and spermathecae thick and slightly twisted (Fig. 89B, C)	**6**
6	Atrium light bulb-shaped (Figs 73B, 81B)	***C. dengpao* sp. nov.**
–	Atrium ellipsoid (Figs 73C, 81C)	***C. yejiei* sp. nov.**
7	Atrium reduced (Figs 76A, 84A); bursae globular (Fig. 92A)	***C. kurosawai***
–	Atrium present (Figs 73E, F, 74A, D, 81E, F, 82A, D); bursae ovoid or reniform	**8**
8	Atrium narrowed, anteriorly cordiform, posteriorly elongate (Figs 73E, F, 81E, F)	**9**
–	Atrium not as above	**10**
9	Copulatory ducts distinctly long, with a long course forming 2 loops before entering spermathecae (Fig. 89E)	***C. subrama***
–	Copulatory duct relatively short, directly connected to spermathecae (Fig. 89F)	***C. zhigangi* sp. nov.**
10	Copulatory ducts thick, heavily sclerotised (Fig. 90A)	***C. xiakong* sp. nov.**
–	Copulatory duct indistinct, almost invisible in dorsal view (Fig. 90D)	***C. tiane***
11	Atrium with atrial membrane on anterior margin (Figs 74C, E, F, 82 C, E, F)	**12**
–	Atrial membrane absent (Figs 74B, 75A–F, 82B, 83A–F)	**14**
12	Atrial membrane disc-shaped (Figs 74C, 82C); bursae sclerotised (Fig. 90C)	***C. kai***
–	Atrial membrane subtriangular (Figs 74E, F, 82E, F); bursae membranous (Fig. 90E, F)	**13**
13	Atrial membrane tongue-shaped (Figs 74E, 82E); copulatory ducts absent (Fig. 90E)	***C. didentata***
–	Atrial membrane equilateral triangle (Figs 74F, 82F); copulatory ducts present (Fig. 90F)	***C. subdidentata* sp. nov.**
14	Copulatory openings widely separated by ca. 2.5–3.0 diameters (Figs 75D, 83D)	***C. multidentata***
–	Copulatory openings partly fused or close together, separated by not more than one diameter (Figs 74B, 75A–C, E, F, 82B, 83A–C, E, F)	**15**
15	Spermathecae subtriangular (Figs 90B, 91F)	**16**
–	Spermathecae almost spherical (Fig. 91A–C, E)	**17**
16	Atrial anterior margin M-shaped (Figs 74B, 82B); copulatory ducts indistinct (Fig. 90B)	***C. zhaoi* sp. nov.**
–	Atrial anterior margin transverse (Figs 75F, 83F); copulatory ducts longer than spermathecae diameter (Fig. 91F)	***C. subyaginumai***
17	Epigynal plate anteriorly with a nearly horizontal K-shaped sclerite (Fig. 75A, B)	**18**
–	Epigynal plate not as above; atrial anterior margin cambered (Fig. 75C, E)	**19**
18	Copulatory openings separated by ca. one diameter (Figs 75A, 83A); copulatory ducts descend obliquely (Fig. 91A); bursae 2 × longer than the spermathecae (Fig. 91A)	***C. moralis***
–	Copulatory openings close together (Figs 75B, 83B); copulatory ducts descend longitudinally (Fig. 91B); spermathecae almost as large as bursae (Fig. 91B)	***C. submoralis***
19	Copulatory openings separated (Figs 75C, 83C); copulatory ducts thin, diameter ca. 1/4 of spermathecae, descend longitudinally (Fig. 91C); bursae surface relatively smooth (Fig. 91C)	***C. parconcinna***
–	Copulatory openings partly fused (Figs 75E, 83E); copulatory ducts thick, ca. the same diameter as spermathecae, descend obliquely (Fig. 91E); bursae surface wrinkled (Fig. 91E)	***C. xiaoci* sp. nov.**

#### Clubiona cochlearis

Taxon classificationAnimaliaAraneaeClubionidae

Yu & Li, 2019

17F5C4EF-C79C-5D1A-A3A9-A963C302289B

**Figure 1.**
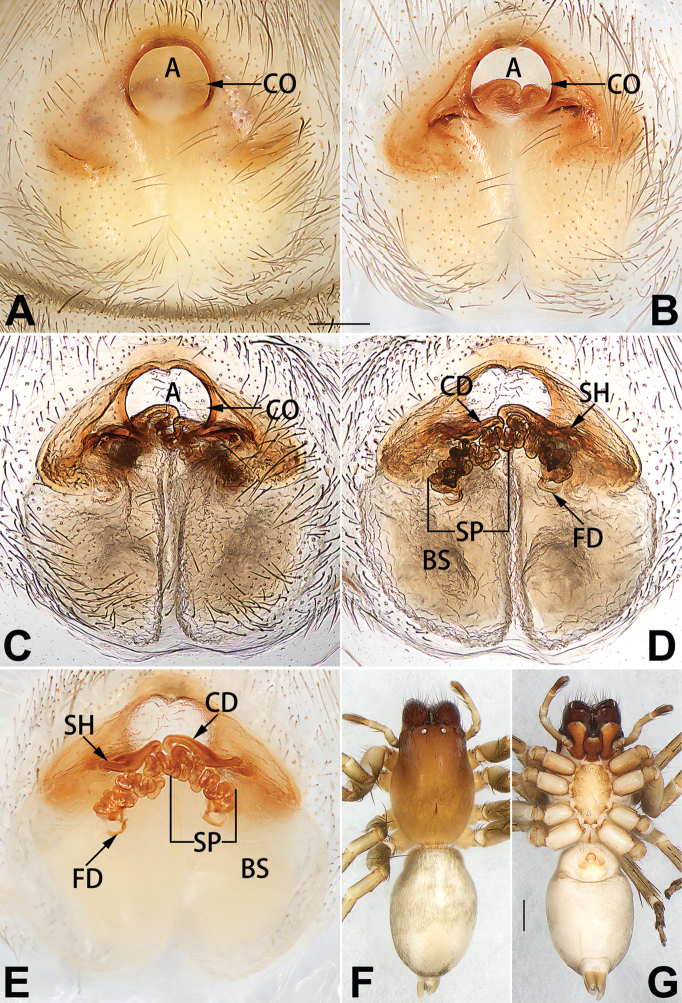
*Clubiona cochlearis*, epigyne (**A–E**) and female habitus (**F, G**) **A** intact, ventral view **B** cleared, ventral view **C** cleared, ventral view **D** cleared, dorsal view **E** cleared, dorsal view **F** dorsal view **G** ventral view. Abbreviations: A = atrium; BS = bursa; CD = copulatory duct; CO = copulatory opening; FD = fertilisation duct; SH = spermathecal head; SP = spermatheca. Scale bars: 0.2 mm (equal for **A–E**); 1 mm (equal for **F**, **G**).

Figs 1 , 53A , 63A , 73A , 81A , 89A

Clubiona cochlearis Yu & Li, 2019b: 202, figs 1A–E, 2A–C (♂).

##### Material examined.

***Types*.** Holotype ♂ (IZCAS Ar 34701), China: Yunnan Province: Xishuangbanna: Mengla County: Menglun Town: XTBG, *Paramichelia baillonii*plantation, 21°54.772'N, 101°16.043'E, ca. 556 m, 18.VII.2007, G. Zheng leg. ***Other material examined.*** 1♀, Jinghong City: Pingguan Town, monsoon forest, 22°13.212'N, 100°53.151'E, ca. 832 m, 21.VII.2012, Q.Y. Zhao and C.X. Gao leg; 1♂ (YHCLU0068), Mengyang County: monsoon forest, 21°54.117'N, 101°55.210'E, ca. 856 m, 16.VII.2012, Q.Y. Zhao and C.X. Gao leg; 1♀ (YHCLU0079), Jinghong City: Pingguan Town, monsoon forest, 22°13.668'N, 100°53.351'E, ca. 888 m, 20.VII.2012, Q.Y. Zhao and C.X. Gao leg.

##### Diagnosis.

Females of *C. cochlearis* are similar to those of *C. lyriformis* (Yin et al. 2012: 1110, fig. 586a–c). The two species share a similarly large atrium, tubular and sinuous spermathecae, and the copulatory ducts are proximally wide and distally narrow. They differ in the following: (1) atrium is nearly apple-shaped in *C. cochlearis* (Figs 1A–C, 73A, 81A) (vs. atrium shaped like a violin in *C. lyriformis*; Yin et al. 2012: fig. 586b); (2) size and location of copulatory openings (copulatory openings are larger and situated laterally in atrium in *C. cochlearis* vs. relatively small and located at basolateral atrial borders in *C. lyriformis*) (cf. Figs 1A–C, 73A, 81A and Yin et al. 2012: fig. 586c). Males of *C. cochlearis* can be easily recognised by the robust, spoon-shaped embolus (Figs 53A, 63A) from all others in the species group.

##### Description.

**Male.** See Yu and Li (2019b). Male palp as in Figs 53A, 63A.

**Female** (Fig. 1F, G). Total length 7.38; carapace 3.64 long, 2.61 wide; opisthosoma 3.75 long, 2.53 wide. Carapace brown, darker in cephalic area, without distinct pattern; cephalic region slightly narrowed, cervical groove indistinct; tegument smooth, with short, fine setae. Eyes: AER slightly recurved, PER slightly wider than AER, almost straight in dorsal view. Eye sizes and interdistances: AME 0.16, ALE 0.10, PME 0.13, PLE 0.15, AME–AME 0.21, AME–ALE 0.18, PME–PME 0.49, PME–PLE 0.38, MOQL 0.40, MOQA 0.49, MOQP 0.76. Chelicerae robust and brownish red, with four promarginal teeth and two retromarginal denticles. Sternum pale brown, 1.86 long, 1.24 wide. Labium and endites coloured as carapace. Legs light brown, without distinct markings. Leg measurements: I 7.18 (2.20, 2.88, 1.33, 0.77), II 7.66 (12.21, 3.12, 1.47, 0.85), III 7.11 (2.34, 2.53, 1.60, 0.65), IV 9.65 (2.93, 3.32, 2.61, 0.78). Abdomen oval, dorsally grey with dense setae and a lengthwise white heart mark reaching posterior half; with a pair of muscle depressions located at distal part of heart mark; venter uniformly white, without pattern.

Epigyne (Figs 1A–E, 73A, 81A, 89A). Epigynal plate nearly as broad as long, spermathecae and bursae indistinctly visible through integument. Atrium ca. 1/3 of epigyne length and width, more or less apple-shaped, slightly concave anteromedially. Copulatory openings large, located at lateral atrial borders. Copulatory ducts long, proximally thick-walled, extend posteriorly, the latter half slender, ascending obliquely, then connecting with spermathecae at central axis of the vulva. Spermathecae tubular, long and sinuous, strongly convoluted. Fertilisation ducts short and curved, acicular. Bursae situated posteriorly, ovoid, relatively large, close together, ca. 1.5 × longer than wide, surface translucent and smooth.

##### Distribution.

Known only from Xishuangbanna.

##### Remarks.

The female of the species is described for the first time.

#### Clubiona dengpao

Taxon classificationAnimaliaAraneaeClubionidae

Yu & Li sp. nov.

D8CFF7E2-DDA7-5FF6-989A-D3AA7DCA8792

http://zoobank.org/63A97FF6-8968-4584-BDBA-536500AA7479

**Figure 2.**
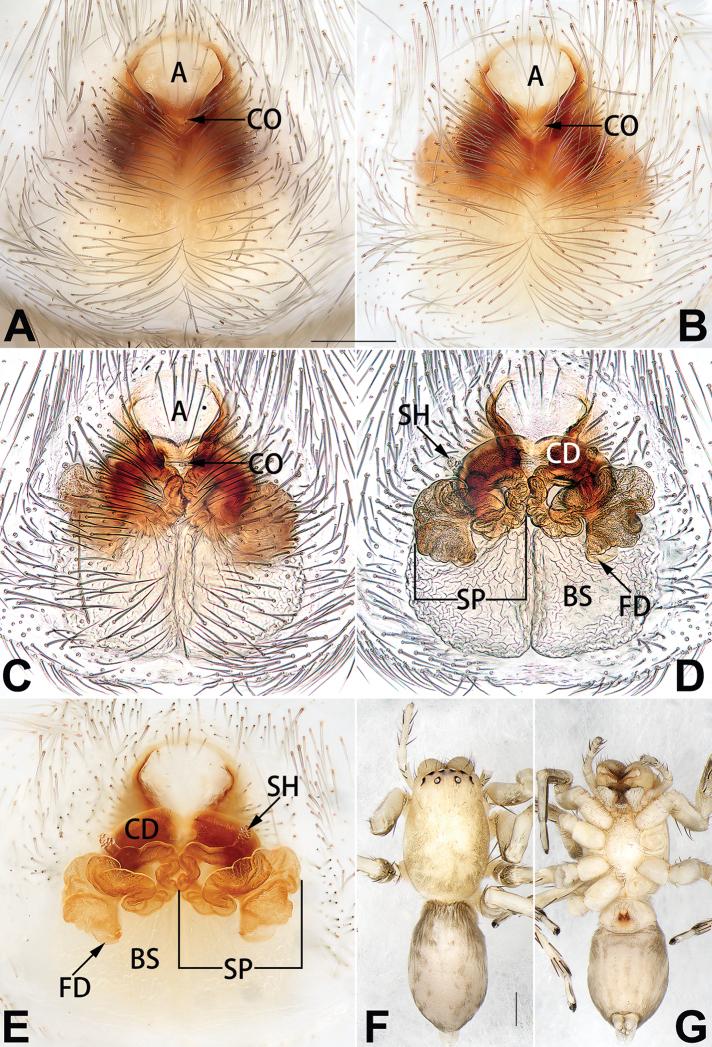
Holotype female of *Clubiona dengpao* sp. nov., epigyne (**A–E**) and habitus (**F, G**) **A** intact, ventral view **B** cleared, ventral view **C** cleared, ventral view **D** cleared, dorsal view **E** cleared, dorsal view **F** dorsal view **G** ventral view. Abbreviations: A = atrium; BS = bursa; CD = copulatory duct; CO = copulatory opening; FD = fertilisation duct; SH = spermathecal head; SP = spermatheca. Scale bars: 0.2 mm (equal for **A–E**); 1 mm (equal for **F, G**).

Figs 2 , 73B , 81B , 89B

##### Holotype.

♀ (IZCAS-Ar 34748), China: Yunnan Province: Xishuangbanna: Jinghong City: Menga Town: Wengnan Village: secondary forest, 21°24.265'N, 101°37.296'E, ca. 693 m, 28.VI.2012, Q.Y. Zhao and C.X. Gao leg. ***Other material examined.*** 1♀ (YHCLU0080), Mengla County: Nanshahe Village: monsoon forest, 21°36.200'N, 101°34.384'E, ca. 826 m, 14.VII.2012, Q.Y. Zhao and C.X. Gao leg.

##### Etymology.

The specific name is derived from the Chinese pinyin *dēng pào*, which means ‘lamp bulb’, referring to the atrium which is shaped like a light bulb; noun in apposition.

##### Diagnosis.

This new species is similar to *C. cochlearis* (Figs 1A–E, 73A, 81A, 89A) and *C. yejiei* sp. nov. (Figs 23A–E, 73C, 81C, 89C) in having a large atrium and long, tubular and sinuous spermathecae but can be easily distinguished by the light bulb-shaped atrium (Figs 2A–C, 73B, 81B) (vs. apple-shaped in *C. cochlearis* and ellipsoid in *C. yejiei*; Figs 1A–C, 73A, 81A, 23A–C, 73C, 81C), and by lacking a fovea (Fig. 2F) (vs. fovea present in *C. cochlearis* and *C. yejiei*; Figs 1F, 23F).

##### Description.

**Female.** Holotype (Fig. 2F, G): Total length 7.86; carapace 3.98 long, 2.51 wide; opisthosoma 3.87 long, 2.36 wide. Carapace greyish white, without pattern, fovea absent; pars cephalica slightly narrowed, cervical groove and radial grooves indistinguishable; tegument smooth, marginally clothed with dense setae. Eyes: AER slightly recurved, PER slightly wider than AER, almost straight in dorsal view. Eye sizes and interdistances: AME 0.11, ALE 0.19, PME 0.18, PLE 0.14, AME–AME 0.18, AME–ALE 0.15, PME–PME 0.44, PME–PLE 0.28, MOQL 0.52, MOQA 0.49, MOQP 0.79. Chelicerae coloured as carapace, with four teeth on promargin and three teeth on retromargin. Sternum nearly pure white, 1.99 long, 1.15 wide. Labium and endites coloured as carapace, sparsely covered with setae. Legs coloured as carapace, dorsally covered with black setae, without markings. Leg measurements: I 6.85 (2.01, 2.67, 1.33, 0.85), II 7.21 (2.19, 2.82, 1.45, 0.75), III 6.57 (2.07, 2.22, 1.64, 0.65), IV 8.94 (2.65, 2.79, 2.58, 0.93). Abdomen oval, dorsum light grey, anteriorly and marginally with conspicuous tuft of brown setae, heart mark and muscle depressions indistinct; venter coloured as dorsum, without pattern.

Epigyne (Figs 2A–E, 73B, 81B, 89B). Epigynal plate slightly longer than wide, margin not rebordered; copulatory ducts, spermathecae and bursae indistinctly visible through transparent integument. Atrium large, shaped like a tungsten lamp bulb, ca. 1.2 × longer than wide, ca. 1/3 of epigyne length. Copulatory openings indistinct, close together, located at posterior atrial margin. Copulatory ducts short and thick, heavily sclerotised, descend obliquely, then connect to spermathecae. Spermathecae long and wrinkled, consisting of tubular proximal part and sac-like distal part, with thin fertilisation ducts terminally. Bursae large, ovoid, 1.3 × longer than wide, surface translucent and wrinkled.

**Male.** Unknown.

##### Distribution.

Known only from the type locality, Xishuangbanna, Yunnan, China.

#### Clubiona didentata

Taxon classificationAnimaliaAraneaeClubionidae

Zhang & Yin, 1998

234F835B-2AB2-59B5-93FF-F2D77D8116C3

Figs 54A , 64A , 74E , 82E , 90E

Clubiona didentata Zhang & Yin, 1998: 11, figs 6–8 (♂); Yu and Li 2019b: 207, figs 5A–E, 6A–G (♂♀).

##### Material examined.

1♂ (YHCLU0015), 1♀ (YHCLU0016), China: Yunnan Province: Xishuangbanna: Mengla County: Menglun Town: XTBG, flower garden, 21°55.915'N, 101°15.099'E, ca. 550 m, 18.VII.2018, X.Q Mi et al. leg; 1♂, XTBG, low evergreen forest, 21°53.794'N, 101°17.152'E, ca. 594 m, 27.XI.2009, G. Tang and Z.Y. Yao leg; 2♀, XTBG, bamboo plantation, 21°53.640'N, 101°16.940'E, ca. 580 m, 3.XII.2009, G. Tang and Z.Y. Yao leg; 1♂3♀, XTBG, rubber-tea plantation, 21°55.239'N, 101°15.854'E, ca. 572 m, 28.VII.2018, Z.G. Chen et al. leg.

##### Diagnosis and description.

See Yu and Li (2019b). Male palp as in Figs 54A, 64A, epigyne as in Figs 74E, 82E, 90E.

##### Distribution.

Known only from Xishuangbanna.

##### Most similar species.

*Clubiona kai*.

#### Clubiona kai

Taxon classificationAnimaliaAraneaeClubionidae

Jäger & Dankittipakul, 2010

1F0298CF-F5B6-5FD1-8710-2F5303FD4FE1

Figs 54B , 64B , 74C , 82C , 90C

Clubiona kai Jäger & Dankittipakul, 2010: 25, figs 4–12 (♂); Yu and Li 2019b: 207, figs 7A–E, 8A–G (♂♀).

##### Material examined.

1♀ (YHCLU0052), China: Yunnan Province: Xishuangbanna: Mengla County: Menglun Town: XTBG, 300 acre-feet teak plantation, 21°54.033'N, 101°16.400'E, ca. 554 m, 10.VIII.2018, Z.G. Chen et al. leg; 1♂ (YHCLU0259), XTBG, *Anogeissus acuminata*plantation, 21°53.992'N, 101°16.948'E, ca. 596 m, 9.V.2019, Z.L. Bai et al. leg; 1♂, XTBG, Lvshilin Forest Park, limestone tropical seasonal rainforest, 21°54.714'N, 101°16.953'E, ca. 660 m, 16.XI.2009, G. Tang and Z.Y. Yao leg; 3♂7♀, XTBG, Lvshilin Forest Park, limestone tropical seasonal rainforest, 21°54.555'N, 101°16.860'E, ca. 610 m, 29.XI.2009, G. Tang and Z.Y. Yao leg.

##### Diagnosis and description.

See Yu and Li (2019b). Male palp as in Figs 54B, 64B, epigyne as in Figs 74C, 82C, 90C.

##### Distribution.

Laos (Luang Prabang), China (Yunnan).

##### Most similar species.

*Clubiona didentata*.

#### Clubiona kurosawai

Taxon classificationAnimaliaAraneaeClubionidae

Ono, 1986

BBCD68C7-3C1A-5396-BB5D-7C3130473D0A

**Figure 3.**
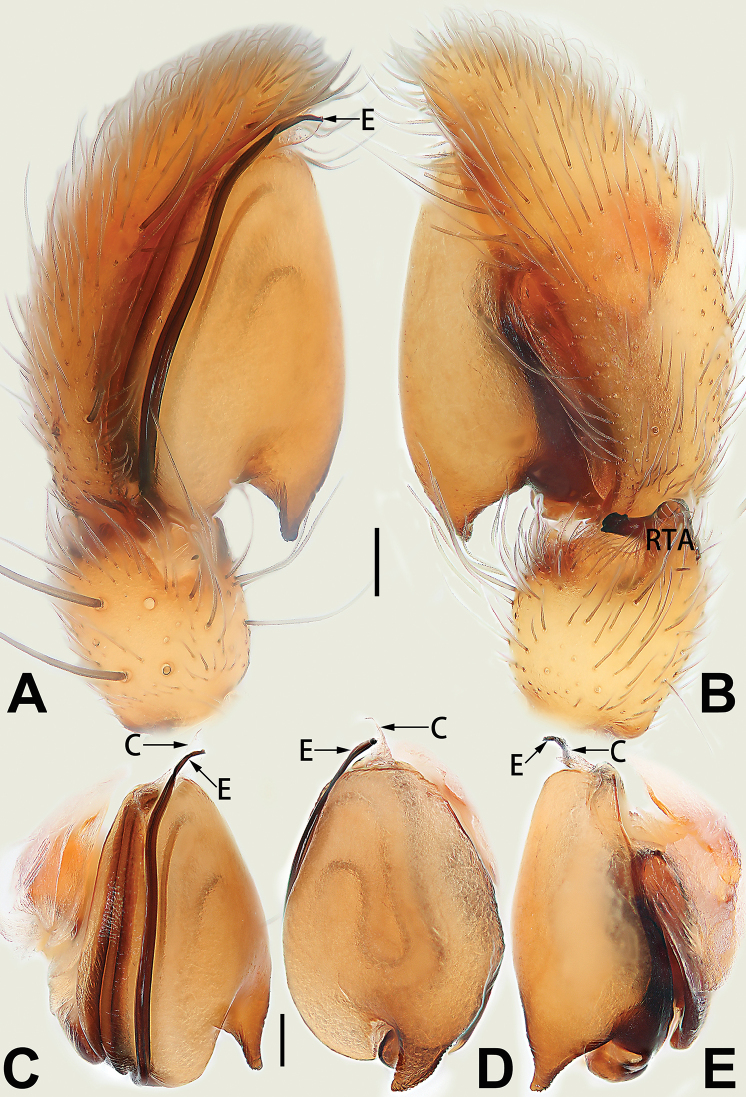
Male palp of *Clubiona kurosawai***A** prolateral view **B** retrolateral view **C** bulb, prolateral view **D** bulb, ventral view **E** bulb, retrolateral view. Abbreviations: C = conductor; E = embolus; RTA = retrolateral tibial apophysis. Scale bars: 0.1 mm (equal for **A, B**, equal for **C–E**).

**Figure 4.**
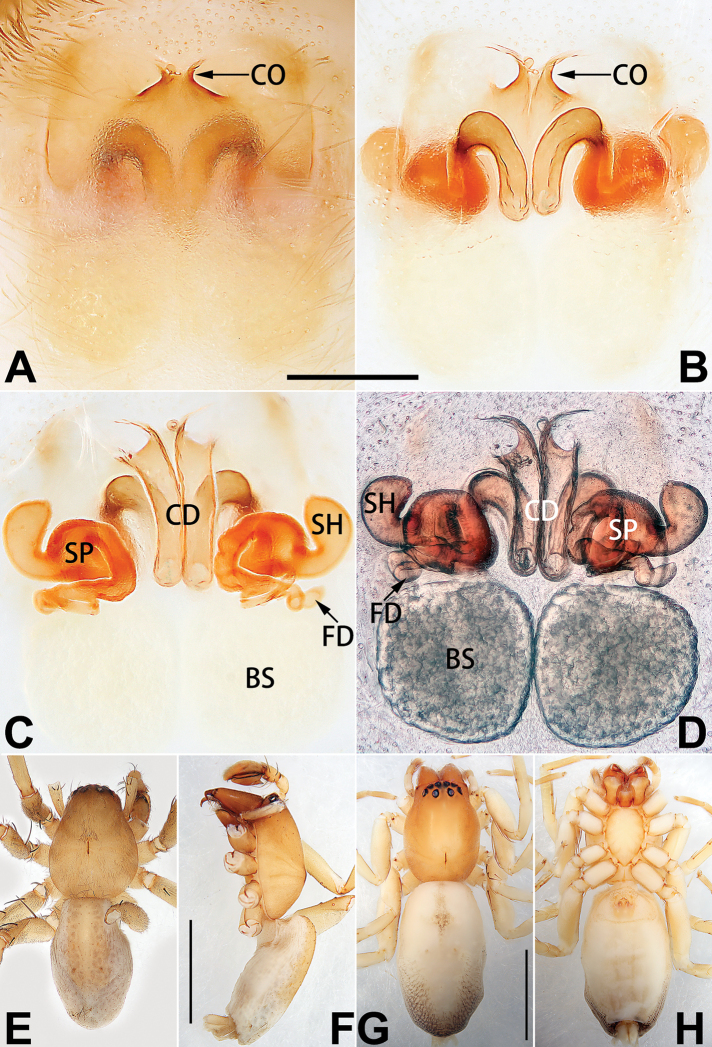
*Clubiona kurosawai*, epigyne (**A–D**), male habitus (**E, F**) and female habitus (**G, H**) **A** intact, ventral view **B** cleared, ventral view **C** cleared, dorsal view **D** cleared, dorsal view **E** dorsal view **F** Lateral view **G** dorsal view **H** ventral view. Abbreviations: BS = bursa; CD = copulatory duct; CO = copulatory opening; FD = fertilisation duct; SH = spermathecal head; SP = spermatheca. Scale bars: 0.2 mm (equal for **A–D**); 2 mm (equal for **E, F**, equal for **G, H**).

Figs 3 , 4 , 56A , 66A , 76A , 84A , 92A

Clubiona kurosawai Ono, 1986: 20, figs 1–8 (♂♀); Mikhailov 1995: 34, fig. 3 (♂); Huang and Chen 2012: 77, figs 22A–F, pl. 6C–D, 7A–B, box 2D (♂♀). For full list of taxonomic references see WSC (2021).

##### Material examined.

1♂, 1♀, China: Yunnan Province: Xishuangbanna: Mengla County: Menglun Town: XTBG, rubber plantation, 21°54.350'N, 101°16.461'E, ca. 614 m, 11.VIII.2007, G. Zheng leg.

##### Diagnosis and description.

See Ono (1986). Male palp as in Figs 3, 56A, 66A, epigyne as in Figs 4A–D, 66A, 84A, 92A, habitus as in Fig. 4E–H.

##### Distribution.

Korea, Japan (from Honshu to Southwest Islands), China (Yunnan, Taiwan).

##### Most similar species.

*Clubiona bucera*.

#### Clubiona moralis

Taxon classificationAnimaliaAraneaeClubionidae

Song & Zhu, 1991

99AC1803-DF52-511B-ADAD-4D46950A4065

**Figure 5.**
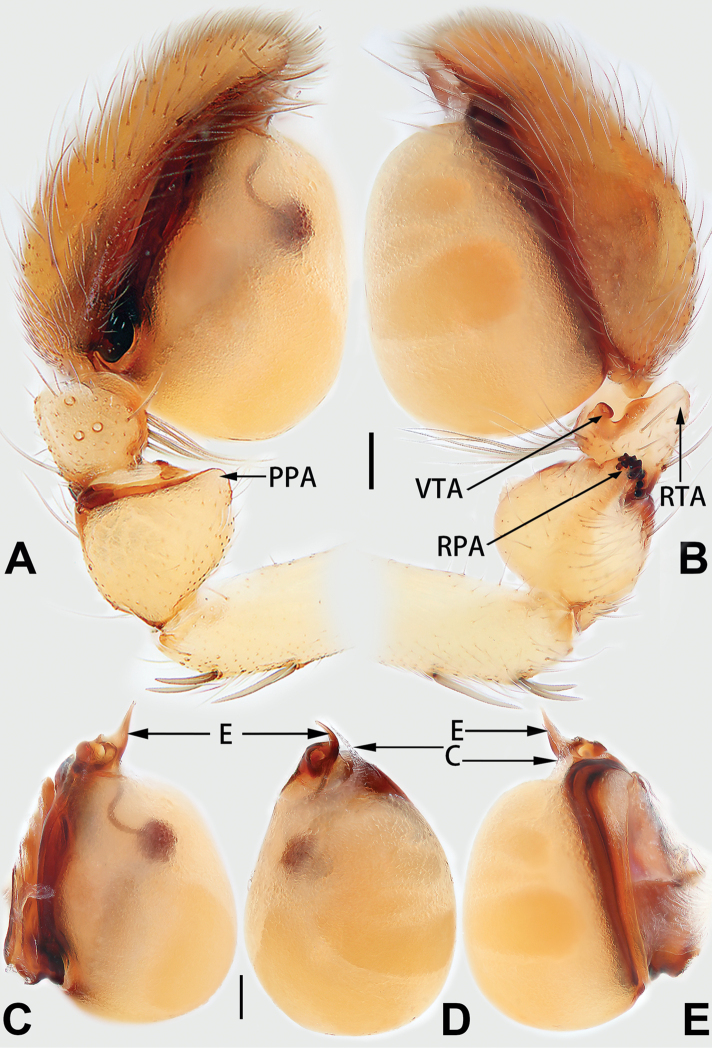
Male palp of *Clubiona moralis***A** prolateral view **B** retrolateral view **C** bulb, prolateral view **D** bulb, ventral view **E** bulb, retrolateral view. Abbreviations: C = conductor; E = embolus; PPA = prolateral patellar apophysis; RPA = retrolateral patellar apophysis; RTA = retrolateral tibial apophysis; VTA = ventral tibial apophysis. Scale bars: 0.1 mm (equal for **A, B**, equal for **C–E**).

**Figure 6.**
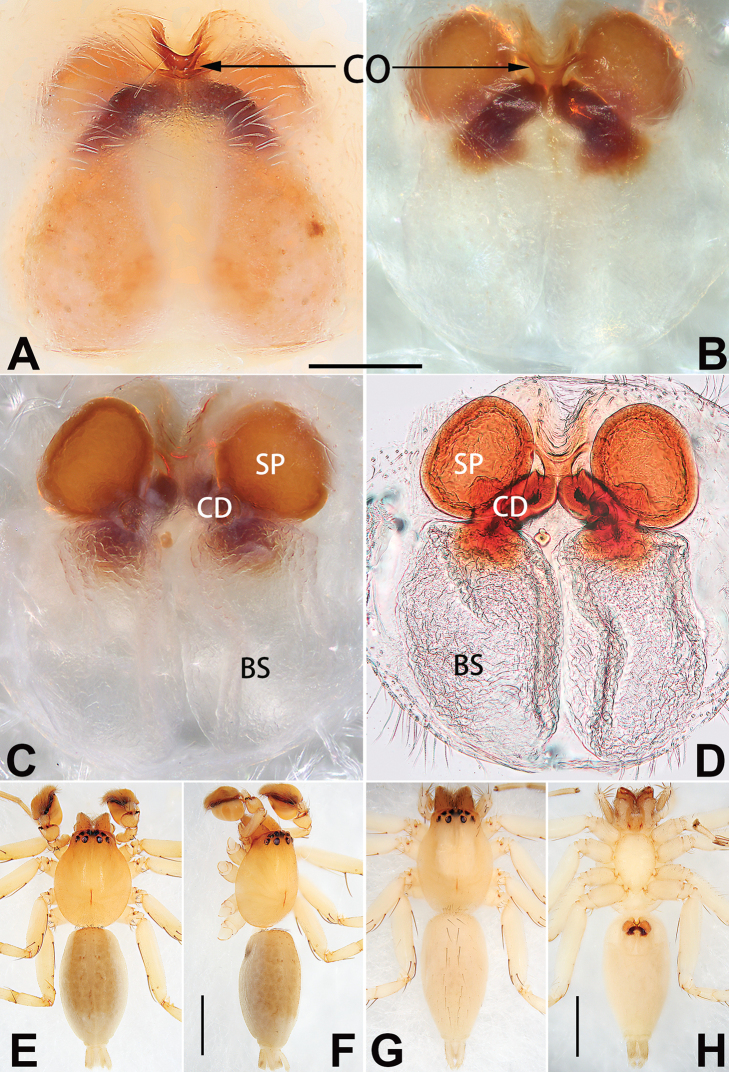
*Clubiona moralis*, epigyne (**A–D**), male habitus (**E, F**) and female habitus (**G, H**) **A** intact, ventral view **B** cleared, ventral view **C** cleared, dorsal view **D** cleared, dorsal view **E** dorsal view **F** lateral view **G** dorsal view **H** ventral view. Abbreviations: BS = bursa; CD = copulatory duct; CO = copulatory opening; SP = spermatheca. Scale bars: 0.2 mm (equal for **A–D**); 1 mm (equal for **E, F**, equal for **G, H**).

Figs 5 , 6 , 55A , 65A , 75A , 83A , 91A

Clubiona moralis Song & Zhu, in Song et al. 1991: 70, fig. 5A–D (♂♀); Song and Li 1997: 429, fig. 24A–D (♂♀); Song et al 1999: 426, figs 250M–N, 252Q–R (♂♀); Huang and Chen 2012: 80, fig. 23A–F, pl. 7C (♂♀).

##### Material examined.

1♂ (YHCLU0025), China: Yunnan Province: Xishuangbanna: Mengla County: Menglun Town: XTBG, *Paramichelia baillonii*plantation, 21°54.183'N, 101°16.967'E, ca. 596 m, 1.VIII.2018, Z.G. Chen leg; 1♀ (YHCLU0024), XTBG, secondary tropical forest, 21°54.833'N, 101°16.781'E, ca. 575 m, 31.VII.2018, Z.G. Chen leg; 4♂6♀, XTBG, *Anogeissus acuminata*plantation, 21°53.992'N, 101°16.948'E, ca. 596 m, 2.XII.2009, G. Tang and Z.Y. Yao leg.

##### Diagnosis and description.

See Huang and Chen (2012). Male palp as in Figs 5, 55A, 65A, epigyne as in Figs 6A–D, 75A, 83A, 91A, habitus as in Fig. 6E–H.

##### Distribution.

China (Yunnan, Hubei, Taiwan).

##### Most similar species.

*Clubiona submoralis*.

#### Clubiona multidentata

Taxon classificationAnimaliaAraneaeClubionidae

Liu, Peng & Yan, 2016

D6507CB7-24B8-5946-B0E4-FACDD9B8B0B3

**Figure 7.**
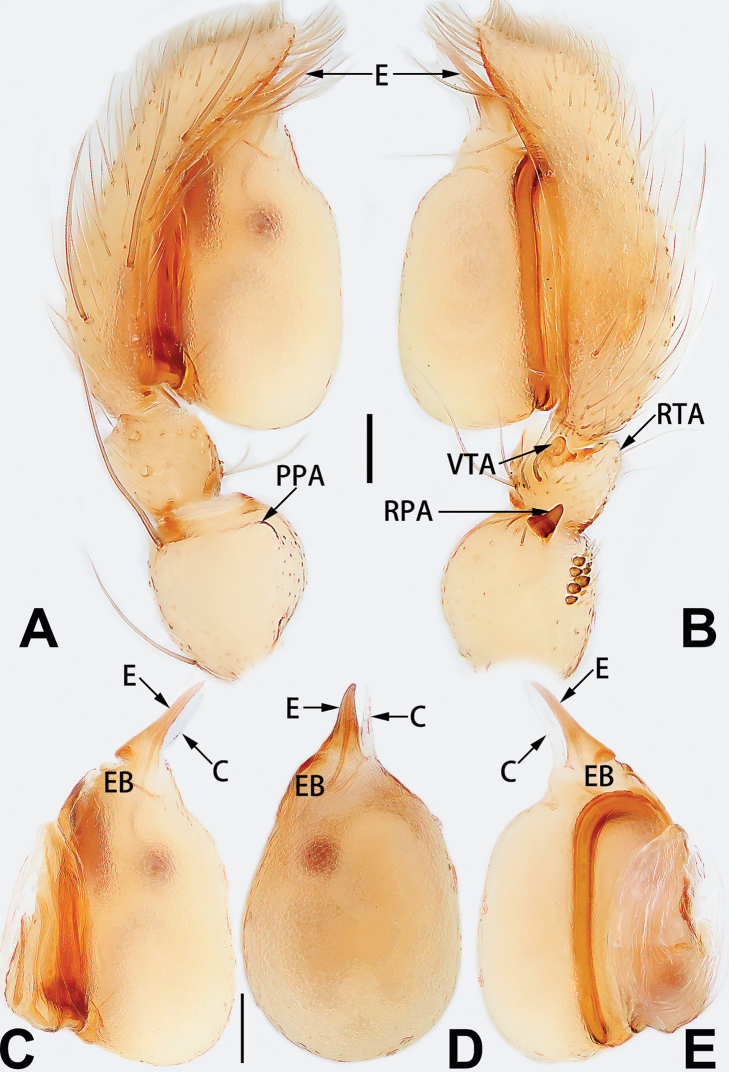
Male palp of *Clubiona multidentata*. **A** prolateral view **B** retrolateral view **C** bulb, prolateral view **D** bulb, ventral view **E** bulb, retrolateral view. Abbreviations: C = conductor; E = embolus; EB = embolar base; PPA = prolateral patellar apophysis; RPA = retrolateral patellar apophysis; RTA = retrolateral tibial apophysis; VTA = ventral tibial apophysis. Scale bars: 0.1 mm (equal for **A, B**, equal for **C–E**).

**Figure 8.**
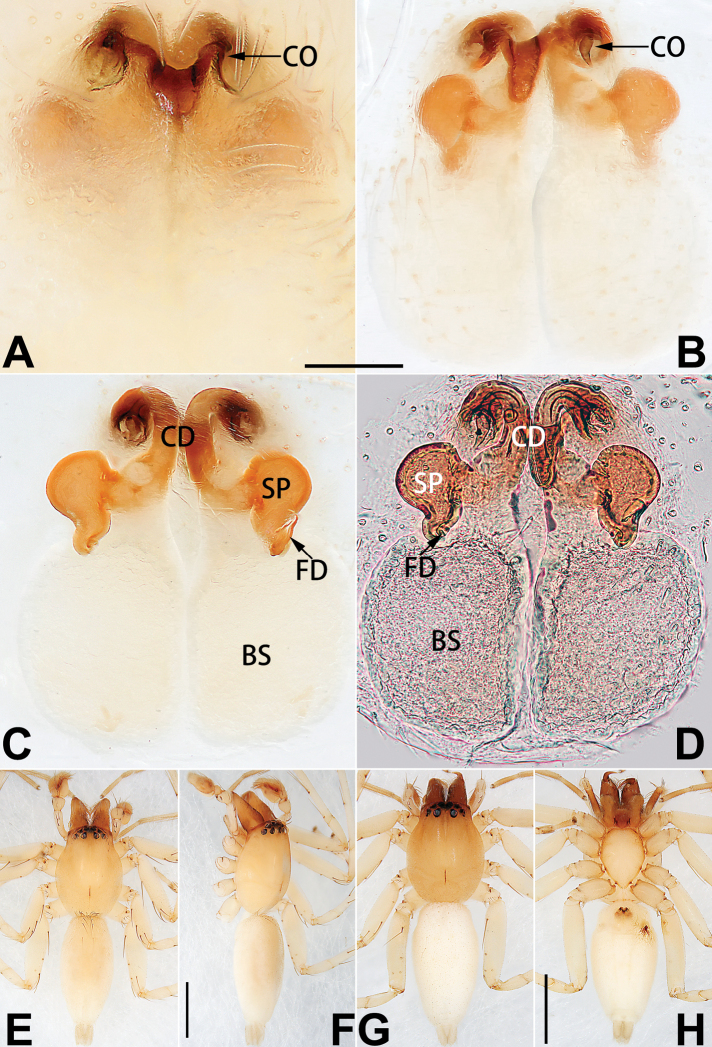
*Clubiona multidentata*, epigyne (**A–D**), male habitus (**E, F**) and female habitus (**G, H**) **A** intact, ventral view **B** cleared, ventral view **C** cleared, dorsal view **D** cleared, dorsal view **E** dorsal view **F** lateral view **G** dorsal view **H** ventral view. Abbreviations: BS = bursa; CD = copulatory duct; CO = copulatory opening; FD = fertilisation duct; SP = spermatheca. Scale bars: 0.1 mm (equal for **A–D**); 1 mm (equal for **E, F**, equal for **G, H**).

Figs 7 , 8 , 56B , 66B , 75D , 83D , 91D

Clubiona multidentata Liu et al., 2016: 569, figs 37–50 (♂♀).

##### Material examined.

1♂ (YHCLU0076), 1♀ (YHCLU0077), China: Yunnan Province: Xishuangbanna: Mengla County: Menglun Town: XTBG, 48^th^ km landmark in Menglun Nature Reserve, 21°58.704'N, 101°19.748'E, ca. 1088 m, 11.VIII.2011, G. Zheng et al. leg; 2♂5♀, XTBG, secondary tropical montane evergreen broad-leaved forest, 21°57.534'N, 101°12.300'E, ca. 860 m, 4.VIII.2007, G. Zheng leg.

##### Diagnosis and description.

See Liu et al. (2016). Male palp as in Figs 7, 56B, 66B, epigyne as in Figs 8A–D, 75D, 83D, 91D, habitus as in Fig. 8E–H.

##### Distribution.

China (Yunnan).

##### Most similar species.

*Clubiona submoralis*.

#### Clubiona parconcinna

Taxon classificationAnimaliaAraneaeClubionidae

Deeleman-Reinhold, 2001

89335C6E-05DD-51F0-B7A1-97C4D89EC1E9

**Figure 9.**
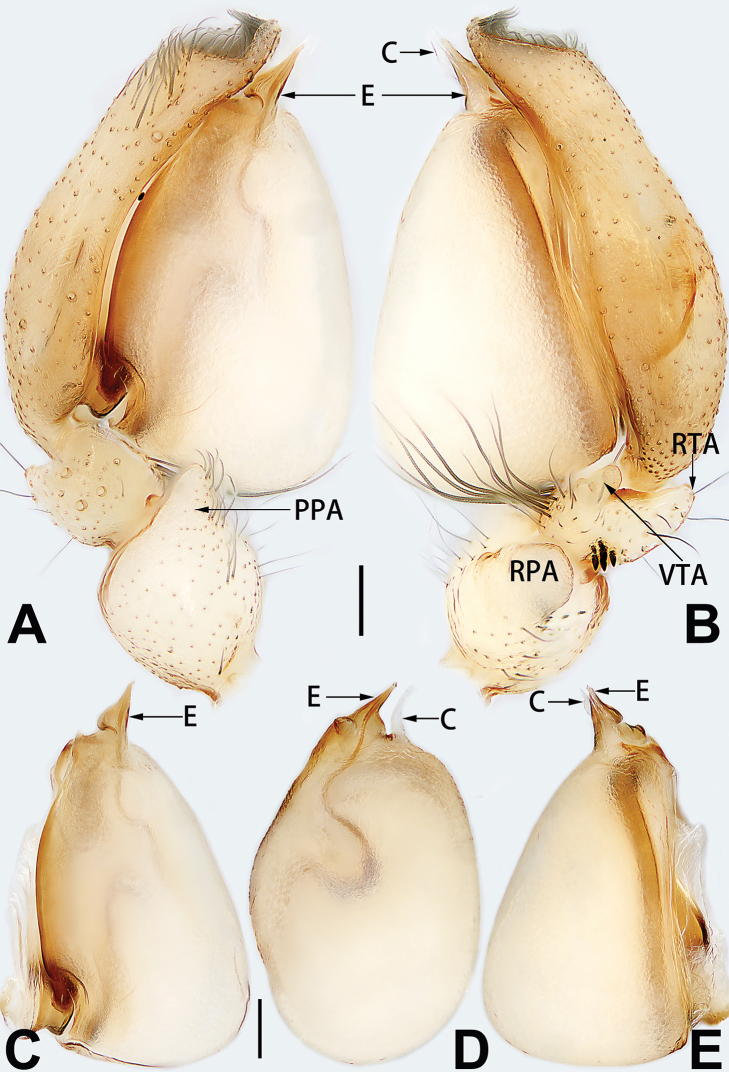
Male palp of *Clubiona parconcinna***A** prolateral view **B** retrolateral view **C** bulb, prolateral view **D** bulb, ventral view **E** bulb, retrolateral view. Abbreviations: C = conductor; E = embolus; PPA = prolateral patellar apophysis; RPA = retrolateral patellar apophysis; RTA = retrolateral tibial apophysis; VTA = ventral tibial apophysis. Scale bars: 0.1 mm (equal for **A, B**, equal for **C–E**).

**Figure 10.**
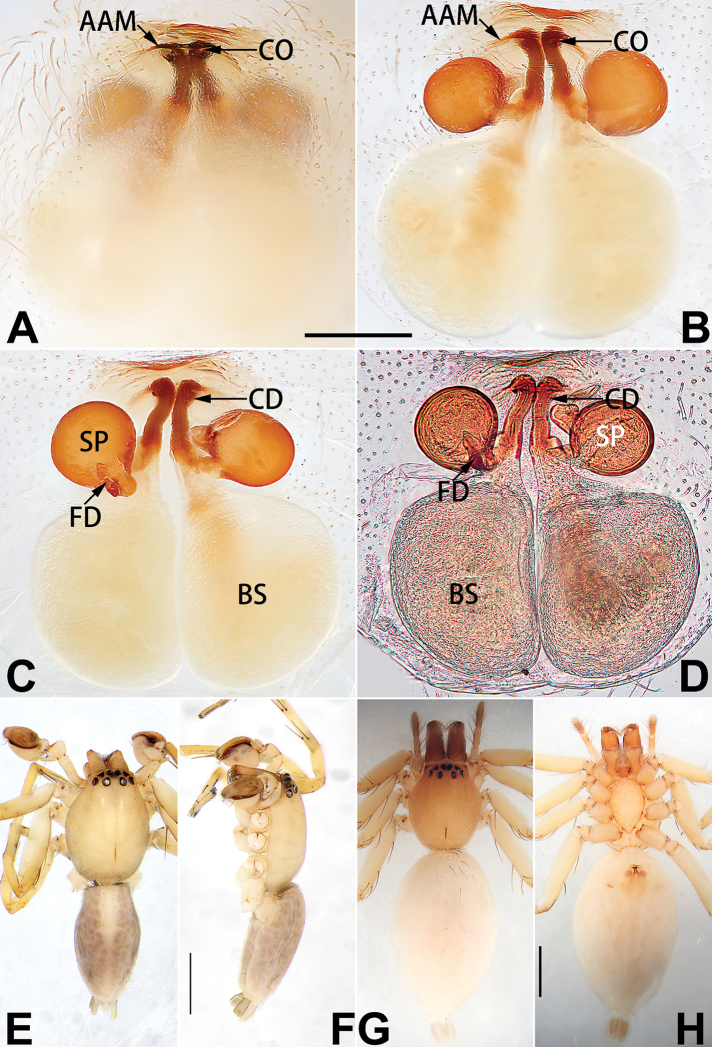
*Clubiona parconcinna*, epigyne (**A–D**), male habitus (**E, F**) and female habitus (**G, H**) **A** intact, ventral view **B** cleared, ventral view **C** cleared, dorsal view **D** cleared, dorsal view **E** dorsal view **F** lateral view **G** dorsal view **H** ventral view. Abbreviations: AAM = atrial anterior margin; BS = bursa; CD = copulatory duct; CO = copulatory opening; FD = fertilisation duct; SP = spermatheca. Scale bars: 0.2 mm (equal for **A–D**); 1 mm (equal for **E, F**, equal for **G, H**).

Figs 9 , 10 , 55C , 65C , 75C , 83C , 91C

Clubiona parconcinna Deeleman-Reinhold, 2001: 117, figs 34–40 (♂♀); Dankittipakul and Singtripop 2008b: 645, figs 1–9 (♂).

##### Material examined.

1♂ (YHCLU0143), China, Yunnan Province: Xishuangbanna: Mengla County: Bubang Village: monsoon forest, 21°36.827'N, 101°34.847'E, ca. 690 m, 12.VIII.2012, G. Zheng et al. leg; 1♀ (YHCLU0260), Mengla County: Manda Village: secondary forest, 22°01.421'N, 101°23.418'E, ca. 1188 m, 28.VII.2012, Q.Y. Zhao and Z.G. Chen leg; 6♂7♀, Menglun Town: XTBG, 55^th^ km landmark in the Menglun Nature Reserve, tropical ravine rainforest, 21°54.883'N, 101°12.147'E, ca. 829 m, 15.VIII.2011, Q.Y. Zhao and Z.G. Chen leg.

##### Diagnosis and description.

See Deeleman-Reinhold (2001) and Dankittipakul and Singtripop (2008b). Male palp as in Figs 9, 55C, 65C, epigyne as in Figs 10A–D, 75C, 83C, 91C, habitus as in Fig. 10E–H.

##### Distribution.

Thailand (Nakhon Ratchasima), Indonesia (Borneo), China (Yunnan).

##### Most similar species.

*Clubiona xiaoci* sp. nov.

#### Clubiona pollicaris

Taxon classificationAnimaliaAraneaeClubionidae

Wu, Zheng & Zhang, 2015

123B10DD-D121-5576-AD9B-7BD005CA2148

**Figure 11.**
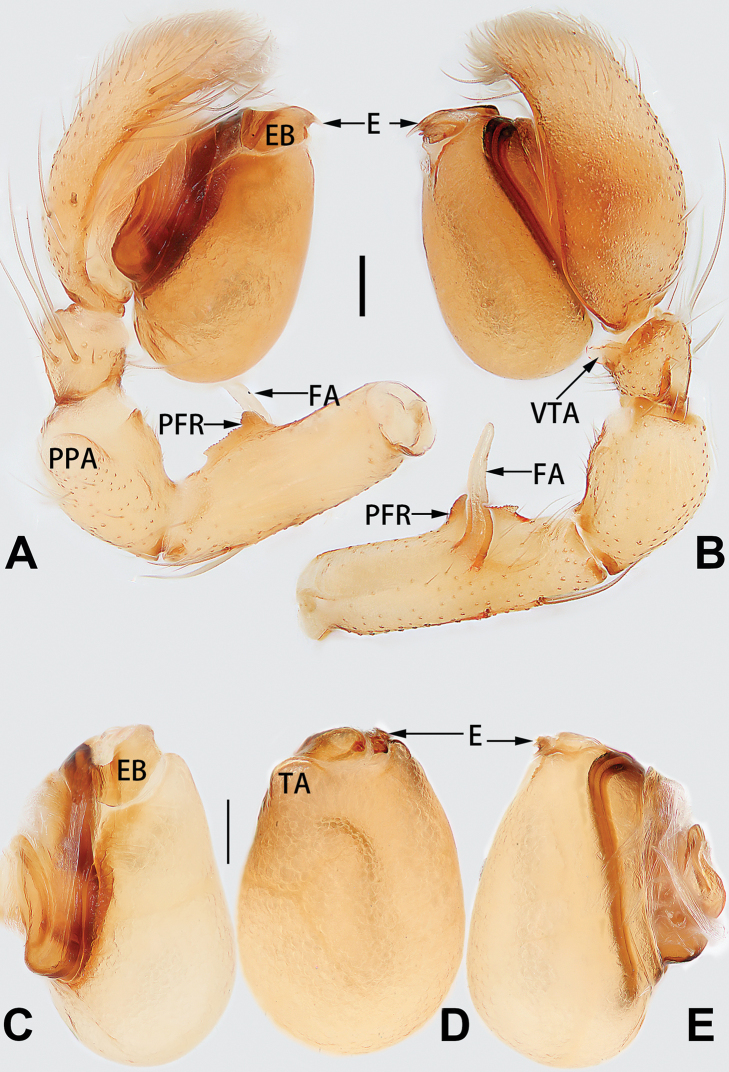
Male palp of *Clubiona pollicaris***A** prolateral view **B** retrolateral view **C** bulb, prolateral view **D** bulb, ventral view **E** bulb, retrolateral view. Abbreviations: E = embolus; EB = embolar base; FA = femoral apophysis; PFR = prolateral femoral ridge; PPA = prolateral patellar apophysis; TA = tegular apophysis; VTA = ventral tibial apophysis. Scale bars: 0.1 mm (equal for **A, B**, equal for **C–E**).

**Figure 12.**
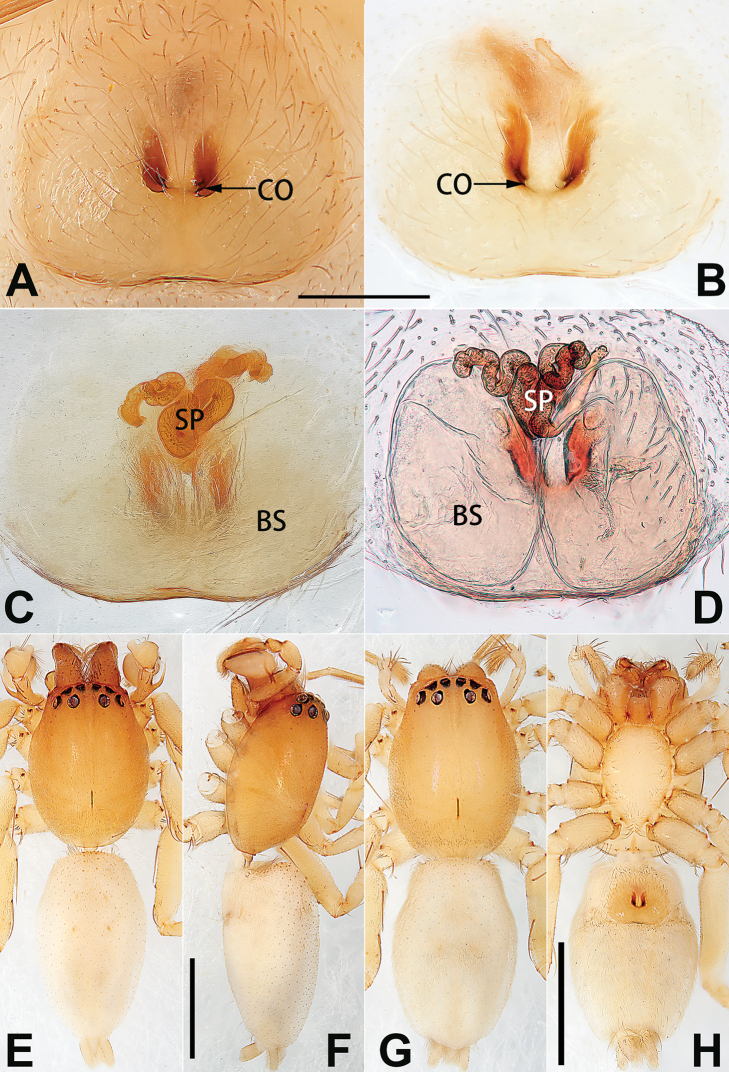
*Clubiona pollicaris*, epigyne (**A–D**), male habitus (**E, F**) and female habitus (**G, H**) **A** intact, ventral view **B** cleared, ventral view **C** cleared, dorsal view **D** cleared, dorsal view **E** dorsal view **F** lateral view **G** dorsal view **H** ventral view. Abbreviations: BS = bursa; CO = copulatory opening; SP = spermatheca. Scale bars: 0.2 mm (equal for **A–D**); 1 mm (equal for **E, F**, equal for **G, H**).

Figs 11 , 12 , 56C , 66C , 76B , 84B , 92B

Clubiona pollicaris Wu et al., 2015: 20, figs 13–19, 23–27 (♂♀).

##### Material examined.

1♂ (YHCLU0020), China: Yunnan Province: Xishuangbanna: Mengla County: Menglun Town: XTBG, Rainforest Nature Park, 21°55.017'N, 101°16.450'E, ca. 572 m, 16.VII.2018, H. Yu leg; 1♀ (YHCLU0021), XTBG, *Paramichelia baillonii* forest, 21°54.772'N, 101°16.043'E, ca. 556 m, 19.VII.2018, H. Yu leg; 16♂32♀, XTBG, low evergreen forest, 21°53.794'N, 101°17.152'E, ca. 594 m, 27.XI.2009, G. Tang and Z.Y. Yao leg.

##### Diagnosis and description.

See Wu et al. (2015). Male palp as in Figs 11, 56C, 66C, epigyne as in Figs 12A–D, 76B, 84B, 92B, habitus as in Fig. 12E–H.

##### Distribution.

Known only from Xishuangbanna.

##### Most similar species.

*Clubiona globosa*.

#### Clubiona rama

Taxon classificationAnimaliaAraneaeClubionidae

Dankittipakul & Singtripop, 2008

99CCB380-6207-5074-B15B-F741ACFE603F

**Figure 13.**
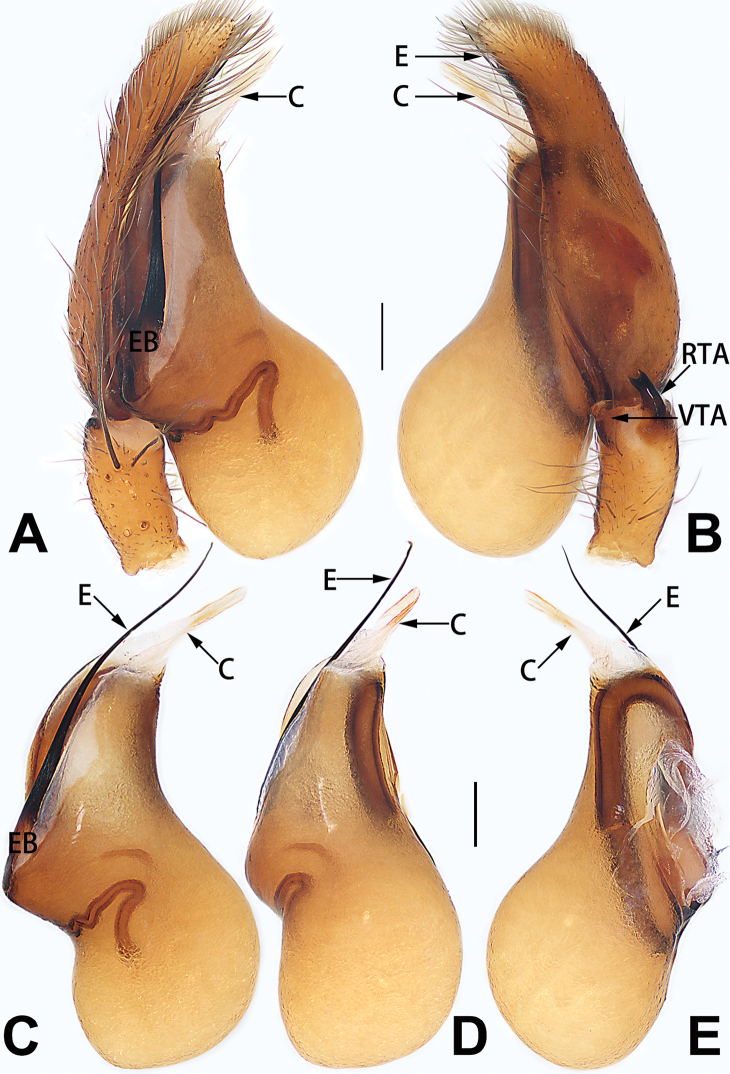
Male palp of *Clubiona rama***A** prolateral view **B** retrolateral view **C** bulb, prolateral view **D** bulb, ventral view **E** bulb, retrolateral view. Abbreviations: C = conductor; E = embolus; EB = embolar base; RTA = retrolateral tibial apophysis; VTA = ventral tibial apophysis. Scale bars: 0.2 mm (equal for **A, B**, equal for **C–E**).

**Figure 14.**
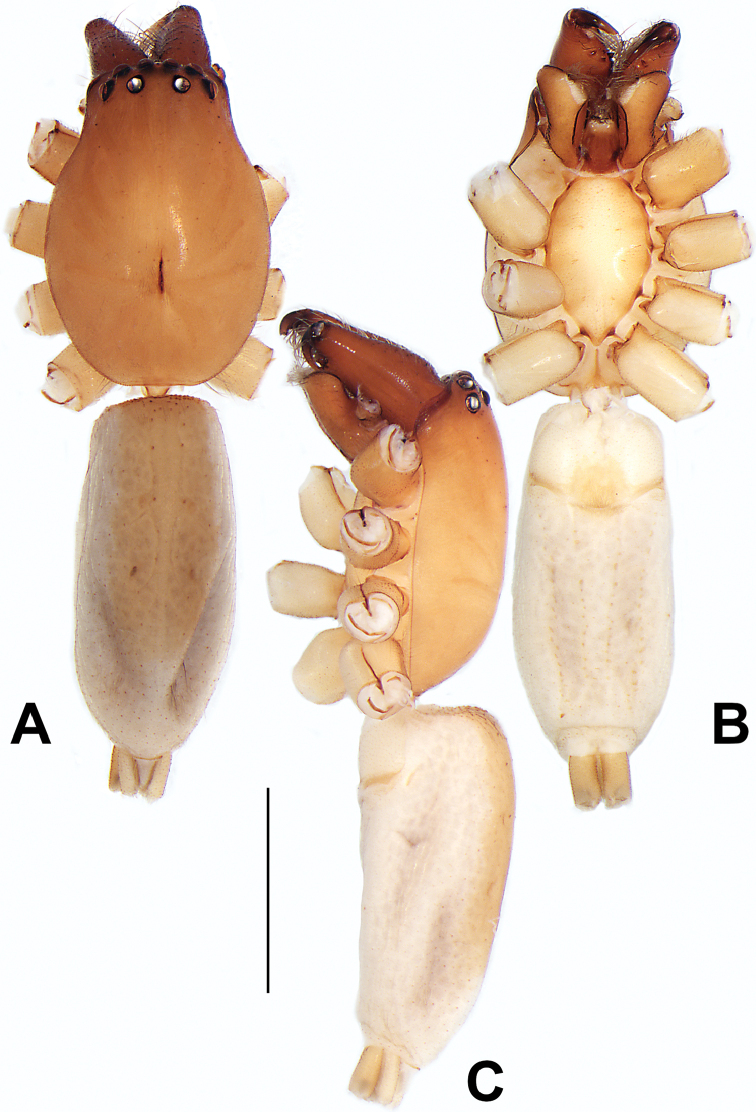
Male habitus of *Clubiona rama*. **A** dorsal view **B** ventral view **C** lateral view. Scale bar: 2 mm (equal for **A–C**).

Figs 13 , 14 , 53B , 63B

Clubiona rama Dankittipakul & Singtripop, 2008b: 645, figs 10–23 (♂♀); Dhali et al. 2016: 289, figs 7A–E, 8A–C (♀); Dhali et al. 2017: 58, figs 237–241, pl. 21 (♀); Yu et al. 2017b: 692, figs 6–10 (♀).

##### Material examined.

1♂, China: Yunnan Province: Xishuangbanna: Jinghong City: Nabanhe Natural Reserve, Mandian Waterfall, monsoon forest, 22°7.845'N, 100°39.749'E, ca. 736 m, 22.VIII.2012, G. Zheng leg.

##### Diagnosis and description.

See Dankittipakul and Singtripop (2008b). Male palp as in Figs 13, 53B, 63B, habitus as in Fig. 14.

##### Distribution.

India (West Bengal), Thailand (Phitsanulok), China (Yunnan).

##### Most similar species.

*Clubiona subrama*.

#### Clubiona subdidentata

Taxon classificationAnimaliaAraneaeClubionidae

Yu & Li sp. nov.

2FE4A8C4-00ED-5CA3-A665-AFE983608CB0

http://zoobank.org/B4B90700-E09E-430A-9B58-C80D6CF84084

**Figure 15.**
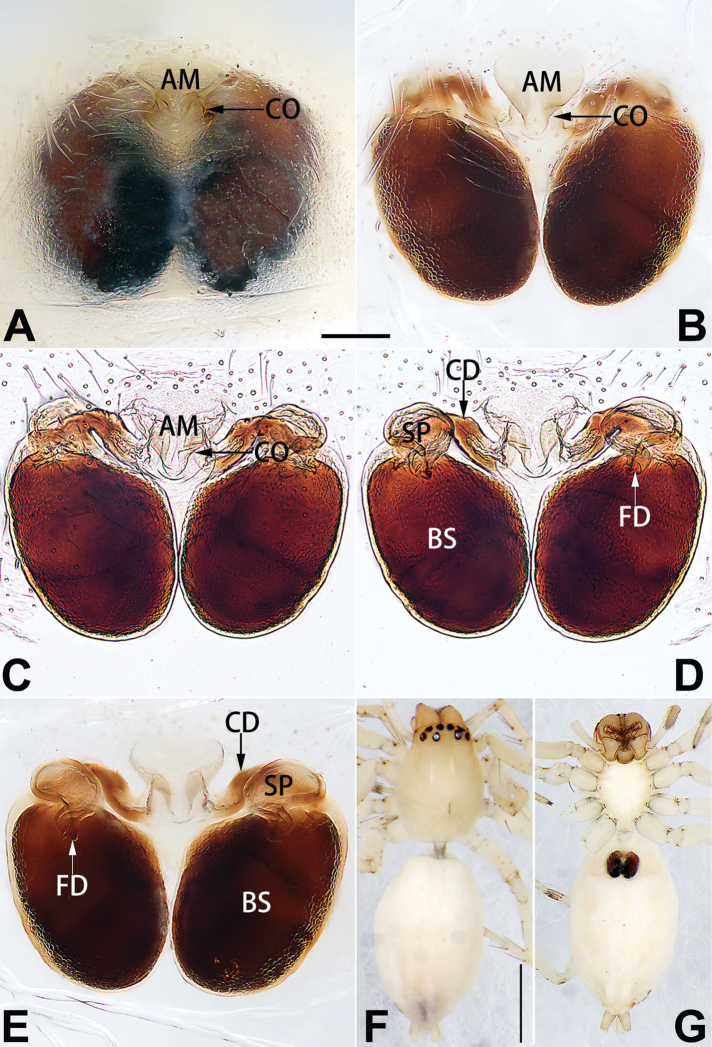
Holotype female of *Clubiona subdidentata* sp. nov., epigyne (**A–E**) and habitus (**F, G**) **A** intact, ventral view **B** cleared, ventral view **C** cleared, ventral view **D** cleared, dorsal view **E** cleared, dorsal view **F** dorsal view **G** ventral view. Abbreviations: AM = atrial membrane; BS = bursa; CD = copulatory duct; CO = copulatory opening; FD = fertilisation duct; SP = spermatheca. Scale bars: 0.1 mm (equal for **A–E**); 1 mm (equal for **F**, **G**).

Figs 15 , 74F , 82F , 90F

##### Holotype.

♀ (IZCAS-Ar 34749), China: Yunnan Province: Xishuangbanna: Mengla County: Xiaolongha Village: 22°5.017'N, 100°22.084'E, ca. 1118 m, 24.VII.2012, Q.Y. Zhao and Z.G. Chen leg. ***Other material examined.*** 1♀ (YHCLU0073), same data as holotype

##### Etymology.

The specific name is taken from its similarity to *C. didentata*; adjective.

##### Diagnosis.

The female of *C. subdidentata* sp. nov. can be distinguished from all other members of the *C. corticalis* group with the exception of *C. didentata* (Yu and Li 2019b: 207, figs 6A–D; Figs 74E, 82E, 90E) by having an atrial membrane (atrial membrane is absent in almost all species of the *corticalis* group) and similar vulva but can be recognised by the nearly equilateral triangular atrial membrane (Figs 15A–C, 74F, 82F) (vs. tongue- shaped in *C. didentata*; Figs 74E, 82E) and by the distinct copulatory ducts (Figs 15D, E, 90F) (copulatory ducts absent in *C. didentata*; Fig. 90E).

##### Description.

**Female.** Holotype (Fig. 15F, G): Total length 3.63; carapace 1.45 long, 1.14 wide; opisthosoma 2.18 long, 1.32 wide. Carapace uniformly greyish white, without any pattern or markings; ocular region distinctly narrowed, cervical groove and radial grooves indistinct; tegument smooth, all setae detached in ethanol. Eyes: AER slightly recurved, PER wider than AER and almost straight in dorsal view. Eye sizes and interdistances: AME 0.09, ALE 0.09, PME 0.09, PLE 0.07, AME–AME 0.06, AME–ALE 0.07, PME–PME 0.19, PME–PLE 0.10, MOQL 0.21, MOQA 0.23, MOQP 0.38. Chelicerae light orange, with three promarginal and two retromarginal teeth. Sternum pale brown, 0.80 long, 0.65 wide. Labium and endites coloured as chelicerae. Legs greyish white, uniformly coloured. Leg measurements: I 2.73 (0.81, 1.15, 0.55, 0.23), II 3.15 (0.93, 1.25, 0.74, 0.22), III 2.85 (0.93, 0.95, 0.70, 0.29), IV 3.39 (1.19, 1.32, 1.05, 0.39). Abdomen oval, nearly pure white, with inconspicuous anterior setal tufts, dorsum with two pairs of inconspicuous muscle depressions; venter without pattern.

Epigyne (Figs 15A–E, 74F, 82F, 90F). Epigynal plate ca. 1.5 × wider than long, margin not delimited; spermathecae and bursae prominently visible through epigynal plate. Atrium small, anteriorly covered by an atrial membrane. Atrial membrane shaped nearly like an equilateral triangle, with a blunt apex. Copulatory openings small, located at basolateral atrial borders. Copulatory ducts distinct, extend transversally, connecting to posteriorly located bursae. Spermathecae small, consisting of a bean-shaped proximal part and an acicular distal part, with short fertilisation ducts terminally. Bursae reniform, close together, ca. 1.3 × longer than wide, bursal surface hyaline and smooth, inside pigmented and sclerotised.

**Male.** Unknown.

##### Distribution.

Known only from the type locality, Xishuangbanna, Yunnan, China.

#### Clubiona submoralis

Taxon classificationAnimaliaAraneaeClubionidae

Wu, Zheng & Zhang, 2015

00A116D3-C155-5682-AD1D-A255A514AA3F

**Figure 16.**
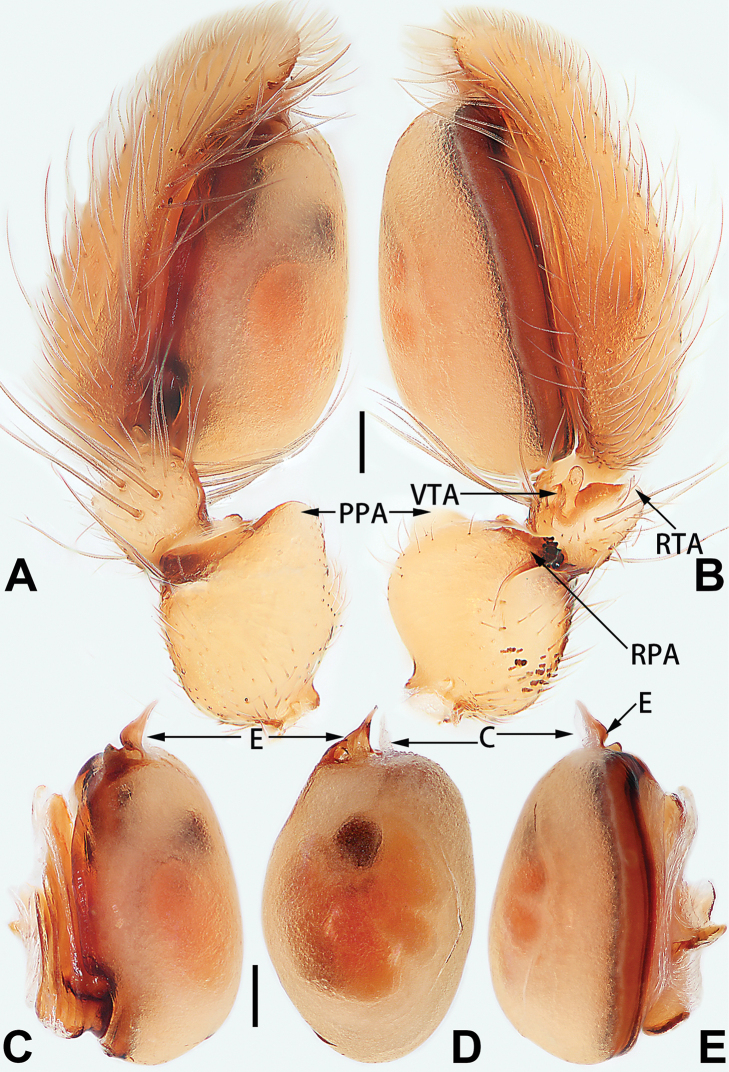
Male palp of *Clubiona submoralis***A** prolateral view **B** retrolateral view **C** bulb, prolateral view **D** bulb, ventral view **E** bulb, retrolateral view. Abbreviations: C = conductor; E = embolus; PPA = prolateral patellar apophysis; RPA = retrolateral patellar apophysis; RTA = retrolateral tibial apophysis; VTA = ventral tibial apophysis. Scale bars: 0.1 mm (equal for **A, B**, equal for **C–E**).

**Figure 17.**
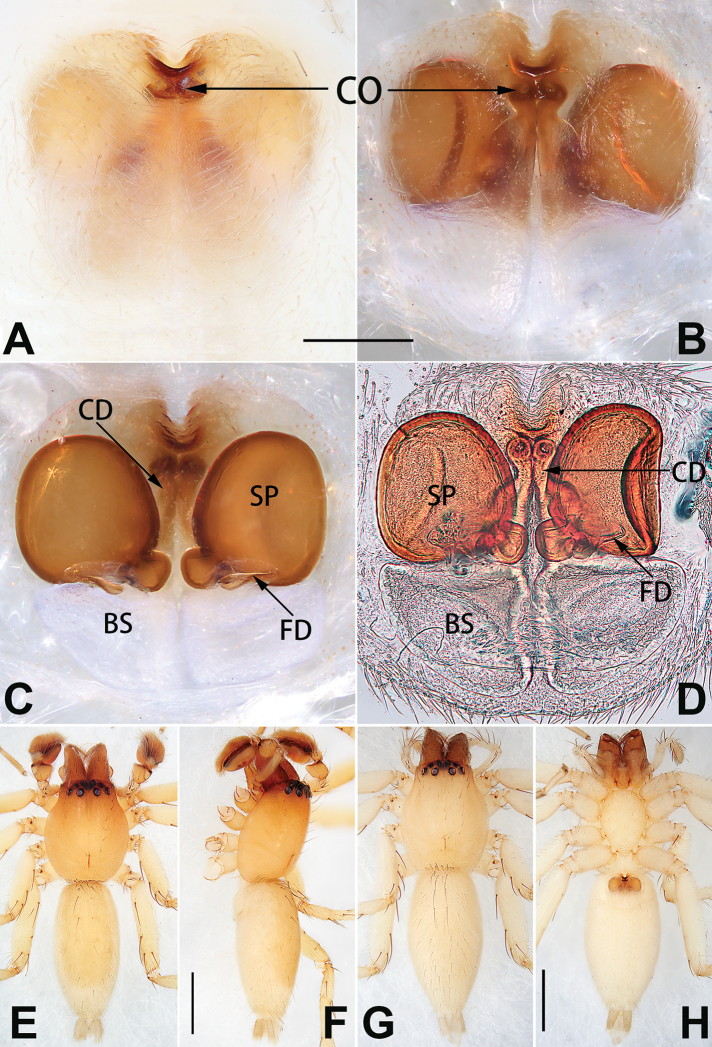
*Clubiona submoralis*, epigyne (**A–D**), male habitus (**E, F**) and female habitus (**G, H**) **A** intact, ventral view **B** cleared, ventral view **C** cleared, dorsal view **D** cleared, dorsal view **E** dorsal view **F** lateral view **G** dorsal view **H** ventral view. Abbreviations: BS = bursa; CD = copulatory duct; CO = copulatory opening; FD = fertilisation duct; SP = spermatheca. Scale bars: 0.2 mm (equal for **A–D**); 1 mm (equal for **E, F**, equal for **G, H**).

Figs 16 , 17 , 55B , 65B , 75B , 83B , 91B

Clubiona submoralis Wu et al., 2015: 17, figs 1–12 (♂♀).

##### Material examined.

1♂ (YHCLU0028), China: Yunnan Province: Xishuangbanna: Mengla County: Menglun Town: XTBG, *Paramichelia baillonii* Forest, 21°54.772'N, 101°16.043'E, ca. 556 m, 19.VII.2018, H. Yu leg; 1♀ (YHCLU0029), XTBG, rubber plantation, 21°54.674'N, 101°16.207'E, ca. 583 m, 21.VII.2018, H. Yu leg.; 6♂8♀, XTBG, secondary forest, 21°54.459'N, 101°16.755'E, ca. 644 m, 20.XI.2009, G. Tang and Z.Y. Yao leg.

##### Diagnosis and description.

See Wu et al. (2015). Male palp as in Figs 16, 55B, 65B, epigyne as in Figs 17A–D, 75B, 83B, 91B, habitus as in Fig. 17E–H.

##### Distribution.

Known only from Xishuangbanna.

##### Most similar species.

*Clubiona moralis*.

#### Clubiona subrama

Taxon classificationAnimaliaAraneaeClubionidae

Yu & Li, 2019

ADC9EC07-E2D8-5ECF-A1EA-FB4A45B34127

Figs 53C , 63C , 73E , 81E , 89E

Clubiona subrama Yu & Li, 2019a: 153, figs 3A–E, 4A–G (♂♀).

##### Material examined.

***Types*.** Holotype ♂ (IZCAS Ar 34524), China: Yunnan Province: Xishuangbanna: Mengla County: Menglun Town: XTBG, Lvshilin Forest Park, limestone tropical seasonal rainforest, 21°54.705'N, 101°16.898'E, ca. 656 m, 13.XI.2009, G. Tang and Z.Y. Yao leg. ***Other material examined.*** 1♀, XTBG, rubber plantation, 21°54.498'N, 101°16.326'E, ca. 586 m, 29.II.2009, G. Zheng leg; 1♂ (YHCLU0083) and 1♀ (YHCLU0084), XTBG, Lvshilin Forest Park, evergreen forest, 21°54.555'N, 101°16.860'E, ca. 616 m, 29.XI.2009, G. Tang and Z.Y. Yao leg.

##### Diagnosis and description.

See Yu and Li (2019a). Male palp as in Figs 53C, 63C, epigyne as in Figs 73E, 81E, 89E.

##### Distribution.

Known only from Xishuangbanna.

##### Most similar species.

*Clubiona rama*.

#### Clubiona subyaginumai

Taxon classificationAnimaliaAraneaeClubionidae

Yu & Li, 2019

7A96D7F7-2BF8-5826-B524-8B375F5E1B26

Figs 54C , 64C , 75F , 83F , 91F

Clubiona subyaginumai Yu & Li, 2019a: 158, figs. 5A–E, 6A–G (♂♀).

##### Material examined.

***Types*.** Holotype ♂ (IZCAS Ar 34548), China: Yunnan Province: Xishuangbanna: Mengla County: Menglun Town: XTBG, secondary tropical forest, 21°54.380'N, 101°16.815'E, ca. 627 m, 23.XI.2009, G. Tang and Z.Y. Yao leg. ***Other material examined.*** 1♀, XTBG, bamboo plantation, 21°54.380'N, 101°16.815'E, ca. 620 m, 21.XI.2009, G. Tang and Z.Y. Yao leg.

##### Diagnosis and description.

See Yu and Li (2019a). Male palp as in Figs 54C, 64C, epigyne as in Figs 75F, 83F, 91F.

##### Distribution.

Known only from Xishuangbanna.

##### Most similar species.

*Clubiona yaginumai*.

#### Clubiona tixing

Taxon classificationAnimaliaAraneaeClubionidae

Yu & Li sp. nov.

31DC512F-7298-5C24-B97D-462BECB43C52

http://zoobank.org/0E0C7ACD-01A4-4D3F-833A-0F84AE4285AB

**Figure 18.**
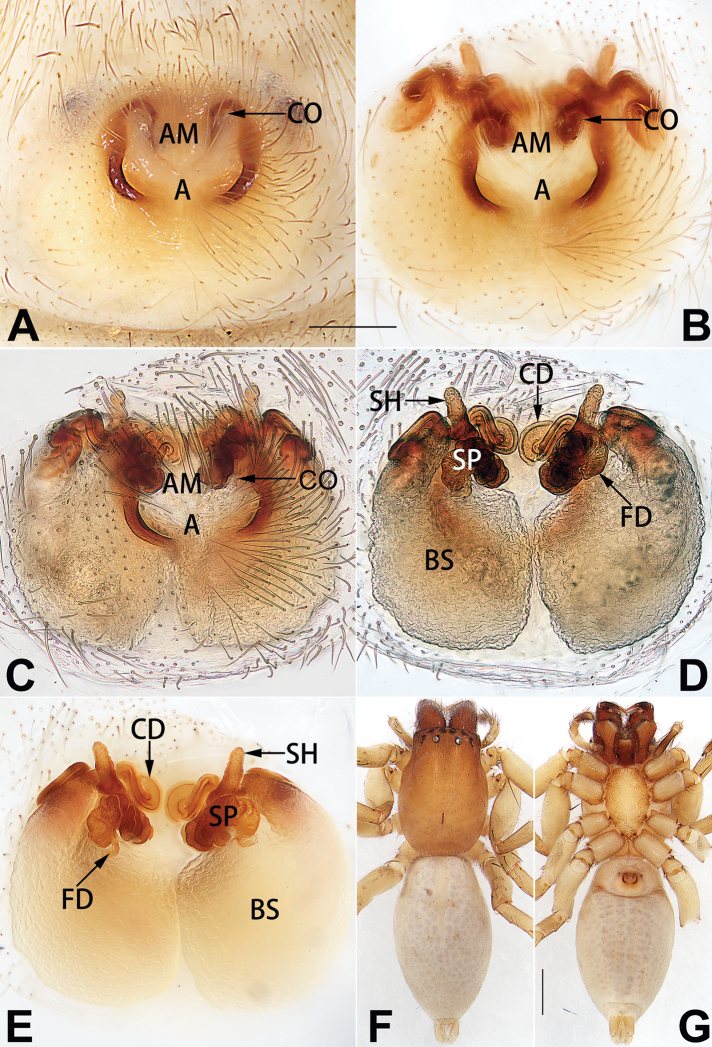
Holotype female of *Clubiona tixing* sp. nov., epigyne (**A–E**) and habitus (**F, G**) **A** intact, ventral view **B** cleared, ventral view **C** cleared, ventral view **D** cleared, dorsal view **E** cleared, dorsal view **F** dorsal view **G** ventral view. Abbreviations: A = atrium; AM = atrial membrane; BS = bursa; CD = copulatory duct; CO = copulatory opening; FD = fertilisation duct; SH = spermathecal head; SP = spermatheca. Scale bars: 0.2 mm (equal for **A–E**); 1 mm (equal for **F**, **G**).

Figs 18 , 73D , 81D , 89D

##### Holotype

♀ (IZCAS-Ar 34750), China: Yunnan Province: Xishuangbanna: Jinghong City: Nabanhe Natural Reserve, Mandian Waterfall, monsoon forest, 22°7.845'N, 100°39.749'E, ca. 736 m, 22.VIII.2012, G. Zheng leg.

##### Etymology.

The specific name is derived from the Chinese pinyin *tī xíng*, which means ‘trapezoid’, referring to the trapezoidal atrium; noun in apposition.

##### Diagnosis.

Females of this species resemble those of *C. zhangmuensis* (Zhang et al. 2007: 38, figs 7–8) in having a similar atrium and endogyne but can be recognised by the presence of an atrial membrane (Figs 18A–C, 73D, 81D) (vs. absent; Zhang et al. 2007: 38, fig. 7).

##### Description.

**Female.** Holotype (Fig. 18F, G): Total length 6.62; carapace 2.80 long, 2.02 wide; opisthosoma 3.82 long, 2.28 wide. Carapace brown in alcohol, uniformly coloured, without distinct pattern, ocular region distinctly narrowed, cervical groove and radial grooves indistinct; tegument smooth, clothed with short setae. Eyes: in dorsal view, both AER and PER almost straight, the former narrower than the latter. Eye sizes and interdistances: AME 0.14, ALE 0.15, PME 0.14, PLE 0.12, AME–AME 0.12, AME–ALE 0.13, PME–PME 0.39, PME–PLE 0.24, MOQL 0.43, MOQA 0.41, MOQP 0.67. Chelicerae robust and dark brown, with five promarginal and three retromarginal teeth. Sternum yellowish brown, darker marginally, 1.63 long, 1.00 wide. Labium and endites coloured as carapace. Legs uniformly yellowish white. Leg measurements: I – (1.71, 2.25, –, –), II – (1.78, 2.42, –, –), III – (1.60, 1.88, –, –), IV – (2.11, 2.55, 1.17, –). Abdomen elongate-oval, uniformly pale brown, with numerous brown spots; dorsally with a lengthwise white heart mark, reaching posterior half, with two pairs of muscle depressions located at lateral part of heart mark; venter medially with four longitudinal dotted lines.

Epigyne (Figs 18A–E, 73D, 81D, 89D). Epigynal plate distinctly wider than long, margin not rebordered, spermathecae and bursae are indistinctly visible through epigynal plate in ventral view. Atrium large, more or less rectangular or trapezoidal, ca. 1.3 × wider than long, ca. 1/3 of epigyne length, with tongue-shaped membrane on anterior margin, posterior atrial border not delimited. Copulatory openings indistinct, located at anterolateral atrial borders. Copulatory ducts short, bent dorsally and ascend obliquely to connect with bursae. Spermathecae convoluted, anteriorly situated, spermathecal heads thick. Bursae reniform, close together, ca. 1.6 × longer than wide, surface translucent and wrinkled.

**Male.** Unknown.

##### Distribution.

Known only from the type locality, Xishuangbanna, Yunnan, China.

#### Clubiona tiane

Taxon classificationAnimaliaAraneaeClubionidae

Yu & Li, 2019

F51979B6-C35D-520B-8AE5-94513D150F01

**Figure 19.**
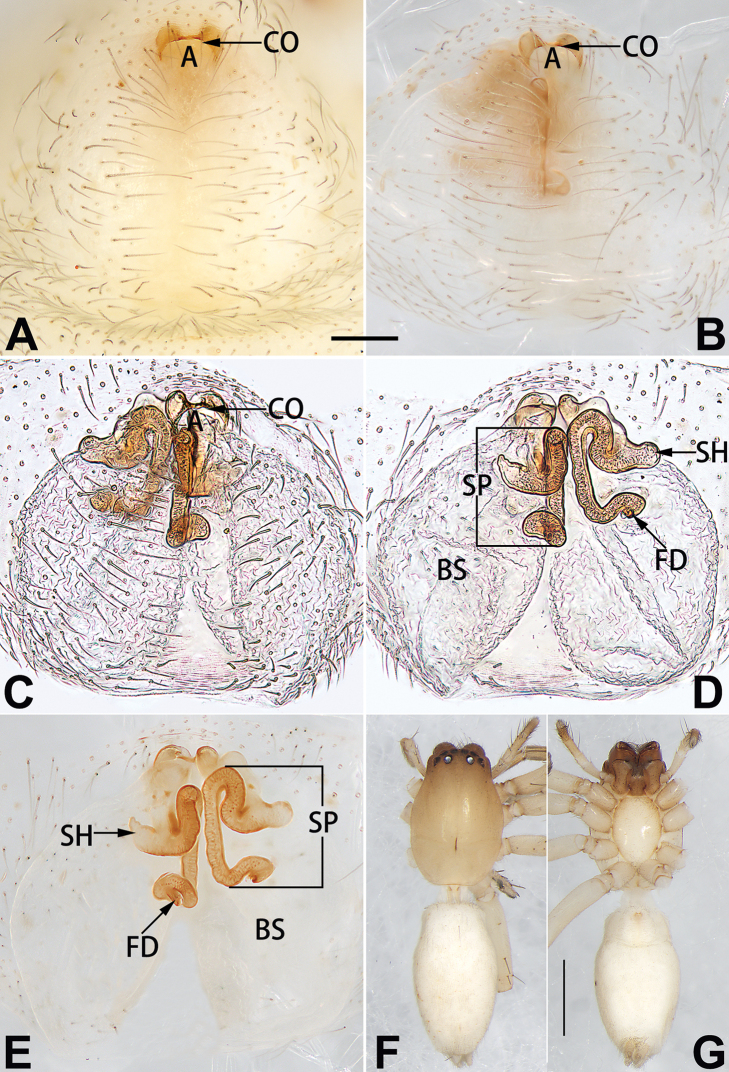
*Clubiona tiane*, epigyne (**A–E**) and female habitus (**F, G**) **A** intact, ventral view **B** cleared, ventral view **C** cleared, ventral view **D** cleared, dorsal view **E** cleared, dorsal view **F** dorsal view **G** ventral view. Abbreviations: A = atrium; BS = bursa; CO = copulatory opening; FD = fertilisation duct; SH = spermathecal head; SP = spermatheca. Scale bars: 0.1 mm (equal for **A–E**); 1 mm (equal for **F**, **G**).

Figs 19 , 54D , 64D , 74D , 82D , 90D

Clubiona tiane Yu & Li, 2019b: 204, figs 3A–E, 4A–C (♂).

##### Material examined.

***Types*.** Holotype ♂ (IZCAS Ar 34703), China: Yunnan Province: Xishuangbanna: Mengla County: Menglun Town: XTBG, bamboo plantation, 21°53.642'N, 101°16.940'E, ca. 589 m, 22.VII.2018, H. Yu and Z.G. Chen leg. ***Other material examined.*** 1♂ (YHCLU0054), XTBG, rubber-tea plantation, 21°55.233'N, 101°15.850'E, ca. 572 m, 16.VII.2018, Hao Yu et al. leg; 1♀ (YHCLU0053), XTBG, Rainforest Nature Park, 21°55.017'N, 101°16.450'E, ca. 572 m, 16.VII.2018, H. Yu et al. leg.

##### Diagnosis.

Females of *C. tiane* resemble those of *C. dactylina* (Liu et al. 2016: figs 18, 19, 23, 24) in having a semi-circular atrium and tubular spermathecae but differ in the following: (1) indistinct copulatory openings hidden at anterior sides of atrial border (Figs 19A–C, 74D, 82D) (vs. distinct copulatory openings located posteriorly in atrium; Liu et al. 2016: figs 18, 23); (2) more or less lengthwise, thick spermathecae (Figs 19D, E, 90D) (vs. nearly horizontal and relatively thin spermathecae); (3) distinct and thick spermathecal heads (Figs 19D, E, 90D) (vs. indistinct and relatively small spermathecal heads; Liu et al. 2016: figs 19, 24). Males of *C. tiane* can be easily recognised by the curved embolus shaped like a swan’s neck (Figs 54D, 64D).

##### Description.

**Male.** See Yu and Li (2019b). Male palp as in Figs 54D, 64D.

**Female** (Fig. 19F, G). Total length 3.90; carapace 1.77 long, 1.06 wide; opisthosoma 2.14 long, 0.93 wide. Carapace light grey except brownish ocular area, without pattern; ocular region slightly narrowed, cervical groove and radial grooves indistinguishable; tegument smooth, all setae detached in ethanol. Eyes: both AER and PER slightly recurved in dorsal view, PER slightly wider than AER. Eye sizes and interdistances: AME 0.09, ALE 0.09, PME 0.10, PLE 0.08, AME–AME 0.09, AME–ALE 0.06, PME–PME 0.17, PME–PLE 0.12, MOQL 0.23, MOQA 0.31, MOQP 0.32. Chelicerae coloured as ocular area, with six promarginal and four retromarginal teeth. Sternum yellowish white, 0.94 long, 0.61 wide. Labium and endites brownish. Legs yellowish white, without distinct markings. Leg measurements: I 2.38 (0.68, 1.12, 0.28, 0.30), II 2.87 (0.87, 1.25, 0.35, 0.39), III 2.30 (0.63, 0.63, 0.79, 0.25), IV 3.78 (1.39, 1.19, 0.88, 0.32). Abdomen elongate, oval, nearly pure white, with inconspicuous anterior setal tufts, dorsum clothed with dense setae; venter uniformly white, without pattern.

Epigyne (Figs 19A–E, 74D, 82D, 90D). Epigynal plate slightly wider than long, margin not rebordered, spermathecae and bursae obscured through epigynal plate in ventral view. Atrium small, semi-circular, ca. 1/4 of epigyne width, anterior atrial border heavily sclerotised. Copulatory openings indistinct, hidden by anterior margin of atrium. Copulatory ducts indistinct, directed posteriorly to connect with spermathecae. Spermathecae longitudinal, with short, acicular fertilisation ducts. Spermathecal heads digitiform and horizontal, arising on the proximal part of the spermathecae. Bursae ovoid, large, close together, ca. 1.7 × longer than wide, surface translucent, with a wrinkled and ribbed appearance.

##### Distribution.

Known only from Xishuangbanna.

##### Remarks.

The female of the species is described for the first time.

#### Clubiona xiaoci

Taxon classificationAnimaliaAraneaeClubionidae

Yu & Li sp. nov.

BB1A2289-7B22-5ADB-BEC1-3485D33279D5

http://zoobank.org/1044ED46-1096-46CF-A856-CF991E21B404

**Figure 20.**
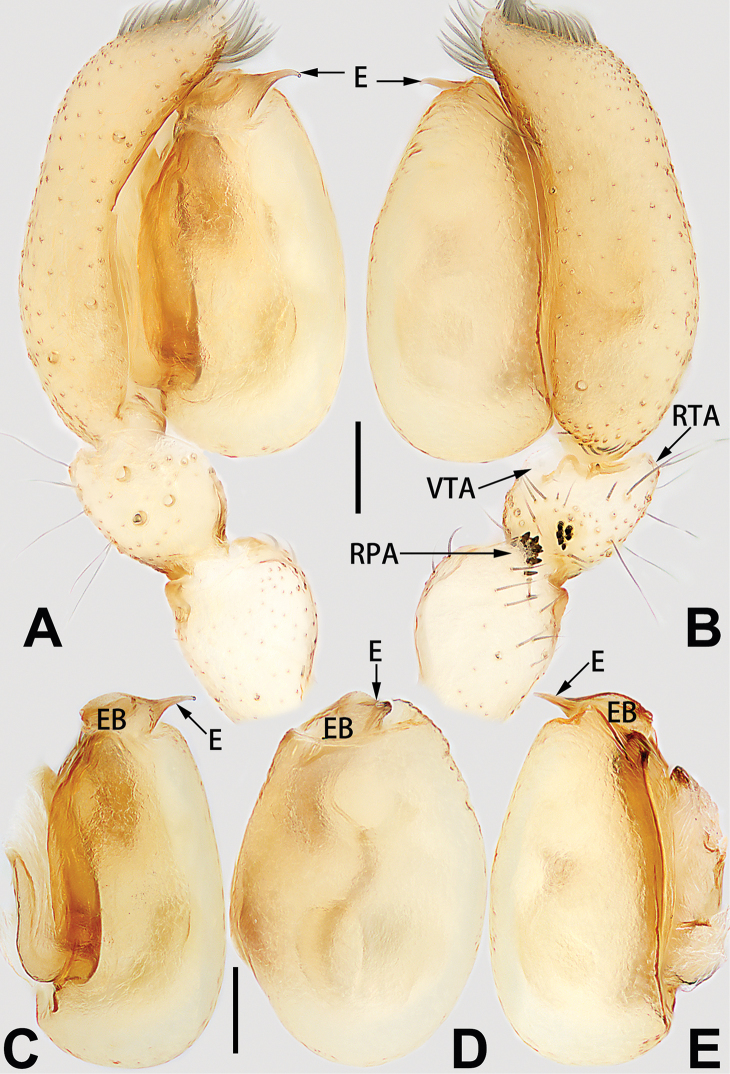
Male palp of the holotype of *Clubiona xiaoci* sp. nov. **A** prolateral view **B** retrolateral view **C** bulb, prolateral view **D** bulb, ventral view **E** bulb, retrolateral view. Abbreviations: E = embolus; EB = embolar base; RPA = retrolateral patellar apophysis; RTA = retrolateral tibial apophysis; VTA = ventral tibial apophysis. Scale bars: 0.1 mm (equal for **A, B**, equal for **C–E**).

**Figure 21.**
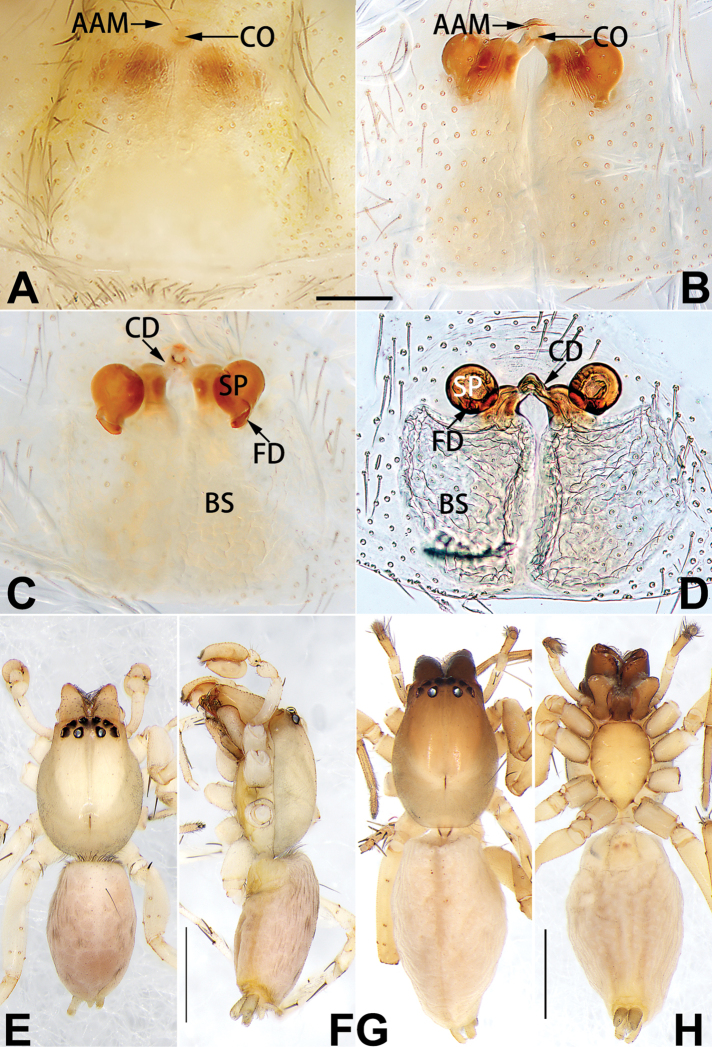
*Clubiona xiaoci* sp. nov., female paratype and male holotype, epigyne (**A–D**), male habitus (**E, F**) and female habitus (**G, H**) **A** intact, ventral view **B** cleared, ventral view **C** cleared, dorsal view **D** cleared, dorsal view **E** dorsal view **F** lateral view **G** dorsal view **H** ventral view. Abbreviations: AAM = atrial anterior margin; BS = bursa; CD = copulatory duct; CO = copulatory opening; FD = fertilisation duct; SP = spermatheca. Scale bars: 0.1 mm (equal for **A–D**); 1 mm (equal for **E, F**, equal for **G, H**).

Figs 20 , 21 , 55D , 65D , 75E , 83E , 91E

##### Holotype

♂ (IZCAS-Ar 34751), China: Yunnan Province: Xishuangbanna: Jinghong City: Menga Town: Wengnan Village: secondary forest, 22°5.020'N, 100°22.087'E, ca. 1118 m, 24.VII.2012, Q.Y. Zhao and Z.G. Chen leg. ***Paratype***: 1♀ (IZCAS-Ar 34752), same data as holotype. ***Other material examined.*** 1 ♂ (YHCLU0088) and 1 ♀ (YHCLU0089), same locality and same collectors as holotype, 22°4.997'N, 100°22.223'E, ca. 1137 m, 25.VII.2012.

##### Etymology.

The specific name is derived from the Chinese pinyin *xiǎo cì*, which means ‘small spines’, referring to the short spines located on the palpal tibia and patella; noun in apposition.

##### Diagnosis.

*Clubiona xiaoci* sp. nov. is very similar to *C. parconcinna* (see Figs 9, 10, 55C, 65C, 75C, 83C, 91C and Deeleman-Reinhold 2001: 117, figs 34–40). Males are similar by the palpal tibia with several short spines. Females of *C. xiaoci* sp. nov. resemble those of *C. parconcinna* in having similar atrial anterior margins and globular spermathecae. *C. xiaoci* sp. nov. can be distinguished from *C. parconcinna* by the following characters: for the males, conductor absent in *C. xiaoci* sp. nov. (Figs 20, 55D, 65D) (vs. distinct; Figs 9B, D, E, 55C, 65C), retrolateral patellar apophysis with short, modified spines in new species (Figs 20B, 55D, 65D) (vs. without spines; Figs 9B, 55C, 65C); for the females, copulatory openings are partly fused in *C. xiaoci* sp. nov. (Figs 21A, B, 75E, 83E) (vs. copulatory openings separated; Figs 10A, B, 75C, 83C), copulatory ducts distinctly shorter and thicker in *C. xiaoci* sp. nov. (cf. Figs 21C, D, 91E and Figs 10C, D, 91C).

##### Description.

**Male.** Holotype (Fig. 21E, F): Total length 3.17; carapace 1.51 long, 1.10 wide; opisthosoma 1.66 long, 0.94 wide. Carapace greyish white, slightly lighter in cephalic area, with a pair of short faint lines running longitudinally from behind AME, ocular region distinctly narrowed; cervical groove indistinct; tegument smooth, clothed with short setae. Eyes: AER slightly recurved, PER almost straight, the latter wider than the former. Eye sizes and interdistances: AME 0.07, ALE 0.08, PME 0.07, PLE 0.06, AME–AME 0.07, AME–ALE 0.05, PME–PME 0.10, PME–PLE 0.07, MOQL 0.19, MOQA 0.23, MOQP 0.31. Chelicerae robust and light orange, with three promarginal and two retromarginal teeth. Sternum pale yellow, 0.83 long, 0.63 wide. Labium and endites coloured as chelicerae. Legs yellowish white, without markings. Leg measurements: I – (1.06, 1.41, 0.75, –), II 4.22 (1.19, 1.76, 0.83, 0.44), III 3.02 (0.93, 1.12, 0.75, 0.22), 4.44 (1.27, 1.43, 1.27, 0.46). Abdomen oval, dorsally light pink with conspicuous anterior setal tufts; venter pale yellow, without pattern.

Palp (Figs 20A–E, 55D, 65D). Femur unmodified. Patella with single retrolateral apophysis, apophysis with short, modified spines at apex. Tibia short, cup-shaped, retrolaterally with several short spines near base, with two apophyses: a papilliform, partly membranous ventro-retrolateral apophysis and a dorsal one, with a blunt apex, trapezoidal. Cymbium 2.3 × longer than wide. Tegulum elongate, oval, and bulging, 1.5 × longer than wide; sperm duct indistinct in ventral view. Embolus needle-like, distinctly short, originating at distal portion of tegulum, gradually tapering toward tip, apex sharp and prolaterally pointed; embolar base represented by enlarged tubercle.

**Female.** Paratype (Fig. 21G, H): total length 3.93; carapace 1.69 long, 1.21 wide; opisthosoma 2.24 long, 1.30 wide. Eye sizes and interdistances: AME 0.09, ALE 0.10, PME 0.10, PLE 0.09, AME–AME 0.05, AME–ALE 0.05, PME–PME 0.19, PME–PLE 0.09, MOQL 0.28, MOQA 0.23, MOQP 0.38. Sternum 0.99 long, 0.67 wide. Leg measurements: I 3.56 (1.11, 1.42, 0.63, 0.41), II 3.80 (1.15, 1.52, 0.67, 0.44), III – (0.90, –, 0.78, 0.34), IV 4.42 (1.32, 1.48, 1.15, 0.47). Distinctly larger and darker than male, other characters as in male.

Epigyne (Figs 21A–D, 75E, 83E, 91E). Epigynal plate nearly as wide as long, spermathecae distinctly visible through integument. Atrium indistinct, anterior margin rebordered. Copulatory openings small and partly fused, situated anteriorly on atrium. Copulatory ducts short, ascending obliquely, connecting to posteriorly located bursae then ascending to anteriorly located spermathecae. Spermathecae nearly globular, separated by 1.5 diameters. Fertilisation ducts small, acicular, and membranous, located on posterior surface of spermathecae. Bursae reniform, large, close together, ca. 1.25 × longer than wide, surface translucent and wrinkled.

##### Distribution.

Known only from the type locality, Xishuangbanna, Yunnan, China.

#### Clubiona xiaokong

Taxon classificationAnimaliaAraneaeClubionidae

Yu & Li sp. nov.

AA593285-1B9F-5A28-9E05-1CBB1B8D4490

http://zoobank.org/D63C0094-D38F-4FFC-9937-D0A336324E28

**Figure 22.**
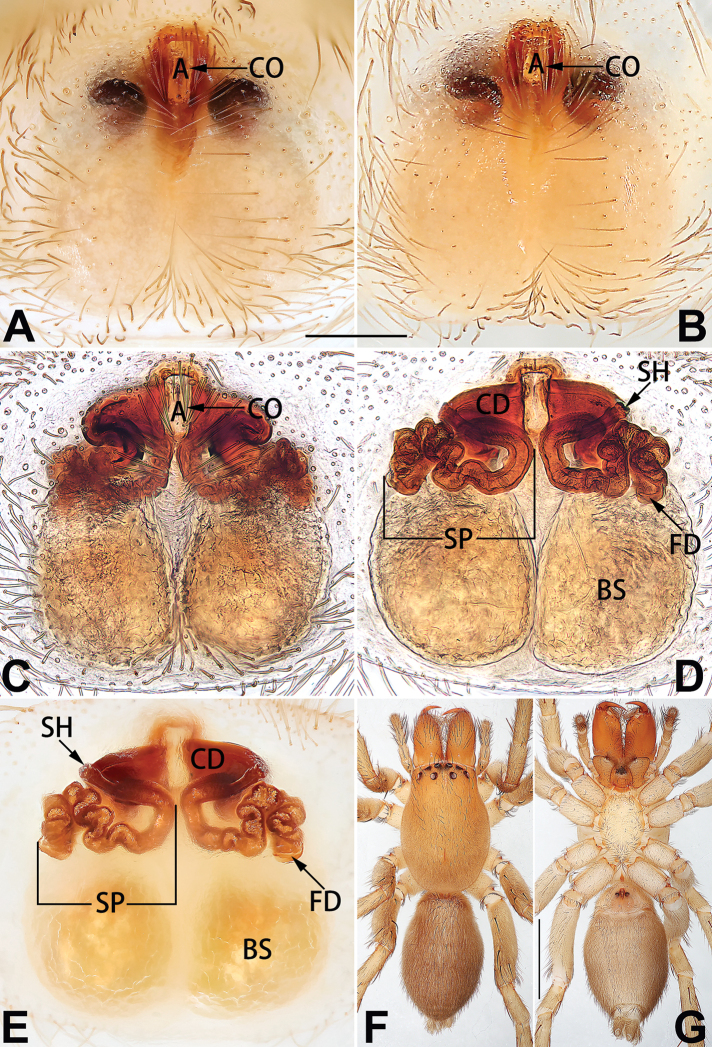
Holotype female of *Clubiona xiaokong* sp. nov., epigyne (**A–E**) and habitus (**F, G**) **A** intact, ventral view **B** cleared, ventral view **C** cleared, ventral view **D** cleared, dorsal view **E** cleared, dorsal view **F** dorsal view **G** ventral view. Abbreviations: A = atrium; BS = bursa; CD = copulatory duct; CO = copulatory opening; FD = fertilisation duct; SH = spermathecal head; SP = spermatheca. Scale bars: 0.2 mm (equal for **A–E**); 2 mm (equal for **F**, **G**).

Figs 22 , 74A , 82A , 90A

##### Holotype.

♀ (IZCAS-Ar 34753), China: Yunnan Province: Xishuangbanna: Mengla County: Menglun Town: XTBG, *Anogeissus acuminata*plantation (ca. 20 yr.), 21°53.819'N, 101°17.075'E, ca. 609 m, 27.XI.2009, G. Tang and Z.Y. Yao leg. ***Other material examined.*** 1♀ (YHCLU0078), XTBG, secondary tropical forest, 21°54.833'N, 101°16.781'E, ca. 617 m, 26.IV.2019, Z.G. Chen leg.

##### Etymology.

The specific name is derived from the Chinese pinyin *xiǎo kǒng*, which means small opening, referring to the small atrium; noun in apposition.

##### Diagnosis.

The new species is similar to *C. falciforma* (Liu et al. 2016: 567, figs 30, 31, 35, 36) by the ɔc-shaped spermathecae. From *C. falciforma*, the female of the new species can be easily distinguished by: the nearly trapezoidal atrium distinctly longer than wide (Figs 22A–C, 74A, 82A) (vs. elliptical atrium wider than long; Liu et al. 2016: figs 30, 35); copulatory openings located at lateral atrial borders (Figs 22A–C, 74A, 82A) (vs. situated basolaterally in atrium; Liu et al. 2016: figs 30, 35); (and the transverse copulatory ducts (Figs 22D, E, 90A) (vs. copulatory ducts descending longitudinally; Liu et al. 2016: figs 31, 36).

##### Description.

**Female.** Holotype (Fig. 22F, G): Total length 6.48; carapace 3.28 long, 2.26 wide; opisthosoma 3.19 long, 2.03 wide. Carapace elongate, oval, light brown, uniformly coloured, without pattern, fovea red; pars cephalica slightly narrowed, cervical groove indistinct; tegument smooth, with erect, thin, dark setae on front ridge. Eyes: In dorsal view, AER slightly recurved, PER almost straight, PER wider than AER. Eye sizes and interdistances: AME 0.13, ALE 0.16, PME 0.14, PLE 0.12, AME–AME 0.16, AME–ALE 0.11, PME–PME 0.35, PME–PLE 0.25, MOQL 0.36, MOQA 0.46, MOQP 0.68. Chelicerae protruding and robust, coloured as carapace, with long, orange fangs, with five promarginal and two retromarginal teeth. Sternum nearly white, 1.60 long, 1.10 wide. Labium and endites coloured as carapace. Legs light coloured, dorsally slightly darker, without markings. Leg measurements: I 6.23 (1.76, 2.60, 1.18, 0.68), II 6.87 (2.04, 2.68, 1.34, 0.81), III 5.97 (1.85, 2.15, 1.39, 0.57), IV 8.26 (2.35, 2.72, 2.41, 0.78). Abdomen oval and light brown, without pattern, dorsum densely covered with long, dark setae on a light background; venter sparsely covered with short, white setae.

Epigyne (Figs 22A–E, 74A, 82A, 90A). Epigynal plate slightly longer than wide, margin not rebordered; spermathecae and bursae indistinctly visible through transparent integument. Atrium represented by small pore, ca. 2 × longer than wide, ca. 1/6 of epigyne length and 1/11 of epigyne width. Copulatory openings large, situated laterally on the atrium. Copulatory ducts short and thick, heavily sclerotised, expanding laterally, then connecting to tubular spermathecae. Spermathecae long, consisting of smooth proximal half and wrinkled distal half, with small fertilisation ducts terminally, proximal half shaped like ɔc, the distal half irregularly shaped. Bursae reniform, close together, distinctly larger than spermathecae, 1.3 × longer than wide, surface translucent and smooth.

**Male.** Unknown.

##### Distribution.

Known only from the type locality, Xishuangbanna, Yunnan, China.

#### Clubiona yejiei

Taxon classificationAnimaliaAraneaeClubionidae

Yu & Li sp. nov.

EA3DD79B-0ED0-51BF-A944-31F7AF6A9355

http://zoobank.org/2EDB9A18-CA1C-4C1E-BA1B-3D83D01DC25A

**Figure 23.**
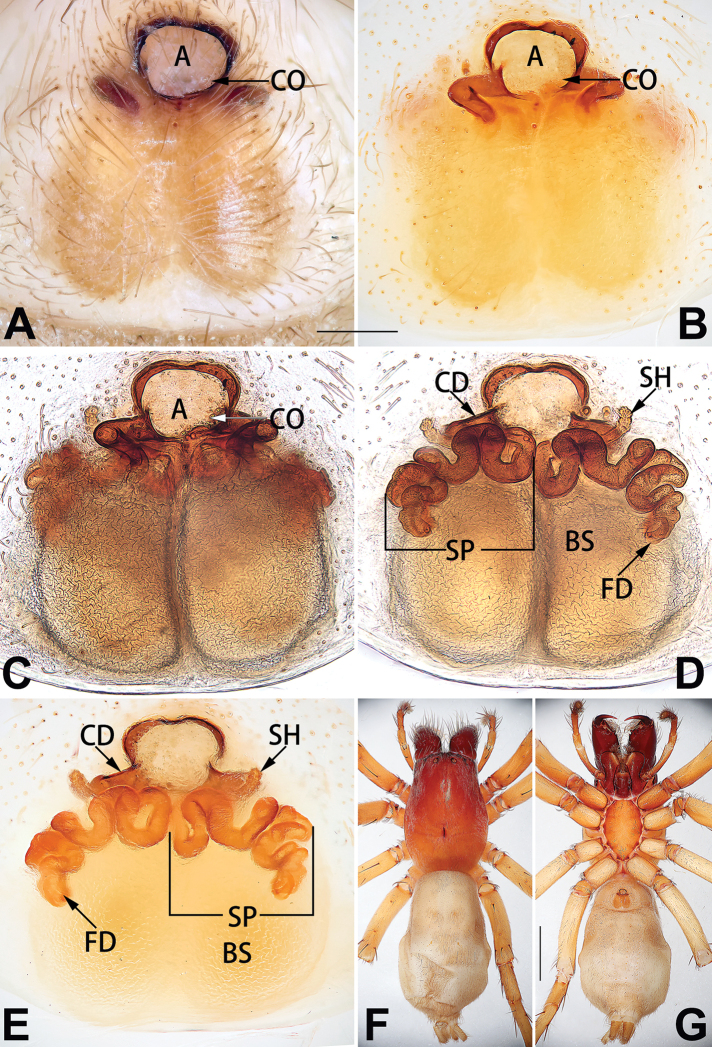
Holotype female of *Clubiona yejiei* sp. nov., epigyne (**A–E**) and habitus (**F, G**) **A** intact, ventral view **B** cleared, ventral view **C** cleared, ventral view **D** cleared, dorsal view **E** cleared, dorsal view **F** dorsal view **G** ventral view. Abbreviations: A = atrium; BS = bursa; CD = copulatory duct; CO = copulatory opening; FD = fertilisation duct; SH = spermathecal head; SP = spermatheca. Scale bars: 0.2 mm (equal for **A–E**); 1 mm (equal for **F**, **G**).

Figs 23 , 73C , 81C , 89C

##### Holotype.

♀ (IZCAS-Ar 34754), China: Yunnan Province: Xishuangbanna: Mengla County: Menglun Town: XTBG, *Paramichelia baillonii*plantation, 21°53.823'N, 101°17.072'E, ca. 613 m, 1–17.II.2007, G. Zheng leg.

##### Etymology.

This species is named after Mr. Yejie Lin (Beijing City, China) who has helped us greatly with this research.

##### Diagnosis.

The new species is similar to *C. cochlearis* (Figs 1A–E, 73A, 81A, 89A) in the general appearance of the atrium and endogyne. From *C. cochlearis*, the female of the new species can be easily distinguished by the shape of the atrium and spermathecae: (1) atrium ellipsoid (Figs 23A–C, 73C, 81C) (vs. atrium apple-shaped; Figs 1A–C, 73A, 81A); (2) spermathecae thicker and not twisted, diameter ca. 1/3 of atrium length (Figs 23D, E, 89C) (vs. thinner and strongly convoluted, diameter not more than 1/8 of atrium length; Figs 1D, E, 89A).

##### Description.

**Female.** Holotype (Fig. 23F, G). Total length 9.47; carapace 4.47 long, 2.56 wide; opisthosoma 5.50 long, 3.82 wide. Carapace brownish red, pars cephalica slightly darker in ocular area, without markings, cephalic region slightly narrowed, cervical groove indistinct; tegument smooth, covered with numerous fine setae. Eyes: AER slightly recurved, PER slightly procurved, PER wider than AER. Eye sizes and interdistances: AME 0.17, ALE 0.17, PME 0.17, PLE 0.09, AME–AME 0.25, AME–ALE 0.85, PME–PME 0.45, PME–PLE 0.47, MOQL 0.55, MOQA 0.58, MOQP 0.79. Chelicerae robust and protruding, coloured as ocular area, with four promarginal and two retromarginal teeth. Sternum centrally orange and marginally red, 2.13 long, 1.36 wide. Labium and endites coloured as chelicerae. Legs light orange, uniformly coloured, without pattern. Leg measurements: I 8.64 (2.33, 3.63, 2.03, 0.64), II 8.73 (2.76, 3.60, 1.44, 0.93), III 8.16 (2.51, 2.96, 1.77, 0.92), IV 10.54 (3.32, 3.48, 2.60, 1.14). Abdomen uniformly cream coloured, dorsum with two pairs of conspicuous muscle depressions; venter medially with two longitudinal broken lines.

Epigyne (Figs 23A–E, 73C, 81C, 89C). Epigynal plate slightly longer than wide, through which bursae and copulatory ducts are easily visible. Atrium shaped like an irregular ellipse, ca. 1.3 × wider than long, ca. 1/5 epigyne length. Copulatory openings indistinct, located at basolateral atrial borders. Copulatory ducts short and thick, heavily sclerotised, running laterally, bent 90 degrees dorsally and then obliquely descending, connecting with tubular spermathecae. Spermathecae long and sinuous, with even thickness throughout. Fertilisation ducts acicular, membranous, located terminally on spermathecae. Bursae large and oblong, close together, ca. 1.5 × longer than wide, surface translucent and wrinkled.

**Male.** Unknown.

##### Distribution.

Known only from the type locality, Xishuangbanna, Yunnan, China.

#### Clubiona zhaoi

Taxon classificationAnimaliaAraneaeClubionidae

Yu & Li sp. nov.

6809F123-0C64-50E7-B3E1-C21BD9DE7F71

http://zoobank.org/DB382D68-B622-49C7-A799-773EDB02E05E

**Figure 24.**
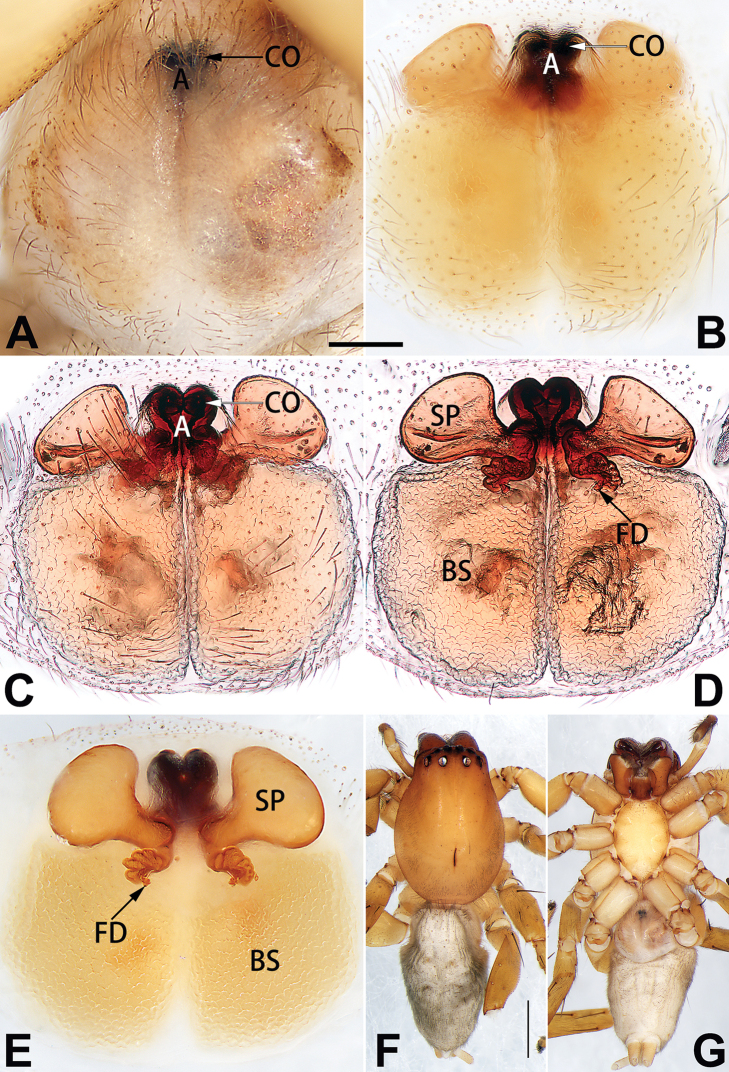
Holotype female of *Clubiona zhaoi* sp. nov., epigyne (**A–E**) and habitus (**F, G**) **A** intact, ventral view **B** cleared, ventral view **C** cleared, ventral view **D** cleared, dorsal view **E** cleared, dorsal view **F** dorsal view **G** ventral view. Abbreviations: A = atrium; BS = bursa; CO = copulatory opening; FD = fertilisation duct; SP = spermatheca. Scale bars: 0.2 mm (equal for **A–E**); 1 mm (equal for **F**, **G**).

Figs 24 , 74B , 82B , 90B

##### Holotype.

♀ (IZCAS-Ar 34755), China, Yunnan Province: Xishuangbanna: Mengla County: Xiaolongha Village: biodiversity preservation corridor, 21°24.798'N, 101°37.880'E, ca. 693 m, 28.VI.2012, Q.Y. Zhao and C.X. Gao leg. ***Other material examined.*** 1♀ (YHCLU0086), same locality, same time, and same collectors as holotype, 21°24.408'N, 101°37.827'E, ca. 662 m.

##### Etymology.

The specific name is a patronym after Qingyuan Zhao (Beijing City, China), collector of several specimens examined in this study.

##### Diagnosis.

Females of the new species are easily distinguished from others in the species group, with the exception for *C. altissimoides* (Liu et al. 2007: 65, figs 6, 7), by the similarly shaped atria and the general shape of the endogyne. *Clubiona zhaoi* sp. nov. can be separated from *C. altissimoides* by the copulatory openings located at the anterior atrial margins (Figs 24A–C, 74B, 82B) (vs. located at the underside of the atrium; Liu et al. 2007: fig. 6) and by having the spermathecae distinctly smaller than the bursae (Figs 24D, E, 90B) (vs. larger; Liu et al. 2007: fig. 7).

##### Description.

**Female.** Holotype (Fig. 24F, G): Total length 5.82; carapace 2.80 long, 1.98 wide; opisthosoma 3.02 long, 1.65 wide. Carapace light brown except dark brown ocular area, without distinct pattern; fovea dark; cephalic region distinctly narrowed, cervical groove distinct, radial grooves inconspicuous; tegument smooth, clothed with short, fine setae. Eyes: AER slightly recurved, PER slightly wider than AER, almost straight in dorsal view. Eye sizes and interdistances: AME 0.11, ALE 0.15, PME 0.12, PLE 0.08, AME–AME 0.11, AME–ALE 0.08, PME–PME 0.32, PME–PLE 0.19, MOQL 0.39, MOQA 0.37, MOQP 0.59. Chelicerae robust and brownish red, both margins with four teeth. Sternum yellowish brown, 1.38 long, 0.95 wide. Labium and endites coloured as carapace. Legs yellowish, without distinct markings. Leg measurements: I – (1.58, –, –, –), II 6.60 (1.89, 2.52, 1.38, 0.81), III 5.29 (1.64, 1.69, 1.43, 0.53), IV – (–, –, 1.17, –). Abdomen long, oval, dorsally grey with dense setae and a lengthwise white heart mark, reaching posterior half; with a pair of muscle depressions located at distal part of heart mark; venter off-white.

Epigyne (Figs 24A–E, 74B, 82B, 90B). Epigynal plate nearly as broad as long, spermathecae and bursae indistinctly visible through integument. Atrium small, ca. 1/4 of epigyne width, with M-shaped anterior margin (or hood), without posterior margin. Copulatory openings small but distinct, circular, located on anterior part of atrium. Copulatory ducts indistinct. Spermathecae consisting of fan-shaped proximal part and convoluted distal part, with small fertilisation ducts terminally; the two spermathecae separated by 0.8 diameters. Bursae oval, close together, ca. 1.4 × longer than wide, surface translucent and wrinkled.

**Male.** Unknown.

##### Distribution.

Known only from the type locality, Xishuangbanna, Yunnan, China.

#### Clubiona zhigangi

Taxon classificationAnimaliaAraneaeClubionidae

Yu & Li sp. nov.

D897AC49-B437-5C43-B346-4146C250E3FA

http://zoobank.org/03F6ECE2-ACAF-4DF3-9169-C04CF5BD84AF

**Figure 25.**
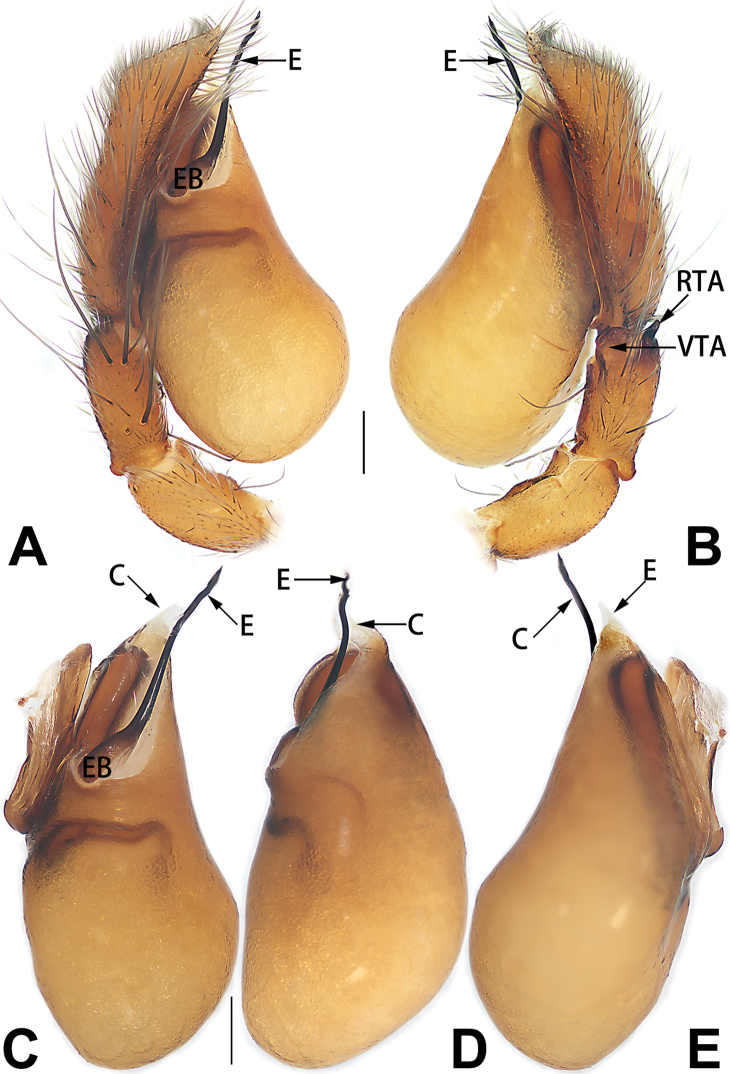
Male palp of the holotype of *Clubiona zhigangi* sp. nov. **A** prolateral view **B** retrolateral view **C** bulb, prolateral view **D** bulb, ventral view **E** bulb, retrolateral view. Abbreviations: C = conductor; E = embolus; EB = embolar base; RTA = retrolateral tibial apophysis; VTA = ventral tibial apophysis. Scale bars: 0.2 mm (equal for **A, B**, equal for **C–E**).

**Figure 26.**
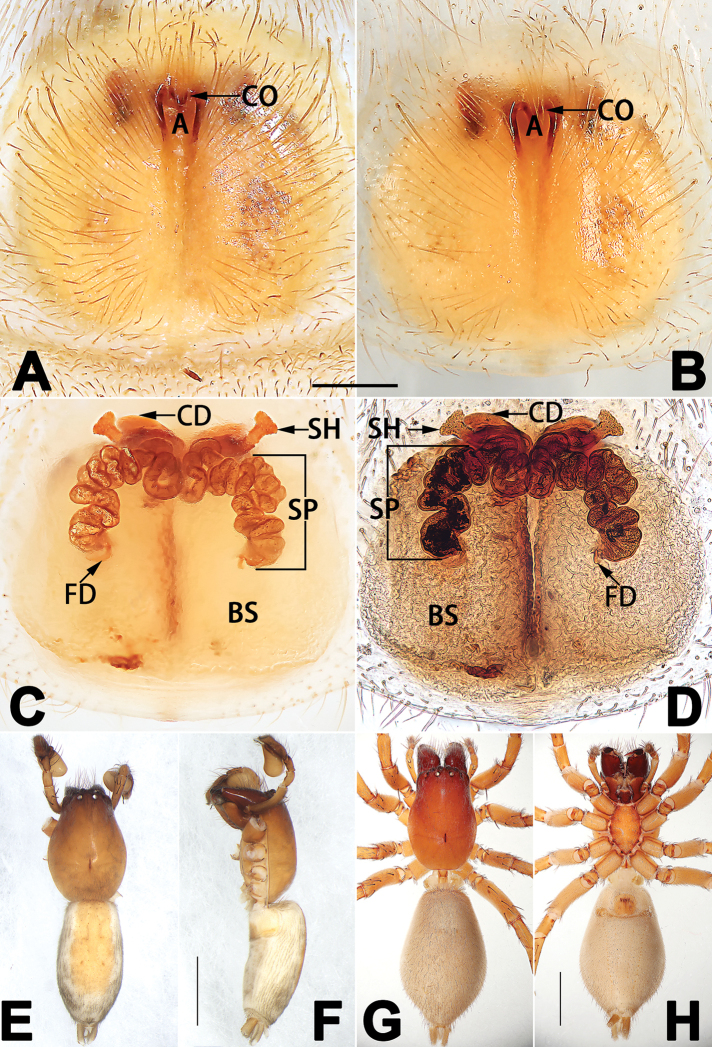
*Clubiona zhigangi* sp. nov., female paratype and male holotype, epigyne (**A–D**), male habitus (**E, F**) and female habitus (**G, H**) **A** intact, ventral view **B** cleared, ventral view **C** cleared, dorsal view **D** cleared, dorsal view **E** dorsal view **F** lateral view **G** dorsal view **H** ventral view. Abbreviations: A = atrium; BS = bursa; CD = copulatory duct; CO = copulatory opening; FD = fertilisation duct; SP = spermatheca; SH = spermathecal head. Scale bars: 0.2 mm (equal for **A–D**); 2 mm (equal for **E, F**, equal for **G, H**).

Figs 25 , 26 , 53D , 63D , 73F , 81F , 89F

##### Holotype

♂ (IZCAS-Ar 34756, YHCLU0185), China: Yunnan Province: Xishuangbanna: Mengla County: Menga Town: XTBG, secondary tropical forest, 21°53.634'N, 101°17.172'E, ca. 620 m, 28. IV.2019, Z.G. Chen et al. leg. ***Paratype***: 1♀ (IZCAS-Ar 34757), XTBG, secondary tropical seasonal moist forest, 21°54.718'N, 101°16.940'E, ca. 645 m, 27. VII.2007, G. Zheng leg. ***Other material examined.*** 1♀ (YHCLU0138), China: Yunnan Province: Xishuangbanna: Mengla County: Menga Town: XTBG, *Paramichelia baillonii*plantation, 21°53.823'N, 101°17.072'E, ca. 613 m, 15. IV.2007, G. Zheng leg.

##### Etymology.

The specific name is a patronym after Zhigang Chen (Beijing City, China), collector of several specimens used in this study.

##### Diagnosis.

Males of *C. zhigangi* sp. nov. resemble those of *C. subrama* (Yu and Li 2019a: 153, fig. 3A–E; Figs 53C, 63C) in having similar retrolateral and ventral tibial apophyses and a slender embolus but differ by: (1) a thicker embolus, with a sinuate apex (Figs 25A–E, 53D, 63D) (vs. filiform embolus, thinner, embolar tip not curved; Figs 53C, 63C); (2) short conductor, ca. 1/5 of tegulum length, with a blunt tip (Fig. 25C–E) (vs. conductor long, not less than 1/3 of tegulum length, with a sharp tip; Fig. 53C). Females also resemble those of *C. subrama* in having an anteriorly cordiform and posteriorly elongate, narrowed atrium and the general shape of the endogyne but can be distinguished from the latter by the distinctly shorter copulatory ducts not convoluted (Figs 26C, D, 89F) (vs. long copulatory ducts strongly entwined, moving longitudinally and expanding obliquely 2 ×, respectively, forming two horizontal loops; Fig. 89E).

##### Description.

**Male.** Holotype (Fig. 26E, F). Total length 5.91; carapace 2.85 long, 2.17 wide; opisthosoma 3.06 long, 1.88 wide. Carapace brown, darker in the front, without distinct pattern, fovea red; cephalic region distinctly narrowed, cervical groove and radial grooves indistinct; tegument smooth, clothed with short, fine setae. Eyes: AER slightly recurved, PER wider than AER and slightly procurved in dorsal view. Eye sizes and interdistances: AME 0.13, ALE 0.12, PME 0.14, PLE 0.11, AME–AME 0.19, AME–ALE 0.13, PME–PME 0.30, PME–PLE 0.24, MOQL 0.36, MOQA 0.32, MOQP 0.53. Chelicerae robust, red wine coloured, with three promarginal and two retromarginal teeth. Sternum pale brown, 1.64 long, 1.02 wide. Labium and endites coloured as carapace. All legs missing. Abdomen elongate, oval, dorsal scutum trapezoidal, lightly sclerotised, with a thick tuft of setae anteriorly; dorsum brown, with dense setae, with a pair of muscle depressions located at central part of dorsal scutum; venter grey.

Palp (Figs 25A–E, 53D, 63D). Femur and patella unmodified. Tibia short, ca. 1/2 of cymbium length, with 2 apophyses: a large, semi-circular ventral one, ca. 1/3 of palpal tibia length, and a relatively small, claw-shaped retrolateral apophysis. Tegulum elongate, oval, 1.9 × longer than wide; bulb strongly bulging and prolapsed, sperm duct indistinct in ventral view. Embolus slender, originating from prolateral side of tegulum, ca. 1/2 of tegulum length, tip sinuate and extending above the apex of the cymbium. Conductor short, membranous, ca. 1/3 of the embolus length, originating from retrolateral side of tegulum, with basal torsion and a distal finger-like point, tip hidden behind embolus.

**Female.** Paratype (Fig. 26G, H). Total length 8.51; carapace 3.56 long, 2.48 wide; opisthosoma 4.95 long, 3.05 wide. Eye sizes and interdistances: AME 0.16, ALE 0.19, PME 0.15, PLE 0.15, AME–AME 0.12, AME–ALE 0.11, PME–PME 0.35, PME–PLE 0.28, MOQL 0.36, MOQA 0.46, MOQP 0.65. Sternum 1.88 long, 1.14 wide. Leg measurements: I 5.99 (1.17, 2.33, 1.13, 0.74), II 6.74 (1.99, 2.67, 1.29, 0.79), III 5.89 (1.74, 2.11, 1.51, 0.54), IV 8.05 (2.31, 2.82, 2.30, 0.62). Distinctly larger and darker than male, other characters as in male.

Epigyne (Figs 26A–E, 73F, 81F, 89F). Epigynal plate slightly wider than long, spermathecae indistinctly visible and bursae prominently visible through integument in ventral view. Atrium small and elongate, narrow, ca. 1/2 × epigyne length and 1/7 × epigyne width, atrial anterior margin M-shaped, posterior margin not delimited. Copulatory openings small, located on anterolateral margin of atrium. Copulatory ducts directed laterally then ascending obliquely, connecting to spermathecae at central axis of the vulva. Spermathecae tubular, long and sinuous, strongly twisted. Fertilisation ducts acicular, curved, located terminally on spermathecae. Bursae reniform, large, close together, ca. 1.5 × longer than wide, surface translucent and wrinkled.

##### Distribution.

Known only from the type locality, Xishuangbanna, Yunnan, China.

#### Clubiona ternatensis

Taxon classificationAnimaliaAraneaeClubionidae

group

E0E32BC8-B6DE-555A-B263-E393AD91C451

Hirtia Thorell, 1881: 222 (type species H. ternatensis Thorell, 1881).
Clubiona : Simon 1897: 76 (synonymised Hirtia).
Clubiona hystrix group: Deeleman-Reinhold 2001: 90.

##### Diagnosis.

See Deeleman-Reinhold (2001) and Yu and Li (2019a).

##### Description.

See Deeleman-Reinhold (2001).

##### Composition and distribution.

The *Clubiona ternatensis* group tentatively contains 33 species (see Table 4).

##### Comments.

This group was defined by Deeleman-Reinhold (2001) for Oriental species. However, the three first described species were placed in the genus *Hirtia* Thorell, 1881, and *H. ternatensis* was chosen as the type species by Thorell (1881). Simon (1897) synonymised *Hirtia* with *Clubiona*. The recently discovered species of the group are strikingly different from *Clubiona pallidula* (Clerck, 1757) (type species of *Clubiona*). It seems that *Hirtia* should be resurrected in the future.

**Table 4.** Species in the *Clubiona ternatensis* group.

	Species name	Known sex	Distribution
1	*C. analis* Thorell, 1895	♀	India (West Bengal), Bangladesh (Barisal), Myanmar (Moulmein)
2	*C. bachmaensis* Ono, 2009	♂	Vietnam
3	*C. bengalensis* Biswas, 1984	♀	India (Maharashtra)
4	*C. brevispina* Huang & Chen, 2012	♂♀	China (Taiwan)
5	*C. chathamensis* Simon, 1905	♂	New Zealand (Chatham Is.)
6	*C. damirkovaci* Deeleman-Reinhold, 2001	♂♀	Malaysia (Kuala Lumpur)
7	*C. ericius* Chrysanthus, 1967	♂♀	New Guinea
8	*C. esuriens* Thorell, 1897	♂	Myanmar (specific locality not clear)
9	*C. hatamensis* (Thorell, 1881)	♂	New Guinea
10	*C. heteroducta* Zhang & Yin, 1998	♀	China (Yunnan)
11	*C. hitchinsi* Saaristo, 2002	♂♀	Seychelles, French Polynesia
12	*C. hystrix* Berland, 1938	♂♀	Indonesia (Lesser Sunda Is.)
13	*C. jaegeri* Ono, 2011	♂	Palau Is.
14	*C. kapataganensis* Barrion & Litsinger, 1995	♀	Philippines (Laguna)
15	*C. kowong* Chrysanthus, 1967	♂♀	New Guinea
16	*C. kuu* Jäger & Dankittipakul, 2010	♂	Laos
17	*C. maipai* Jäger & Dankittipakul, 2010	♂♀	Thailand (Mae Hong Son)
18	*C. meraukensis* Chrysanthus, 1967	♂♀	New Guinea
19	*C. oceanica* Ono, 2011	♂♀	Japan (Chichijima Is.)
20	*C. pantherina* Chrysanthus, 1967	♂♀	New Guinea
21	*C. papuana* Chrysanthus, 1967	♀	New Guinea
22	*C. paranghinlalakirta* Barrion & Litsinger, 1995	♂	Philippines (Misamis Oriental)
23	*C. pseudomaxillata* Hogg, 1915	♀	New Guinea
24	*C. pseudopteroneta* Raven & Stumkat, 2002	♂♀	Australia (Queensland)
25	*C. ramoiensis* (Thorell, 1881)	♀	New Guinea
26	*C. sertungensis* Hayashi, 1996	♂♀	Indonesia (Krakatau)
27	*C. subkuu* Yu & Li, 2019	♂♀	China (Yunnan)
28	*C. ternatensis* (Thorell, 1881)	♀	Indonesia (Moluccas)
29	*C. theoblicki* Yu & Li, 2019	♂♀	China (Yunnan)
30	*C. tongi* Yu & Li, 2019	♂♀	China (Yunnan)
31	*C. zhengi* Yu & Li, 2019	♂♀	China (Yunnan)
32	*C. mii* Yu & Li, sp. nov.	♀	China (Yunnan)
33	*C. subtongi* Yu & Li, sp. nov.	♂	China (Yunnan)

#### Key to *C. ternatensis* group species occurring in Xishuangbanna (males)

Male of *C. mii* sp. nov. is unknown.

**Table:** 

1	Palp with two tibial apophyses (Figs 57E, 67E)	***C. zhengi***
–	Palp with single tibial apophysis	**2**
2	Emblous short, tip extending to 1/3 tegulum (Figs 57C, 67C)	***C. subkuu***
–	Emblous distinctly long, tip extending basad more than 4/5 × length of tegulum (Figs 57A, B, D, 67A, B, D)	**3**
3	Tegular hump nearly quadrate (Figs 57A)	***C. theoblicki***
–	tegular hump with a blunt and semi-circular tip, resembling a wave crest in ventral view (Figs Fig. 57B, D)	**4**
4	Embolar apex terminating at ca. 5 o’clock position of tegulum; tegular hump is ca. 1/3 tegulum length; tegular base with a papilliform flange (Figs 57B, 67B)	***C. tongi***
–	Embolar tip terminating at approximately 4 o’clock position; tegular hump ca. 1/5 tegulum length; tegular base is unmodified (Figs 57D, 67D)	***C. subtongi* sp. nov.**

#### Key to *C. ternatensis* group species occurring in Xishuangbanna (females)

*C. heteroducta* is excluded due to lack of specimens.

**Table:** 

1	Epigyne with atrium (Figs 77E, 85E, 93E)	***C. zhengi***
–	Epigyne without atrium	**2**
2	Copulatory ducts short, not longer than epigyne length, not convoluted (Fig. 93A, B)	**3**
–	Copulatory ducts long, > 2 × longer than epigyne length, strongly convoluted (Fig. 93C, D)	**4**
3	Epigynal ridges triangular (Figs 77A, 85A)	***C. mii* sp. nov.**
–	Epigynal ridges more or less blade-shaped (Figs 77B, 85B)	***C. subkuu***
4	Epigynal ridges diagonal (Figs 77C, 85C); anterior part of copulatory ducts forming 2 longitudinal loops (Fig. 93C)	***C. theoblicki***
–	Epigynal ridges longitudinal (Figs 77D, 85D); anterior part of copulatory ducts with 2 transversal loops (Fig. 93D)	***C. tongi***

#### Clubiona heteroducta

Taxon classificationAnimaliaAraneaeClubionidae

Zhang & Yin, 1998

4DDD6DF5-CD3B-5BAF-B3E6-F056F8D33C1D

Clubiona heteroducta Zhang & Yin, 1998: 12, figs 12, 13 (♂♀).

##### Material examined.

None.

##### Diagnosis and description.

See Zhang and Yin (1998).

##### Distribution.

China (Yunnan).

#### Clubiona mii

Taxon classificationAnimaliaAraneaeClubionidae

Yu & Li sp. nov.

862FA171-CCCC-5B61-AA6B-EE79FBFE1F51

http://zoobank.org/3C857D44-FF2D-4C7B-B459-4B83F0AB2361

**Figure 27.**
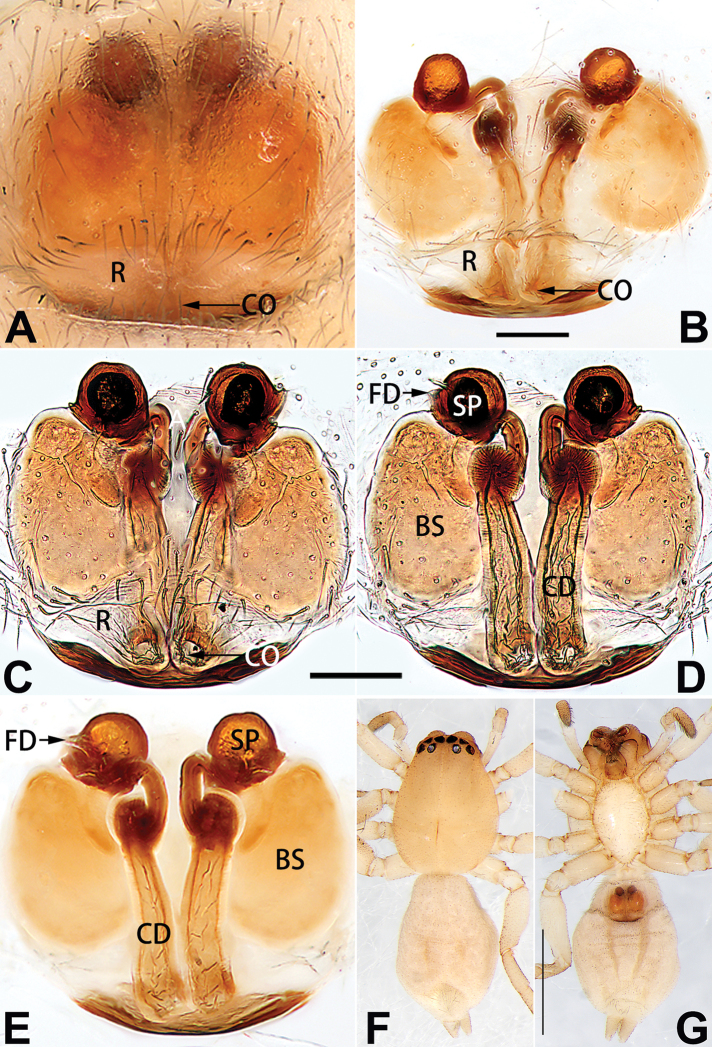
Holotype female of *Clubiona mii* sp. nov., epigyne (**A–E**) and habitus (**F, G**) **A** intact, ventral view **B** cleared, ventral view **C** cleared, ventral view **D** cleared, dorsal view **E** cleared, dorsal view **F**dorsal view **G** ventral view. Abbreviations: BS = bursa; CD = copulatory duct; CO = copulatory opening; FD = fertilisation duct; R = epigynal ridge; SP = spermatheca. Scale bars: 0.1 mm (equal for **A–E**); 1 mm (equal for **F**, **G**).

Figs 27 , 77A , 85A , 93A

##### Holotype

♀ (IZCAS-Ar 34758, YHCLU0065), China, Yunnan Province: Xishuangbanna: Mengla County: Nanshahe Village: monsoon forest, 21°36.388'N, 101°34.247'E, ca. 797 m, 13.VII.2012, Q.Y. Zhao and C.X. Gao leg.

##### Etymology.

This species is named after Mr. Xiaoqi Mi (Tongren City, China) who has helped us greatly with this research.

##### Diagnosis.

The female of the new species is easily distinguished from those of the other species in the group, with the exception of *C. hystrix* (Deeleman-Reinhold 2001: 103, figs 17, 18), by the general shape of the vulva but can be recognised by the: (1) triangular epigynal ridge (Figs 27A–C, 77A, 85A) (vs. pocket-like; Deeleman-Reinhold 2001 fig. 17); (2) copulatory openings close together (Figs 27A–C, 77A, 85A) (vs. copulatory openings separated by one diameter; Deeleman-Reinhold 2001 fig. 17).

##### Description.

**Female.** Holotype (Fig. 27F, G): total length 2.63; carapace 1.29 long, 0.99 wide; opisthosoma 1.34 long, 0.90 wide. Carapace light orange, darker anteriorly, without distinct pattern, pars cephalica slightly narrowed, cervical groove indistinguishable; tegument smooth, all setae detached in ethanol. Eyes: AER slightly recurved, PER slightly wider than AER, almost straight in dorsal view. Eye sizes and interdistances: AME 0.08, ALE 0.07, PME 0.07, PLE 0.06, AME–AME 0.04, AME–ALE 0.02, PME–PME 0.17, PME–PLE 0.05, MOQL 0.18, MOQA 0.22, MOQP 0.31. Chelicerae coloured as ocular area, with six promarginal and two retromarginal teeth. Sternum yellowish white, 1.38 long, 0.95 wide. Labium and endites coloured as chelicerae. Legs coloured as sternum, without markings. Leg measurements: I 1.84 (0.53, 0.73, 0.33, 0.25), II 1.98 (0.58, 0.77, 0.40, 0.23), III 1.81 (0.58, 0.59, 0.41, 0.22), IV 2.85 (0.83, 1.00, 0.71, 0.30). Abdomen yellowish white, uniformly coloured, clothed with dense setae, without pattern.

Epigyne (Figs 27A–E, 77A, 85A, 93A). Epigynal plate slightly longer than wide, spermathecae and bursae prominently visible through integument in ventral view, posterior margin not rebordered. Copulatory openings small, contiguous, situated at medial portion of epigynal plate posterior margin, hidden in two transverse ridges in ventral view. Ridge represented by triangular sclerite. Copulatory ducts thick and straight, close together, ascending parallel, entering the connecting piece located inside of bursal surface, then continuing upward and turning sideways, finally connecting to anteriorly located spermathecae. Spermathecae globular, separated by one diameter. Fertilisation ducts small, acicular. Bursae oblong, large, approximately as long as copulatory ducts, separated by 0.5 × diameters, ca. 1.5 × longer than wide, with smooth surface.

**Male.** Unknown.

##### Comments.

According to WSC (2021), a total of seven described *C. ternatensis* group species are known only from males (See Table 4). Among them, *C. bachmaensis*, *C. esuriens*, and *C. kuu* were found form the adjacent area of Xishuangbanna. We cannot rule out the possibility that these three species are conspecific to *C. mii* sp. nov.

##### Distribution.

Known only from the type locality, Xishuangbanna, Yunnan, China.

#### Clubiona subkuu

Taxon classificationAnimaliaAraneaeClubionidae

Yu & Li, 2019

C7CF5BC1-ECC2-5393-844E-44DD9FD75FA1

Figs 57C , 67C , 77B , 85B , 93B

Clubiona subkuu Yu & Li, 2019a: 164, figs 9A–E, 10A–H (♂♀).

##### Material examined.

***Types*.** Holotype ♂ (IZCAS Ar 34604), 1♀ (paratype, IZCAS Ar 34605), China: Yunnan Province: Xishuangbanna: Mengla County: Menglun Town: XTBG, Lvshilin Forest Park, limestone tropical seasonal rainforest, 21°54.555'N, 101°16.860'E, ca. 610 m, 29.XI.2009, G. Tang and Z.Y. Yao leg. ***Other material examined.*** 1♂ (YHCLU0039), XTBG, G213 roadside, leprosy village, 21°53.593'N, 101°17.329'E, ca. 559 m, 5.VIII.2018, H. Yu et al. leg; 1♀ (YHCLU0038), XTBG, secondary tropical forest, 21°54.833'N, 101°16.781'E, ca. 575 m, 31.VII.2018, Z.G. Chen leg.

##### Diagnosis and description.

See Yu and Li (2019a). Male palp as in Figs 57C, 67C, epigyne as in Figs 77B, 85B, 93B.

##### Distribution.

Known only from Xishuangbanna.

##### Most similar species.

*Clubiona kuu*.

#### Clubiona subtongi

Taxon classificationAnimaliaAraneaeClubionidae

Yu & Li sp. nov.

9EF03641-91D8-5F34-AA96-850307507501

http://zoobank.org/071280B5-EB72-4445-9605-EAC6699CF789

**Figure 28.**
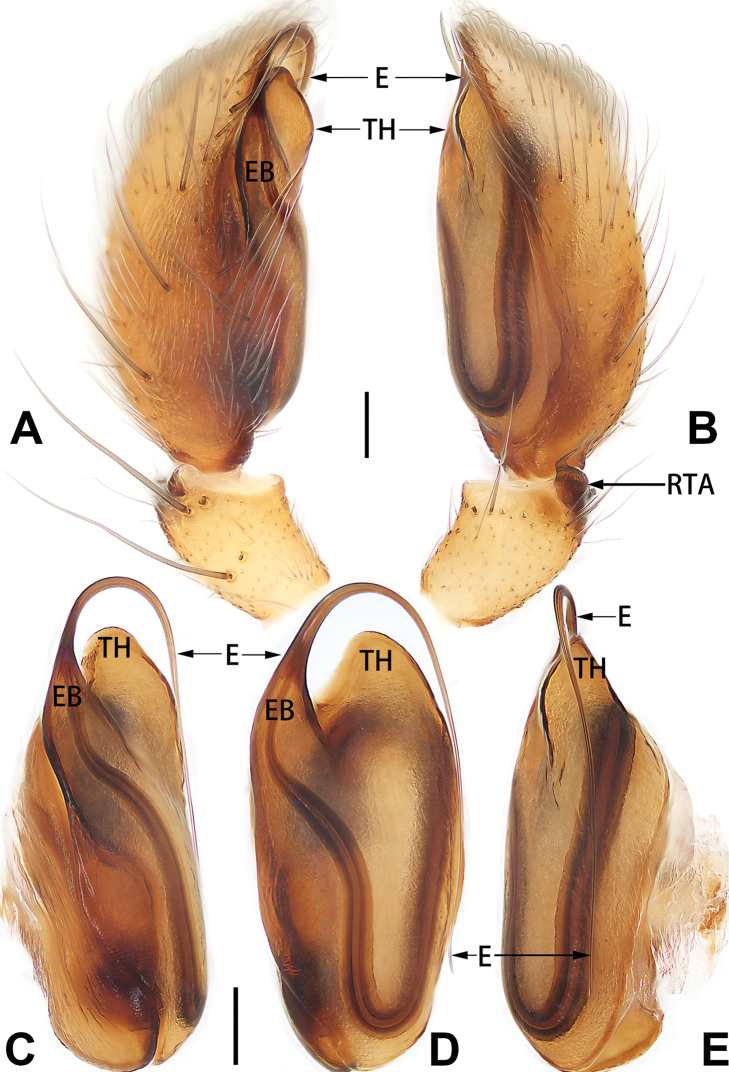
Male palp of the holotype of *Clubiona subtongi* sp. nov. **A** prolateral view **B** retrolateral view **C** bulb, prolateral view **D** bulb, ventral view **E** bulb, retrolateral view. Abbreviations: E = embolus; EB = embolar base; RTA = retrolateral tibial apophysis; TH = tegular hump. Scale bars: 0.1 mm (equal for **A, B**, equal for **C–E**).

**Figure 29.**
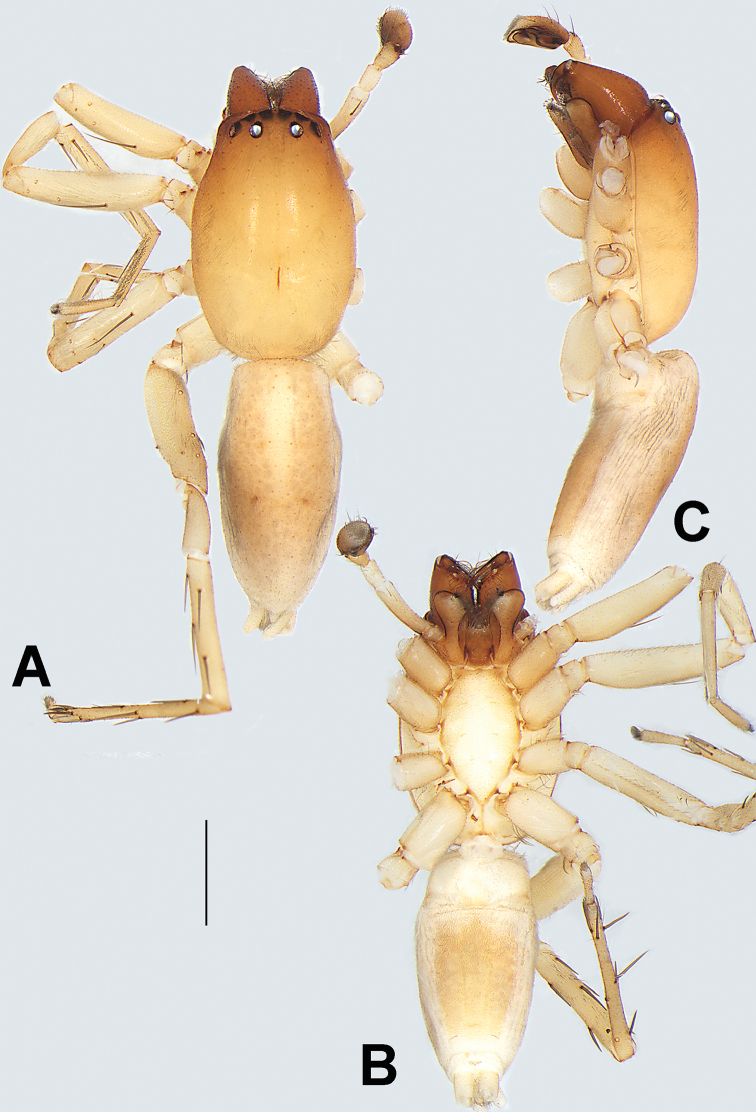
Habitus of the male holotype of *Clubiona subtongi* sp. nov. **A** dorsal view **B** ventral view **C** lateral view. Scale bar: 1 mm (equal for **A–C**).

Figs 28 , 29 , 57D , 67D

##### Holotype

♂ (IZCAS-Ar 34759, YHCLU0056), : Yunnan Province: Xishuangbanna: Mengla County: Menglun Town: XTBG, *Anogeissus acuminata*plantation, 21°54.033'N, 101°16.900'E, ca. 606 m, 2.VIII.2018, Z.G. Chen et al. leg.

##### Etymology.

The specific name is taken from its similarity to *Clubiona tongi*; modified noun (name) in genitive case..

##### Diagnosis.

*Clubiona subtongi* sp. nov. resembles *C. tongi* (Yu and Li 2019b: 212, figs 9A–E, 10E, F; Figs 57B, 67B) by having similar pale colouration and habitus (Fig. 29; Yu and Li 2019b: fig. 10E, F) and general shape of the palp (Figs 28A–E, 57B, 67B; Yu and Li 2019b: 212, fig. 9A–E) but can be distinguished by the: (1) embolar apex terminating at approximately the 4 o’clock position (Figs 28D, 57D) (vs. relatively longer tip terminating at approximately the 5 o’clock position; Figs 57B, 67B); (2) tegular hump that is ca. 1/5 tegulum length (Figs 28D, 57D) (vs. tegular hump ca. 1/3 tegulum length; Fig. 57B); (3) tegular base is unmodified (Figs 28B, 57D, 67D) (vs. with a papilliform flange; Fig. 67B).

##### Description.

**Male.** Holotype (Fig. 29): Total length 4.63; carapace 2.13 long, 1.54 wide; opisthosoma 2.50 long, 1.16 wide. Carapace light brown, slightly darker on the front ridge, without pattern, ocular area slightly narrowed, cervical groove indistinct; tegument smooth, marginally clothed with long, thin setae. Eyes: in dorsal view, AER slightly recurved, PER slightly procurved, PER slightly wider than AER. Eye sizes and interdistances: AME 0.09, ALE 0.10, PME 0.12, PLE 0.11, AME–AME 0.06, AME–ALE 0.03, PME–PME 0.26, PME–PLE 0.15, MOQL 0.33, MOQA 0.30, MOQP 0.46. Chelicerae brownish red, promargin with six teeth, retromargin with three teeth. Sternum yellowish white, 1.38 long, 0.95 wide. Labium and endites coloured as carapace. Legs yellowish white, without distinct markings. Leg measurements: I 4.28 (1.30, 1.70, 0.80, 0.48), II 4.70 (1.29, 1.98, 0.93, 0.50), III 3.83 (1.13, 1.28, 0.94, 0.49), IV 6.22 (1.88, 2.05, 1.79, 0.49). Abdomen lanceolate, dorsally grey with a lengthwise white heart shaped mark, reaching posterior half; with a pair of muscle depressions located on both sides of heart-shaped mark; venter centrally with an inverted trapezoidal orange patch.

Palp (Figs 28, 57D, 67D). Femur and patella unmodified. Tibia short, ca. 1/3 of cymbium length, with single retrolateral apophysis; RTA small, ca. 1/3 palpal tibia length, with a thumb-like base and spine-like tip. Tegulum elongate, oval, relatively flat, 2.1 × longer than wide, sperm duct distinct, V-shaped; tegular hump prominent, ca. 1/5 of tegulum length. Embolus filiform, originating on the retrolateral flank (ca. 10–11 o’clock on tegulum), aligning clockwise along the tegular hump, apex filiform, terminating at ca. 4 o’clock position.

**Female.** Unknown.

##### Comments.

According to the WSC (2021), a total of eight described *C. ternatensis* group species are known only from females (See Table 4). We describe this species based on the male, although it may be synonymised in future.

##### Distribution.

Known only from the type locality, Xishuangbanna, Yunnan, China.

#### Clubiona theoblicki

Taxon classificationAnimaliaAraneaeClubionidae

Yu & Li, 2019

BD53B7FC-8EBB-5729-98F2-4C1306249EF3

Figs 57A , 67A , 77C , 85C , 93C

Clubiona quadrata Yu & Li, 2019a: 161, figs 7A–E, 8A–H (♂♀)
Clubiona theoblicki Yu & Li, 2019c: 40 (replacement name).

##### Material examined.

***Type*.** Holotype ♂ (IZCAS Ar 34568), China: Yunnan Province: Xishuangbanna: Mengla County: Menglun Town: XTBG, secondary tropical forest, 21°54.380'N, 101°16.815'E, ca. 627 m, 23.XI.2009, G. Tang and Z.Y. Yao leg. ***Other material examined.*** 1♀, XTBG, bamboo plantation, 21°53.901'N, 101°16.884'E, ca. 568 m, 12.V.2019, Z.G. Chen et al. leg; 1♂ (YHCLU0092) and 1♀ (YHCLU0093), XTBG, 48^th^ km landmark in Menglun Nature Reserve, 21°53.997'N, 101°16.957'E, ca. 593 m, 11.VIII.2011, G. Zheng et al. leg.

##### Diagnosis and description.

See Yu and Li (2019a). Male palp as in Figs 57A, 67A, epigyne as in Figs 77C, 85C, 93C.

##### Distribution.

Known only from Xishuangbanna.

##### Most similar species.

*Clubiona tongi*.

#### Clubiona tongi

Taxon classificationAnimaliaAraneaeClubionidae

Yu & Li, 2019

F284A349-9331-5877-8B14-7797B83C81C9

Figs 57B , 67B , 77D , 85D , 93D

Clubiona tongi Yu & Li, 2019b: 212, figs 9A–E, 10A–H (♂♀).

##### Material examined.

***Types*.** Holotype ♂ (IZCAS Ar 34705), China: Yunnan Province: Xishuangbanna: Mengla County: Menglun Town: XTBG, garbage dump, secondary tropical forest, 21°54.380'N, 101°16.815'E, ca. 627 m, 23.XI.2009, G. Tang and Z.Y. Yao leg; 1♀ (paratype, IZCAS Ar 34707), XTBG, G213 roadside, bamboo plantation, 21°54.622'N, 101°16.955'E, ca. 581 m, 26.XI.2009, G. Tang and Z.Y. Yao leg. ***Other material examined.*** 1♂ (YHCLU0055), XTBG, rubber-tea plantation, 21°55.239'N, 101°15.854'E, ca. 572 m, 28.VII.2018, Z.G. Chen et al. leg; 1♀ (YHCLU0095), XTBG, 48^th^ km landmark in Menglun Nature Reserve, 21°53.997'N, 101°16.957'E, ca. 593 m, 11.VIII.2011, G. Zheng et al. leg.

##### Diagnosis and description.

See Yu and Li (2019b). Male palp as in Figs 57B, 67B, epigyne as in Figs 77D, 85D, 93D.

##### Distribution.

Known only from Xishuangbanna.

##### Most similar species.

*Clubiona theoblicki*.

#### Clubiona zhengi

Taxon classificationAnimaliaAraneaeClubionidae

Yu & Li, 2019

5253CF32-E6C2-5C12-9DFD-C8CE21F72D10

Figs 57E , 67E , 77E , 85E , 93E

Clubiona zhengi Yu & Li, 2019a: 167, figs 11A–E, 12A–H (♂♀).

##### Material examined.

***Types*.** Holotype ♂ (IZCAS Ar 34583), China: Yunnan Province: Xishuangbanna: Mengla County: Menglun Town: XTBG, *Anogeissus acuminata*plantation, 21°54.017N, 101°16.900E, ca. 561 m, 27.IV.2019, Z.G. Chen et al. leg; 1♀ (paratype, IZCAS Ar 34590), XTBG, Lvshilin Forest Park, 21°54.609'N, 101°17.090'E, ca. 643 m, 17.XI.2009, G. Tang and Z.Y. Yao leg. ***Other material examined.*** 1♂ (YHCLU0042) and 1♀ (YHCLU0043), XTBG, *Anogeissus acuminata plantation*, 21°54.033N, 101°16.900E, ca. 606 m, 2.VIII.2018, Z.G. Chen et al. leg.

##### Diagnosis and description.

See Yu and Li (2019a). Male palp as in Figs 57E, 67E, epigyne as in Figs 77E, 85E, 93E.

##### Distribution.

Known only from Xishuangbanna.

##### Most similar species.

*Clubiona jaegeri*.

#### Clubiona japonicola

Taxon classificationAnimaliaAraneaeClubionidae

group

F4DAFE8C-B449-546F-956F-0703732313E1

##### Diagnosis and description.

See Mikhailov (1995).

##### Composition and distribution.

According to Mikhailov (1995), the group includes only two species: *C. japonicola* Bösenberg & Strand, 1906 widespread from the Far East of Russia to the Philippines and Indonesia, and *C. yasudai* Ono, 1991, endemic to Hokkaido, Japan.

##### Comments.

Morphological characters and our unpublished molecular data (pers. obs.) strongly suggest a close relationship between *C. japonicola* and *C. riparia* L. Koch, 1866 (assigned to the *lutescens* group by Mikhailov (1995)).

#### Clubiona japonicola

Taxon classificationAnimaliaAraneaeClubionidae

Bösenberg & Strand, 1906

80B4161E-68C0-57DE-BDF9-1E1CA8CD0EF9

Clubiona japonicola Bösenberg & Strand, 1906: 281, pl. 16, fig. 498 (♂♀); Ono and Hayashi 2009: 535, figs 50–52 (♂♀); Yin et al. 2012: 1101, figs 580a–e, 3-14a–b (♂♀); Wang et al. 2018: 325, fig. 10A–F (♂♀). For full list of taxonomic references see WSC (2021)

##### Material examined.

None.

##### Diagnosis and description.

See Huang and Chen (2012).

##### Distribution.

China (from Jili and south to Yunnan); Russia (Far East); South Korea; Japan; Philippines; Indonesia.

#### Clubiona filicata

Taxon classificationAnimaliaAraneaeClubionidae

group

FFF83172-0DA9-51C3-B384-05D657A21DA8

Tolophus Thorell, 1891: 26 (type species T. submaculatus Thorell, 1891).
Japoniona Mikhailov, 1990: 443 (described as subgenus).
Clubiona : Deeleman-Reinhold 2001: 90 (synonymised Tolophus and Japoniona)
Clubiona japonica group: Deeleman-Reinhold 2001: 90

##### Diagnosis.

See Mikhailov (1990) and Yu et al. (2017a).

##### Description.

See Mikhailov (1995) and Deeleman-Reinhold (2001).

##### Composition and distribution.

A list of the species of the *japonica* group was provided by Yu et al. (2017a). Combined with new taxonomic data in the present paper, we provide an updated list (Table 5).

##### Comments.

*Japoniona* Mikhailov,1990 was described to accommodate the *japonica* group. Later, the subgenus Japoniona was synonymised by Deeleman-Reinhold (2001) with *Clubiona*. A long-forgotten genus, *Tolophus* Thorell, 1891: 26 (type species *T. submaculatus* Thorell, 1891, belonging to the *japonica* group), is currently considered a junior synonym of *Clubiona*. The monophyly of the group is supported by morphological characters and molecular data (pers. obs.). However, the exact placement of the group within *Clubiona* or Clubionidae remains to be investigated using integrative taxonomy. *Tolophus* should perhaps be removed from synonymy with *Clubiona*.

**Table 5.** *Clubiona filicata* group.

	Species name	Known sex	Distribution
1	*C. annuligera* Lessert, 1929	♂♀	Congo, Mozambique
2	*C. abnormis* Dankittipakul, 2008	♂♀	Thailand (Nakhorn Ratchasima), Laos, China (Yunnan)
3	*C. bilobata* Dhali, Roy, Saha & Raychaudhuri, 2016	♀	India (West Bengal)
4	*C. calycina* Wu & Zhang, 2014	♂♀	China (Henan)
5	*C. campylacantha* Dankittipakul, 2008	♂♀	Thailand (Loei)
6	*C. charleneae* Barrion & Litsinger, 1995	♂♀	Philippines (Quezon)
7	*C. circulata* Zhang et Yin, 1998	♂♀	China (Yunnan)
8	*C. coreana* Paik, 1990	♂♀	Russia (south part of the Far East), Korea China (Niaoning)
9	*C. reichlini* Schenkel, 1944	♂♀	China (Zhejiang)
10	*C. digitata* Dankittipakul, 2012	♂♀	Thailand (Loei, Pathum Thani)
11	*C. drassodes* O. P.-Cambridge, 1874	♂♀	India, Bangladesh, China
12	*C. filicata* O. Pickard-Cambridge, 1874	♂♀	Pakistan to Taiwan, south China, south to Thailand
13	*C. filifera* Dankittipakul, 2008	♂♀	Thailand (Nakhorn Ratchasima)
14	*C. filoramula* Zhang & Yin, 1998	♂	China (Yunnan)
15	*C. gallagheri* Barrion & Litsinger, 1995	♀	Indonesia (Java)
16	*C. grucollaris* Yu, Zhang & Chen, 2017	♂♀	China (Hainan, Guizhou, and Yunnan)
17	*C. japonica* L. Koch, 1878	♂♀	Russia (Sakhalin, Kurile Is.), China (Taiwan), Korea, Japan
18	*C. lala* Jäger & Dankittipakul, 2010	♀	Laos (China (Yunnan).
19	*C. melanosticta* Thorell, 1890	♂♀	Thailand (Chiang Mai, Samut Songkram), Indonesia (Sumatra, Krakatau), New Guinea, Laos, China (Yunnan).
20	*C. munda* Thorell, 1887	♀	Myanmar (Kachin State)
21	*C. nigromaculosa* Blackwall, 1877	♂♀	Seychelles, Réunion
22	*C. octoginta* Dankittipakul, 2008	♂♀	Thailand (Ubon Ratchathani)
23	*C. picturata* Deeleman-Reinhold, 2001	♂♀	Indonesia (Bali)
24	*C. pila* Dhali, Roy, Saha & Raychaudhuri, 2016	♀	India (West Bengal)
25	*C. pupula* Thorell, 1897	♂♀	Myanmar (Kachin State)
26	*C. scandens* Deeleman-Reinhold, 2001	♂♀	Malaysia (Borneo)
27	*C. submaculata* (Thorell, 1891)	♂♀	India (Nicobar Is.)
28	*C. suthepica* Dankittipakul, 2008	♂♀	Thailand (Chiang Mai), China (Yunnan)
29	*C. vigil* Karsch, 1879	♂♀	Kuril Isles, Korea, Japan, China (Hebei, Hubei)
30	*C. yueya* Yu & Li, 2019	♂♀	China (Yunnan)
31	*C. zhanggureni* Yu & Li, 2019	♂	China (Yunnan)
32	*C. zhangyongjingi* Li & Blick, 2019	♀	China (Yunnan)
33	*C. banna* Yu & Li, sp. nov.	♂♀	China (Yunnan)

#### Key to *C. filicata* group species occurring in Xishuangbanna (males)

*C. filoramula* is excluded due to lack of specimens.

**Table:** 

1	Conductor absent (Figs 58A, B, 68A, B)	**2**
–	Conductor large and beak-shaped, transversely aligned anteriorly (Figs 58C, 59A–D, 60A–D, 68C, 69A–D, 70A–D)	**3**
2	Embolus short, no longer than tegulum width, sickle-shaped; tegular apophysis absent (Figs 58A, 68A)	***C. reichlini***
–	Conductor distinctly longer than tegulum width, filiform and slender, tapering gradually in ca. two loops; tegular apophysis small and digitiform (Figs 58B, 68B)	***C. filicata***
3	Tegular apophysis distinct, heavily sclerotised (Figs 58C, 60A–D, 68C, 70A–D)	**4**
–	Tegular apophysis absent, or present but reduced, partly membranous, indistinct (Figs 59A–D, 69A–D)	**8**
4	Tegulum obscuring sperm duct in ventral view (Fig. 60C, D)	**5**
–	Sperm duct sinuate and distinct (Figs 58C, 60A, B)	**6**
5	Tegular apophysis large, crescent-shaped; embolus spiniform (Fig. 60D)	***C. yueya***
–	Tegular apophysis small, beak-shaped; embolus filiform (Fig. 60C)	***C. abnormis***
6	Embolar apex coiled (Fig. 42D); tegular apophysis large and boomerang shaped in ventral view (Fig. 60B)	***C. lala***
–	Embolar tip not coiled, tegular apophysis not as above	**7**
7	Tegular apophysis with tubercle-shaped base and rostrate tip; sperm duct sinuate, forming a loop along tegular margin (Fig. 58C)	***C. banna* sp. nov.**
–	Tegular apophysis petal-shaped, sperm duct U- or S-shaped (Fig. 60A)	***C. grucollaris***
8	Retrolateral tibial apophysis hook-shaped, strongly excavated on the ventral side; cymbium with basolateral extension (Fig. 69D)	***C. suthepica***
–	Retrolateral tibial apophysis not so; cymbium without basolateral extension	**9**
9	Conductor strongly sclerotised, horn-shaped, pointing retrolatero-distally (Fig. 59C); retrolateral tibial apophysis bent, thumb-like (Fig. 69C)	***C. circulata***
–	Conductor membranous except for the beak-shaped tip (Fig. 59A, B), retrolateral tibial apophysis triangular (Fig. 69A, B)	**10**
10	Embolus spiniform (Fig. 59B)	***C. zhanggureni***
–	Embolus filiform (Fig. 59A)	***C. melanosticta***

#### Key to females of *C. filicata* group species occurring in Xishuangbanna (females)

(the females of *C. abnormis*, *C. filicata*, *C. filoramula*, *C. zhanggureni*, and *C. zhangyongjingi* are excluded due to lack of specimens)

**Table:** 

1	Atrium small, width no more than 1/2 epigyne width (Figs 78E, 86E)	***C. banna* sp. nov.**
–	Atrium broad, width almost equal to epigyne width (Figs 78F, 79A–F, 86F, 87A–F)	**2**
2	Posterior margin of atrium delimited	**3**
–	Posterior margin of atrium not delimited	**4**
3	Atrium anterior margin not delimited (Figs 78F, 86F); spermathecae consisting of base, stalk and head (Fig. 94F)	***C. melanosticta***
–	Atrium anterior margin distinct and long, ˄-shaped (Figs 79A, 87A); spermathecae distinctly smaller than bursae, lobe-shaped, not subdivided (Fig. 95A)	***C. circulata***
4	Atrium anterior margin heavily sclerotised, M-shaped, lateral atrial margins not rebordered (Figs 79E, 87E)	***C. suthepica***
–	Atrium anterior margin not sclerotised; lateral atrial margins rebordered	**5**
5	Atrium anterior margin medially concave (Figs 79C, 87C, F)	**6**
–	Atrium anterior margin medially not concave (Figs 79B, 87B, D)	**7**
6	Spermathecae larger than bursae, consisting of papilliform base, tubular stalk and ovoid head, ascending spirally (Fig. 95C)	***C. grucollaris***
–	Spermathecae distinctly smaller than bursae, consisting of bean-shaped proximal part and digitiform distal part (Fig. 95F)	***C. yueya***
7	Atrium more rectangular (Fig. 87D); spermathecae located in the centre of vulva, well separated from atrium anterior margin; spermathecae separated by ca. one diameter, consisting of base and head (Fig. 95D)	***C. lala***
–	Atrium more cambered (Fig. 79B, 87B); spermathecae located at anterior part of vulva, close to atrium anterior margin; spermathecae close together, consisting of base, stalk and head (Fig. 95B)	***C. reichlini***

#### Clubiona abnormis

Taxon classificationAnimaliaAraneaeClubionidae

Dankittipakul, 2008

8E4A3C26-C986-5150-8900-FB422361C48F

**Figure 30.**
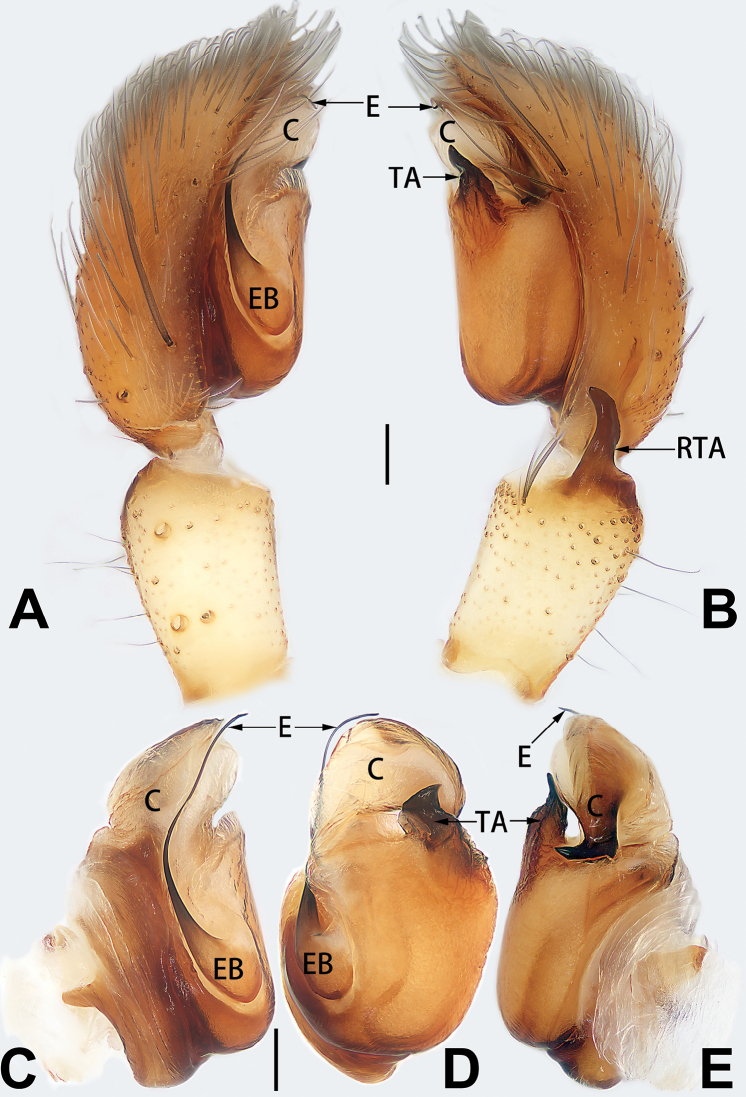
Male palp of *Clubiona abnormis***A** prolateral view **B** retrolateral view **C** bulb, prolateral view **D** bulb, ventral view **E** bulb, retrolateral view. Abbreviations: C = conductor; E = embolus; EB = embolar base; RTA = retrolateral tibial apophysis; TA = tegular hump. Scale bars: 0.1 mm (equal for **A, B**, equal for **C–E**).

**Figure 31.**
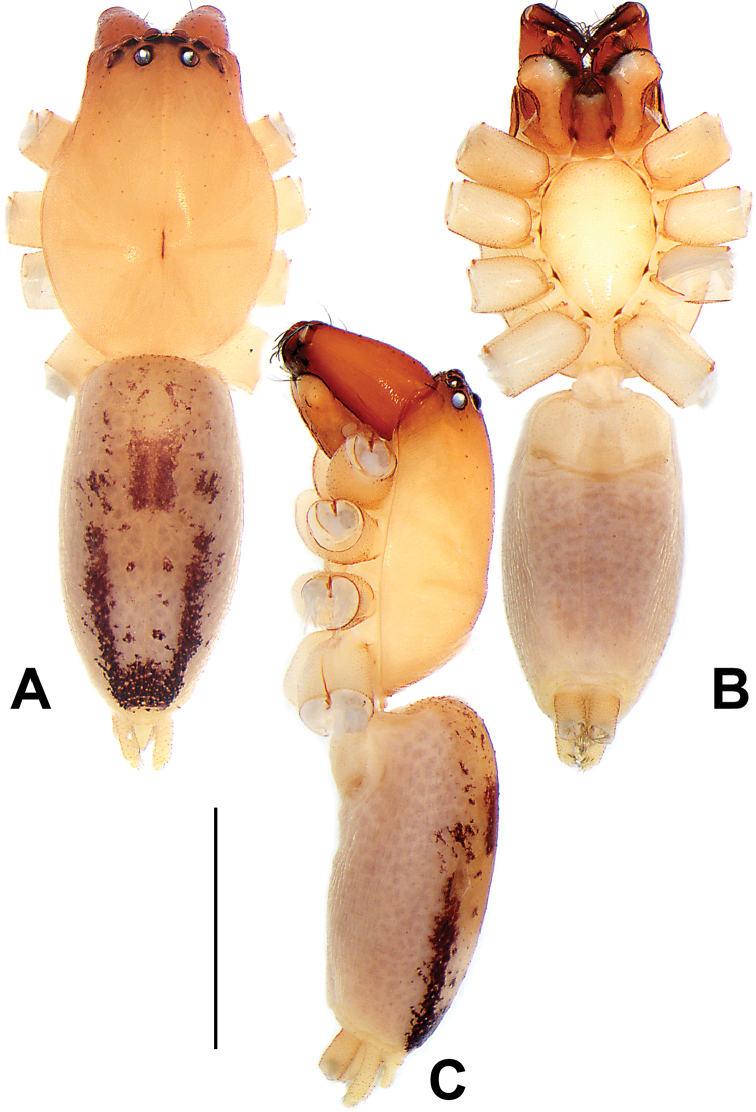
Male habitus of *Clubiona abnormis***A** dorsal view **B** ventral view **C** lateral view. Scale bar: 1 mm (equal for **A–C**).

Figs 30 , 31 , 60C , 70C

Clubiona abnormis Dankittipakul, in Dankittipakul and Singtripop 2008a: 44, figs 28, 29, 61–68 (♂♀); Jäger and Dankittipakul 2010: 32, figs 27, 31–33 (♀).

##### Material examined.

1♂ (YHCLU0113), China: Yunnan Province: Xishuangbanna: Mengla County: Menglun Town: XTBG, 48^th^ km landmark in Menglun Nature Reserve, 21°58.704'N, 101°19.748'E, ca. 1088 m, 12.VIII.2011, G. Zheng et al. leg.

##### Diagnosis and description.

See Dankittipakul and Singtripop (2008a). Male palp as in Figs 30, 60C, 70C, habitus as in Fig. 31.

##### Distribution.

Thailand (Nakhorn Ratchasima), Laos, China (Yunnan).

##### Most similar species.

*Clubiona vigil*.

#### Clubiona banna

Taxon classificationAnimaliaAraneaeClubionidae

Yu & Li sp. nov.

A12F1FF9-D9DE-5B2D-B77C-EF67ED08CEB3

http://zoobank.org/A821EEFC-C147-4DA6-AE4B-866DD621B2B8

**Figure 32.**
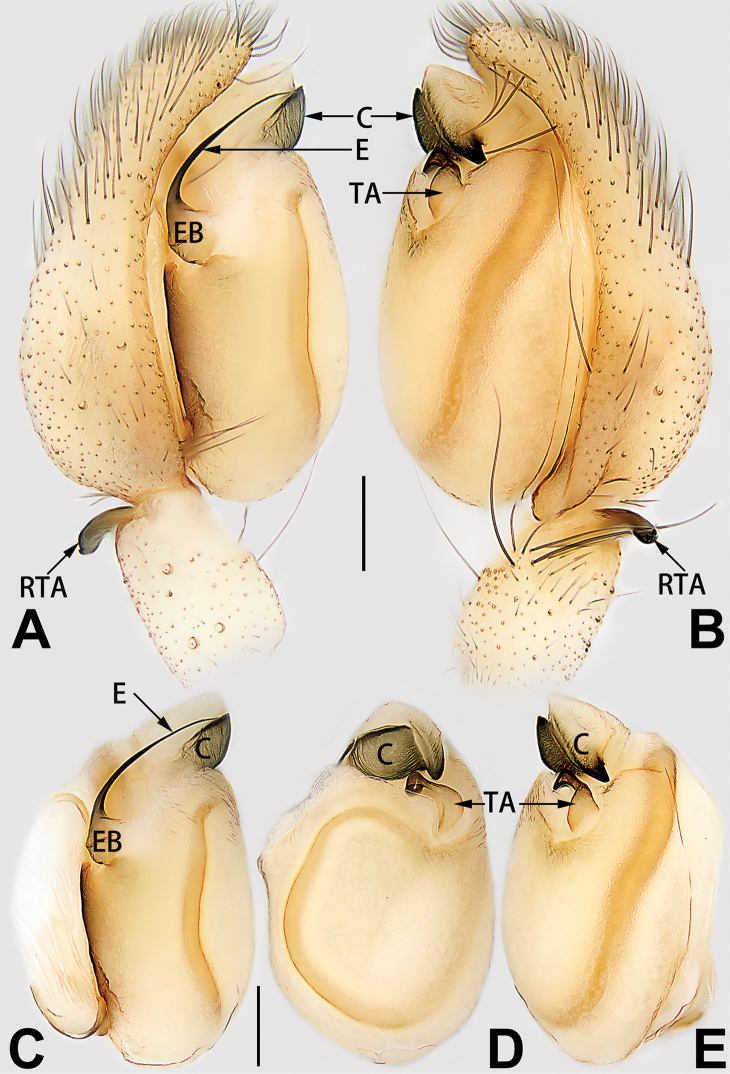
Male palp of the holotype of *Clubiona banna* sp. nov. **A** prolateral view **B** retrolateral view **C** bulb, prolateral view **D** bulb, ventral view **E** bulb, retrolateral view. Abbreviations: C = conductor; E = embolus; EB = embolar base; RTA = retrolateral tibial apophysis; TA = tegular hump. Scale bars: 0.2 mm (equal for **A, B**, equal for **C–E**).

**Figure 33.**
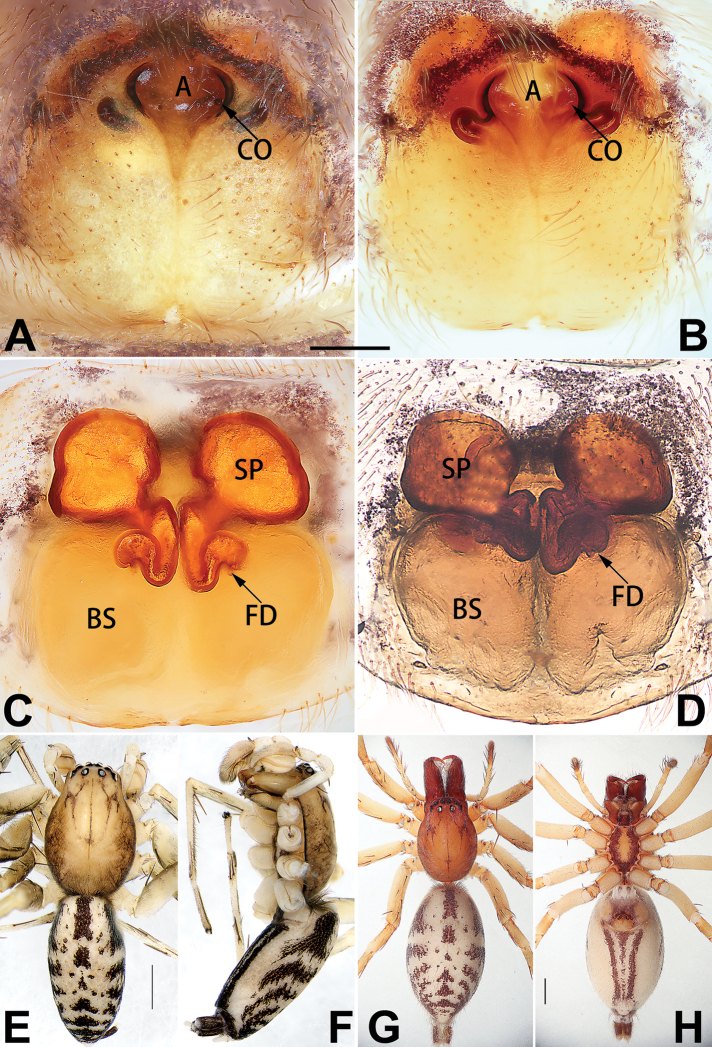
*Clubiona banna* sp. nov., female paratype and male holotype, epigyne (**A–D**), male habitus (**E, F**) and female habitus (**G, H**) **A** intact, ventral view **B** cleared, ventral view **C** cleared, dorsal view **D** cleared, dorsal view **E** dorsal view **F** Lateral view **G** dorsal view **H** ventral view. Abbreviations: A = atrium; BS = bursa; CO = copulatory opening; FD = fertilisation duct; SP = spermatheca. Scale bars: 0.2 mm (equal for **A–D**); 1 mm (equal for **E, F**, equal for **G, H**).

Figs 32 , 33 , 58C , 68C , 78E , 86E , 94E

##### Holotype.

♂ (IZCAS-Ar 34760), China: Yunnan Province: Xishuangbanna: Mengla County: Xiaolongha Village, 21°24.798'N, 101°37.880'E, ca. 693 m, 28.VI.2012, Q.Y. Zhao and C.X. Gao leg; paratype: 1♀ (IZCAS-Ar 34761, YHCLU0139), Menglun Town, XTBG, primary tropical seasonal forest, 21°57.445'N, 101°12.997'E, ca. 774 m, 30.VIII.2007, G. Zheng leg. ***Other material examined.*** 1♂ (YHCLU0104), same data as holotype.

##### Etymology.

The species name is derived from the name of the type locality; noun in apposition.

##### Diagnosis.

Males of *C. banna* sp. nov. can be easily distinguished from those of all others in the species group by the tegular apophysis, having a tubercle-shaped base and a rostrate tip (Fig. 32B, D, E). Females of *C. banna* sp. nov. are similar to those of *C. vigil* (Kim and Lee 2014: 38, fig. 26B) by the relatively small atrium (vs. atria usually broad in almost all members of the *C. filicata* group, including *C. circulata*, *C. reichlini*, *C. grucollaris* and *C. lala*; Figs 35A, B, 79A, 87A, 37A, B, 79B, 87B, 41A, B, 79C, 87C, 43A, B, 79D, 87D) but can be recognised by the atrium being anteriorly elliptic and posteriorly shaped like an inverted triangle (Figs 33A, B, 78E, 86E) (vs. cordiform in *C. vigil*).

##### Description.

**Male.** Holotype (Fig. 33E, F): Total length 6.53; carapace 2.84 long, 2.02 wide; opisthosoma 3.69 long, 1.73 wide. Carapace brown, marginally dark, a pair of Y-shaped black markings starting from behind PME and PLE, almost reaching reddish fovea; pars cephalica distinctly narrowed; cervical groove and radial grooves indistinct; tegument smooth, marginally clothed with short, dense setae. Eyes: in dorsal view, both anterior and posterior eye rows recurved, PER slightly wider than AER. Eye sizes and interdistances: AME 0.11, ALE 0.15, PME 0.15, PLE 0.14, AME–AME 0.14, AME–ALE 0.07, PME–PME 0.26, PME–PLE 0.17, MOQL 0.40, MOQA 0.36, MOQP 0.53. Chelicerae brown, with red fangs, with three promarginal and two retromarginal teeth. Sternum centrally pale brown, marginally dark, 1.52 long, 1.02 wide. Labium and endites coloured as chelicerae. Legs light brown, without distinct markings. Leg measurements: I 9.08 (2.61, 3.60, 1.85, 1.02), II 10.55 (2.87, 3.96, 2.59, 1.13), III 8.25 (2.44, 2.65, 2.46, 0.71), IV 10.98 (3.02, 3.50, 3.43, 1.02). Abdomen: dorsum with broken dark median band in anterior half, posteriorly with 5 chevrons; venter with three dark longitudinal lines.

Palp (Figs 32A–E, 58C, 68C). Tibia short, ca. 1/3 of cymbium length, with single retrolateral apophysis; hammer-like or clavate RTA small, slightly curved, and bluntly pointed. Tegulum more or less spherical, 2.1 × longer than wide, proapically and apically membranous, slightly excavated prolatero-apically to accommodate embolus; sperm duct sinuate, forming a loop along tegular margin. Embolus filiform, arising at approximately the 9–10 o’clock position, terminating at ca. 12 o’clock position, tip hidden behind conductor. Conductor large, beak-shaped, transversely aligned at the apical portion of the bulb, basal part partly membranous, terminal part heavily sclerotised, directed retrolaterad then abruptly bending distad. Tegular apophysis with tubercle-shaped base and rostrate tip, located at distal-retrolateral position of tegulum (ca. 1 o’clock position of tegulum).

Female. Paratype (Fig. 33G, H). Total length 8.05; carapace 3.40 long, 2.19 wide; opisthosoma 4.65 long, 2.95 wide. Eye sizes and interdistances: AME 0.15, ALE 0.20, PME 0.18, PLE 0.16, AME–AME 0.11, AME–ALE 0.11, PME–PME 0.31, PME–PLE 0.20, MOQL 0.49, MOQA 0.45, MOQP 0.66. Chelicerae with three promarginal and two retromarginal teeth. Sternum 1.88 long, 1.14 wide. Leg measurements: I 8.71 (2.42, 3.50, 1.78, 1.01), II 9.56 (2.67, 3.71, 2.10, 1.07), III 7.78 (2.23, 2.75, 2.08, 0.72), IV 10.60 (2.86, 3.47, 3.21, 0.97). Colouration lighter than in male. Other characters as in male.

Epigyne (Figs 33A–D, 78E, 86E, 94E). Epigynal plate nearly square, margin not rebordered, spermathecae and bursae indistinctly visible through integument. Atrium small, with delimited margin, ca. 1/2 epigyne length and 1/3 epigyne width, anteriorly elliptic, posteriorly triangular. Copulatory openings located at lateral atrial borders. Copulatory ducts short, running sideways, then retracing posteriorly to bursae. Spermathecae with subglobular proximal part and tubular distal part; the two proximal parts separated by 0.5 diameters, and the two distal parts close together. Fertilisation ducts short and curved, acicular, located on distal surface of spermathecae. Bursae reniform, ca. 1.5 × longer than wide, close together, surface membranous and smooth.

##### Distribution.

Known only from the type locality, Xishuangbanna, Yunnan, China.

#### Clubiona circulata

Taxon classificationAnimaliaAraneaeClubionidae

Zhang & Yin, 1998

242DBCFF-81F9-5557-99E0-EE9E85B9A403

**Figure 34.**
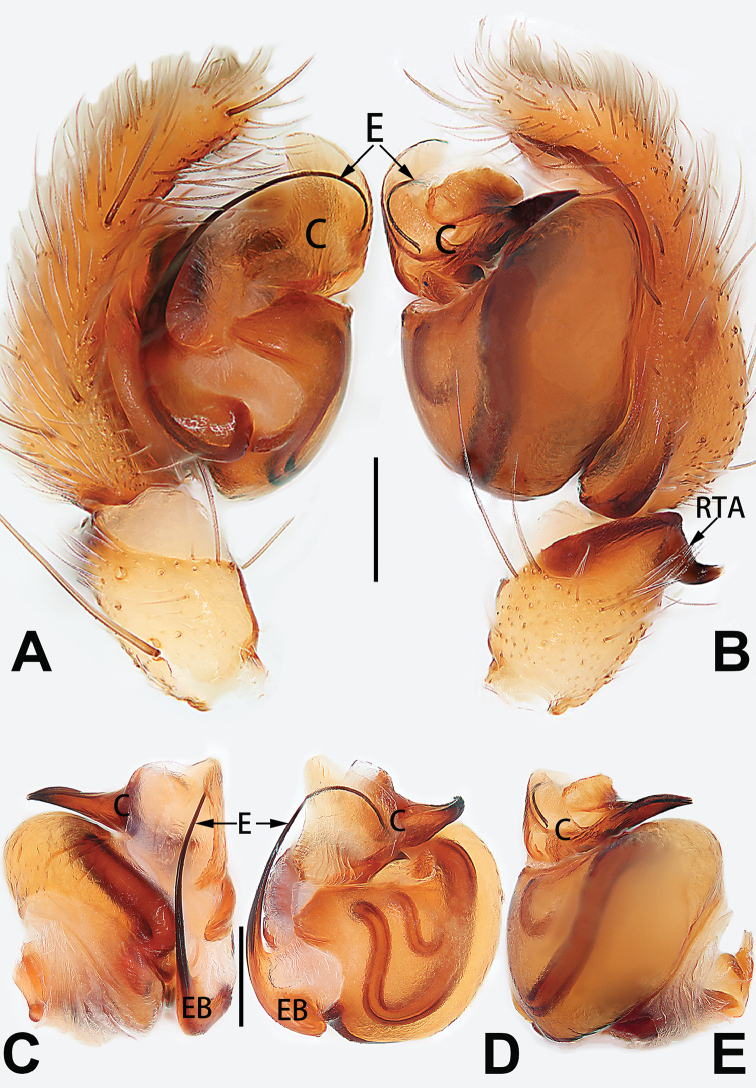
Male palp of *Clubiona circulata***A** prolateral view **B** retrolateral view **C** bulb, prolateral view **D** bulb, ventral view **E** bulb, retrolateral view. Abbreviations: C = conductor; E = embolus; EB = embolar base; RTA = retrolateral tibial apophysis. Scale bars: 0.2 mm (equal for **A, B**, equal for **C–E**).

**Figure 35.**
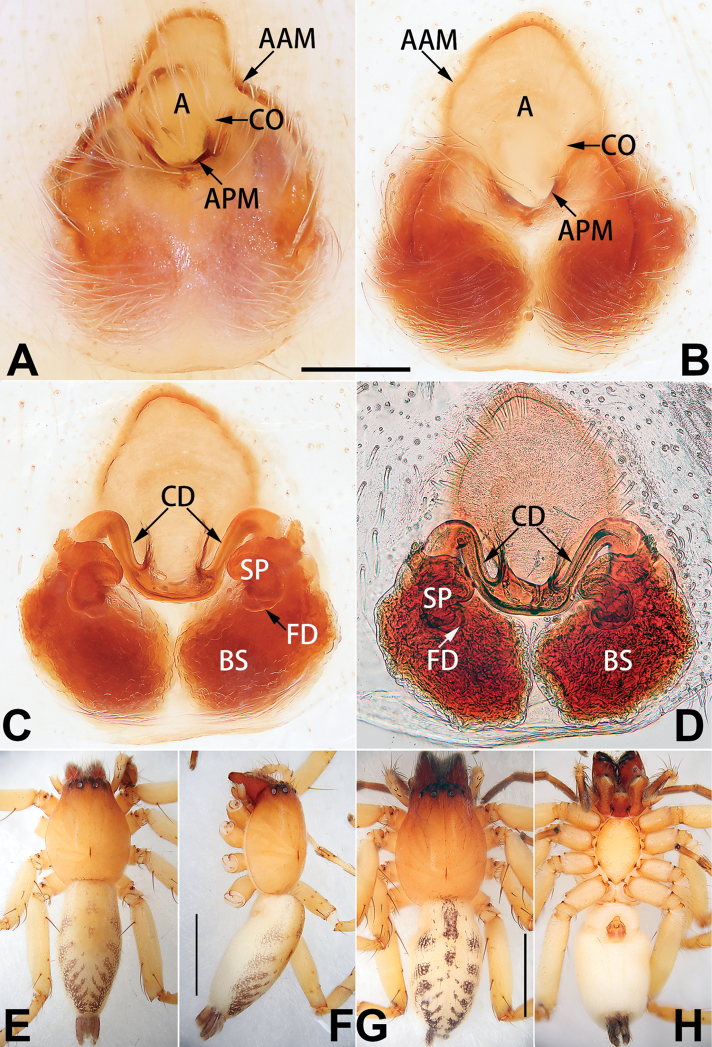
*Clubiona circulata*, epigyne (**A–D**), male habitus (**E, F**) and female habitus (**G, H**) **A** intact, ventral view **B** cleared, ventral view **C** cleared, dorsal view **D** cleared, dorsal view **E** dorsal view **F** lateral view **G** dorsal view **H** ventral view. Abbreviations: A = atrium; AAM = atrial anterior margin; APM = atrial posterior margin; BS = bursa; CD = copulatory duct; CO = copulatory opening; FD = fertilisation duct; SP = spermatheca. Scale bars: 0.2 mm (equal for **A–D**); 2 mm (equal for **E, F**, equal for **G, H**).

Figs 34 , 35 , 59C , 69C , 79A , 87A , 95A

Clubiona circulata Zhang & Yin, 1998: 9, figs 1–2 (♀ only, ♂ mismatched).
Clubiona vukomi Jäger & Dankittipakul, 2010: 27, figs 13–21 (♂). Syn. nov.

##### Material examined.

1♂, China: Yunnan Province: Xishuangbanna: Mengla County: Menglun Town: XTBG, rubber plantation, 21°54.498'N, 101°16.326'E, ca. 586 m, 17.VII.2007, G. Zheng leg; 2♀, XTBG, rubber plantation, 21°54.350'N, 101°16.461'E, ca. 614 m, 11.VIII.2007, G. Zheng leg; 1♂ (YHCLU0108), Jinghong City: Menghai County: Manda Village, secondary forest, 22°1.702'N, 100°23.697'E, ca. 1188 m, 28.VII.2012, Q.Y. Zhao and Z.G. Chen leg; 1♀ (YHCLU0156), XTBG, seedling culture base, 21°54.007'N, 101°16.395'E, ca. 550 m, 10.V.2019, H. Yu et al. leg; 7♂ 6♀, XTBG, *Flocculus banyan*plantation, 22°4.598'N, 100°37.013'E, ca. 1137 m, 21.VIII.2011, Q.Y. Zhao and C.X. Gao leg;

##### Diagnosis.

The female of *C. circulata* is easily differentiated from other members of the group by having an epigynal atrium with ˄-shaped anterior margin and a V-shaped posterior margin (Figs 35A, B, 79A, 87A) (vs. anterior margin not ˄-shaped, atrial posterior margin absent in almost all others, including *C. reichlini*, *C. grucollaris*, and *C. lala*; Figs 37A, B, 79B, 87B, 41A, B, 79C, 87C, 43A, B, 79D, 87D). Males of *C. cicrulata* differ from all other group members by the large and strongly sclerotised, horn-shaped conductor, pointing retrolatero-distally (Fig. 34B–E) (vs. conductors of almost all other congroupers, such as *C. grucollaris* and *C. suthepica*, are beak-shaped, pointing retrolatero-proximally; Figs 40B–E, 60A, 70A, 46B–E, 59D, 69D).

##### Description.

**Male.** See Jäger and Dankittipakul (2010). Male palp as in Figs 34, 59C, 69C, habitus as in Fig. 35E, F.

**Female.** (Fig. 35G, H): Total length 6.00; carapace 2.67 long, 2.01 wide; opisthosoma 3.33 long, 1.59 wide. Carapace reddish brown, anteriorly darker, with a pair of indistinct, Y-shaped purplish patterns starting from behind PER, almost reaching indistinct cervical groove, fovea dark reddish; cephalic region raised, radial grooves indistinct; tegument smooth, with erect, thin, dark setae on the front ridge. Eyes: AER slightly recurved, PER slightly wider than AER, almost straight in dorsal view. Eye sizes and interdistances: AME 0.10, ALE 0.14, PME 0.13, PLE 0.09, AME–AME 0.08, AME–ALE 0.04, PME–PME 0.24, PME–PLE 0.16, MOQL 0.40, MOQA 0.36, MOQP 0.51. Chelicerae robust and dark brownish red, promargin with three teeth, retromargin with two subequal teeth. Sternum pale yellow, 1.43 long, 1.01 wide. Labium and endites orange. Legs light brown, without distinct markings. Leg measurements: I 7.40 (2.11, 3.00, 1.46, 0.83), II 7.88 (2.26, 3.14, 1.62, 0.86), III 6.30 (1.69, 2.23, 1.88, 0.53), IV 9.58 (2.55, 3.34, 2.96, 0.74). Abdomen elongate, oval, with a thick tuft of setae anteriorly; dorsum with broken purplish longitudinal band medially, starting anteriorly for half the length; 6–7 pair of broken lateral bands fused posteriorly. Venter uniformly creamy white, without markings.

Epigyne (Figs 35A–D, 79A, 87A, 95A). Epigynal plate ca. 1.1 × longer than wide, through which large spermathecae and bursae are clearly visible. Atrium distinctly large, with rebordered margin, more than 2/3 epigyne length and 4/5 epigyne width; atrial anterior margin long and shaped like ˄, atrial posterior margin relatively short and nearly V- or U-shaped. Copulatory openings near the middle part of the epigyne, close to the anterolateral borders of the APM. Copulatory ducts distinct, almost equal to bursae length, extending obliquely toward the anterior, between the spermathecae, before abruptly bending posteriorly, finally entering the connecting piece between the spermathecae and bursae. Spermathecae relatively small, lobe-shaped, separated by two diameters. Fertilisation ducts acicular, membranous, located terminally on spermathecae. Bursae oblong, 1.4 × longer than wide, close together, bursal surface hyaline, wrinkled and ribbed, inside pigmented and sclerotised.

##### Remarks.

*Clubiona circulata* was described based on five females and two males from Xishuangbanna. The female was chosen as the holotype. However, we have found that the male and female of *C. circulata* were mismatched. While examining spider specimens collected from Xishuangbanna, we found pairs of *filicata* group specimens in the same location that have a similar habitus, markings, leg spination, and other characters (Fig. 35E–H). Therefore, it is very likely they are the opposite sexes of the same species. The females were identified as *C. circulata* based on comparison with the original illustrations of Zhang and Yin (1998). However, the males were identified to be *C. vukomi*, which was established as a new species by Jäger and Dankittipakul (2010) from Luang Nam Tha Province in Laos. Our molecular analysis of COI indicates that the female of *C. circulata* and the male of *C. vukomi* are conspecific, and therefore *C. vukomi* should be considered a junior synonym of *C. circulata*.

##### Distribution.

Thailand (Chiang Mai Province and district, Chai Ya Phum Province), Laos (Luang Nam Tha Province), China (Yunnan).

#### Clubiona reichlini

Taxon classificationAnimaliaAraneaeClubionidae

Schenkel, 1944, species resurrected

D3529FE8-3CCB-5C93-B929-D55897EFECF5

**Figure 36.**
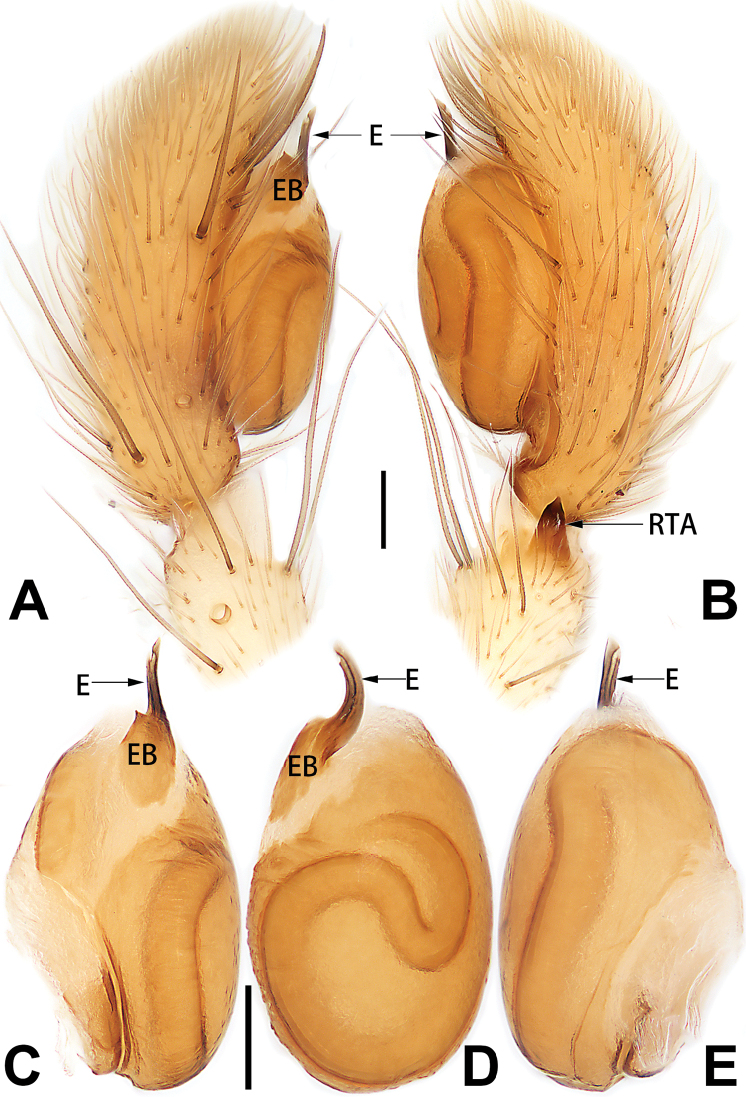
Male palp of *Clubiona reichlini***A** prolateral view **B** retrolateral view **C** bulb, prolateral view **D** bulb, ventral view **E** bulb, retrolateral view. Abbreviations: E = embolus; EB = embolar base; RTA = retrolateral tibial apophysis. Scale bars: 0.2 mm (equal for **A, B**, equal for **C–E**).

**Figure 37.**
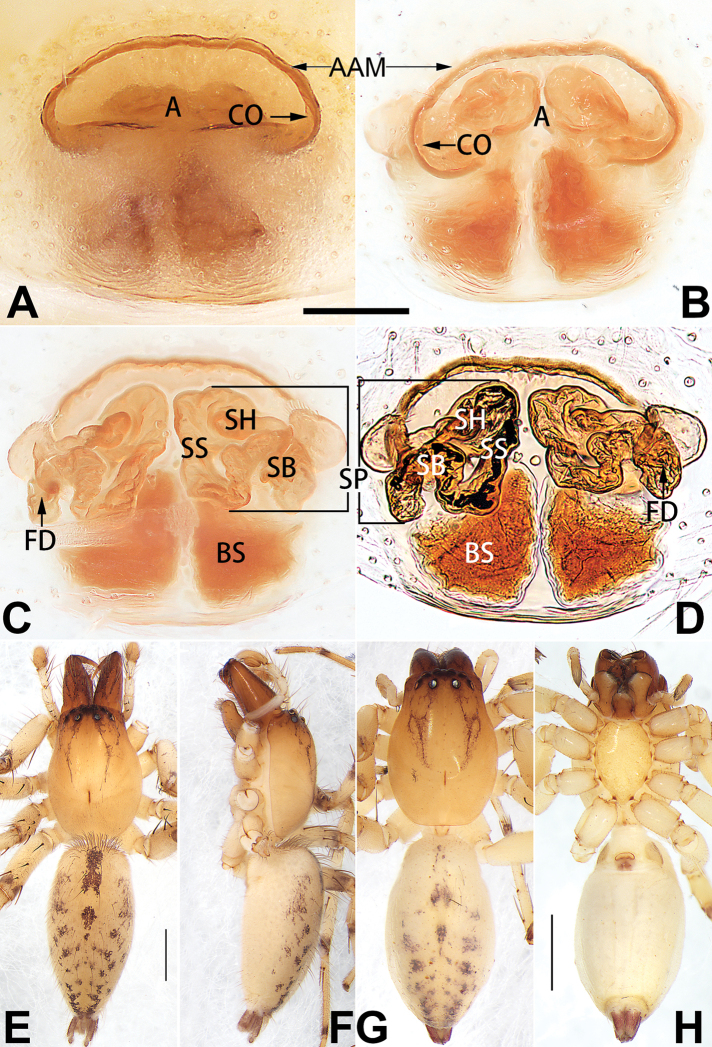
*Clubiona reichlini*, epigyne (**A–D**), male habitus (**E, F**) and female habitus (**G, H**) **A** intact, ventral view **B** cleared, ventral view **C** cleared, dorsal view **D** cleared, dorsal view **E** dorsal view **F** lateral view **G** dorsal view **H** ventral view. Abbreviations: A = atrium; AAM = atrial anterior margin; BS = bursa; CO = copulatory opening; FD = fertilisation duct; SB = spermathecal base; SH = spermathecal head; SP = spermatheca; SS = spermathecal stalk. Scale bars: 0.1 mm (equal for **A–D**); 1 mm (equal for **E, F**, equal for **G, H**).

Figs 36 , 37 , 58A , 68A , 79B , 87B , 95B

Clubiona reichlini Schenkel, 1944: 203, fig. 14 (♂♀).

##### Material examined.

1♂ (YHCLU0263), 1♀(YHCLU0264), China: Yunnan Province: Xishuangbanna: Mengla County: Xiaolongha Village, 22°5.017'N, 100°22.084'E, ca. 1118 m, 24.VII.2012, Q.Y. Zhao and Z.G. Chen leg.

##### Diagnosis and description.

See Schenkel (1944). Male palp as in Figs 36, 58A, 68A, epigyne as in Figs 37A–D, 79B, 87B, 95B, habitus as in Fig. 37E–H.

##### Remarks.

*Clubiona reichlini* was considered a senior synonym of *C. deletrix* O. Pickard-Cambridge, 1885 by Zhang (1991), but this is not accepted here based on comments by Y. Marusik (pers. comm.). As a result, *C. reichlini* is removed from synonymy with *C. deletrix*.

##### Distribution.

China (Zhejiang, Yunan).

##### Most similar species.

*Clubiona campylacantha*.

#### Clubiona filicata

Taxon classificationAnimaliaAraneaeClubionidae

O. Pickard-Cambridge, 1874

A4EB9FD0-D60E-5F68-9CF6-3FB3898BB88F

**Figure 38.**
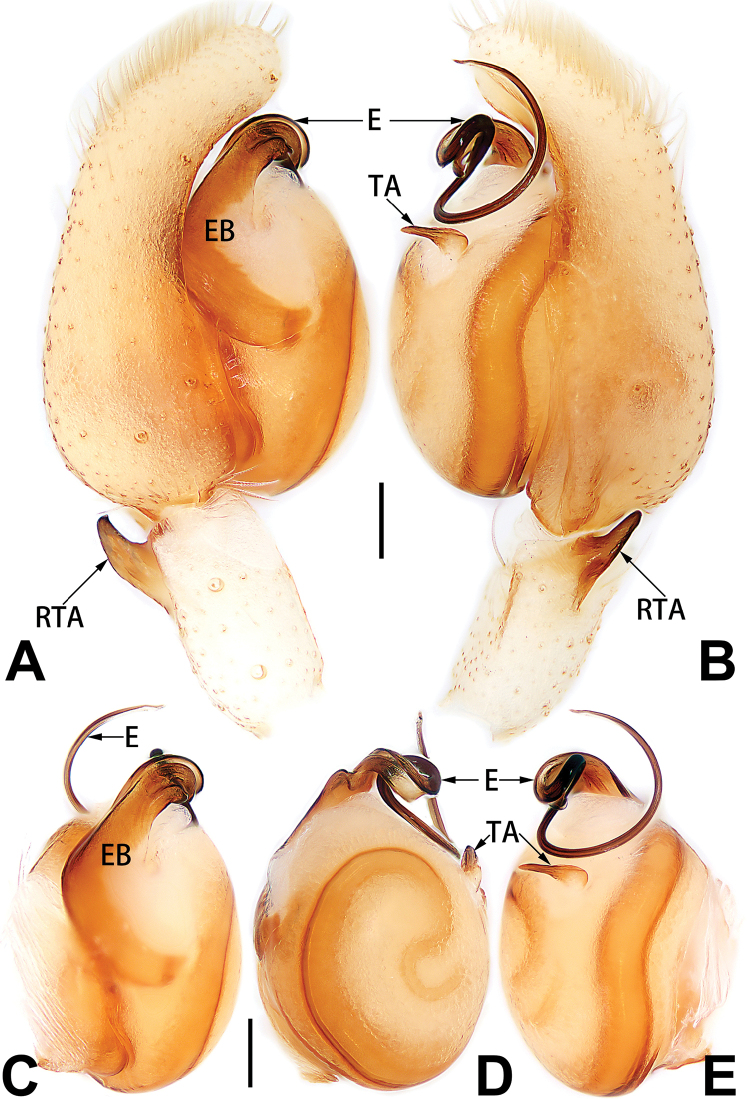
Male palp of *Clubiona filicata***A** prolateral view **B** retrolateral view **C** bulb, prolateral view **D** bulb, ventral view **E** bulb, retrolateral view. Abbreviations: E = embolus; EB = embolar base; RTA = retrolateral tibial apophysis; TA = tegular hump. Scale bars: 0.1 mm (equal for **A, B**, equal for **C–E**).

**Figure 39.**
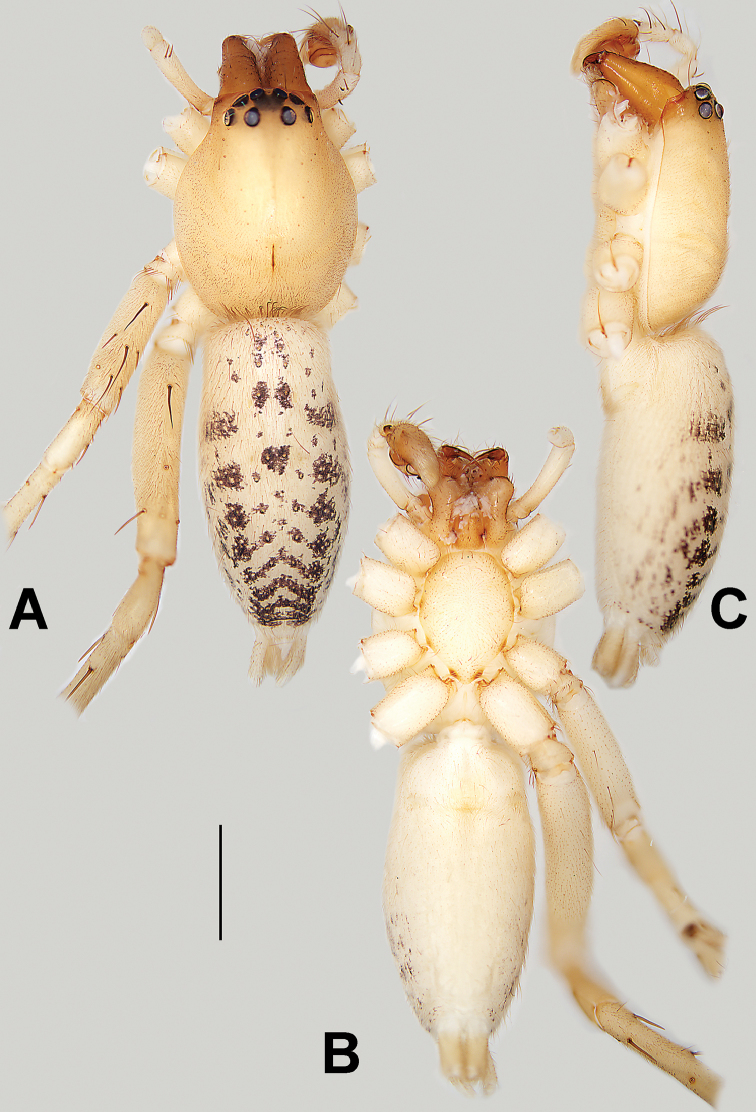
Male habitus of *Clubiona filicata***A** dorsal view **B** ventral view **C** lateral view. Scale bar: 1 mm (equal for **A–C**).

Figs 38 , 39 , 58B , 68B

Clubiona filicata O. Pickard-Cambridge, 1874: 413, pl. 52, fig. 35 (♂♀); Dankittipakul et al. 2012: 59, figs 25–31 (♂♀); Caleb 2020: 15719, figs 4A–F, 25G (♀).
Clubiona distincta Thorell, 1887: 48 (♀).
Clubiona swatowensis Strand, 1907: 562 (♀); Strand 1909: 39, fig. 24 (♀).
Clubiona pashabhaii Patel & Patel, 1973: 2, fig. 1a–c (♀).
Clubiona foliata Keswani & Vankhede, 2014: 36, figs 1–13 (♂♀). For full list of taxonomic references see WSC (2021).

##### Material examined.

1♂ (YHCLU0107), China: Yunnan Province: Xishuangbanna: Mengla County: Menglun Town: XTBG, flower garden, 21°55.919'N, 101°14.994'E, ca. 545 m, 14.V.2019, C. Wang and H. Yu leg.

##### Diagnosis and description.

**Male.** See Dankittipakul and Singtripop (2008a). Male palp as in Figs 38, 58B, 68B, habitus as in Fig. 39.

**Female.** See Keswani and Vankhede (2014).

##### Remarks.

*Clubiona deletrix* was described based on both sexes in the original publication. Y. Marusik studied the types of *C. deletrix* and *C. filicata* and the original drawings. He found that the male and female of *C. deletrix* were not conspecific in the original description, and the male type is *C. filicata* (pers. comm.).

##### Distribution.

From Pakistan to Taiwan, south to Thailand, China (Fujian, Hunan, Guangdong, Guangxi, Taiwan, Yunnan).

##### Most similar species.

*Clubiona filoramula*.

#### Clubiona filoramula

Taxon classificationAnimaliaAraneaeClubionidae

Zhang & Yin, 1998

081BEA46-E6E3-55FC-9B3A-E32E3D4E5E3C

Clubiona filoramula Zhang & Yin, 1998: 12, figs 9–11 (♂).

##### Material examined.

None.

##### Diagnosis and description.

See Zhang and Yin (1998).

##### Distribution.

China (Yunnan).

##### Most similar species.

*Clubiona filicata*.

#### Clubiona grucollaris

Taxon classificationAnimaliaAraneaeClubionidae

Yu, Zhang & Chen, 2017

24630D87-016F-59A2-A4DA-D4BFEFCE6D33

**Figure 40.**
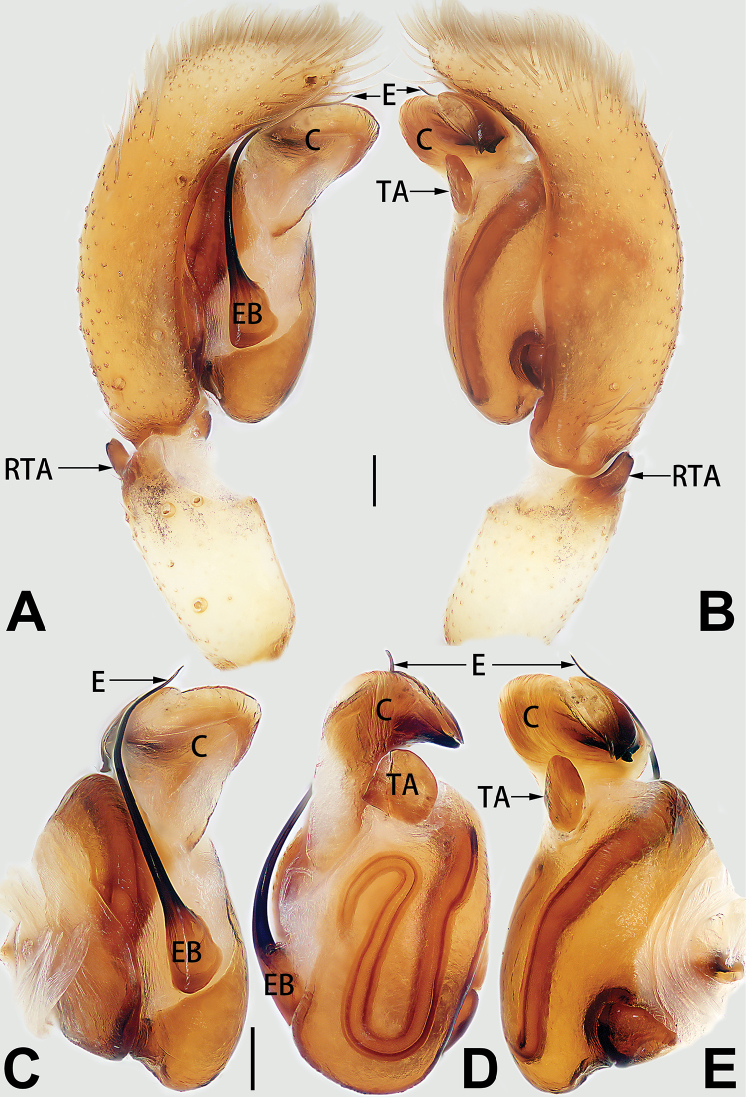
Male palp of *Clubiona grucollaris***A** prolateral view **B** retrolateral view **C** bulb, prolateral view **D** bulb, ventral view **E** bulb, retrolateral view. Abbreviations: C = conductor; E = embolus; EB = embolar base; RTA = retrolateral tibial apophysis; TA = tegular hump. Scale bars: 0.1 mm (equal for **A, B**, equal for **C–E**).

**Figure 41.**
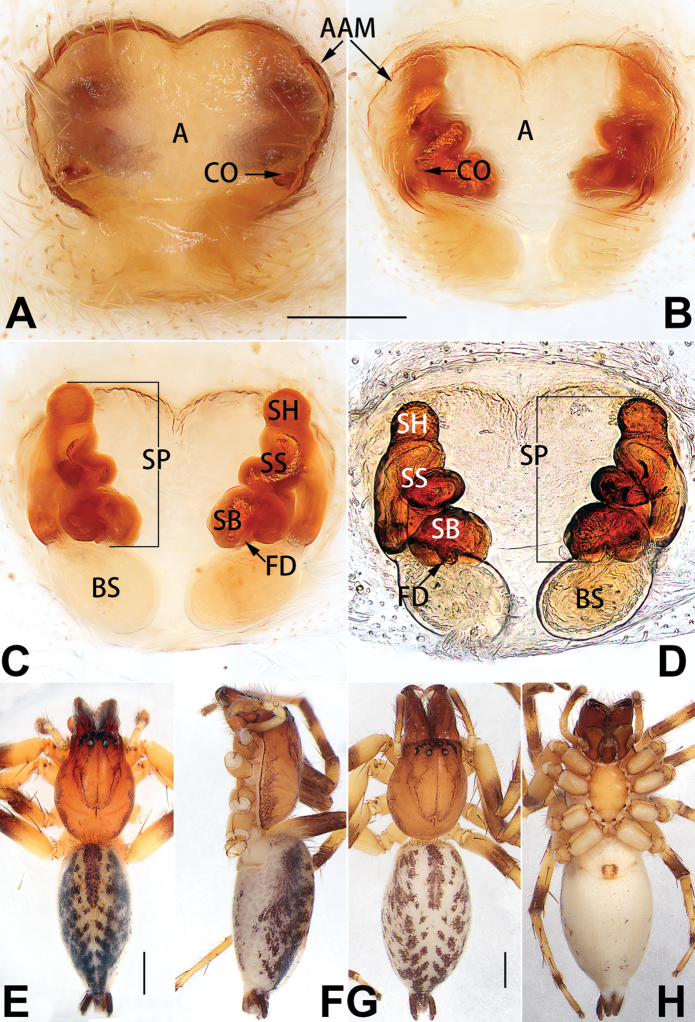
*Clubiona grucollaris*, epigyne (**A–D**), male habitus (**E, F**) and female habitus (**G, H**) **A** intact, ventral view **B** cleared, ventral view **C** cleared, dorsal view **D** cleared, dorsal view **E** dorsal view **F** lateral view **G** dorsal view **H** ventral view. Abbreviations: A = atrium; AAM = atrial anterior margin; BS = bursa; CO = copulatory opening; FD = fertilisation duct; SB = spermathecal base; SH = spermathecal head; SP = spermatheca; SS = spermathecal stalk. Scale bars: 0.2 mm (equal for **A–D**); 1 mm (equal for **E, F**, equal for **G, H**).

Figs 40 , 41 , 60A , 70A , 79C , 87C , 95C

Clubiona grucollaris Yu et al., 2017a: 4, figs 1–2, 4–6, 13–19 (♂♀).

##### Material examined.

1♂ (YHCLU0105), 1♀ (YHCLU0106), China: Yunnan Province: Xishuangbanna: Jinghong City: Menga Town: Wengnan Village: secondary forest, 22°4.997'N, 100°22.223'E, ca. 1137 m, 25.VII.2012, Q.Y. Zhao and Z.G. Chen leg.

##### Diagnosis and description.

See Yu et al. (2017a). Male palp as in Figs 40, 60A, 70A, epigyne as in Figs 41A–D, 79C, 87C, 95C, habitus as in Fig. 41E–H.

##### Distribution.

China (Hainan, Guizhou, and Yunnan).

##### Most similar species.

*Clubiona lala*.

#### Clubiona lala

Taxon classificationAnimaliaAraneaeClubionidae

Jäger & Dankittipakul, 2010

47C41D5D-FD27-522E-B6D6-E4FE4DC14F82

**Figure 42.**
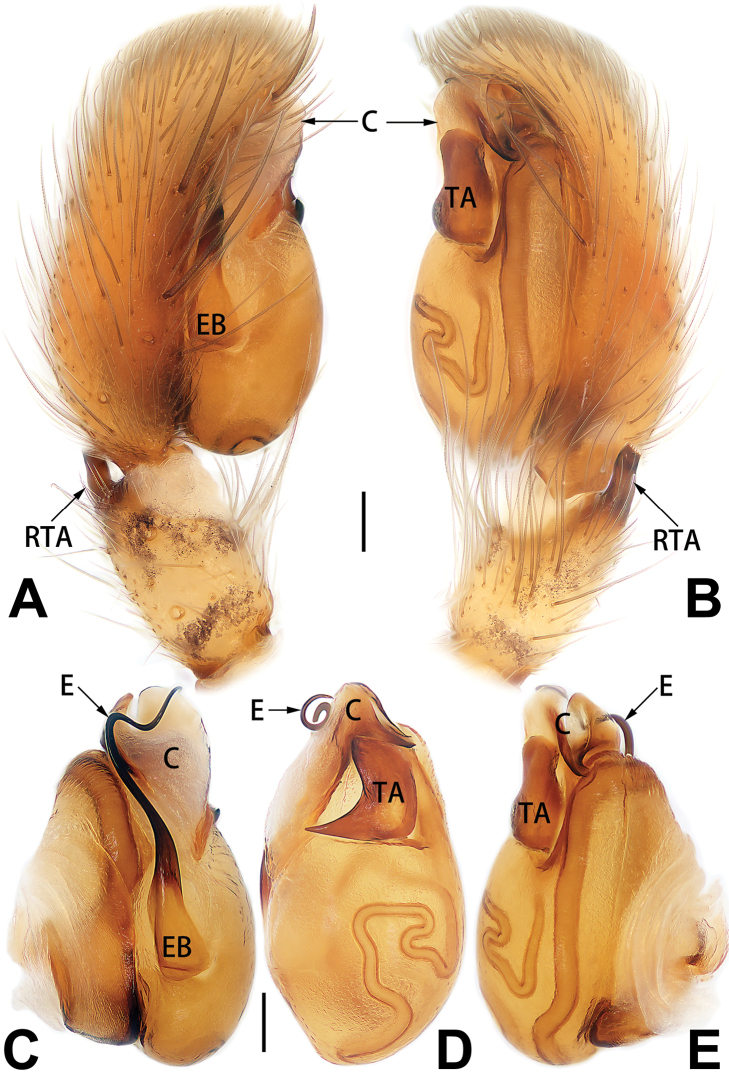
Male palp of *Clubiona lala***A** prolateral view **B** retrolateral view **C** bulb, prolateral view **D** bulb, ventral view **E** bulb, retrolateral view. Abbreviations: C = conductor; E = embolus; EB = embolar base; RTA = retrolateral tibial apophysis; TA = tegular hump. Scale bars: 0.1 mm (equal for **A, B**, equal for **C–E**).

**Figure 43.**
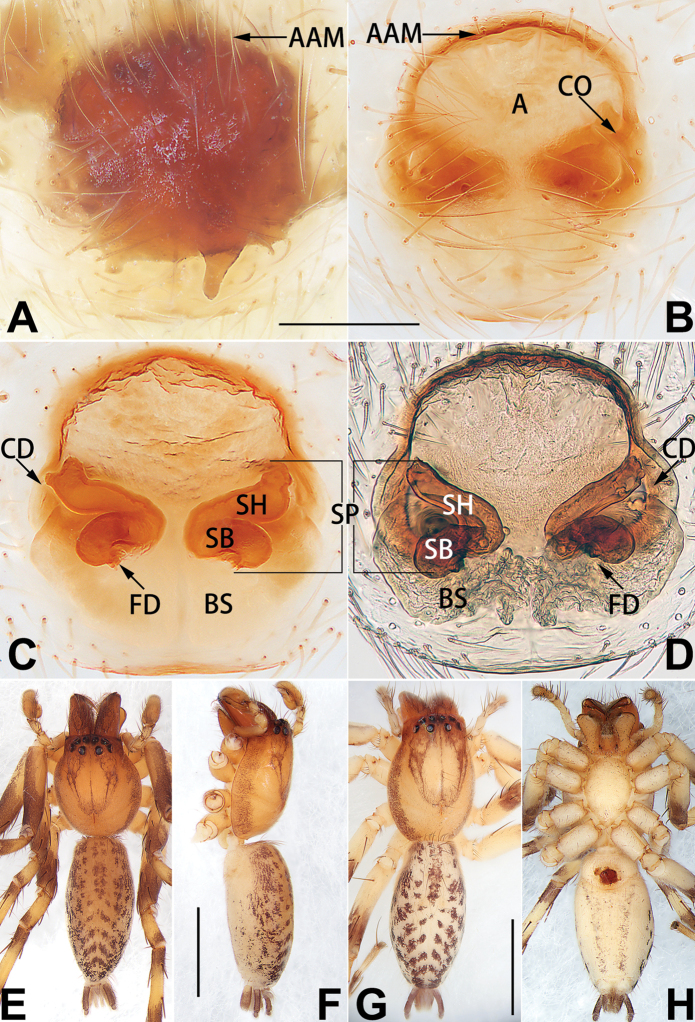
*Clubiona lala*, epigyne (**A–D**), male habitus (**E, F**) and female habitus (**G, H**) **A** intact, ventral view **B** cleared, ventral view **C** cleared, dorsal view **D** cleared, dorsal view **E** dorsal view **F** lateral view **G** dorsal view **H** ventral view. Abbreviations: A = atrium; AAM = atrial anterior margin; BS = bursa; CD = copulatory duct; CO = copulatory opening; FD = fertilisation duct; SB = spermathecal base; SH = spermathecal head; SP = spermatheca. Scale bars: 0.2 mm (equal for **A–D**); 2 mm (equal for **E, F**, equal for **G, H**).

Figs 42 , 43 , 60B , 70B , 79D , 87D , 95D

Clubiona lala Jäger & Dankittipakul, 2010: 29, figs 22–25, 28–30 (♀).

##### Material examined.

1♂, China: Yunnan Province: Xishuangbanna: Mengla County: Menglun Town: XTBG, tropical evergreen rainforest, 21°55.139'N, 101°16.295'E, ca. 523 m, 30.XI.2009, G. Tang and Z.Y. Yao leg; 1♀, XTBG, rubber plantation, 21°54.554N, 101°16.311'E, ca. 570 m, 14.V.2019, Z.G. Chen leg; 1♂ (YHCLU0110), Mengla County: Xiaolongha Village, 21°24.159'N, 101°37.178'E, ca. 635 m, 14.V.2019, Q.Y. Zhao and C.X. Gao leg; 1♀ (YHCLU0111), Jinghong City: Mengla County: Bubang Village, 21°36.384'N, 101°34.543'E, ca. 823 m, 10.VII.2012, Q.Y. Zhao and C.X. Gao leg.

##### Diagnosis.

The male of *C. lala* resembles those of *C. grucollaris* (Figs 40, 60A, 70A) in having a long, columnar conductor base and beak-shaped conductor apex but differs in the following: the embolar apex is coiled (Figs 42D, E, 60B) (vs. not coiled; Figs 40, 60A); the tegular apophysis is boomerang-shaped in ventral view (Figs 42D, 60B) (vs. petal-shaped; Figs 40B, D, E, 60A, 70A); the finger-like retrolateral tibial apophysis (Figs 42A, B, 70B) (vs. triangular; Figs 40A, B, 70A). The female of *C. lala* can be separated from that of *C. grucollaris* by the atrium anterior margin medially not concave (vs. medially concave) (cf. Fig. 87C and 87D), spermathecae tubular, consisting of base and head (vs. ascending spirally, consisting of base, stalk, and head) (cf. Fig. 95C and 95D). The female of *C. lala* also appears to be closely related to *C. campylacantha* (Dankittipakul and Singtripop 2008a: 38, figs 2–4, 13, 14, 38–40), *C. octoginta* (Dankittipakul and Singtripop 2008a: 39, figs 18–19, 45–47) and *C. reichlini* (Figs 37A–D, 79B, 87B) by the general shape of the atrium and vulva but can be easily distinguished from these species by the: (1) more rectangular atrium (Figs 43A, B, 87D) (vs. atrium more cambered); (2) spermathecae posterior to the atrium, well separated from atrium anterior margin (Figs 43C, D, 95D) (vs. spermathecae situated anteriorly, close to atrium anterior margin); (3) spermathecae separated by ca. one diameter (Figs 43C, D, 95D). (vs. spermathecae close together).

##### Description.

**Male.** (Fig. 43E, F): Total length 6.30; carapace 2.95 long, 1.96 wide; opisthosoma 3.53 long, 1.53 wide. Carapace brown, distinctly dark brown in ocular area, with a distinct pattern on pars cephalica consisting of a pair of dark, lateral bands and Ψ-shaped markings behind PER; ocular region distinctly narrowed, cervical groove and radial grooves indistinguishable; tegument smooth, clothed with short, dense setae. Eyes: AER slightly recurved, PER slightly procurved, the former wider than the latter. Eye sizes and interdistances: AME 0.11, ALE 0.15, PME 0.12, PLE 0.13, AME–AME 0.12, AME–ALE 0.10, PME–PME 0.27, PME–PLE 0.16, MOQL 0.36, MOQA 0.35, MOQP 0.52. Chelicerae robust and dark brown, dorsally with dark pattern. Cheliceral furrow with three anterior and two posterior teeth. Sternum yellowish white, 1.47 long, 0.96 wide. Labium and endites light orange. Legs brownish, all legs with conspicuous dark brown annuli on the distal parts of the femur, patella, tibia, metatarsus, and tarsus. Leg measurements: I 6.51 (1.78, 2.67, 1.32, 0.75), II 6.99 (1.90, 2.80, 1.44, 0.86), III 5.62 (1.72, 1.60, 1.74, 0.56), IV 8.29 (2.44, 2.93, 2.29, 0.60). Abdomen brown, with conspicuous anterior setal tufts; dorsum light yellow, antero-laterally with disconnected longitudinal bands, posteriorly with dark purple markings; venter with two indistinct purplish longitudinal markings.

Palp (Figs 42A–E, 60B, 70B). Tibia short, ca. 1/2 × cymbium length, with retrolateral apophysis; RTA digitiform, broad at base, apex truncated. Bulb more or less spherical, ca. twice longer than wide, oval; sperm duct sinuate, running an irregular course in the postero-retrolateral part of the tegulum. Embolus flagelliform; embolar base situated meso-prolaterally on the tegulum; embolar apex coiled, resting on an apical portion of the tegulum, covered by conductor in prolateral view. Conductor large, longer than 1/2 length of tegulum, with a heavily sclerotised and beak-shaped apex, base membranous, long, and columnar. Tegular apophysis heavily sclerotised, boomerang-shaped in ventral view.

**Female.** See Jäger and Dankittipakul (2010). Epigyne as in Figs 43A–D, 79D, 87D, 95D, habitus as in Fig. 43G–H.

##### Distribution.

Laos, China (Yunnan).

##### Remarks.

Male of the species is described for the first time.

#### Clubiona melanosticta

Taxon classificationAnimaliaAraneaeClubionidae

Thorell, 1890

04ED1012-F39A-5962-A83E-B43F178036F7

**Figure 44.**
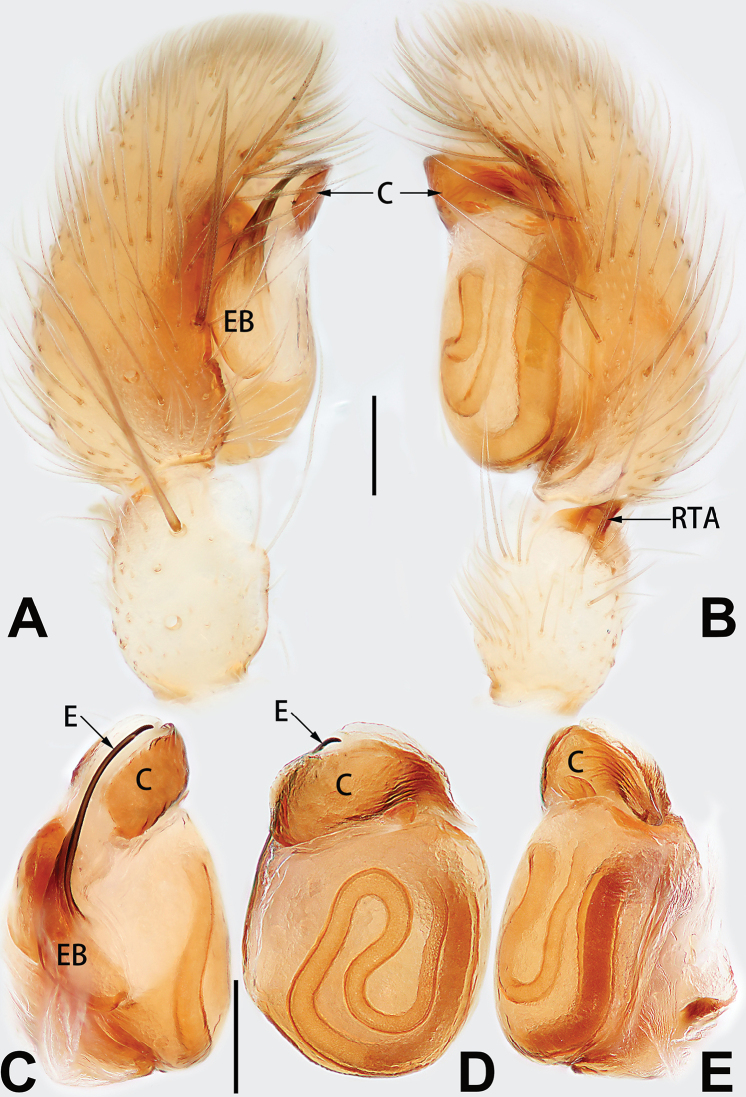
Male palp of *Clubiona melanosticta***A** prolateral view **B** retrolateral view **C** bulb, prolateral view **D** bulb, ventral view **E** bulb, retrolateral view. Abbreviations: C = conductor; E = embolus; EB = embolar base; RTA = retrolateral tibial apophysis. Scale bars: 0.1 mm (equal for **A, B**, equal for **C–E**).

**Figure 45.**
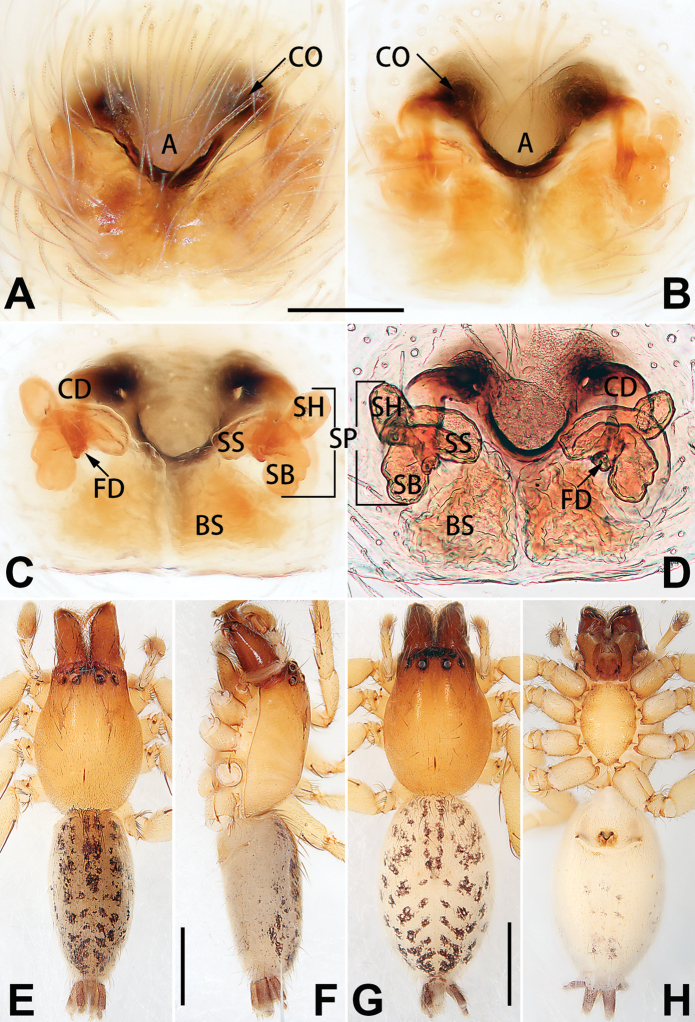
*Clubiona melanosticta*, epigyne (**A–D**), male habitus (**E, F**) and female habitus (**G, H**) **A** intact, ventral view **B** cleared, ventral view **C** cleared, dorsal view **D** cleared, dorsal view **E** dorsal view **F** lateral view **G** dorsal view **H** ventral view. Abbreviations: A = atrium; BS = bursa; CD = copulatory duct; CO = copulatory opening; FD = fertilisation duct; SB = spermathecal base; SH = spermathecal head; SP = spermatheca; SS = spermathecal stalk. Scale bars: 0.1 mm (equal for **A–D**); 1 mm (equal for **E, F**, equal for **G, H**).

Figs 44 , 45 , 59A , 69A , 78F , 86F , 94F

Clubiona melanosticta Thorell, 1890: 374 (♂); Thorell 1895: 42 (♀); Deeleman-Reinhold 2001: 123, figs 51–52 (♂); Dankittipakul and Singtripop 2008a: 42, figs 8–10, 52–54 (♂).
Clubiona melanothele Thorell, 1895: 42 (♀). Syn. nov.

##### Material examined.

1♂3♀, China: Yunnan Province: Xishuangbanna: Mengla County: Menglun Town: XTBG, *Paramichelia baillonii*plantation (ca. 20 yr.), 21°54.200'N, 101°16.923'E, ca. 608 m, 18.VIII.2007, G. Zheng leg; 1♂ (YHCLU0011), XTBG, teak plantation, 21°54.117'N, 101°16.167'E, ca. 549 m, 8.VIII.2018, H. Yu et al. leg; 1♀ (YHCLU0164), XTBG, 100 acre-feet sample plot (beside a hut), 21°54.117'N, 101°16.167'E, ca. 549 m, 11.VIII.2018, H. Yu et al.

##### Diagnosis and description.

See Deeleman-Reinhold (2001). Male palp as in Figs 44, 59A, 69A, epigyne as in Figs 45A–D, 78F, 86F, 94F, habitus as in Fig. 45E–H.

##### Remarks.

*Clubiona melanosticta* and *C. melanothele* were considered separate species for more than 120 years. After examining the holotypes, Deeleman-Reinhold (2001) illustrated the two species and suggested that they could be conspecific; however, she made no taxonomic changes at the time. New material has been collected from Xishuangbanna containing both sexes. According to drawings of Deeleman-Reinhold (2001), the males were identified as *C. melanosticta*, and the females were identified as *C. melanothele*. Based on morphology (Fig. 45E–H) and DNA barcoding data (Table 1), we matched the females and males together. Therefore, the two names are synonymised, and priority is given to *C. melanosticta*.

##### Distribution.

Thailand (Chiang Mai, Samut Songkram), Indonesia (Sumatra, Krakatau), New Guinea, Myanmar, Laos, China (Yunnan).

##### Most similar species.

*Clubiona zhanggureni*.

#### Clubiona suthepica

Taxon classificationAnimaliaAraneaeClubionidae

Dankittipakul, 2008

1DA253F6-FBCE-5269-8E8E-9770EDFE319B

**Figure 46.**
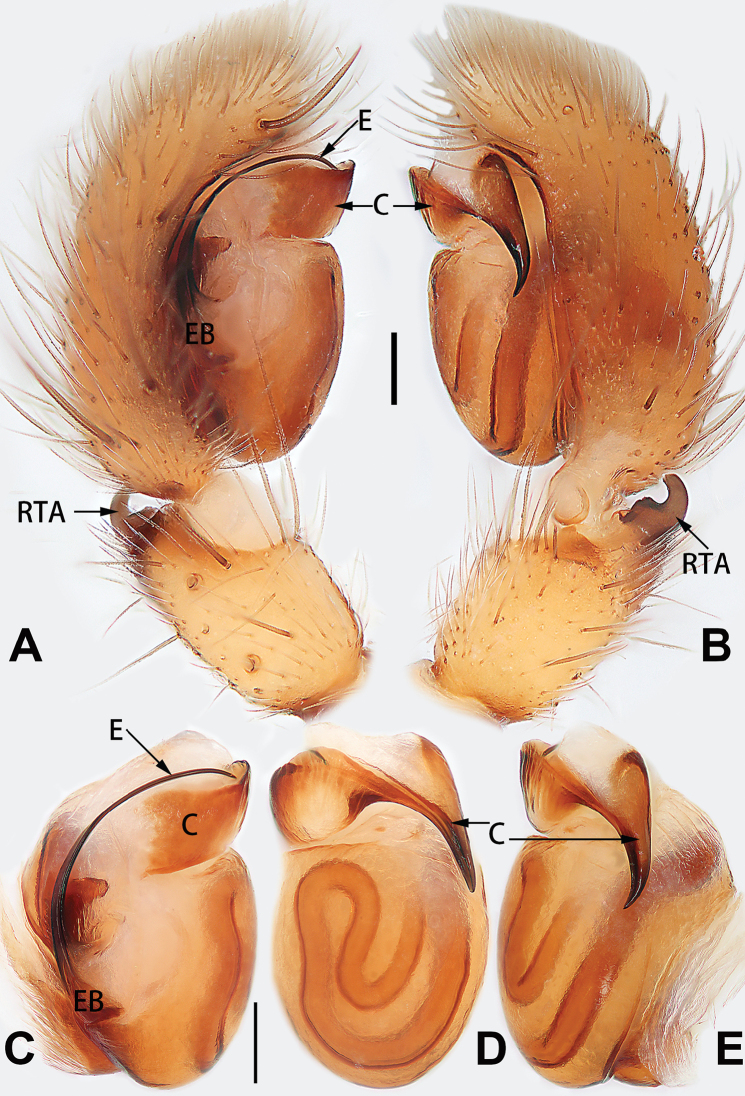
Male palp of *Clubiona suthepica***A** prolateral view **B** retrolateral view **C** bulb, prolateral view **D** bulb, ventral view **E** bulb, retrolateral view. Abbreviations: C = conductor; E = embolus; EB = embolar base; RTA = retrolateral tibial apophysis. Scale bars: 0.1 mm (equal for **A, B**, equal for **C–E**).

**Figure 47.**
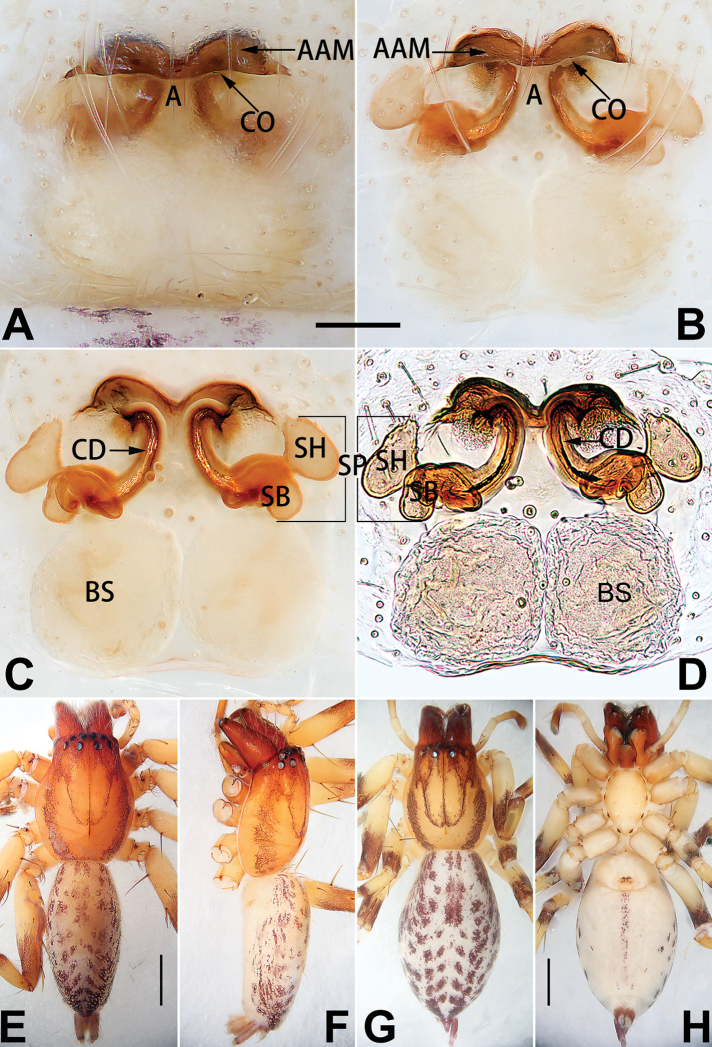
*Clubiona suthepica*, epigyne (**A–D**), male habitus (**E, F**) and female habitus (**G, H**) **A** intact, ventral view **B** cleared, ventral view **C** cleared, dorsal view **D** cleared, dorsal view **E** dorsal view **F** lateral view **G** dorsal view **H** ventral view. Abbreviations: A = atrium; AAM = atrial anterior margin; BS = bursa; CD = copulatory duct; CO = copulatory opening; SB = spermathecal base; SH = spermathecal head; SP = spermatheca. Scale bars: 0.1 mm (equal for **A–D**); 1 mm (equal for **E, F**, equal for **G, H**).

Figs 46 , 47 , 59D , 69D , 79E , 87E , 95E

Clubiona suthepica Dankittipakul, in Dankittipakul and Singtripop 2008a 42, figs 22, 23, 55–58 (♂ only, ♀ mismatched).

##### Material examined.

1♂, China: Yunnan Province: Xishuangbanna: Mengla County: Menglun Town: XTBG, secondary tropical montane evergreen broad-leaved forest, 21°57.534'N, 101°12.300'E, ca. 860 m, 4.VIII.2007, Guo Zheng leg; 1♂ (YHCLU0114), XTBG, 48^th^ km landmark in Menglun Nature Reserve, 21°58.704'N, 101°19.748'E, ca. 1088 m, 12.VIII.2011, G. Zheng et al. leg; 1♀ (YHCLU0209), XTBG, 48^th^ km landmark in Menglun Nature Reserve, 21°58.764'N, 101°19.748'E, ca. 1038 m, 10.VIII.2011, Q.Y. Zhao and Z.G. Chen leg.

##### Diagnosis.

Females of *C. suthepica* can be easily distinguished from other members of the group by the heavily sclerotised anterior margin of the atrium (Figs 47A, B, 79E, 87E). The male of *C. suthepica* differs from other members of the group by having a hook-shaped retrolateral tibial apophysis (Figs 46B, 69D) (vs. retrolateral tibial apophysis variable but not hook-shaped; for example, triangular in *C. melanosticta* and *C. zhanggureni*, hammer-like or clavate in *C. banna* sp. nov., digitiform in *C. lala* and *C. yueya*.; Figs 68C, 69A, B, 70B, D), the conductor apex terminating at ca. 9 o’clock position (Figs 46B, 59D) (vs. relatively shorter tip terminating at ca. 7–8 o’clock position in other species of the group; Figs 58C, 59A–C, 60A–D).

##### Description.

**Male.** See Dankittipakul and Singtripop (2008a). Palp as in Figs 46, 59D, 69D, habitus as in Fig. 47E, F.

**Female.** (Fig. 47G, H): Total length 6.46; carapace 2.63 long, 1.88 wide; opisthosoma 3.83 long, 2.16 wide. Carapace brown, distinctly dark brown in ocular area, with a distinctive pattern on pars cephalica consisting of a pair of dark lateral bands and Ψ-shaped markings behind PER; ocular area slightly narrowed, cervical groove and radial grooves indistinguishable; tegument smooth, clothed with short setae. Eyes: AER slightly recurved, PER slightly wider than AER, almost straight in dorsal view. Eye sizes and interdistances: AME 0.14, ALE 0.15, PME 0.14, PLE 0.13, AME–AME 0.13, AME–ALE 0.10, PME–PME 0.28, PME–PLE 0.20, MOQL 0.39, MOQA 0.37, MOQP 0.54. Chelicerae robust and dark brownish red, cheliceral furrow with three anterior and two posterior teeth. Sternum light yellow, 1.02 long, 0.68 wide. Labium and endites orange. Legs light yellow, femora with a broad distal band occupying almost half its length; tibiae with broad distal and proximal annuli; metatarsi with dark, thin distal annulus; tarsi pale yellow. Leg measurements: I 6.21 (1.83, 2.44, 1.17, 0.77), II 6.43 (1.89, 2.44, 1.28, 0.83), III 5.37 (1.65, 1.80, 1.34, 0.59), IV 7.35 (1.92, 2.57, 2.14, 0.73). Abdomen oval, with conspicuous anterior setal tufts, dorsum with dense grey setae and a broken purplish median band, half opisthosoma length, posteriorly with paired purplish markings consisting of numerous stripes and spots; venter yellowish white, medially with a longitudinal and linear marking.

Epigyne (Figs 47A–D, 79E, 87E, 95E). Epigynal plate nearly square, copulatory ducts visible through transparent integument, ca. 1/3 epigyne width. Anterior margin (or hood) heavily sclerotised, M-shaped, distinctly wide, almost equal to epigyne width. Copulatory openings indistinct, located in the hood. Copulatory ducts relatively long, nearly equal to bursal diameter. Spermathecae consisting of fan-shaped head and lobe-shaped base, with small fertilisation ducts terminally; the two spermathecal bases separated by 1.2 × length. Bursae close together, more or less spherical, surface translucent and wrinkled.

##### Distribution.

Thailand (Chiang Mai), China (Yunnan).

##### Remarks.

The female of the species is described for the first time.

#### Clubiona yueya

Taxon classificationAnimaliaAraneaeClubionidae

Yu & Li, 2019

491BF920-D6AC-555F-8242-A20FD162381F

Figs 60D , 70D , 79F , 87F , 95F

Clubiona yueya Yu & Li, 2019b: 215, figs 11A–E, 12A–G (♂♀).

##### Material examined.

***Types*.** Holotype ♂ (IZCAS Ar 34709), 1♀ (paratype, IZCAS Ar 34711), China: Yunnan Province: Xishuangbanna: Mengla County: Menglun Town: XTBG, 300 acre-feet bamboo plantation, 21°53.901'N, 101°16.884'E, ca. 515 m, 7.VIII.2018, H. Yu and Z.G. Chen leg. ***Other material examined.*** 1♂ (YHCLU0116) and 1♀ (YHCLU0117), same locality as holotype, 12.V.2019, Z.G. Chen et al. leg.

##### Diagnosis and description.

See Yu and Li (2019b). Male palp as in Figs 60D, 70D, epigyne as in Figs 79F, 87F, 95F.

##### Distribution.

Known only from Xishuangbanna.

##### Most similar species.

*Clubiona lala*.

#### Clubiona zhanggureni

Taxon classificationAnimaliaAraneaeClubionidae

Yu & Li, 2019

6677F0BD-2079-5C22-88F1-2258376890EE

Figs 59B , 69B

Clubiona zhanggureni Yu & Li, 2019b: 216, figs 13A–E, 14A–C (♂).

##### Material examined.

***Types*.** Holotype ♂ (IZCAS Ar 34714), 1♂ (paratype, IZCAS Ar 34715), China: Yunnan Province: Xishuangbanna: Mengla County: Menglun Town: XTBG, secondary tropical montane evergreen broad-leaved forest, 21°57.809'N, 101°12.173'E, ca. 888 m, 4.VIII.2007, G. Zheng leg.

##### Diagnosis and description.

See Yu and Li (2019b). Palp as in Figs 59B, 69B.

##### Distribution.

Known only from Xishuangbanna.

##### Most similar species.

*Clubiona melanosticta*.

#### Clubiona zhangyongjingi

Taxon classificationAnimaliaAraneaeClubionidae

Li & Blick, 2019

16A820C6-8D08-50FC-8D67-A9961EB38603

Clubiona transversa Zhang & Yin, 1998: 14, figs 16–19 (♀ only).
Clubiona zhangyongjingi Li & Blick, 2019: 131 (replacement name for C. transversa; ♂ mismatched).

##### Material examined.

None.

##### Diagnosis and description.

See Zhang and Yin (1998).

##### Distribution.

China (Yunnan).

##### Remarks.

Based on the original figures, the female is almost the same as that of *C. melanosticta*, and the male belongs to the *C. ternatensis* group and resembles *C. kuu* and *C. subkuu*.

#### Clubiona trivialis

Taxon classificationAnimaliaAraneaeClubionidae

group

1B4E6DE7-D792-59E6-9EA3-9B0A984C21A1

Microclubiona : Lohmander, 1944: 20 (type species C. trivialis C.L. Koch, 1834).
Clubiona trivialis group: Dondale and Redner 1976: 1155; Mikhailov 1995: 43.

##### Diagnosis.

See Dondale and Redner (1976) and Yu and Li (2019a).

##### Description.

See Dondale and Redner (1976) and Mikhailov (1995).

##### Composition and distribution.

Currently, the *trivialis* group includes at least 28 species mainly distributed in Eurasia and Australia (Mikhailov 1995; Dondale and Redner 1982; Dong and Zhang 2016). Among these, at least 16 species (including the new species described here) have been recorded from China (see Table 6).

##### Comments.

Lohmander (1944) established the genus *Microclubiona* with the type species *C. trivialis*. However, Lohmander’s work was neglected by North American taxonomists. *Clubiona trivialis* and related species were placed in Locket and Millidge’s (1951) Group III and Edwards’ (1958) Group I, respectively. The *Clubiona trivialis* group was formally established by Dondale and Redner (1976) and was redefined by Mikhailov (1995) based on 19 Holarctic species. The group had been separated from genus *Clubiona* sensu lato, to be resurrected to genus level by Wunderlich (2011) but latter synonymised with *Clubiona* by Mikhailov (2012). The present study follows Mikhailov (2012) and the WSC (2021) in regarding *Microclubiona* as a synonym of *Clubiona* rather than revalidating the generic status of the *trivialis* group. Consequently, we temporarily place the five Xishuangbanna species in *Clubiona* sensu lato and assign them to *C. trivialis* group.

**Table 6.** Clubiona*trivialis* group.

	Species name	Known sex	Distribution
1	*C. amurensis* Mikhailov, 1990	♂♀	Russia (Far East), Japan (Hokkaido)
2	*C. asrevida* Ono, 1992	♂♀	China (Taiwan)
3	*C. baimaensis* Song & Zhu, 1991	♂♀	China (Hubei, Hunan, Sichuan)
4	*C. basarukini* Mikhailov, 1990	♂♀	Russia (South Siberia, Far East), Mongolia, Japan (Hokkaido)
5	*C. bicornis* Yu & Li, 2019	♂♀	China (Yunnan)
6	*C. cheni* Yu & Li, 2019	♂♀	China (Yunnan)
7	*C. diversa* O. Pickard-Cambridge, 1862	♂♀	Trans Palaearctic
8	*C. duoconcava* Zhang & Hu, 1991	♂♀	South China
9	*C. hedini* Schenkel, 1936	♀	China (Hunan, Gansu)
10	*C. hooda* Dong & Zhang, 2016	♂♀	China (Hebei)
11	*C. huaban* Xin, Zhang, Li, Zeng & Yu, 2020	♂	China (Guizhou)
12	*C. insulana* Ono, 1989	♂♀	China (Taiwan), Japan (Ryukyu Is.)
13	*C. janae* Edwards, 1958	♀	USA (California)
14	*C. juvenis* Simon, 1878	♂♀	West Palaearctic
15	*C. moesta* Banks, 1896	♂♀	Nearctic, China (Hunan, Hubei, Qinghai, Guizhou)
16	*C. pygmaea* Banks, 1892	♂♀	Nearctic
17	*C. quebecana* Dondale & Redner, 1976	♂♀	Nearctic
18	*C. rostrata* Paik, 1985	♂♀	FE Palaearctic
19	*C. subasrevida* Yu & Li, 2019	♂♀	China (Yunnan)
20	*C. subquebecana* Yu & Li, 2019	♂♀	China (Yunnan)
21	*C. subrostrata* Zhang & Hu, 1991	♂♀	China (Fujian, Hunan, Guizhou)
22	*C. subtilis* L. Koch, 1867	♂♀	Trans Palaearctic
23	*C. subtrivialis* Strand, 1906	♂♀	East Africa
24	*C. subyangmingensis* Gan & Wang, 2020	♂♀	China (Guizhou)
25	*C. transbaicalica* Mikhailov, 1992	♂	Baikal Lake
26	*C. trivialis* C. L. Koch, 1843	♂♀	Holarctic
27	*C. yangmingensis* Hayashi & Yoshida, 1993	♂♀	China (Taiwan)
28	*C. menglun* Yu & Li, sp. nov.	♀	China (Yunnan)

#### Key to *C. trivialis* group species occurring in Xishuangbanna (males)

**Table:** 

1	Palp with prolateral tibial apophysis (Figs 61A, B, 71A, B)	**2**
–	Palp without prolateral tibial apophysis (Figs 61C, D, 71C, D)	**3**
2	Retrolateral tibial apophysis branched, both ventral and dorsal branches sharply pointed (Fig. 71A); embolar base bearing only dentiform process (Figs 61A, 71A)	***C. bicornis***
–	Retrolateral tibial apophysis not branched, with a blunt tip (Fig. 71B); embolar base bearing two processes (Fig. 71B)	***C. cheni***
3	Retrolateral tibial apophysis broad, flat and triangular, with sharp tip (Figs 61C, 71C)	***C. subasrevida***
–	Retrolateral tibial apophysis small, thumb-like, with a blunt tip (Figs 61D, 71D)	***C. subquebecana***

#### Key to *C. trivialis* group species occurring in Xishuangbanna (females)

**Table:** 

1	Copulatory openings fused (Figs 78C, 86C)	***C. subasrevida***
–	Copulatory openings separated (Figs 77F, 78A, B, D, 85F, 86A, B, D)	**2**
2	Spermathecae larger than bursae (Figs 93F, 94D)	**3**
–	Spermathecae smaller than bursae (Fig. 94A, B)	**4**
3	Spermathecae peanut-shaped (Fig. 93F)	***C. bicornis***
–	Spermathecae subglobular (Fig. 94D)	***C. subquebecana***
4	Spermathecae ellipsoidal (Fig. 94A)	***C. cheni***
–	Spermathecae globular (Fig. 94B)	***C. menglun* sp. nov.**

#### Clubiona bicornis

Taxon classificationAnimaliaAraneaeClubionidae

Yu & Li, 2019

F9BE7B31-2B74-5F0F-AC3F-6FC32CB951AD

**Figure 48.**
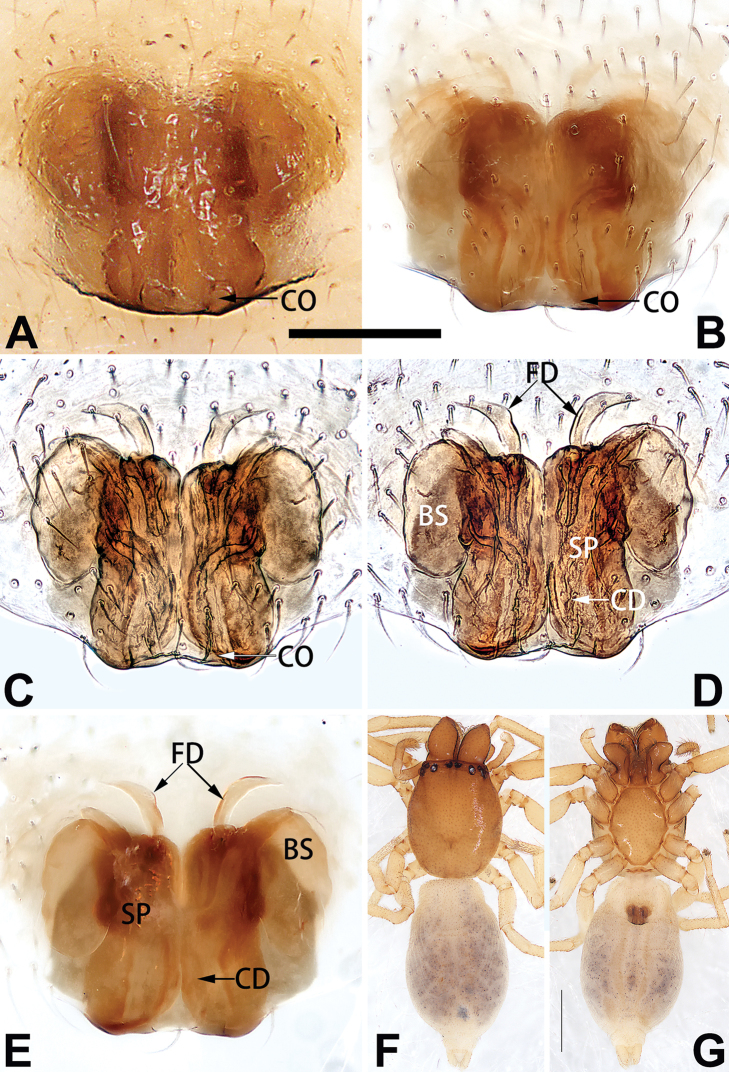
*Clubiona bicornis*, epigyne (**A–E**) and female habitus (**F, G**) **A** intact, ventral view **B** cleared, ventral view **C** cleared, ventral view **D** cleared, dorsal view **E** cleared, dorsal view **F** dorsal view **G** ventral view. Abbreviations: BS = bursa; CD = copulatory duct; CO = copulatory opening; FD = fertilisation duct; SP = spermatheca. Scale bars: 0.1 mm (equal for **A–E**); 0.5 mm (equal for **F**, **G**).

Figs 48 , 61A , 71A , 77F , 85F , 93F

Clubiona bicornis Yu & Li, 2019b: 221, figs 15A–E, 16A–C (♂).

##### Material examined.

***Type*.** Holotype ♂ (IZCAS Ar 34716), China: Yunnan Province: Xishuangbanna: Mengla County: Menglun Town: XTBG, primary tropical seasonal rainforest, 21°55.035'N, 101°16.500'E, ca. 558 m, 22.VII.2007, G. Zheng leg. ***Other material examined.*** 1♂ (YHCLU0180), Jinghong City: Menga Town: Wengnan Village: secondary forest, 22°4.598'N, 100°22.134'E, ca. 1137 m, 30.VII.2012, Q.Y. Zhao and Z.G. Chen leg; 1♀ (YHCLU0099), XTBG, 48^th^ km landmark in Menglun Nature Reserve, 21°58.704'N, 101°19.748'E, ca. 1088 m, 11.VIII.2011, G. Zheng et al. leg.

##### Diagnosis.

**Figure 49.**
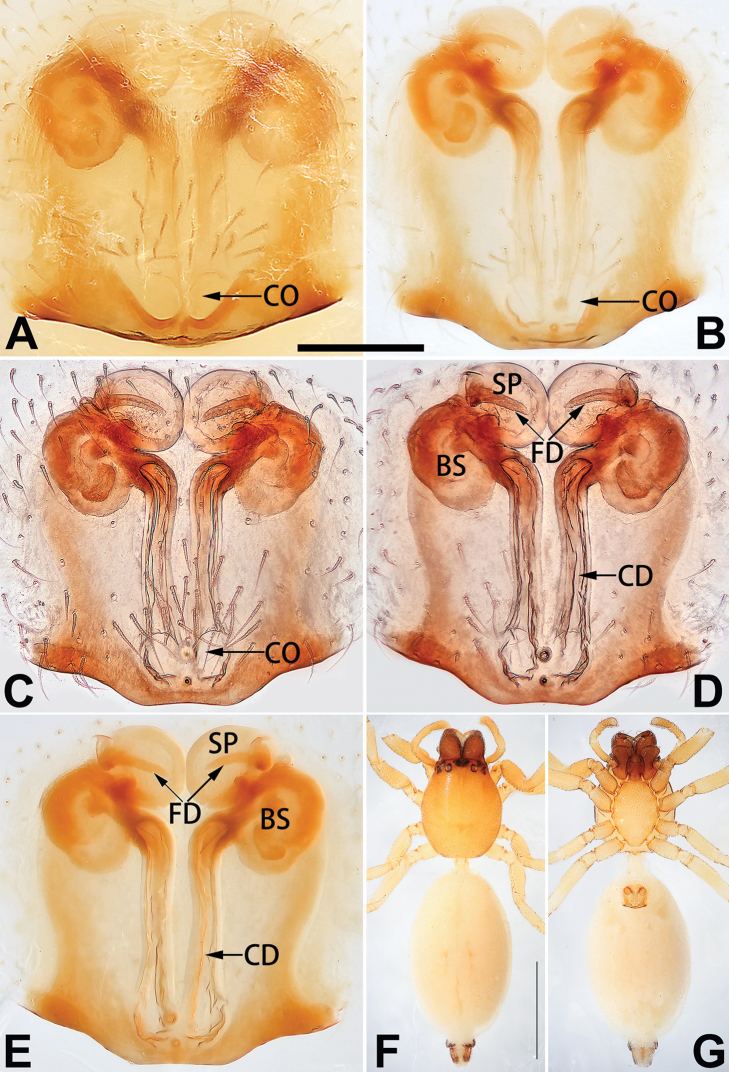
Holotype female of *Clubiona menglun* sp. nov. epigyne (**A–E**) and habitus (**F, G**) **A** intact, ventral view **B** cleared, ventral view **C** cleared, ventral view **D** cleared, dorsal view **E** cleared, dorsal view **F**dorsal view **G** ventral view. Abbreviations: BS = bursa; CD = copulatory duct; CO = copulatory opening; FD = fertilisation duct; SP = spermatheca. Scale bars: 0.1 mm (equal for **A–E**); 1 mm (equal for **F**, **G**).

Females of *C. bicornis* can be easily distinguished from other members of the group except *C. amurensis* (Mikhailov, 1990: 148, figs 21, 22) by the copulatory openings separated by one diameter (Figs 48A–C, 77F, 85F) (vs. copulatory openings fused or close together in almost all species of the *C. trivialis* group, including *C. subasrevida* and *C. menglun* sp. nov.; Figs 49A–C, 78B, C, 86B, C) and the lengthwise spermathecae (Figs 48D, E, 93F) (vs. spermathecae nearly globular in other species of the *trivialis* group, such as *C. cheni* and *C. menglun* sp. nov.; Figs 49D, E, 94A, B) but differ from the latter by the: (1) copulatory opening a small pore (Figs 48A–C, 77F, 85F) (vs. slit like in *C. amurensis*; Mikhailov, 1990: fig. 21); (2) peanut-shaped spermathecae (Figs 48D, E, 93F) (vs. elliptical in *C. amurensis*; Mikhailov, 1990: fig. 22); (3) proximal half of the copulatory ducts close together (Figs 48D, E, 93F) (vs. widely separated by more than four diameters in *C. amurensis*; Mikhailov, 1990: fig. 22); (4)fovea indistinct (Fig. 48F) (vs. fovea distinct in *C. amurensis*).

##### Description.

**Male.** See Yu and Li (2019b). palp as in Figs 61A, 71A.

**Female.** (Fig. 48F, G): Total length 2.33; carapace 0.99 long, 0.77 wide; opisthosoma 1.34 long, 0.83 wide. Carapace, in profile almost flat, brown, slightly darker in front, with a pair of indistinct short lines running longitudinally from behind AME, fovea indistinct; ocular region slightly narrowed, cervical groove and radial grooves indistinct; tegument smooth, clothed with numerous short, fine setae. Eyes: AER almost straight, PER slightly recurved and slightly wider than AER in dorsal view. Eye sizes and interdistances: AME 0.05, ALE 0.06, PME 0.04, PLE 0.05, AME–AME 0.03, AME–ALE 0.13, PME–PME 0.27, PME–PLE 0.08, MOQL 0.17, MOQA 0.13, MOQP 0.32. Chelicerae protruding and robust, coloured as carapace, with distinct lateral bulge, cheliceral furrow with three anterior and two posterior teeth Sternum pale brown, 0.64 long, 0.45 wide. Labium and endites coloured as carapace. Legs light brown, without distinct markings. Leg measurements: I 1.72 (0.48, 0.77, 0.35, 0.13), II 1.69 (0.51, 0.62, 0.40, 0.16), III 1.33 (0.41, 0.48, 0.28, 0.15), IV 1.82 (0.52, 0.78, 0.32, 0.20). Abdomen oval, cream coloured, numerous large pigmented markings prominently visible through integument except anteriorly and on the spinnerets.

Epigyne (Figs 48A–E, 77F, 85F, 93F). Epigynal plate slightly wider than long, the arrangement of the various parts of the vulva are indistinctly visible through the tegument. Copulatory openings indistinct, separated by one diameter, situated at medial portion of epigynal plate posterior margin. Hyaline copulatory ducts ascending in parallel, the proximal half close together, the distal half widely separated. Spermathecae close together, peanut- or gourd-shaped, ca. 2 × longer than wide. Fertilisation ducts curved and acicular, relatively long, > 1/3 spermathecae length, located on anterior surface of spermathecae. Bursae oblong, ca. 1.8 × longer than wide, with a smooth hyaline surface.

##### Distribution.

Known only from Xishuangbanna.

##### Remarks.

The female of the species is described for the first time.

#### Clubiona cheni

Taxon classificationAnimaliaAraneaeClubionidae

Yu & Li, 2019

FA335145-C750-57D5-8F0D-1F519AB1D1C5

Figs 61B , 71B , 78A , 86A , 94A

Clubiona cheni Yu & Li, 2019a: 171, figs 13A–E, 14A–H (♂♀).

##### Material examined.

***Types*.** Holotype ♂ (IZCAS Ar 34625), 1♀ (paratype, IZCAS Ar 34626), China: Yunnan Province: Xishuangbanna: Mengla County: Menglun Town: XTBG, low evergreen forest, 21°53.794'N, 101°17.152'E, ca. 594 m, 27.XI.2009, G. Tang and Z.Y. Yao leg. ***Other material examined.*** 1♂ (YHCLU0033), XTBG, secondary tropical forest, 21°54.168'N, 101°16.866'E, ca. 610 m, 31.VII.2018, Z.G. Chen leg; 1♀ (YHCLU0032), XTBG, low evergreen forest, 21°53.823'N, 101°17.072'E, ca. 613 m, 22.VII.2018, H. Yu et al. leg.

##### Diagnosis and description.

See Yu and Li (2019a). Male palp as in Figs 61B, 71B, epigyne as in Figs 78A, 86A, 94A.

##### Distribution.

Known only from Xishuangbanna.

##### Most similar species.

*Clubiona bicornis*.

#### Clubiona menglun

Taxon classificationAnimaliaAraneaeClubionidae

Yu & Li sp. nov.

53DFC2A4-CACB-5C4A-80E1-12DABFB354DE

http://zoobank.org/2CC441EE-1C9B-44EF-8EA7-F575A599F9DD

Figs 49 , 78B , 86B , 94B

##### Holotype.

♀ (IZCAS-Ar 34762), China: Yunnan Province: Xishuangbanna: Mengla County: Menglun Town: XTBG, secondary tropical seasonal moist forest, 22°54.390'N, 101°16.811'E, ca. 612 m, 10.VIII.2007, G. Zheng leg. ***Other material examined.*** 1♀ (YHCLU0097), Jinghong City: Menga Town: Wengnan Village: secondary forest, 22°4.997'N, 100°22.223'E, ca. 1137 m, 25.VII.2012, Q.Y. Zhao and Z.G. Chen leg.

##### Etymology.

The species name is derived from the name of the type locality; noun in apposition.

##### Diagnosis.

Females of *C. menglun* sp. nov. resemble those of *C. cheni* (Figs 78A, 86A, 94A) by having a similar general shape of the vulva but can be separated from them by the close spermathecae (vs. separated by ca. 0.5 diameter) (cf. Fig. 94B and 94A) and by the globular bursae (vs. ellipsoidal) (cf. Fig. 94B and 94A).

##### Description.

**Female.** Holotype (Fig. 49F, G): Total length 2.99; carapace 1.07 long, 0.82 wide; opisthosoma 1.83 long, 1.07 wide. Carapace orange, slightly darker in front, without distinct pattern, fovea almost indistinguishable; ocular region slightly narrowed, cervical groove indistinct; tegument smooth, clothed with numerous short, fine setae. Eyes: in dorsal view, both anterior and posterior eye rows recurved, PER slightly wider than AER. Eye sizes and interdistances: AME 0.06, ALE 0.03, PME 0.07, PLE 0.05, AME–AME 0.04, AME–ALE 0.04, PME–PME 0.20, PME–PLE 0.04, MOQL 0.13, MOQA 0.12, MOQP 0.31. Chelicerae robust and brownish red, with conspicuous condyle, three promarginal and two retromarginal teeth. Sternum pale yellow, 0.67 long, 0.42 wide. Labium and endites coloured as chelicerae. Legs light yellowish white, without distinct markings. Leg measurements: I 1.77 (0.52, 0.70, 0.34, 0.21), II 2.02 (0.56, 0.86, 0.38, 0.22), III 1.50 (0.48, 0.53, 0.32, 0.17), IV 2.03 (0.60, 0.74, 0.46, 0.23). Abdomen oval, cream coloured, slightly darker dorsally, without pattern.

Epigyne (Figs 49A–E, 78B, 86B, 94B). Epigynal plate slightly longer than wide, vulva clearly visible through the tegument. Copulatory openings distinct, close together, located close to posterior margin of epigynal plate. Hyaline copulatory ducts long and slender, almost parallel, ascending dorsally, then ascending obliquely, finally entering the connecting piece between the spermathecae and bursae. Both spermathecae and bursae globular and smooth, the former anteriad and smaller than the latter. Spermathecae close together, and bursae separated by one diameter. Fertilisation ducts acicular, relatively long, more than half spermathecae diameter, on dorsal surfaces of spermathecae.

**Male.** Unknown.

##### Comments.

Only two *trivialis* group species are known from males (Table 6): *C. transbaicalica* from South Siberia and *C. huaban* from Guizhou in China. We cannot rule out the possibility that *C. transbaicalica* is conspecific to *C. menglun* sp. nov. However, the probability is very small because of: (1) the long distance between the two type localities (Xishuangbanna is 3500 Km from the Selenga Distr.); (2) their different sizes (*C. menglun* sp. nov. is less than 3 mm, *C. transbaicalica* is 5.3–6.1 mm). *C. menglun* sp. nov. and *C. huaban* are considered separate species due to their different sizes (*C. menglun* sp. nov. with 3 mm vs. *C. huaban* with 4.7 mm; different colours (carapace orange, cream-coloured abdomen without pattern in *C. menglun* sp. nov. (vs. carapace light brown, yellowish brown abdomen marked with numerous brown spots in *C. huaban*).

##### Distribution.

Known only from the type locality, Xishuangbanna, Yunnan, China.

#### Clubiona subasrevida

Taxon classificationAnimaliaAraneaeClubionidae

Yu & Li, 2019

4BC18759-E76C-5B3A-A023-0F77818D70BA

Figs 61C , 71C , 78C , 86C , 94C

Clubiona subasrevida Yu & Li, 2019b: 221, figs 17A–E, 18A–H (♂♀).

##### Material examined.

**Types.** Holotype ♂ (IZCAS Ar 34717), 1♀ (paratype, IZCAS Ar 34718), China: Yunnan Province: Xishuangbanna: Mengla County: Menglun Town: XTBG, secondary tropical montane evergreen broad-leaved forest, 21°57.809'N, 101°12.173'E, ca. 888 m, 4.VIII.2007, G. Zheng leg. ***Other material examined.*** 1♂ (YHCLU0100), Huigang Village, monsoon forest, 21°37.027'N, 101°35.161'E, ca. 764 m, 12.VII.2012, Q.Y. Zhao and C.X. Gao leg; 1♀ (YHCLU0101), XTBG, 48^th^ km landmark in Menglun Nature Reserve, 21°58.704'N, 101°19.748'E, ca. 1088 m, 12.VIII.2011, G. Zheng et al. leg.

##### Diagnosis and description.

See Yu and Li (2019b). Male palp as in Figs 61C, 71C, epigyne as in Figs 78C, 86C, 94C.

##### Distribution.

Known only from Xishuangbanna.

##### Most similar species.

*Clubiona asrevida*.

#### Clubiona subquebecana

Taxon classificationAnimaliaAraneaeClubionidae

Yu & Li, 2019

B3EA9042-59F0-5B5E-ADCF-82608A75FAC9

Figs 61D , 71D , 78D , 86D , 94D

Clubiona subquebecana Yu & Li, 2019a: 174, figs 15A–E, 16A–H (♂♀).

##### Material examined.

***Types*.** Holotype ♂ (IZCAS Ar 34685), China: Yunnan Province: Xishuangbanna: Mengla County: Menglun Town: XTBG, secondary forest, 21°54.459'N, 101°16.755'E, ca. 644 m, 20.XI.2009, G. Tang and Z.Y. Yao leg; 1♀ (paratype, IZCAS Ar 34687), XTBG, G213 roadside, *Anogeissus acuminata*plantation, 21°53.819'N, 101°17.075'E, ca. 609 m, 27.XI.2009, G. Tang and Z.Y. Yao leg. ***Other material examined.*** 1♀ (YHCLU0103), Xiaolongha Village, 21°24.198'N, 101°37.013'E, ca. 801 m, 30.VI.2012, Q.Y. Zhao and C.X. Gao leg.

##### Diagnosis and description.

See Yu and Li (2019a). Male palp as in Figs 61D, 71D, epigyne as in Figs 78D, 86D, 94D.

##### Distribution.

Known only from Xishuangbanna.

##### Most similar species.

*Clubiona quebecana*.

###### Species not currently assigned to any group

#### Clubiona jiandan

Taxon classificationAnimaliaAraneaeClubionidae

Yu & Li, 2019

F03EB327-03FB-59BE-A58D-358B607D4B6D

Figs 62C , 72C , 80B , 88B , 96B

Clubiona jiandan Yu & Li, 2019b: 226, figs 19A–E, 20A–H (♂♀).

##### Material examined.

Holotype ♂ (IZCAS Ar 34720), China: Yunnan Province: Xishuangbanna: Mengla County: Menglun Town: XTBG, secondary tropical montane evergreen broad-leaved forest, 21°57.784'N, 101°11.947'E, ca. 895 m, 6.VIII.2007, G. Zheng leg; 1♀ (paratype, IZCAS Ar 34723), XTBG, secondary tropical montane evergreen broad-leaved forest, 21°57.528'N, 101°12.384'E, ca. 890 m, 6.VIII.2007, G. Zheng leg. ***Other material examined.*** 1♂ (YHCLU0066) and 1♀ (YHCLU0067), Xiaolongha Village, 21°24.198'N, 101°37.013'E, ca. 801 m, 30.VI.2012, Q.Y. Zhao and C.X. Gao leg.

##### Diagnosis and description.

See Yu and Li (2019b). Male palp as in Figs 62C, 72C, epigyne as in Figs 80B, 88B, 96B.

##### Distribution.

Known only from Xishuangbanna.

##### Most similar species.

*Clubiona yaoi*.

#### Clubiona shuangsi

Taxon classificationAnimaliaAraneaeClubionidae

Yu & Li sp. nov.

8ABA5954-341F-5560-81C5-92AB2096CC7D

http://zoobank.org/442232E1-4DB2-4AA1-86EA-DA3BA9E21858

**Figure 50.**
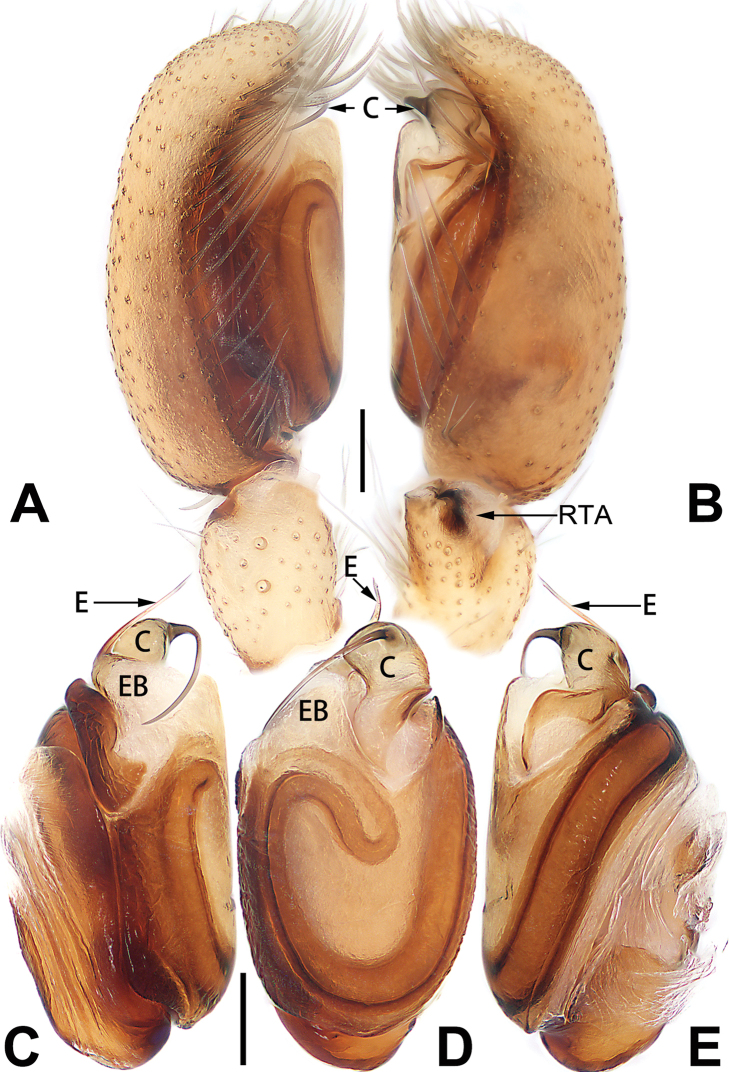
Male palp of the holotype of *Clubiona shuangsi* sp. nov. **A** prolateral view **B** retrolateral view **C** bulb, prolateral view **D** bulb, ventral view **E** bulb, retrolateral view. Abbreviations: C = conductor; E = embolus; EB = embolar base; RTA = retrolateral tibial apophysis. Scale bars: 0.1 mm (equal for **A, B**, equal for **C–E**).

**Figure 51.**
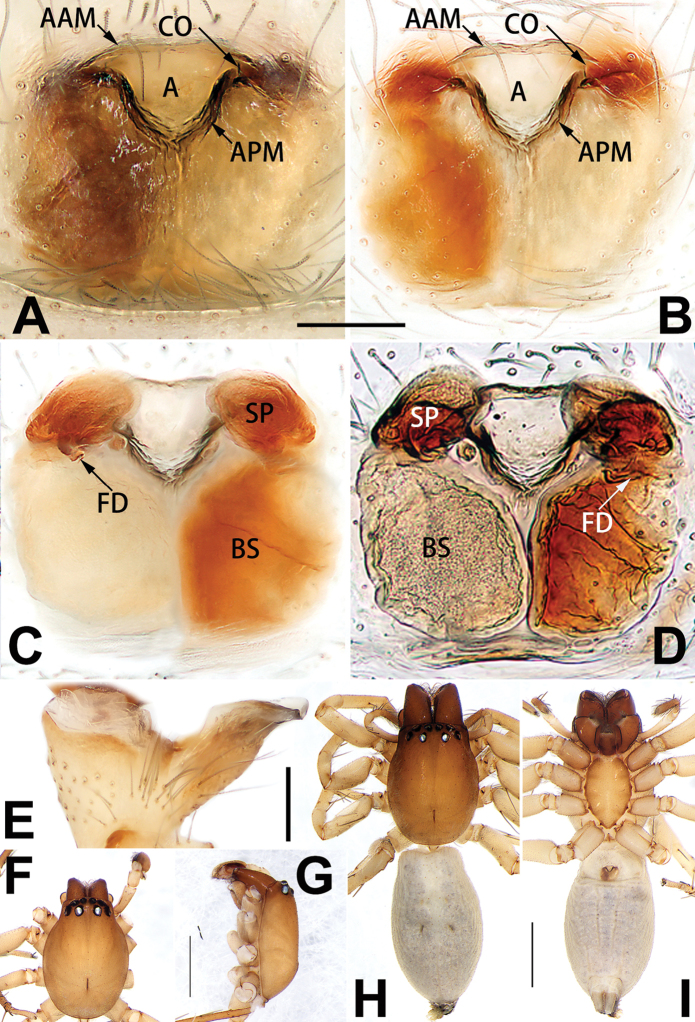
*Clubiona shuangsi* sp. nov., female paratype and male holotype, epigyne (**A–D**), tibia of male palp (**E**), male habitus (**F, G**) and female habitus (**H, I**) **A** intact, ventral view **B** cleared, ventral view **C** cleared, dorsal view **D** cleared, dorsal view **E** ventral view **F** dorsal view **G** lateral view **H** dorsal view **I** ventral view. Abbreviations: A = atrium; AAM = atrial anterior margin; APM = atrial posterior margin; BS = bursa; CO = copulatory opening; FD = fertilisation duct; SP = spermatheca. Scale bars: 0.1 mm (equal for **A–D**); 0.1 mm (**E**); 1 mm (equal for **E, G**, equal for **H, I**).

Figs 50 , 51 , 62D , 72D , 80D , 88D , 96D

##### Holotype.

♂ (IZCAS-Ar 34763, YHCLU0135), China: Yunnan Province: Xishuangbanna: Mengla County: Nanshahe Village: monsoon forest, 21°36.200'N, 101°34.384'E, ca. 826 m, 14.VII.2012, Q.Y. Zhao and C.X. Gao leg. ***Paratype***: 1♀ (IZCAS-Ar 34764), same data as holotype. ***Other material examined.*** 1♀ (YHCLU0085), same data as holotype.

##### Etymology.

The specific name is derived from the Chinese pinyin *shuāng sī*, which means ‘two filaments’, referring to the filiform embolus and conductor; noun in apposition.

##### Diagnosis.

Males of *C. shuangsi* sp. nov. resemble those of *C. biembolata* (Deeleman-Reinhold 2001: 132, figs 67–69) in having a similar filiform embolus and conductor but differ by the retrolateral tibial apophysis with a relatively shorter tip, blunt, without inner apophysis (Figs 50B, 51E, 62D, 72D) (vs. tibial apophysis with long, acuminate tip, accompanied by a small inner apophysis; Deeleman-Reinhold 2001: figs 67, 68); and both the embolus and conductor shorter than the tegulum width (Figs 50C–E, 62D) (vs. embolus and conductor longer than tegulum width; Deeleman-Reinhold 2001: fig. 69). Females also resemble those of *C. biembolata* in having a small and rebordered atrium but can be recognised by the atrium-shaped nearly like an inverted triangle (Figs 51A, B, 80D, 88D) (vs. round; Deeleman-Reinhold 2001: fig. 70) and the bean-shaped spermathecae smaller than bursae (Figs 51C, D, 96D) (vs. tubular spermathecae with convoluted distal part, larger than bursae; Deeleman-Reinhold 2001: fig. 71).

##### Remarks.

*C. shuangsi* sp. nov. resembles *C. biembolata* which was first described and assigned to the *C. japonica* group (called *C. filicata* group in the present paper) by Deeleman-Reinhold (2001) because of the characteristic copulatory organs (for a detailed diagnosis, see above). However, both species lack the dark pattern found on the dorsum of the opisthosoma in all existing members of the *filicata* group. Thus, there remains considerable uncertainty about placing the two species in the *filicata* group.

##### Description.

**Male.** Holotype (Fig. 51F, G): Carapace 1.90 long, 1.36 wide. Carapace uniformly brown, without distinct pattern; ocular area distinctly narrowed, cervical groove and radial grooves indistinctly visible; tegument smooth, marginally clothed with short setae. Eyes: AER slightly recurved, PER slightly wider than AER, almost straight in dorsal view. Eye sizes and interdistances: AME 0.09, ALE 0.14, PME 0.11, PLE 0.09, AME–AME 0.07, AME–ALE 0.04, PME–PME 0.17, PME–PLE 0.09, MOQL 0.29, MOQA 0.25, MOQP 0.42. Chelicerae robust and brown, with four promarginal and three retromarginal teeth. Sternum pale brown, 1.07 long, 0.69 wide. Labium and endites coloured as carapace. Legs light brown, without distinct markings. Leg measurements: I missing, II 4.81 (1.46, 1.92, 0.98, 0.46), III 3.86 (1.18, 1.25, 1.04, 0.42), IV 5.53 (1.53, 1.87, 1.63, 0.50). Abdomen missing.

Palp (Figs 50A–E, 51E, 62D, 72D). Tibia short, ca. 2 × shorter than cymbium, bearing group of ventral setae; RTA large, ca. as long as tibia, proximally broad and heavily sclerotised, distally thinner and partly membranous, tip blunt. Bulb oval, 1.9 × longer than wide; sperm duct distinct and sinuate, forming a double loop. Embolus filiform, arising at ca. 11 o’clock position, broad at base, gradually tapering toward apex, embolar tip pointing distally. Conductor originating from anterior membranous portion of tegulum, consisting of broad base and filiform distal part, base partly membranous and covering embolar apex, apex sharp and pointing prolatero-proximally.

**Female.** Paratype (Fig. 51H, I). Total length 4.07; carapace 1.82 long, 1.37 wide; opisthosoma 2.25 long, 1.40 wide. Eye sizes and interdistances: AME 0.10, ALE 0.13, PME 0.10, PLE 0.11, AME–AME 0.08, AME–ALE 0.07, PME–PME 0.23, PME–PLE 0.15, MOQL 0.26, MOQA 0.23, MOQP 0.44. Chelicerae with three promarginal and two retromarginal teeth. Sternum 0.99 long, 0.67 wide. Leg measurements: I 3.60 (1.34, 1.28, 0.66, 0.42), II 3.91 (1.18, 1.57, 0.72, 0.45), III 3.24 (1.03, 1.08, 0.76, 0.37), IV 4.83 (1.34, 1.68, 1.38, 0.44). Colouration lighter than in male. Other characters as in male.

Epigyne (Figs 51A–D, 80D, 88D, 96D). Epigynal plate ca. 1.2 × wider than long, through which spermathecae and bursae are indistinctly apparent. Atrium small and shaped like an inverted triangle, with distinctly rebordered margin, ca. 1/3 epigyne length and width, anterior margin almost straight, posterior margin V-shaped. Copulatory openings located at anterolateral atrial borders. Copulatory ducts absent. Spermathecae bean-shaped, ca. 1.3 × longer than wide, separated by one diameter. Bursae ovoid, ca. 1.4 × longer than wide, close together, surface membranous and translucent, inside pigmented.

##### Distribution.

Known only from the type locality, Xishuangbanna, Yunnan, China.

#### Clubiona wangchengi

Taxon classificationAnimaliaAraneaeClubionidae

Yu & Li sp. nov.

41A46993-422D-5745-A54A-F1110A4DF41F

http://zoobank.org/4EB33FD0-CBCB-4642-9B22-78220A539678

**Figure 52.**
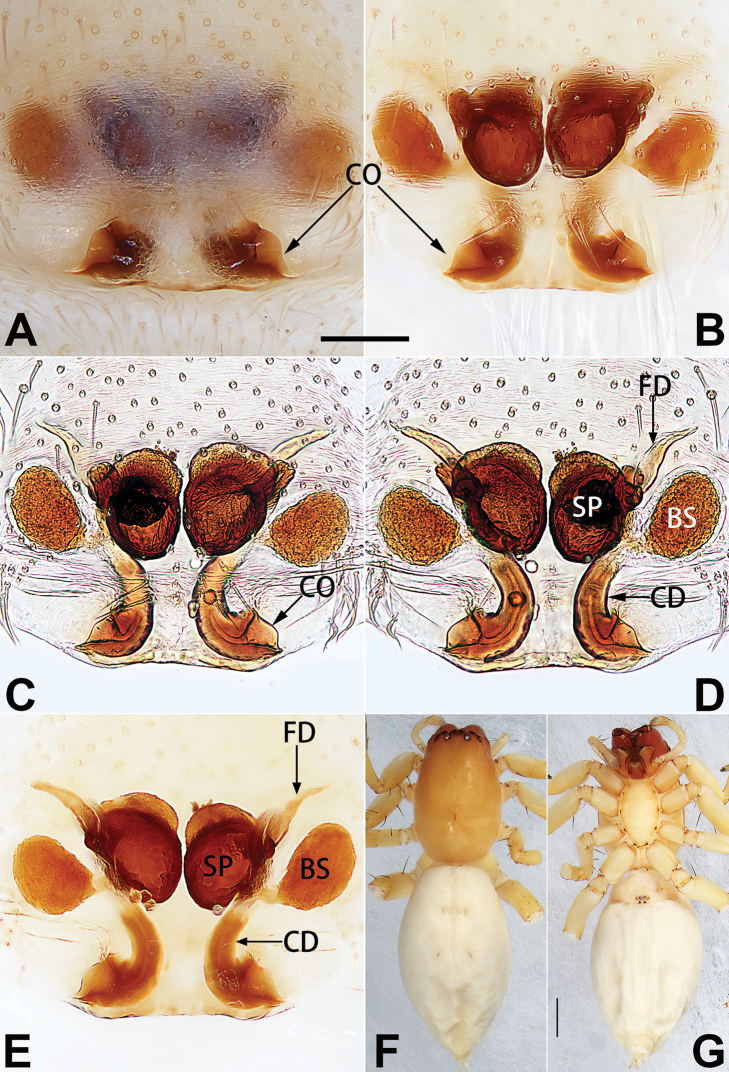
Holotype female of *Clubiona wangchengi* sp. nov., epigyne (**A–E**) and habitus (**F, G**) **A** intact, ventral view **B** cleared, ventral view **C** cleared, ventral view **D** cleared, dorsal view **E** cleared, dorsal view **F** dorsal view **G** ventral view. Abbreviations: BS = bursa; CD = copulatory duct; CO = copulatory opening; FD = fertilisation duct; SP = spermatheca. Scale bars: 0.1 mm (equal for **A–E**); 1 mm (equal for **F**, **G**).

**Figure 53.**
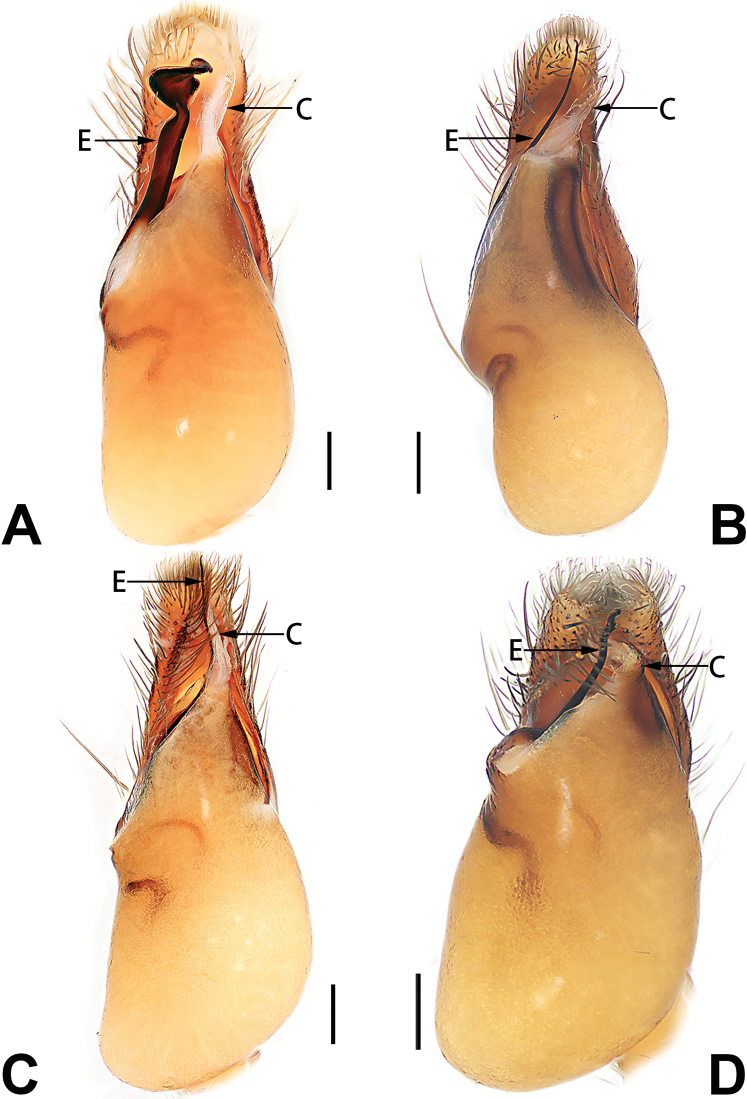
*Clubiona* spp. of the *C. corticalis* group, male palp, ventral view **A***C. cochlearis***B***C. rama***C***C. subrama***D***C. zhigangi* sp. nov., holotype. Abbreviations: C = conductor; E = embolus. Scale bars: 0.2 mm.

**Figure 54.**
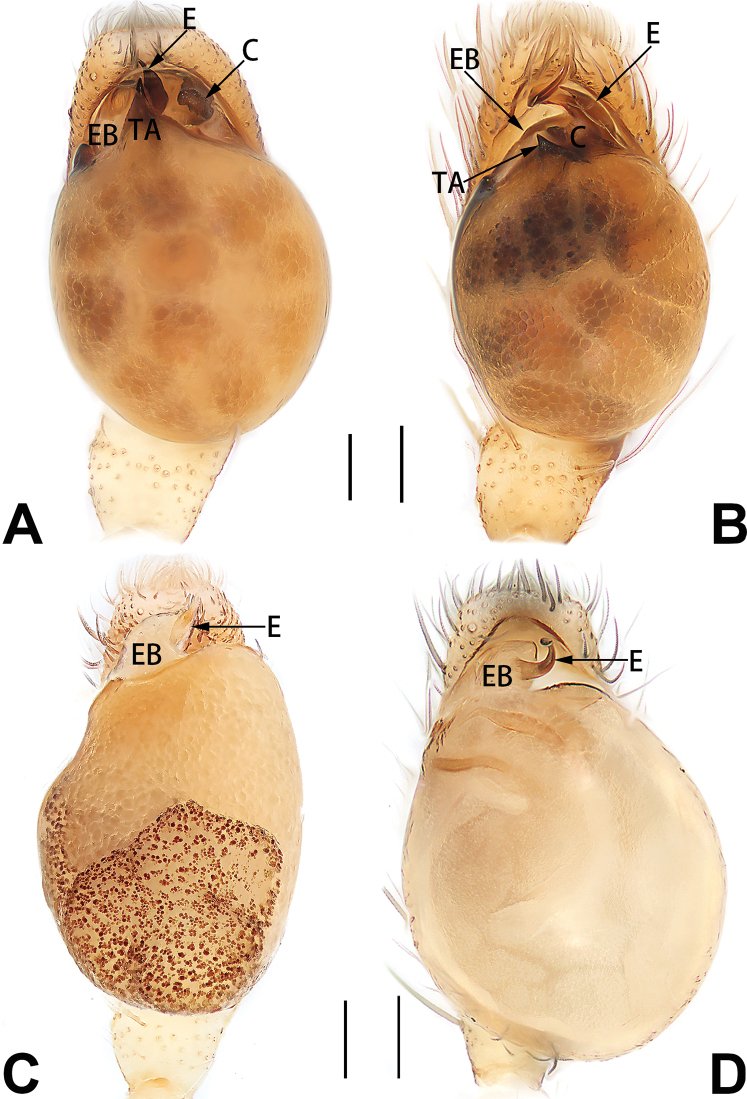
*Clubiona* spp. of the *C. corticalis* group, male palp, ventral view **A***C. didentata***B***C. kai***C***C. subyaginumai***D***C. tiane*. Abbreviations: C = conductor; E = embolus; EB = embolar base; TA = tegular apophysis. Scale bars: 0.1 mm.

**Figure 55.**
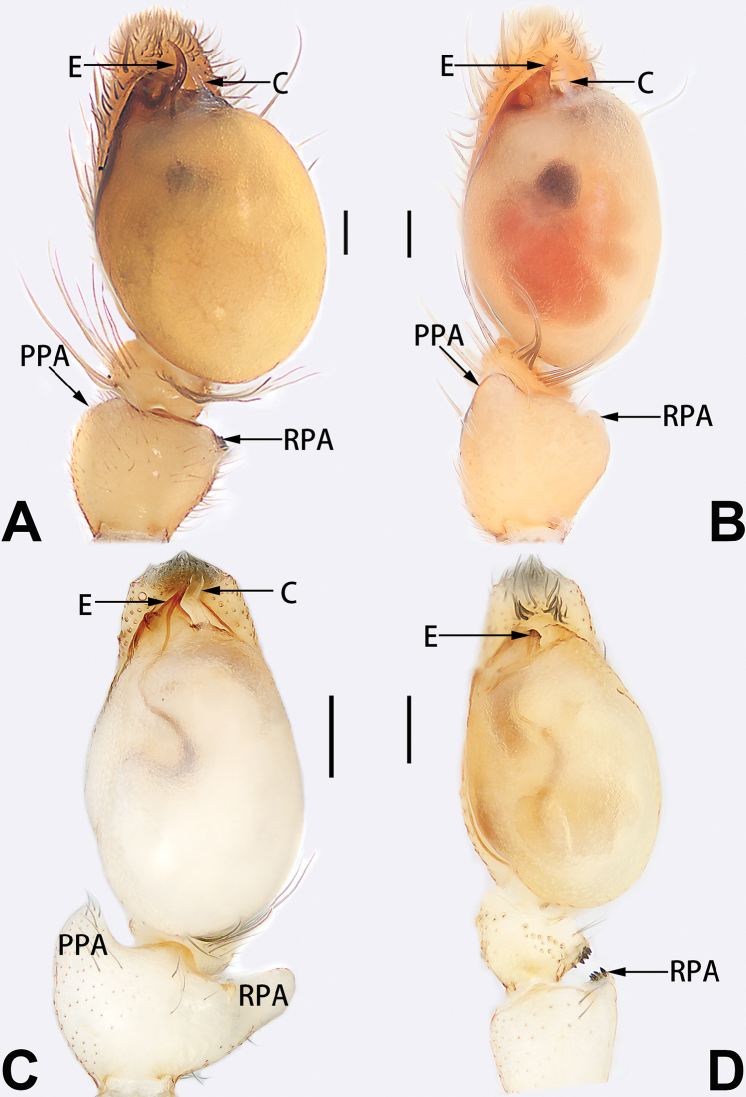
*Clubiona* spp. of the *C. corticalis* group, male palp, ventral view **A***C. moralis***B***C. submoralis***C***C. parconcinna***D***C. xiaoci* sp. nov., holotype. Abbreviations: C = conductor; E = embolus; PPA = prolateral patellar apophysis; RPA = retrolateral patellar apophysis. Scale bars: 0.1 mm.

**Figure 56.**
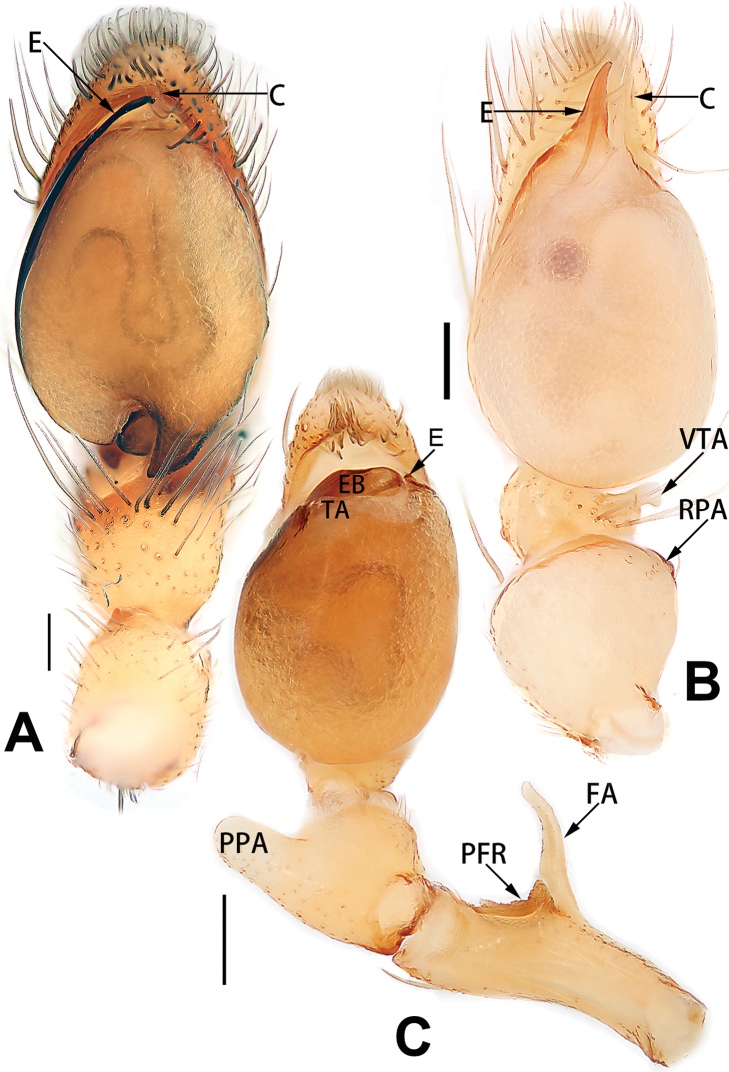
*Clubiona* spp. of the *C. corticalis* group, male palp, ventral view **A***C. kurosawai***B***C. multidentata***C***C. pollicaris*. Abbreviations: C = conductor; E = embolus; EB = embolar base; FA = femoral apophysis; PPA = prolateral patellar apophysis; RPA = retrolateral patellar apophysis; PFR = prolateral femoral ridge; TA = tegular apophysis; VTA, ventral tibial apophysis. Scale bars: 0.1 mm.

**Figure 57.**
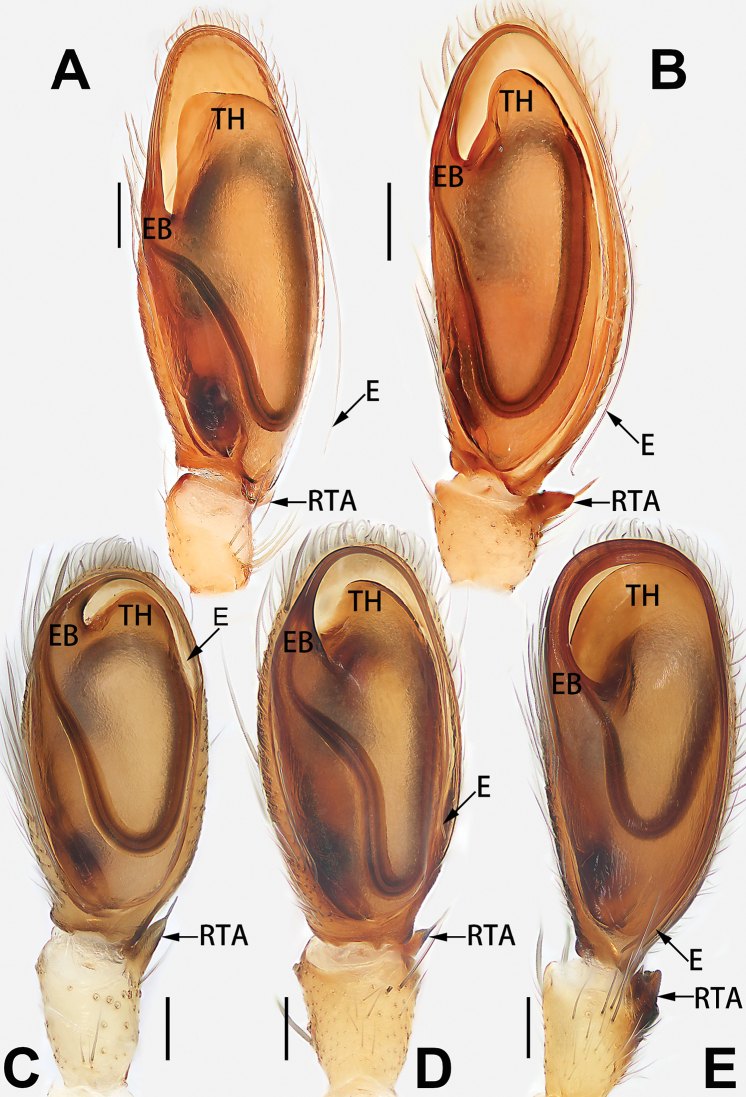
*Clubiona* spp. of the *C. ternatensis* group, male palp, ventral view **A***C. theoblicki***B***C. tongi***C***C. subkuu***D***C. subtongi* sp. nov., holotype **E***C. zhengi*. Abbreviations: E = embolus; EB = embolar base; RTA = retrolateral tibial apophysis; TH = tegular hump. Scale bars: 0.1 mm.

**Figure 58.**
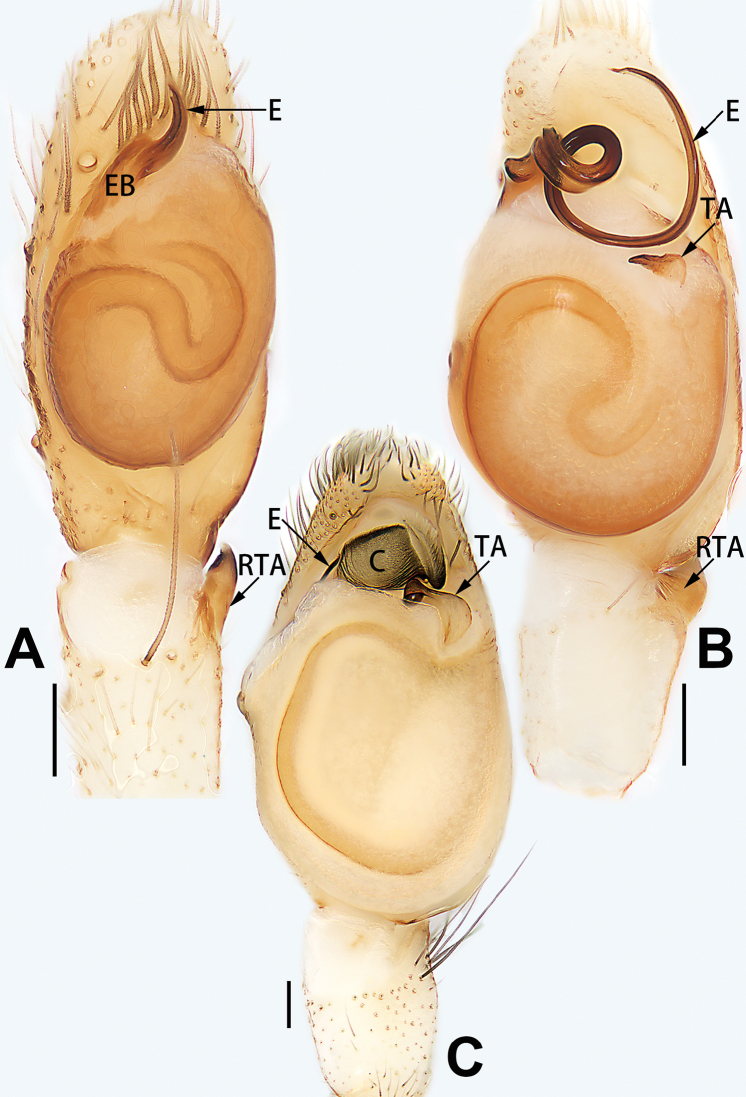
*Clubiona* spp. of the *C. filicata* group, male palp, ventral view **A***C. reichlini***B***C. filicata***C***C. banna* sp. nov., holotype. Abbreviations: C = conductor; E = embolus; EB = embolar base; TA = tegular apophysis; RTA = retrolateral tibial apophysis. Scale bars: 0.1 mm.

**Figure 59.**
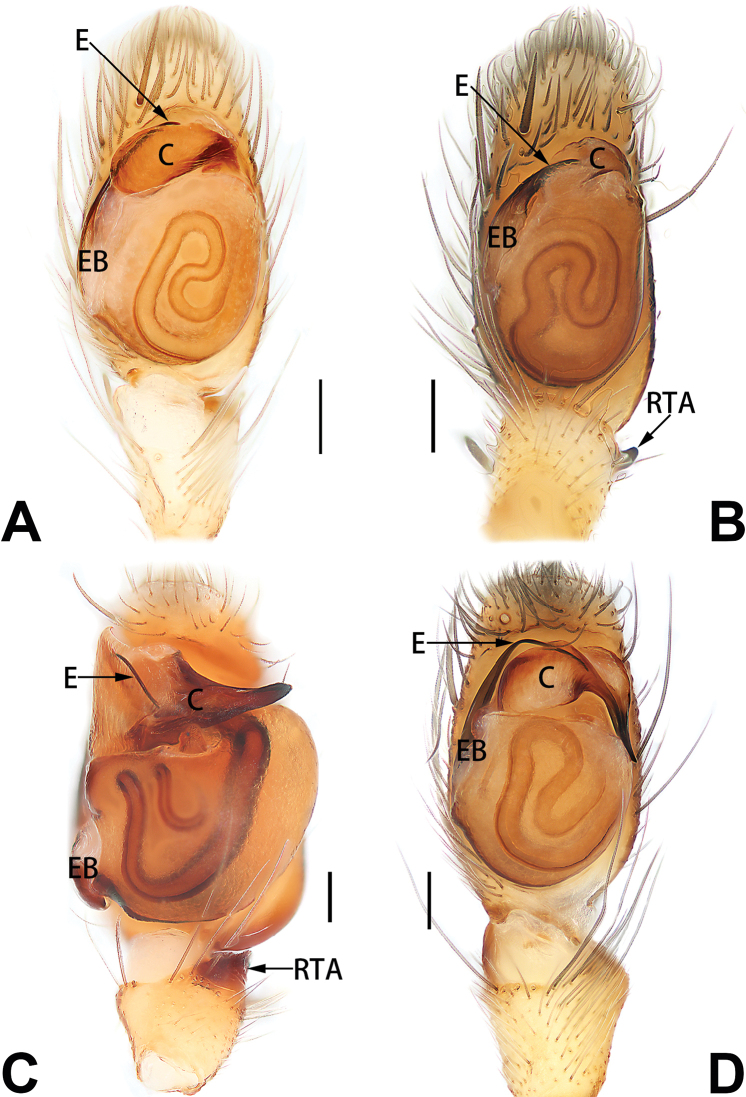
*Clubiona* spp. of the *C. filicata* group, male palp, ventral view **A***C. melanosticta***B***C. zhanggureni***C***C. circulata***D***C. suthepica*. Abbreviations: C = conductor; E = embolus; EB = embolar base; RTA = retrolateral tibial apophysis. Scale bars: 0.1 mm.

**Figure 60.**
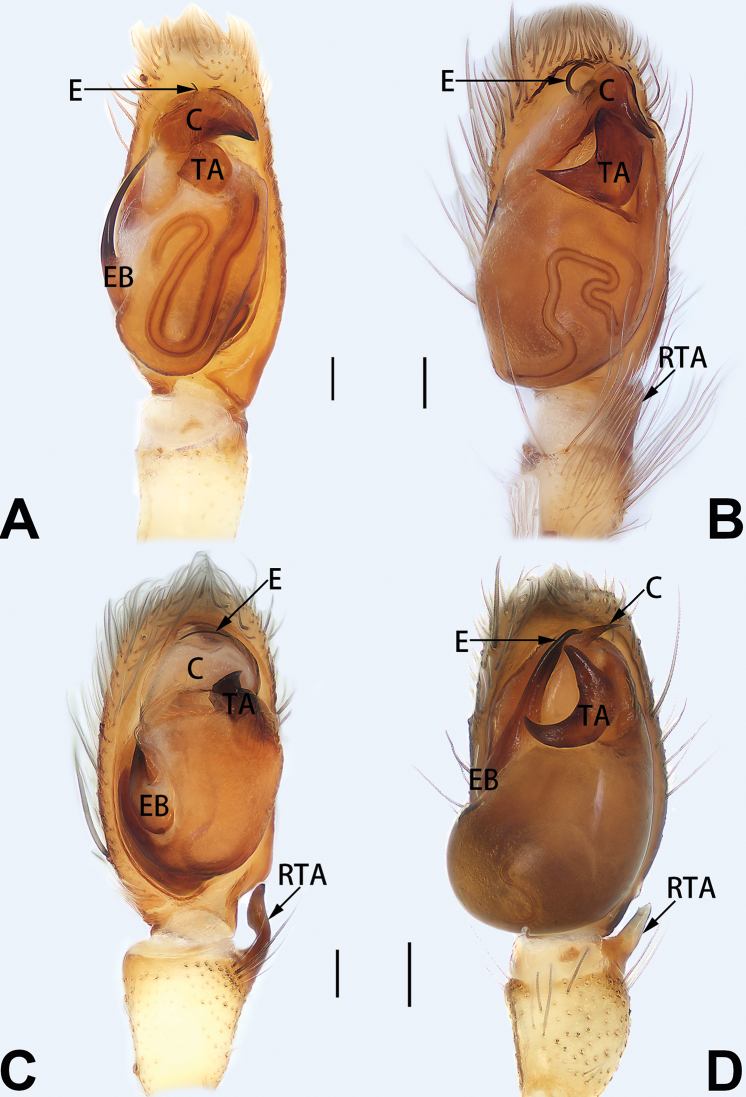
*Clubiona* spp. of the *C. filicata* group, male palp, ventral view **A***C. grucollaris***B***C. lala***C***C. abnormis***D***C. yueya*. Abbreviations: C = conductor; E = embolus; EB = embolar base; TA = tegular apophysis; RTA = retrolateral tibial apophysis. Scale bars: 0.1 mm.

**Figure 61.**
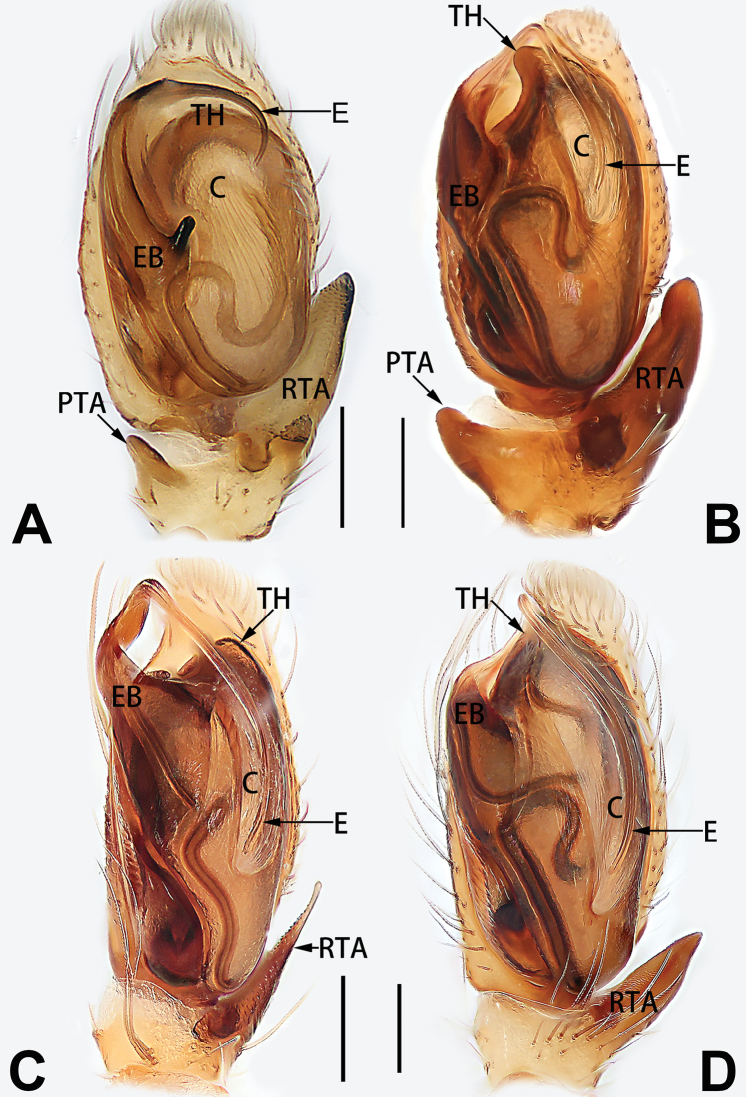
*Clubiona* spp. of the *C. trivialis* group, male palp, ventral view **A***C. bicornis***B***C. cheni***C***C. subasrevida***D***C. subquebecana*. Abbreviations: C = conductor; E = embolus; EB = embolar base; TH = tegular hump; PTA = prolateral tibial apophysis; RTA = retrolateral tibial apophysis. Scale bars: 0.1 mm.

**Figure 62.**
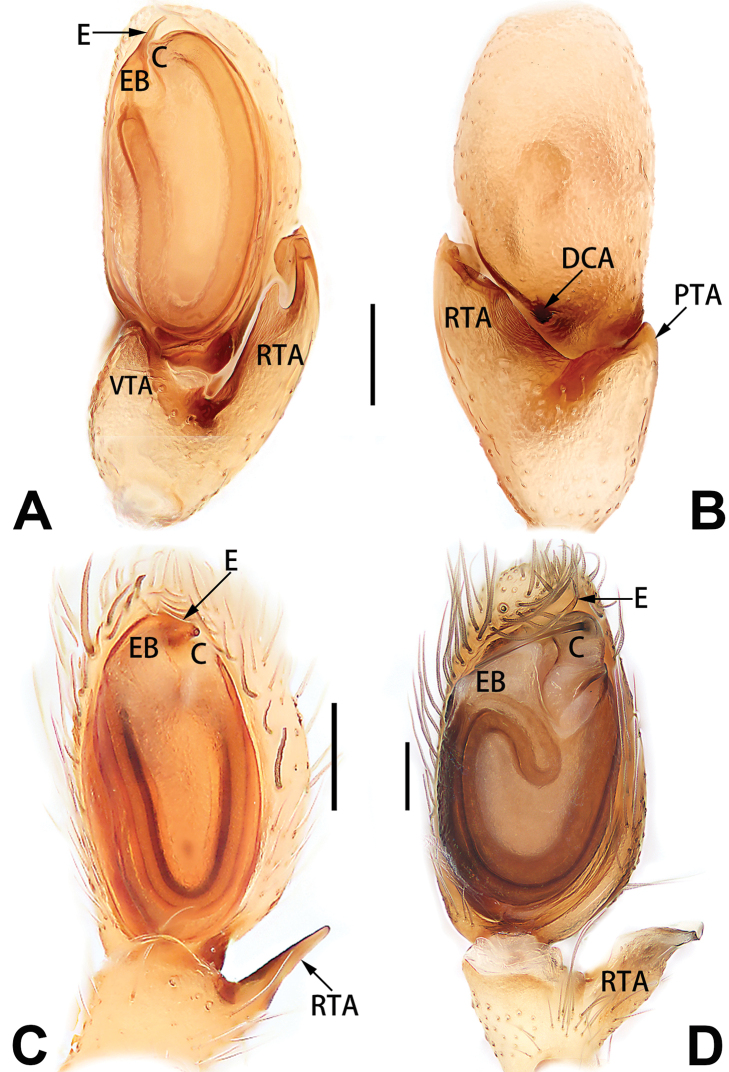
*Clubiona* spp., male palp, ventral view (**A, C, D**) and dorsal view (**B**) **A, B***C. yaoi***C***C. jiandan***D***C. shuangsi* sp. nov., holotype. Abbreviations: C = conductor; DCA = dorsal cymbial apophysis; E = embolus; EB = embolar base; PTA = prolateral tibial apophysis; RTA = retrolateral tibial apophysis; VTA = ventral tibial apophysis. Scale bars: 0.1 mm.

**Figure 63.**
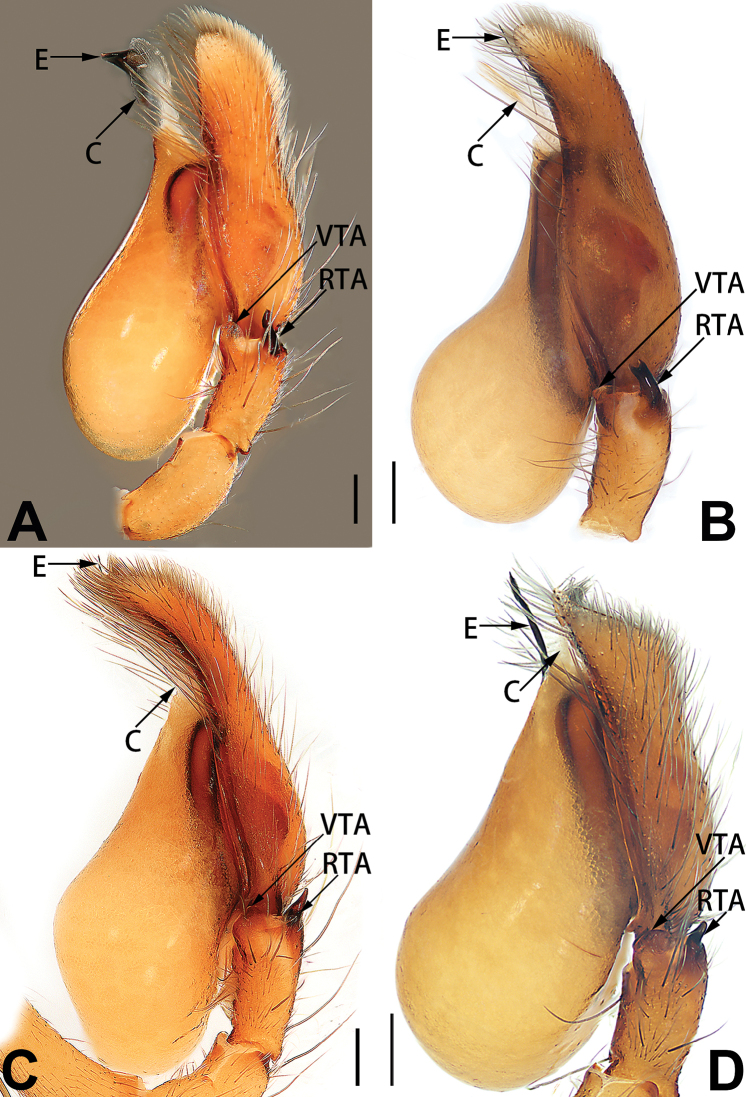
*Clubiona* spp. of the *C. corticalis* group, male palp, retrolateral view **A***C. cochlearis***B***C. rama***C***C. subrama***D***C. zhigangi* sp. nov., holotype. Abbreviations: C = conductor; E = embolus; RTA = retrolateral tibial apophysis; VTA = ventral tibial apophysis. Scale bars: 0.2 mm.

**Figure 64.**
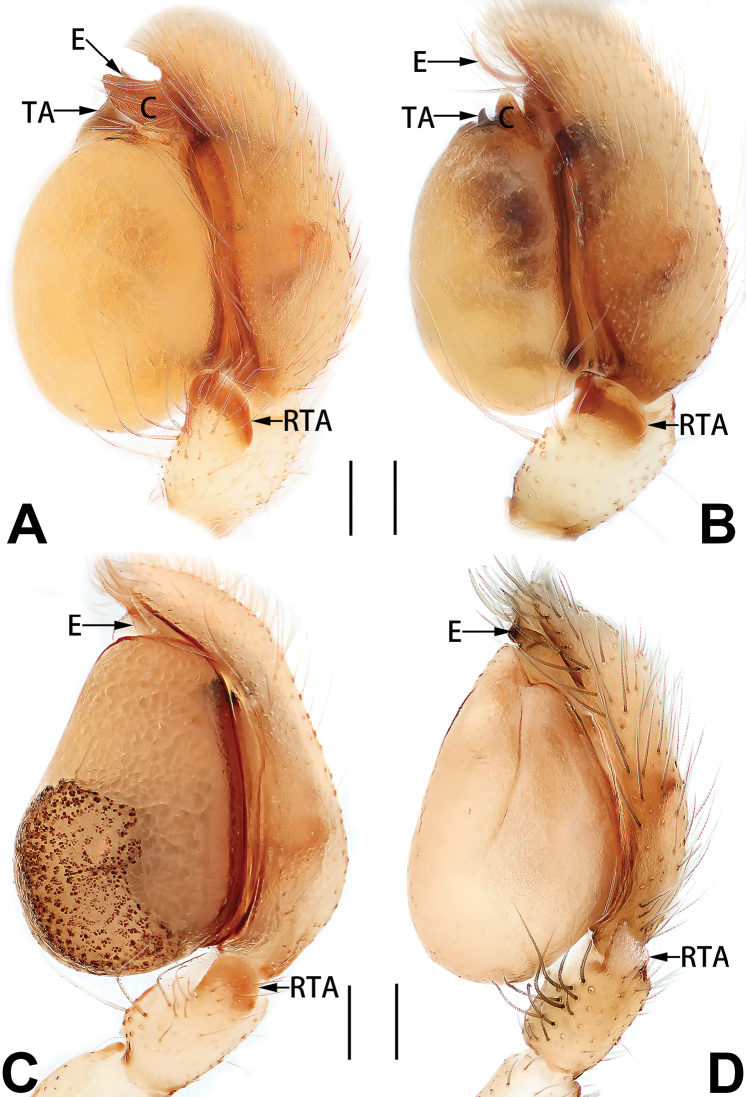
*Clubiona* spp. of the *C. corticalis* group, male palp, retrolateral view **A***C. didentata***B***C. kai***C***C. subyaginumai***D***C. tiane*. Abbreviations: C = conductor; E = embolus; TA = tegular apophysis; RTA = retrolateral tibial apophysis. Scale bars: 0.1 mm.

**Figure 65.**
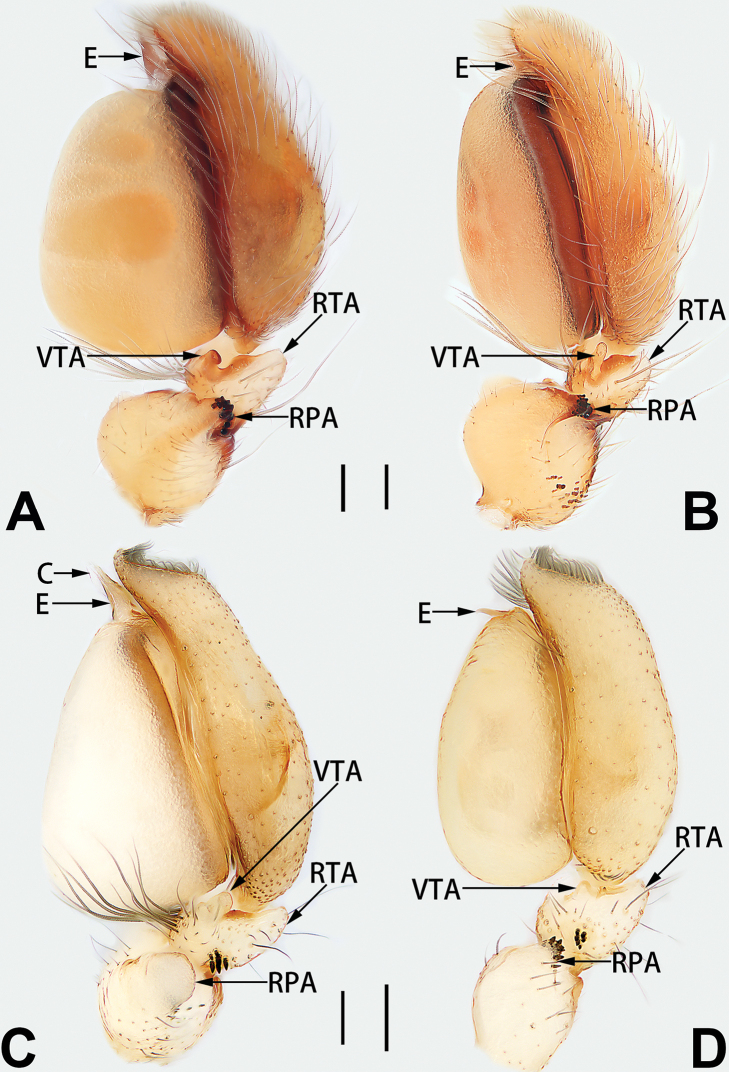
*Clubiona* spp. of the *C. corticalis* group, male palp, retrolateral view **A***C. moralis***B***C. submoralis***C***C. parconcinna***D***C. xiaoci* sp. nov., holotype. Abbreviations: C = conductor; E = embolus; RPA = retrolateral patellar apophysis; RTA, retrolateral tibial apophysis; VTA, ventral tibial apophysis. Scale bars: 0.1 mm.

**Figure 66.**
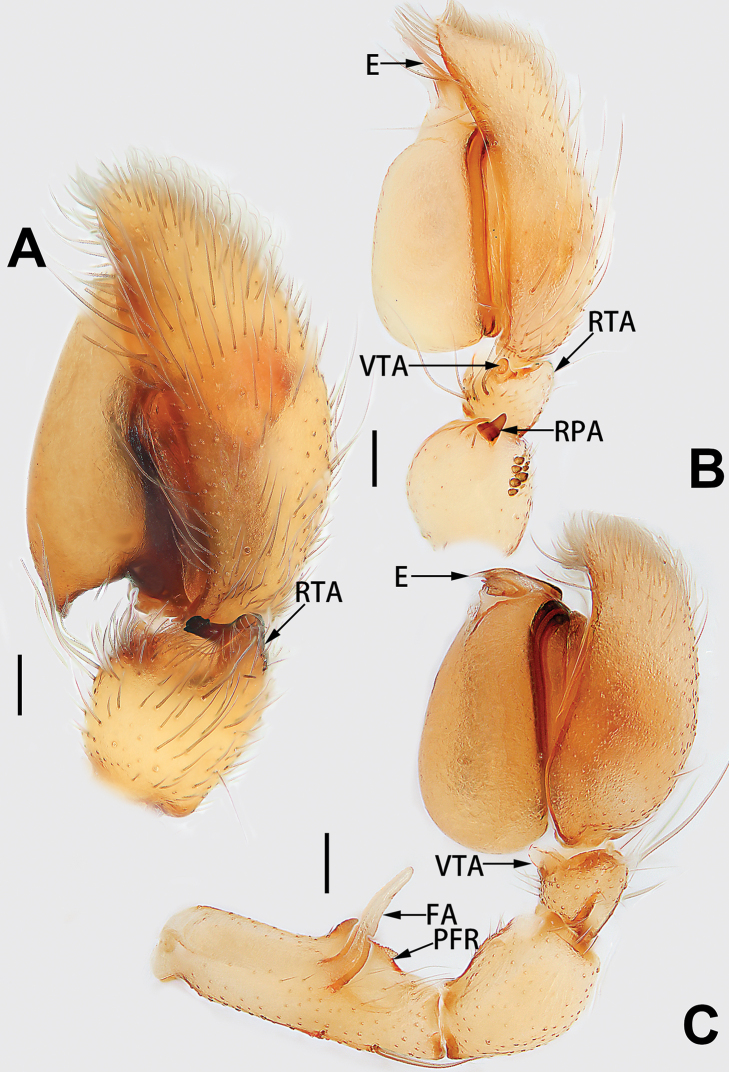
*Clubiona* spp. of the *C. corticalis* group, male palp, retrolateral view **A***C. kurosawai***B***C. multidentata***C***C. pollicaris*. Abbreviations: E = embolus; FA = femoral apophysis; PFR = prolateral femoral ridge; RPA = retrolateral patellar apophysis; RTA = retrolateral tibial apophysis; VTA = ventral tibial apophysis. Scale bars: 0.1 mm.

**Figure 67.**
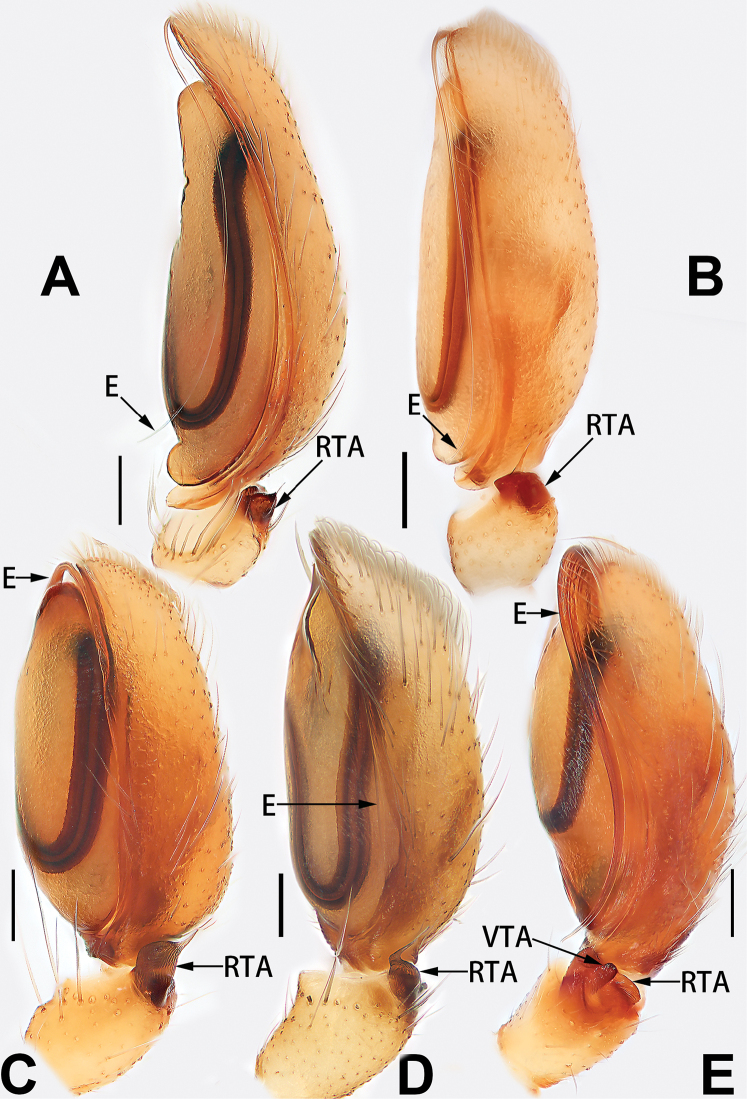
*Clubiona* spp. of the *C. ternatensis* group, male palp, retrolateral view **A***C. theoblicki***B***C. tongi***C***C. subkuu***D***C. subtongi* sp. nov., holotype **E***C. zhengi*. Abbreviations: E = embolus; RTA = retrolateral tibial apophysis; VTA = ventral tibial apophysis. Scale bars: 0.1 mm.

**Figure 68.**
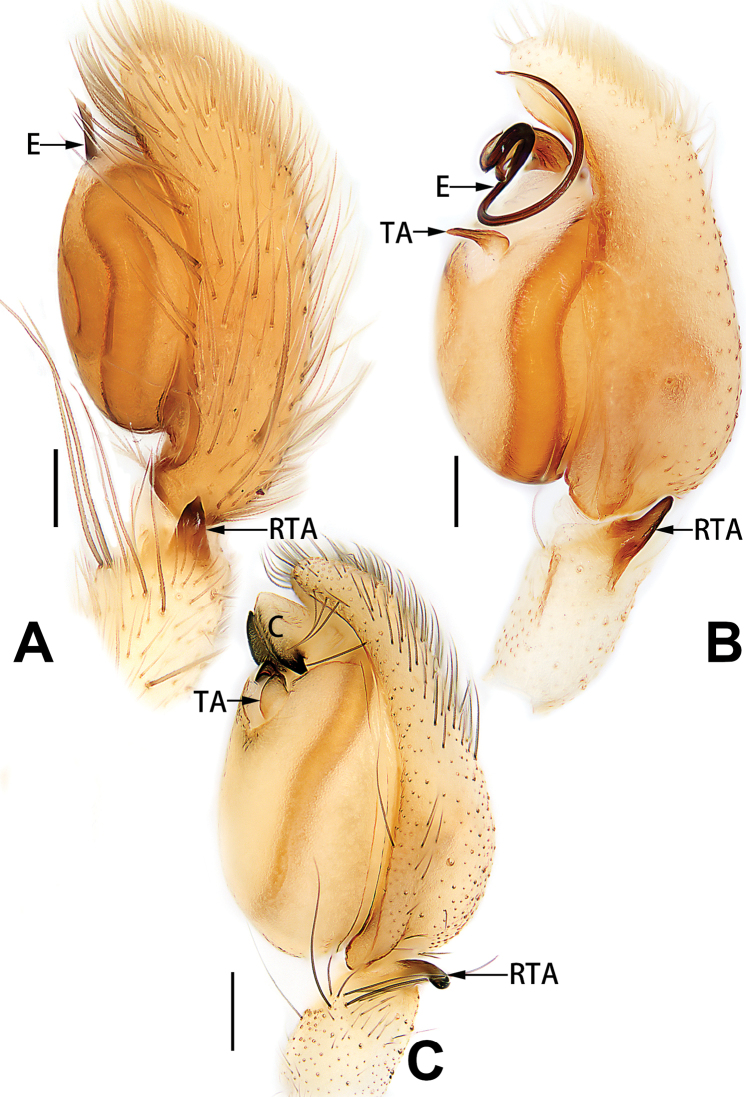
*Clubiona* spp. of the *C. filicata* group, male palp, retrolateral view **A***C. reichlini***B***C. filicata***C***C. banna* sp. nov., holotype. Abbreviations: C = conductor; E = embolus; TA = tegular apophysis; RTA = retrolateral tibial apophysis. Scale bars: 0.1 mm.

**Figure 69.**
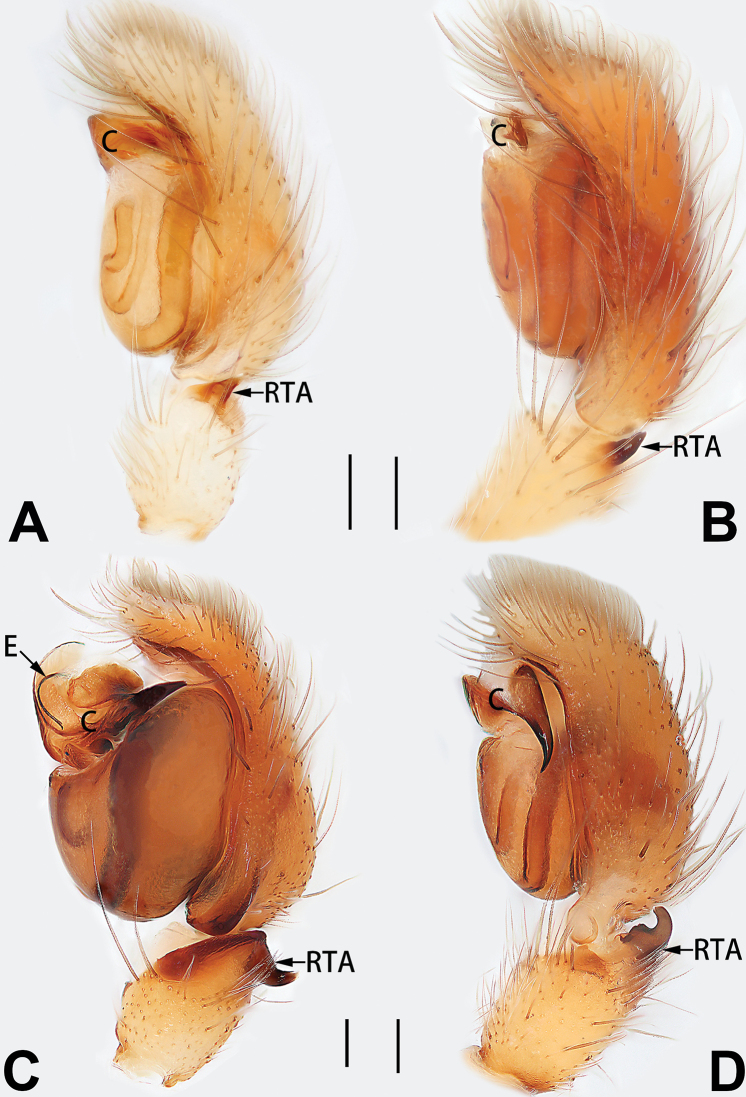
*Clubiona* spp. of the *C. filicata* group, male palp, retrolateral view **A***C. melanosticta***B***C. zhanggureni***C***C. circulata***D***C. suthepica*. Abbreviations: C = conductor; E = embolus; RTA = retrolateral tibial apophysis. Scale bars: 0.1 mm.

**Figure 70.**
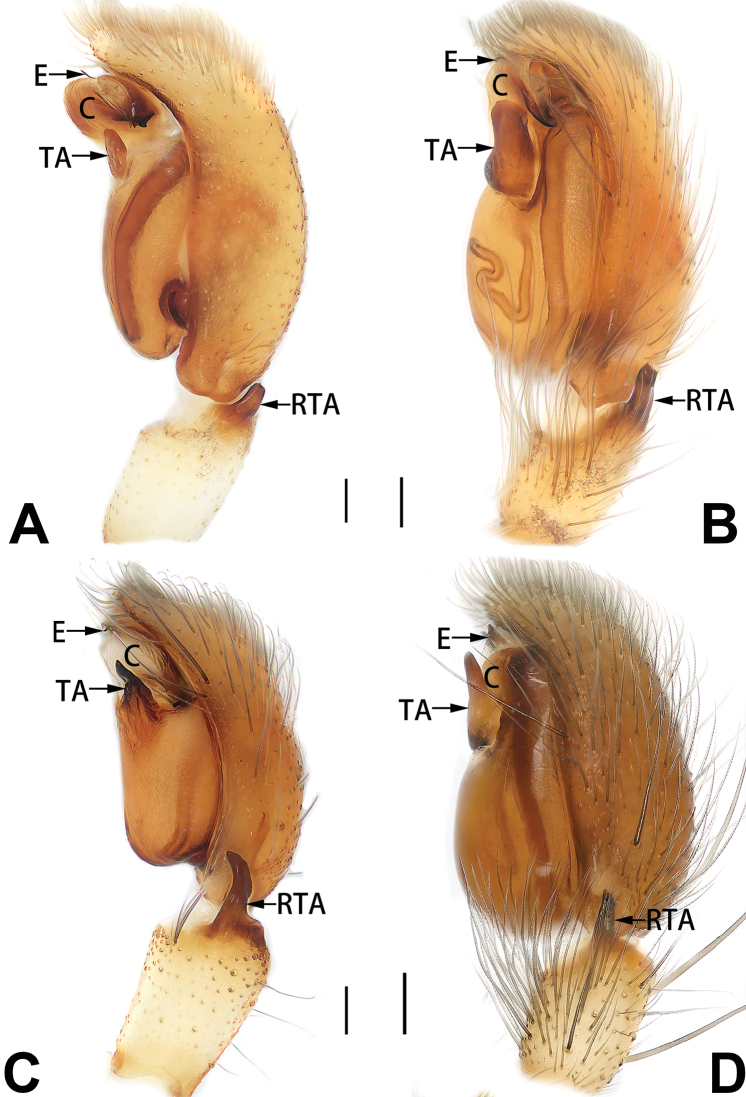
*Clubiona* spp. of the *C. filicata* group, male palp, retrolateral view **A***C. grucollaris***B***C. lala***C***C. abnormis***D***C. yueya*. Abbreviations: C = conductor; E = embolus; TA = tegular apophysis; RTA = retrolateral tibial apophysis. Scale bars: 0.1 mm.

**Figure 71.**
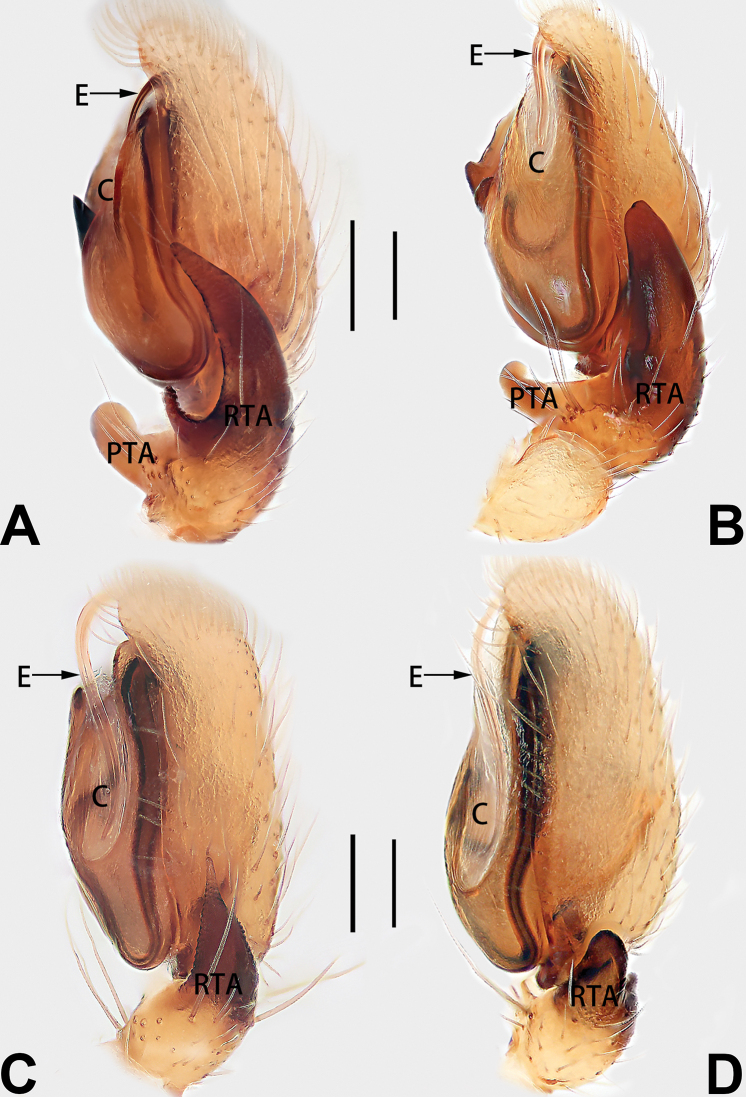
*Clubiona* spp. of the *C. trivialis* group, male palp, retrolateral view **A***C. bicornis***B***C. cheni***C***C. subasrevida***D***C. subquebecana*. Abbreviations: C = conductor; E = embolus; PTA = prolateral tibial apophysis; RTA = retrolateral tibial apophysis. Scale bars: 0.1 mm.

**Figure 72.**
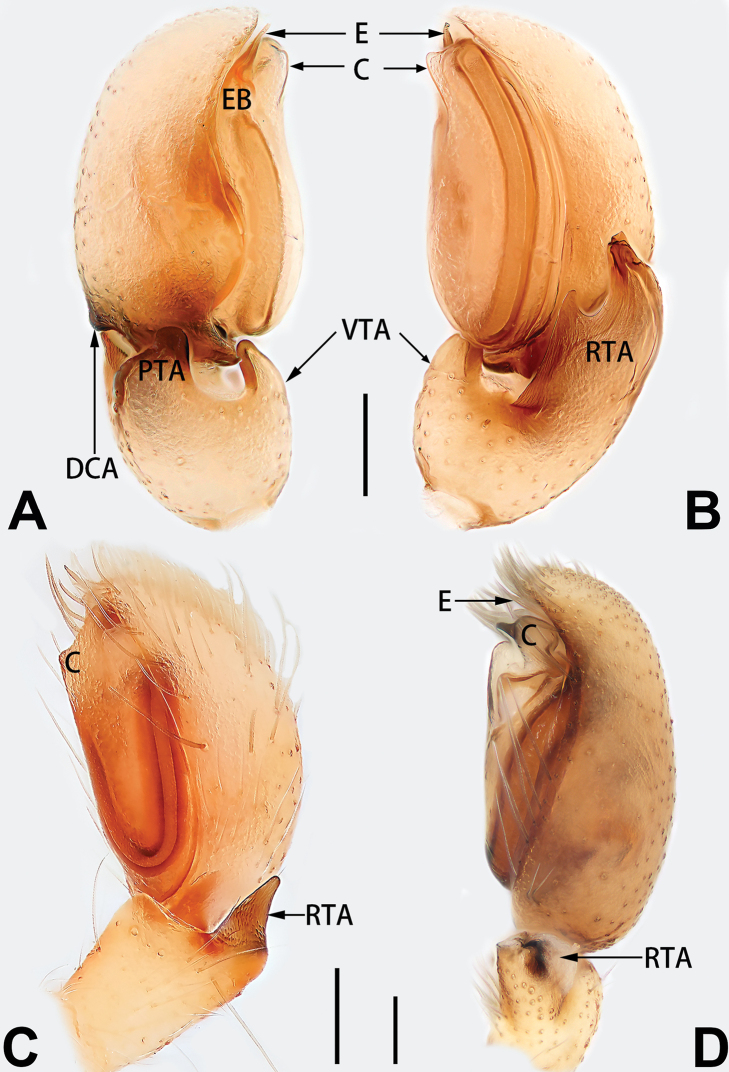
*Clubiona* spp., male palp, prolateral view (**A**) and retrolateral view (**B–D**) **A, B***C. yaoi***C***C. jiandan***D***C. shuangsi* sp. nov., holotype. Abbreviations: C = conductor; DCA = dorsal cymbial apophysis; E = embolus; EB = embolar base; PTA = prolateral tibial apophysis; RTA = retrolateral tibial apophysis; VTA = ventral tibial apophysis. Scale bars: 0.1 mm.

**Figure 73.**
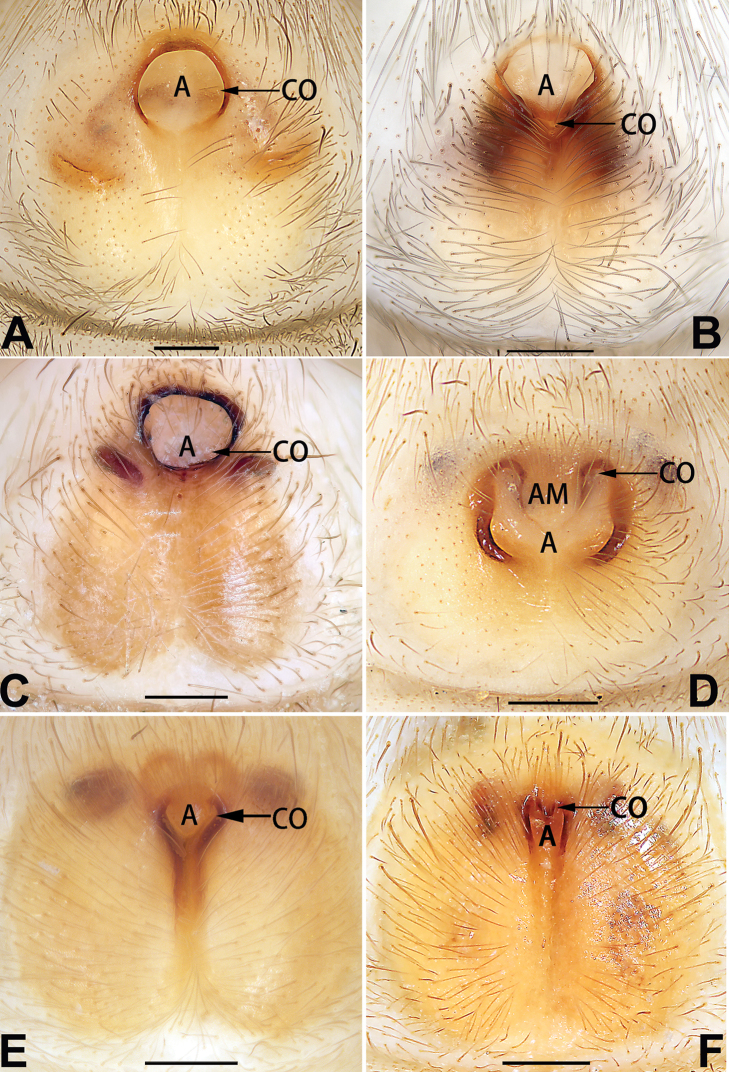
*Clubiona* spp. of the *C. corticalis* group, epigyne, intact, ventral view **A***C. cochlearis***B***C. dengpao* sp. nov., holotype **C***C. yejiei* sp. nov., holotype **D***C. tixing* sp. nov., holotype **E***C. subrama***F***C. zhigangi* sp. nov., paratype. Abbreviations: A = atrium; AM = atrial membrane; CO = copulatory opening. Scale bars: 0.2 mm.

**Figure 74.**
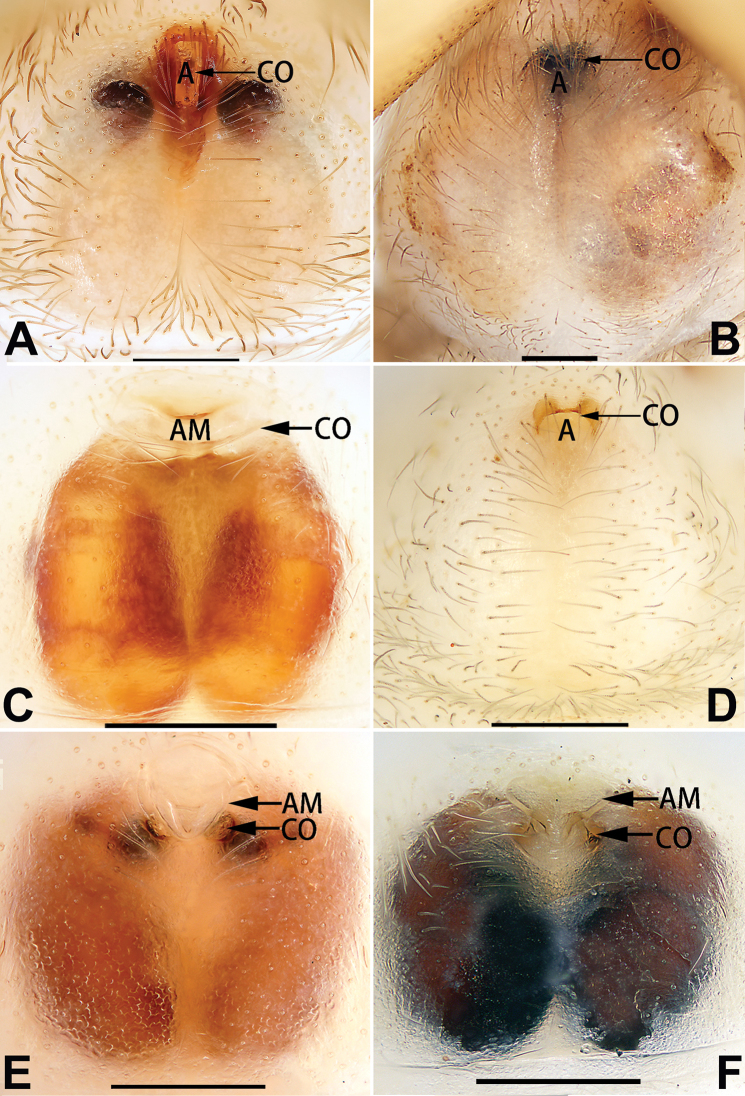
*Clubiona* spp. of the *C. corticalis* group, epigyne, intact, ventral view **A***C. xiaokong* sp. nov., holotype **B***C. zhaoi* sp. nov., holotype **C***C. kai***D***C. tiane***E***C. didentata***F***C. subdidentata* sp. nov., holotype. Abbreviations: A = atrium; AM = atrial membrane; CO = copulatory opening. Scale bars: 0.2 mm.

**Figure 75.**
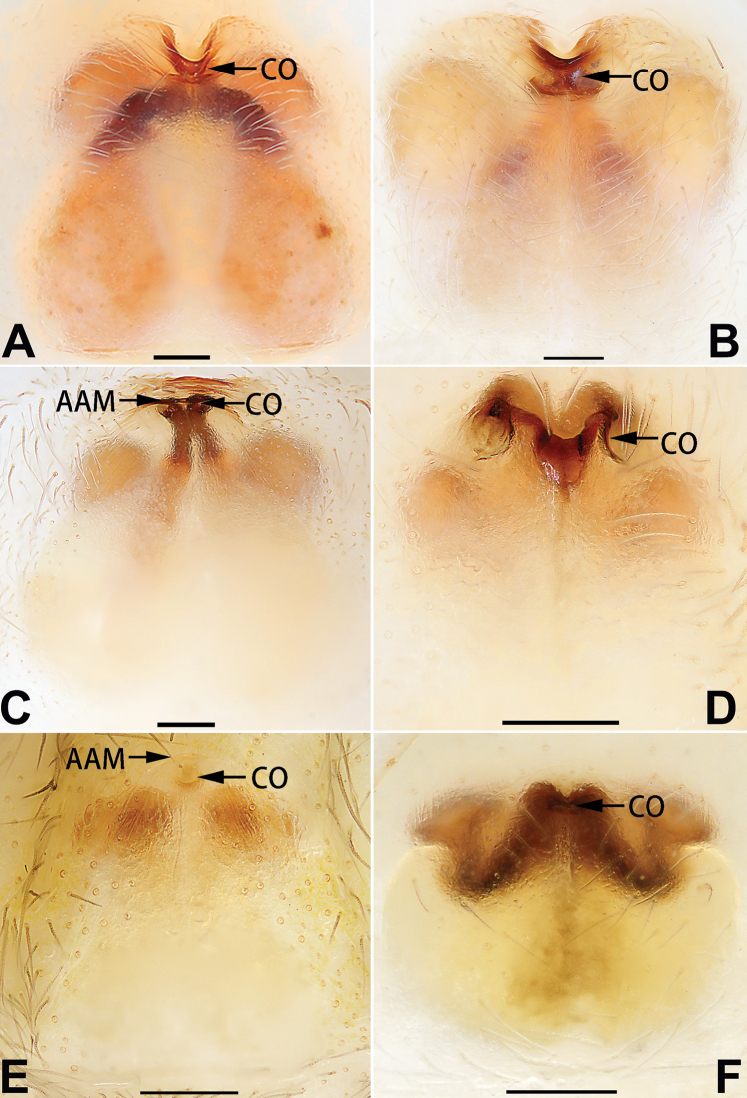
*Clubiona* spp. of the *C. corticalis* group, epigyne, intact, ventral view **A***C. moralis***B***C. submoralis***C***C. parconcinna***D***C. multidentata***E***C. xiaoci* sp. nov., paratype **F***C. subyaginumai*. Abbreviations: AAM = atrial anterior margin; CO = copulatory opening. Scale bars: 0.1 mm.

**Figure 76.**
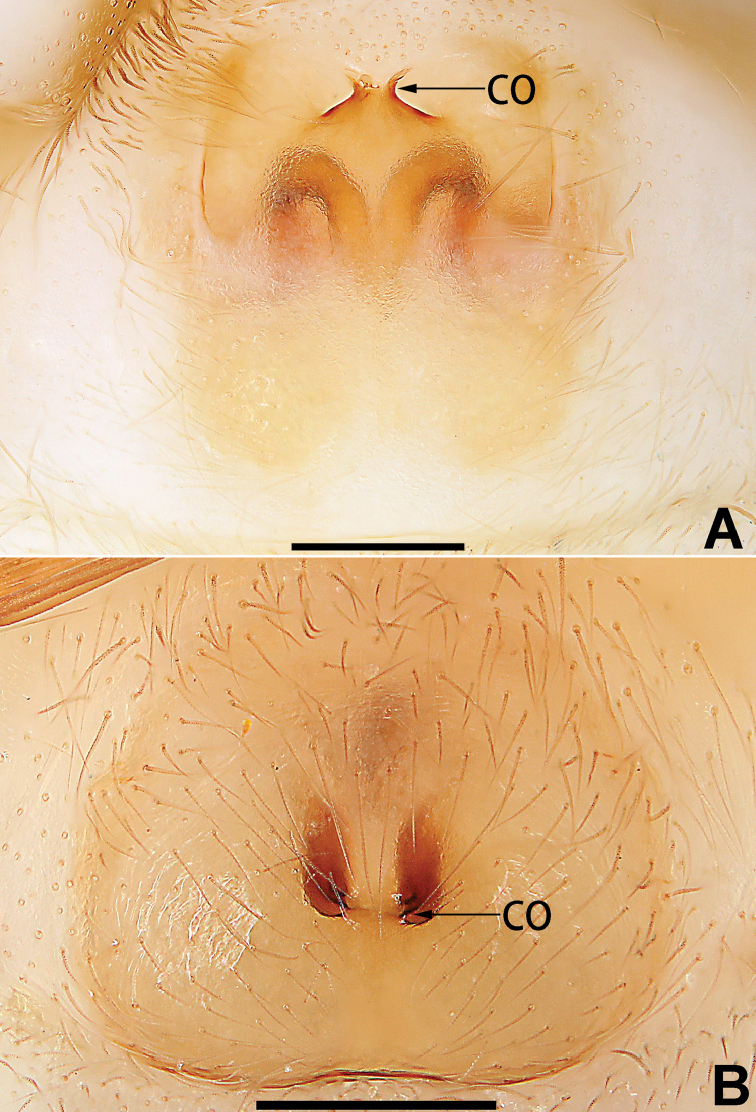
*Clubiona* spp. of the *C. corticalis* group, epigyne, intact, ventral view **A***C. kurosawai***B***C. pollicaris*. Abbreviations: CO = copulatory opening. Scale bars: 0.2 mm.

Figs 52 , 80C , 88C , 96C

##### Holotype.

♀ (IZCAS-Ar 34765), China: Yunnan Province: Xishuangbanna: Mengla County: Menglun Town: XTBG, 48^th^ km landmark in Menglun Nature Reserve, 21°58.704'N, 101°19.748'E, ca. 1088 m, 12.VIII.2011, G. Zheng et al. leg. ***Other material examined.*** 1♀ (YHCLU0087), China: Yunnan Province: Xishuangbanna: Mengla County: Menglun Town: Bubang Village, *Parashorea cathayensis* forest, 21°35.011'N, 101°35.013'E, ca. 680 m, 28.VII.2016, G. Zheng leg.

##### Etymology.

This species is named after Mr. Cheng Wang (Tongren City, China) who has helped us greatly with this research; noun (name) in genitive case.

##### Diagnosis.

**Figure 77.**
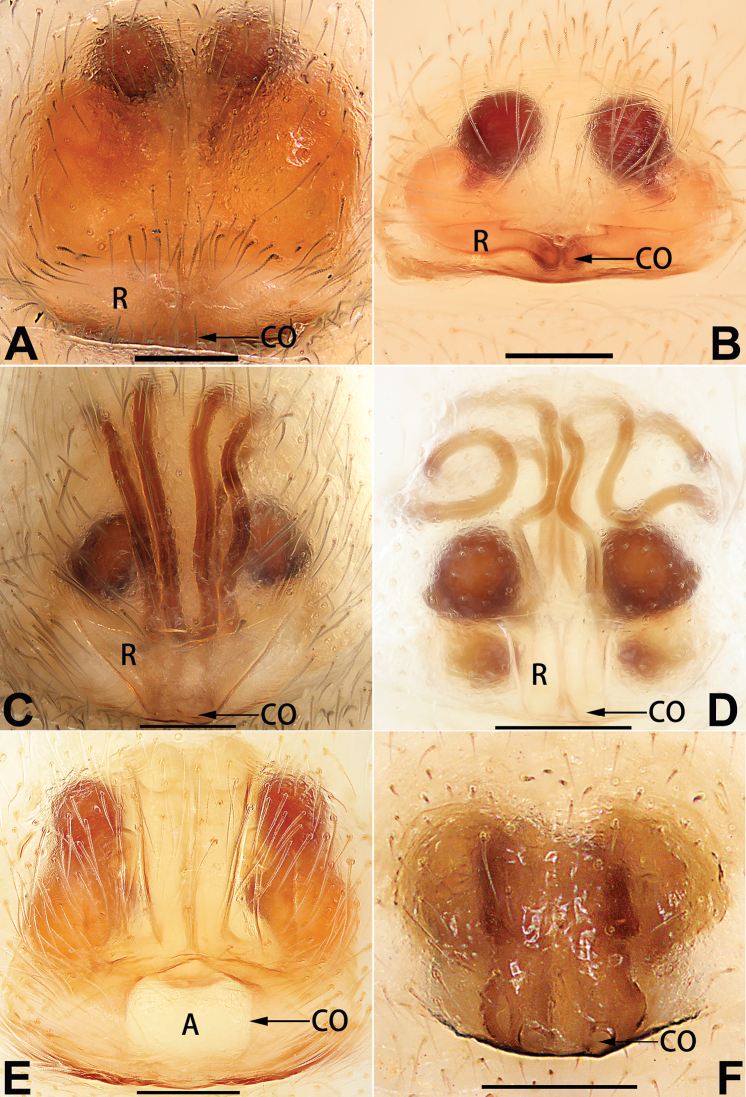
*Clubiona* spp. of the *C. ternatensis* group (**A–E**) and the *C. trivialis* group (**F**), epigyne, intact, ventral view **A***C. mii* sp. nov. holotype **B***C. subkuu***C***C. theoblicki***D***C. tongi***E***C. zhengi***F***C. bicornis*. Abbreviations: A = atrium; CO = copulatory opening; R = epigynal ridge. Scale bars: 0.1 mm.

**Figure 78.**
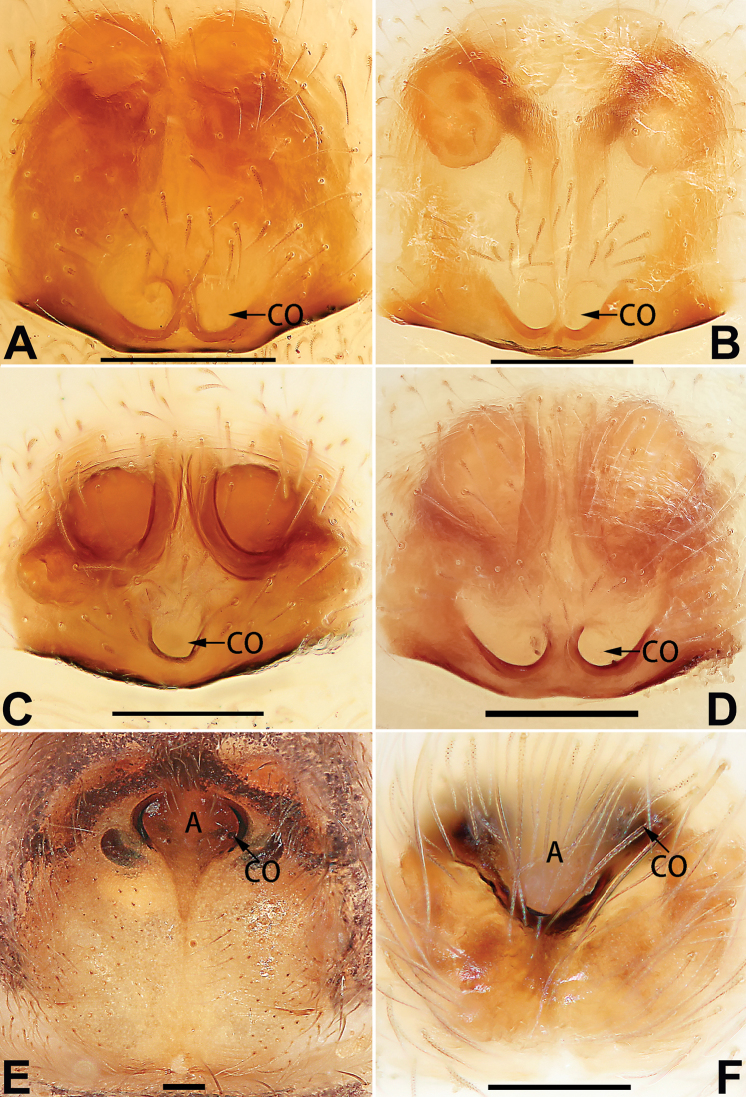
*Clubiona* spp. of the *C. trivialis* group (**A–D**) and the *C. filicata* group (**E, F**), epigyne, intact, ventral view **A***C. cheni***B***C. menglun* sp. nov., holotype **C***C. subasrevida***D***C. subquebecana***E***C. banna* sp. nov., paratype **F***C. melanosticta*. Abbreviations: A = atrium; CO = copulatory opening. Scale bars: 0.1 mm.

**Figure 79.**
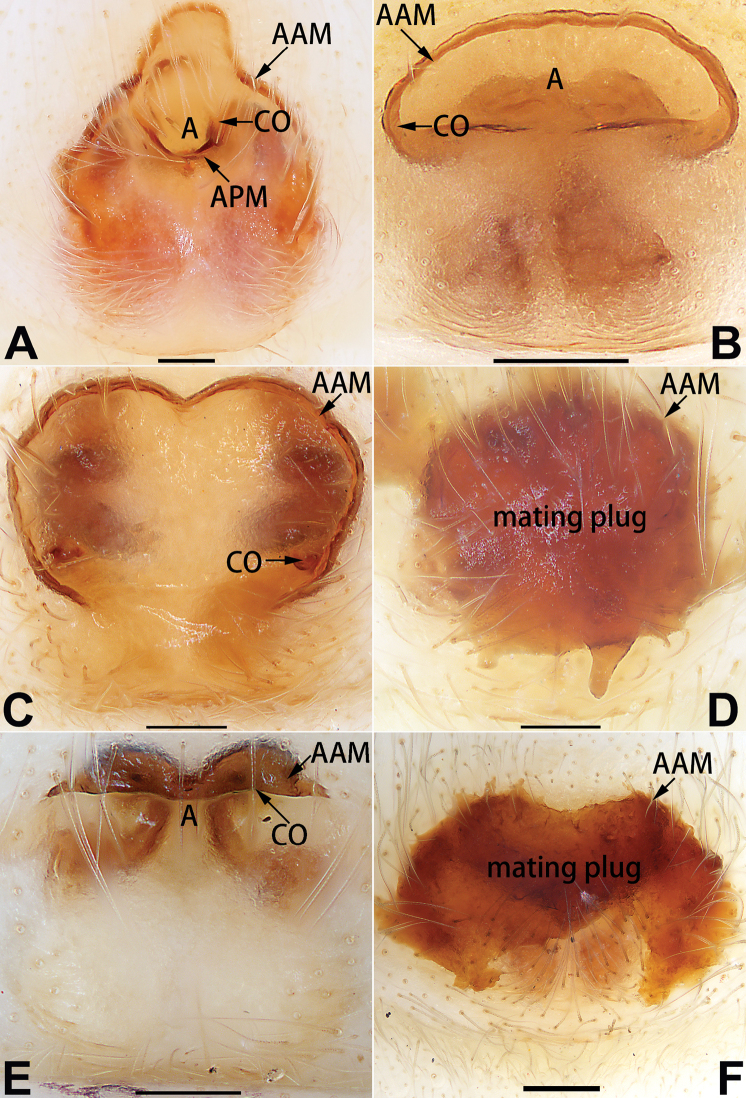
*Clubiona* spp. of the *C. filicata* group, epigyne, intact, ventral view **A***C. circulata***B***C. reichlini***C***C. grucollaris***D***C. lala***E***C. suthepica***F***C. yueya*. Abbreviations: A = atrium; AAM = atrial anterior margin; APM = atrial posterior margin; CO = copulatory opening. Scale bars: 0.1 mm.

**Figure 80.**
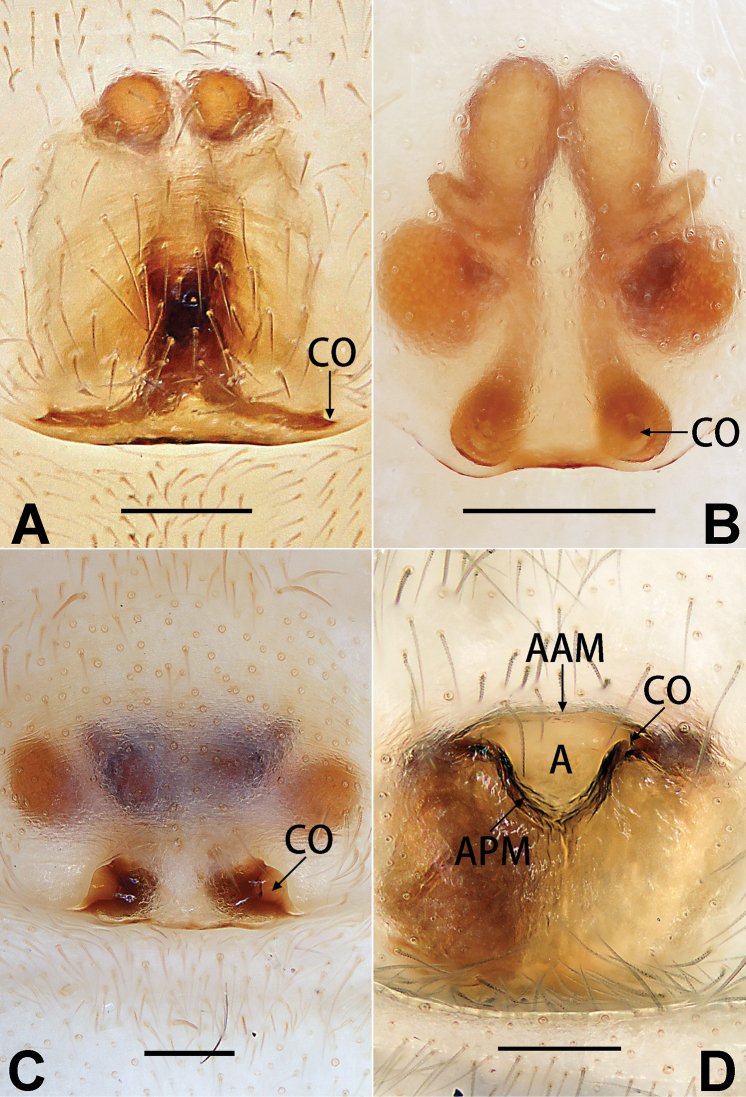
*Clubiona* spp., epigyne, intact, ventral view **A***C. yaoi***B***C. jiandan***C***C. wangchengi* sp. nov., holotype **D***C. shuangsi* sp. nov., paratype. Abbreviations: A = atrium; AAM = atrial anterior margin; APM = atrial posterior margin; CO = copulatory opening. Scale bars: 0.1 mm.

**Figure 81.**
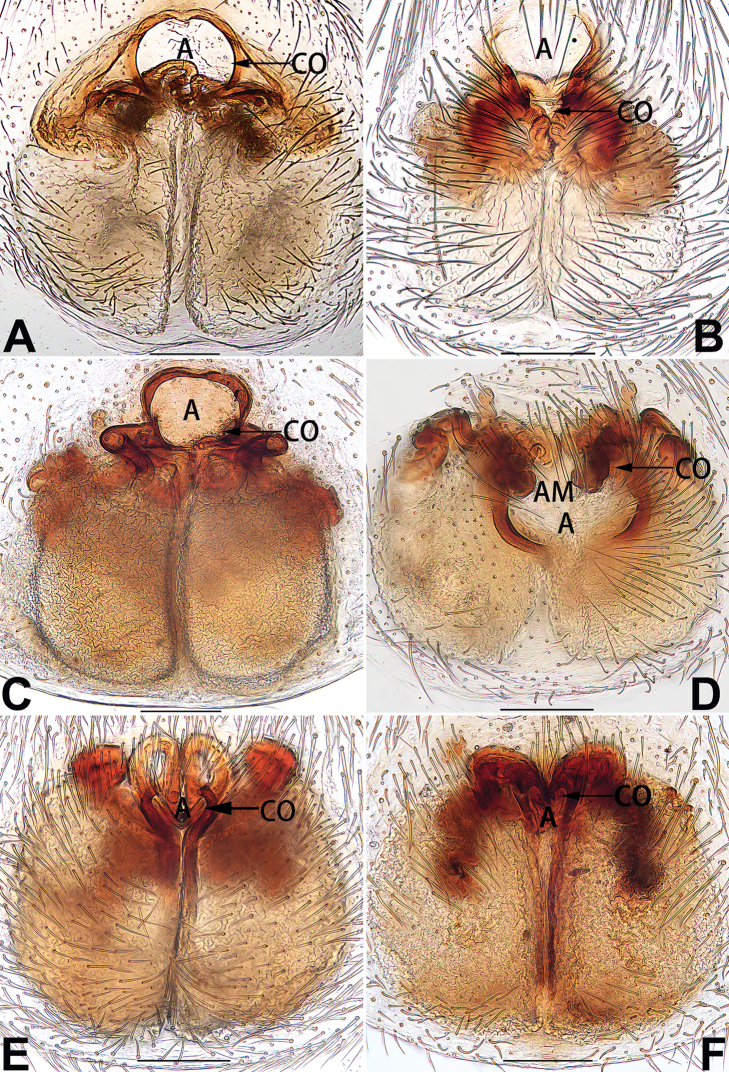
*Clubiona* spp. of the *C. corticalis* group, epigyne, cleared, ventral view **A***C. cochlearis***B***C. dengpao* sp. nov., holotype **C***C. yejiei* sp. nov., holotype **D***C. tixing* sp. nov., holotype **E***C. subrama***F***C. zhigangi* sp. nov., paratype. Abbreviations: A = atrium; AM = atrial membrane; CO = copulatory opening. Scale bars: 0.2 mm.

**Figure 82.**
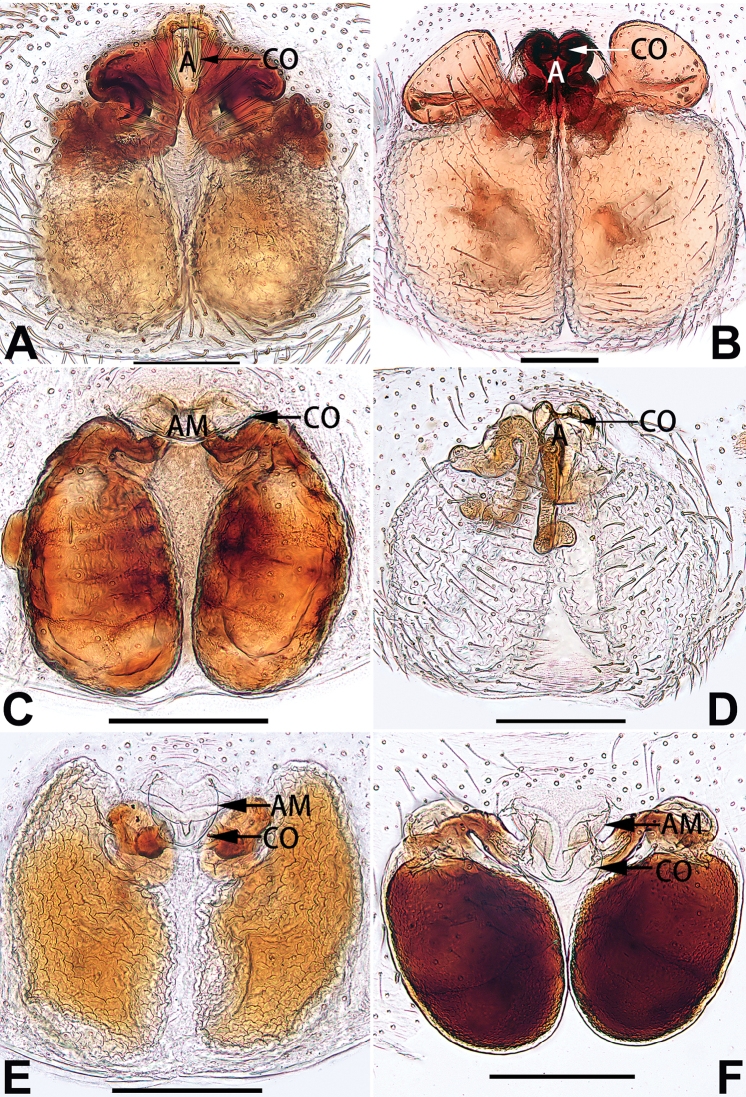
*Clubiona* spp. of the *C. corticalis* group, epigyne, cleared, ventral view **A***C. xiaokong* sp. nov., holotype **B***C. zhaoi* sp. nov., holotype **C***C. kai***D***C. tiane***E***C. didentata***F***C. subdidentata* sp. nov., holotype. Abbreviations: A = atrium; AM = atrial membrane; CO = copulatory opening. Scale bars: 0.2 mm.

**Figure 83.**
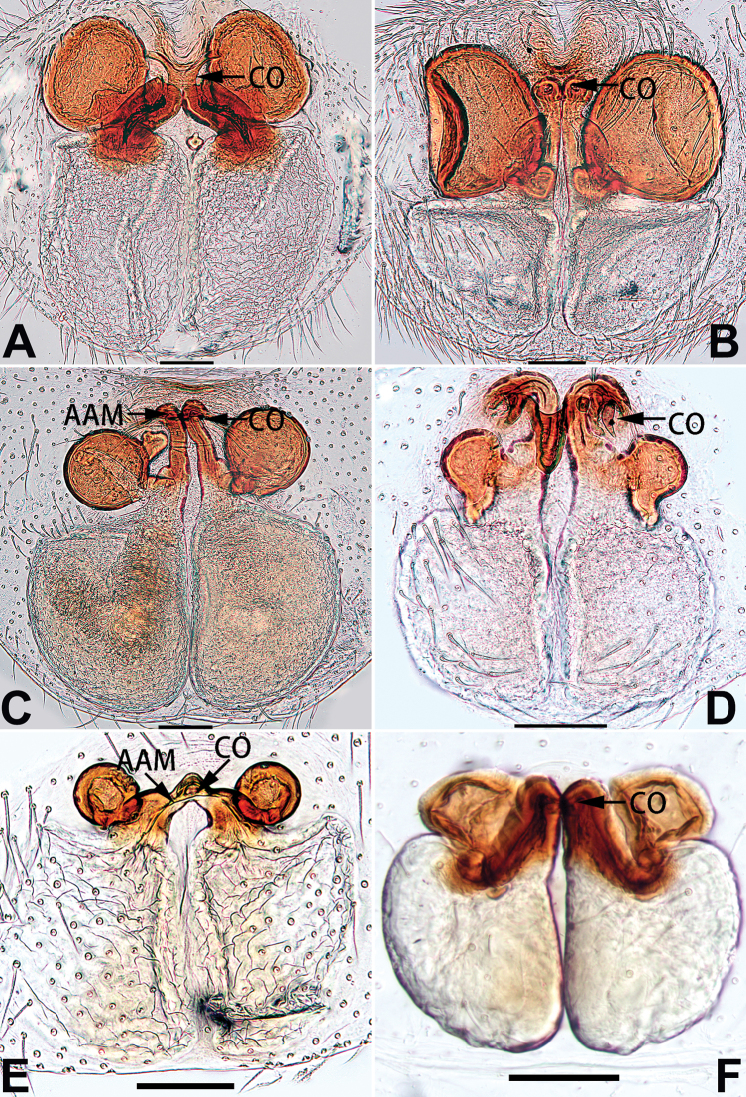
*Clubiona* spp. of the *C. corticalis* group, epigyne, cleared, ventral view **A***C. moralis***B***C. submoralis***C***C. parconcinna***D***C. multidentata***E***C. xiaoci* sp. nov., paratype **F***C. subyaginumai*. Abbreviations: AAM = atrial anterior margin; CO = copulatory opening. Scale bars: 0.1 mm.

**Figure 84.**
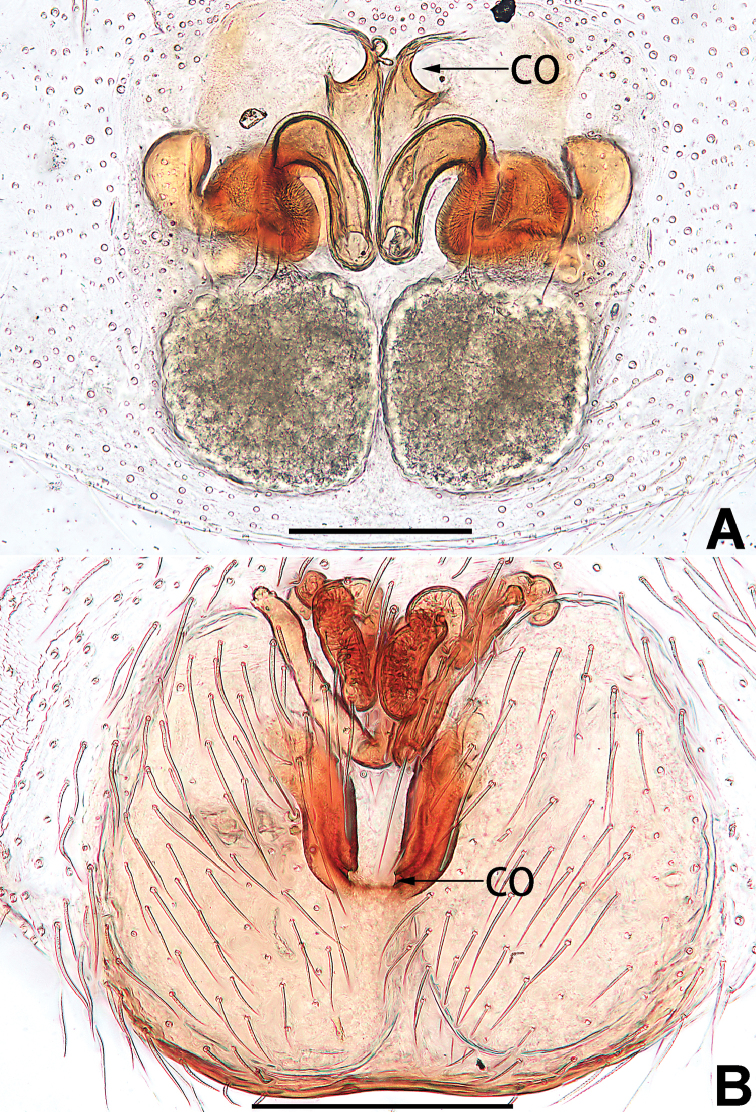
*Clubiona* spp. of the *C. corticalis* group, epigyne, cleared, ventral view **A***C. kurosawai***B***C. pollicaris*. Abbreviations: CO = copulatory opening. Scale bars: 0.2 mm.

**Figure 85.**
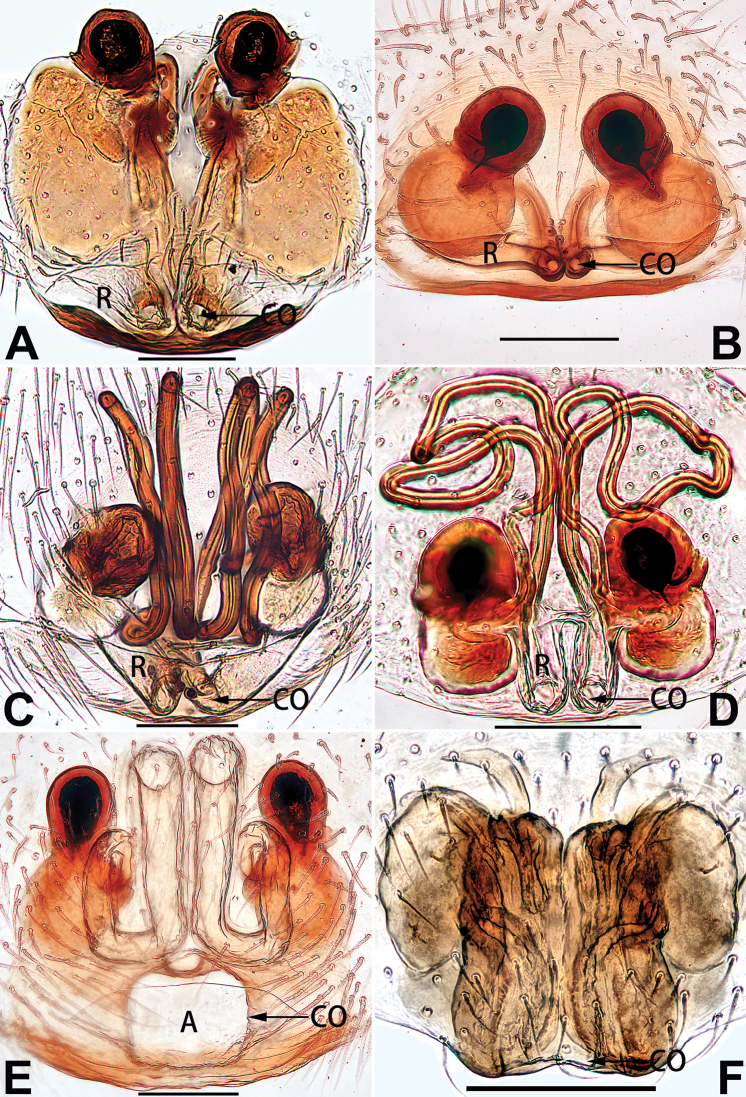
*Clubiona* spp. of the *C. ternatensis* group (**A–E**) and the *C. trivialis* group (**F**), epigyne, cleared, ventral view **A***C. mii* sp. nov., holotype **B***C. subkuu***C***C. theoblicki***D***C. tongi***E***C. zhengi***F***C. bicornis*. Abbreviations: A = atrium; CO = copulatory opening; R = epigynal ridge. Scale bars: 0.1 mm.

**Figure 86.**
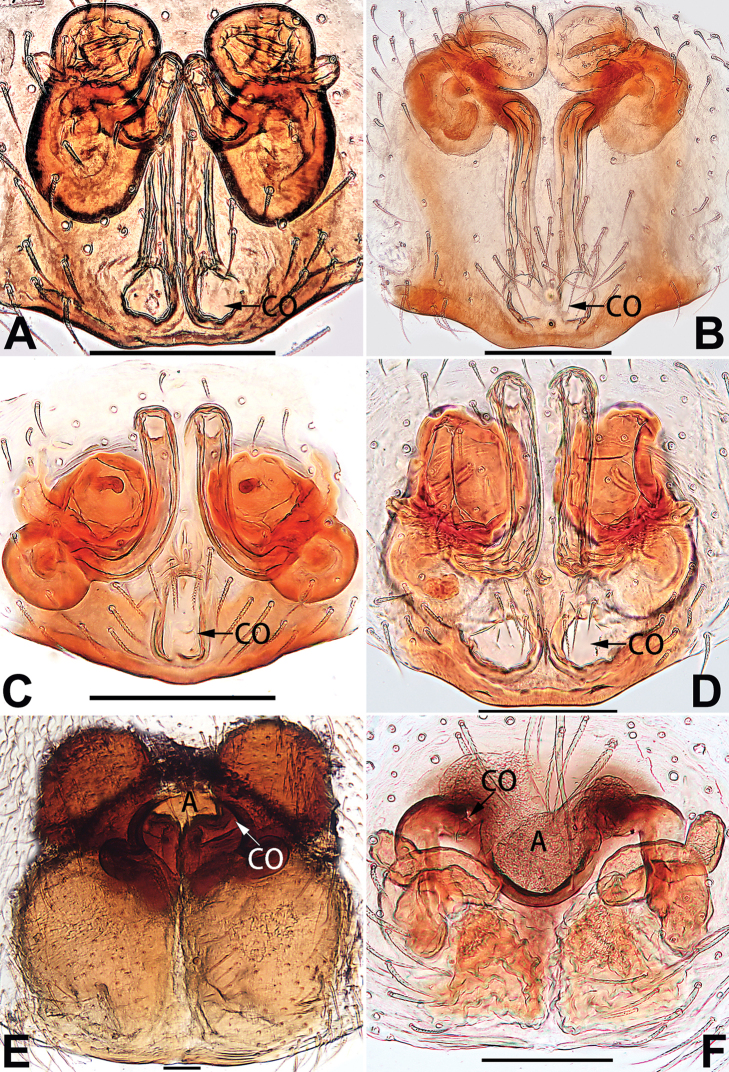
*Clubiona* spp. of the *C. trivialis* group (**A–D**) and the *C. filicata* group (**E, F**), epigyne, cleared, ventral view **A***C. cheni***B***C. menglun* sp. nov., holotype **C***C. subasrevida***D***C. subquebecana***E***C. banna* sp. nov., paratype **F***C. melanosticta*. Abbreviations: A = atrium; CO = copulatory opening. Scale bars: 0.1 mm.

**Figure 87.**
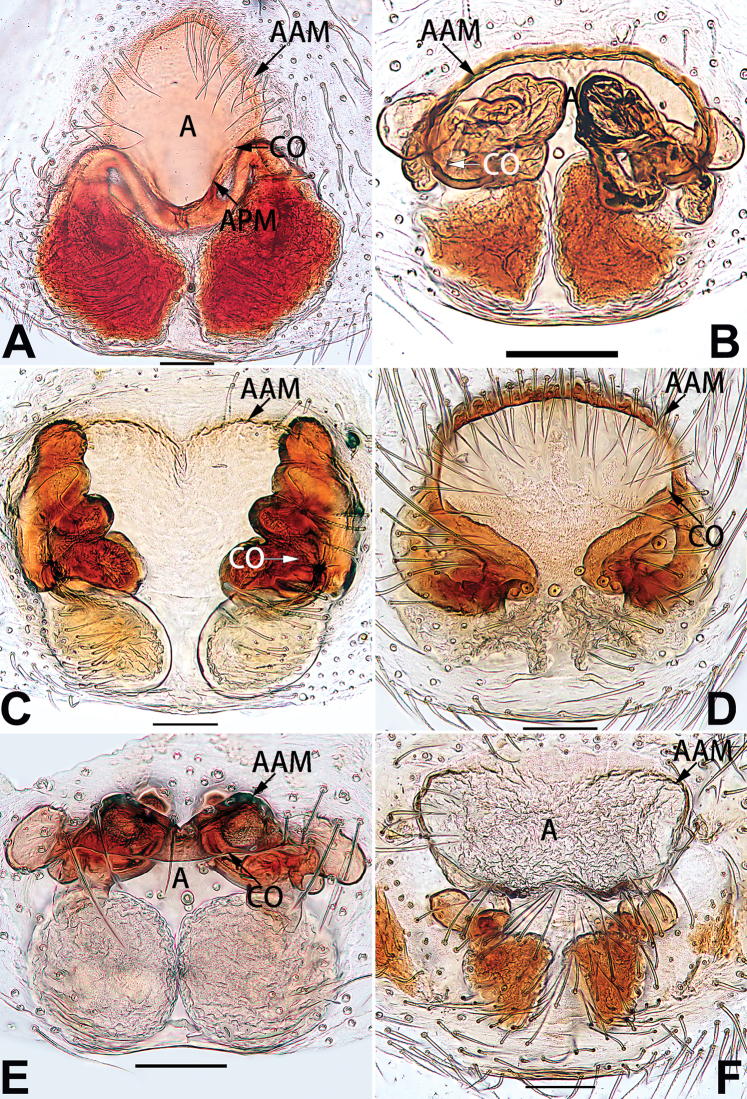
*Clubiona* spp. of the *C. filicata* group, epigyne, cleared, ventral view **A***C. circulata***B***C. reichlini***C***C. grucollaris***D***C. lala***E***C. suthepica***F***C. yueya*. Abbreviations: A = atrium; AAM = atrial anterior margin; APM = atrial posterior margin; CO = copulatory opening. Scale bars: 0.1 mm.

**Figure 88.**
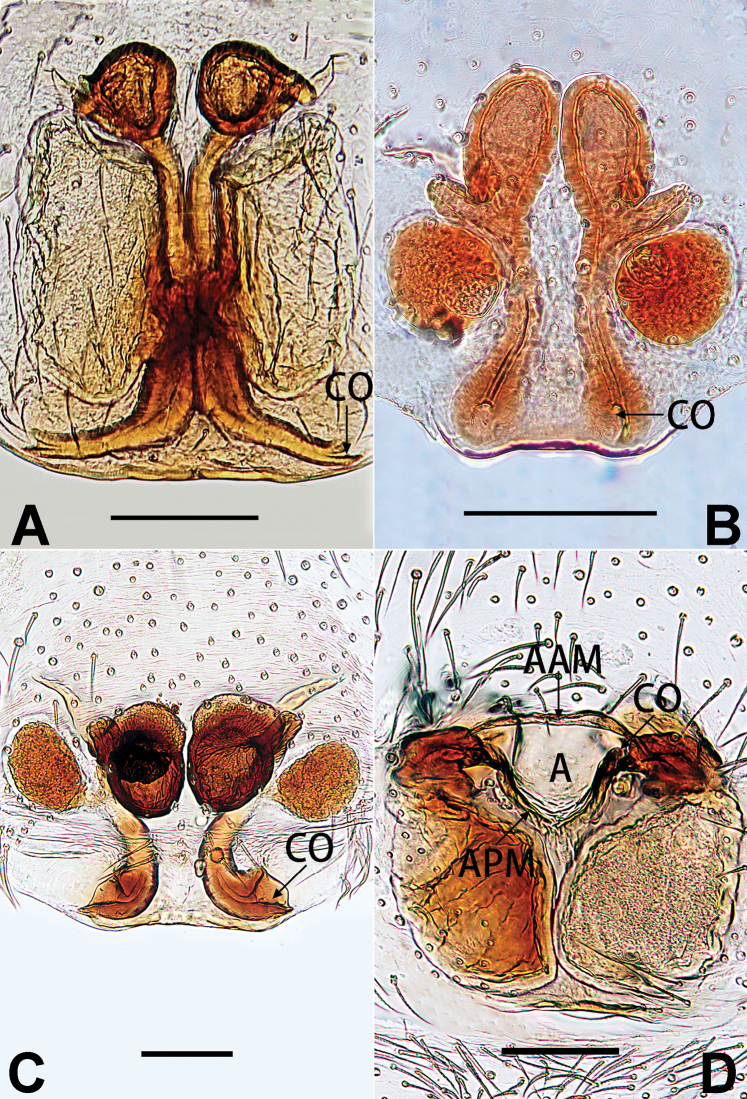
*Clubiona* spp., epigyne, cleared, ventral view **A***C. yaoi***B***C. jiandan***C***C. wangchengi* sp. nov., holotype **D***C. shuangsi* sp. nov., paratype. Abbreviations: A = atrium; AAM = atrial anterior margin; APM = atrial posterior margin; CO = copulatory opening. Scale bars: 0.1 mm.

**Figure 89.**
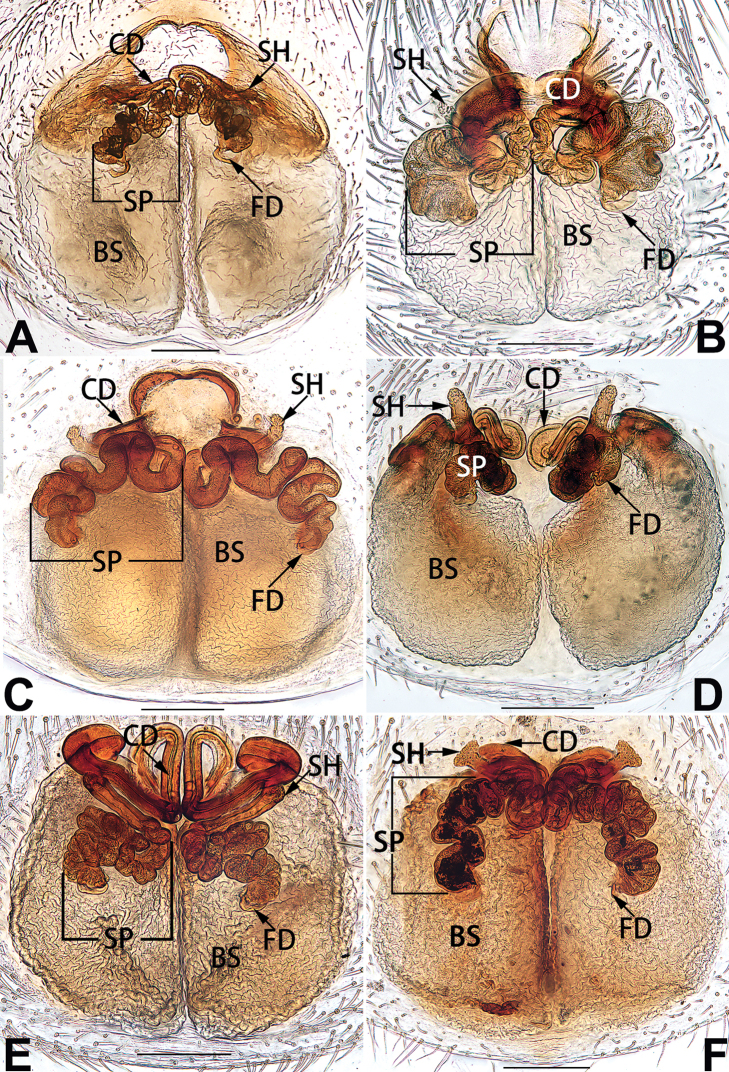
*Clubiona* spp. of the *C. corticalis* group, vulva, cleared dorsal view **A***C. cochlearis***B***C. dengpao* sp. nov., holotype **C***C. yejiei* sp. nov., holotype **D***C. tixing* sp. nov., holotype **E***C. subrama***F***C. zhigangi* sp. nov., paratype. Abbreviations: BS = bursa; CD = copulatory duct; FD = fertilisation duct; SH = spermathecal head; SP = spermatheca. Scale bars: 0.2 mm.

**Figure 90.**
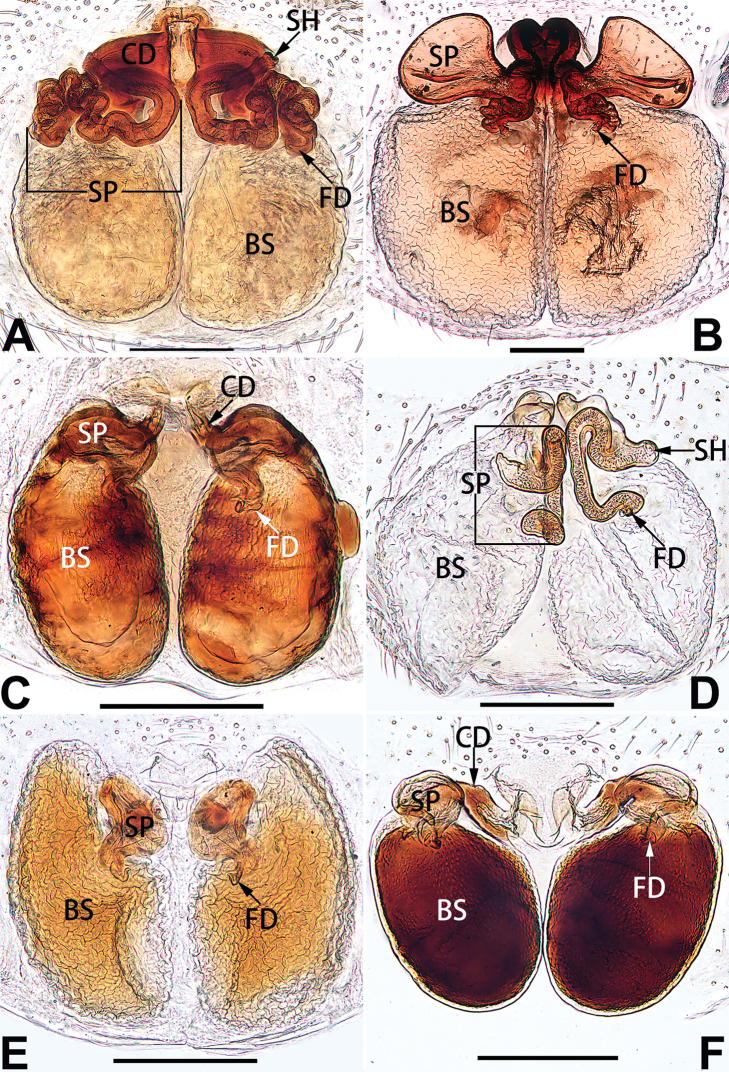
*Clubiona* spp. of the *C. corticalis* group, vulva, cleared, dorsal view **A***C. xiaokong* sp. nov., holotype **B***C. zhaoi* sp. nov., holotype **C***C. kai***D***C. tiane***E***C. didentata***F***C. subdidentata* sp. nov., holotype. Abbreviations: BS = bursa; CD = copulatory duct; FD = fertilisation duct; SH = spermathecal head; SP = spermatheca. Scale bars: 0.2 mm.

**Figure 91.**
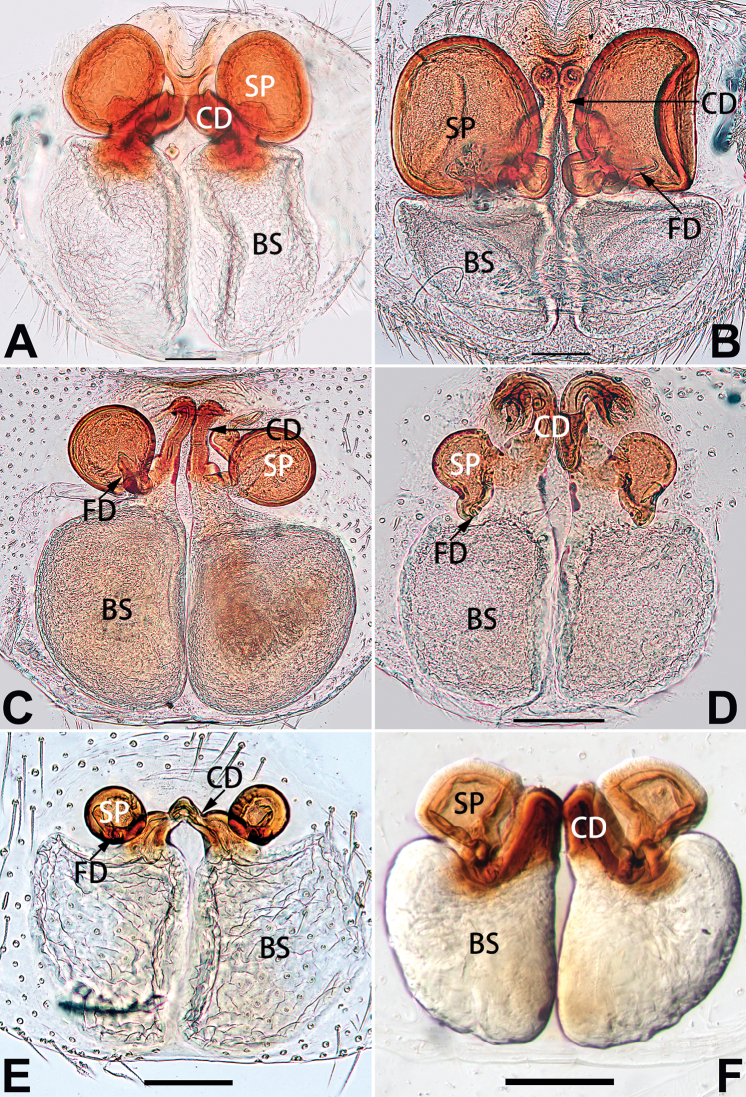
*Clubiona* spp. of the *C. corticalis* group, vulva, cleared, dorsal view **A***C. moralis***B***C. submoralis***C***C. parconcinna***D***C. multidentata***E***C. xiaoci* sp. nov., paratype **F***C. subyaginumai*. Abbreviations: BS = bursa; CD = copulatory duct; FD = fertilisation duct; SP = spermatheca. Scale bars: 0.1 mm.

**Figure 92.**
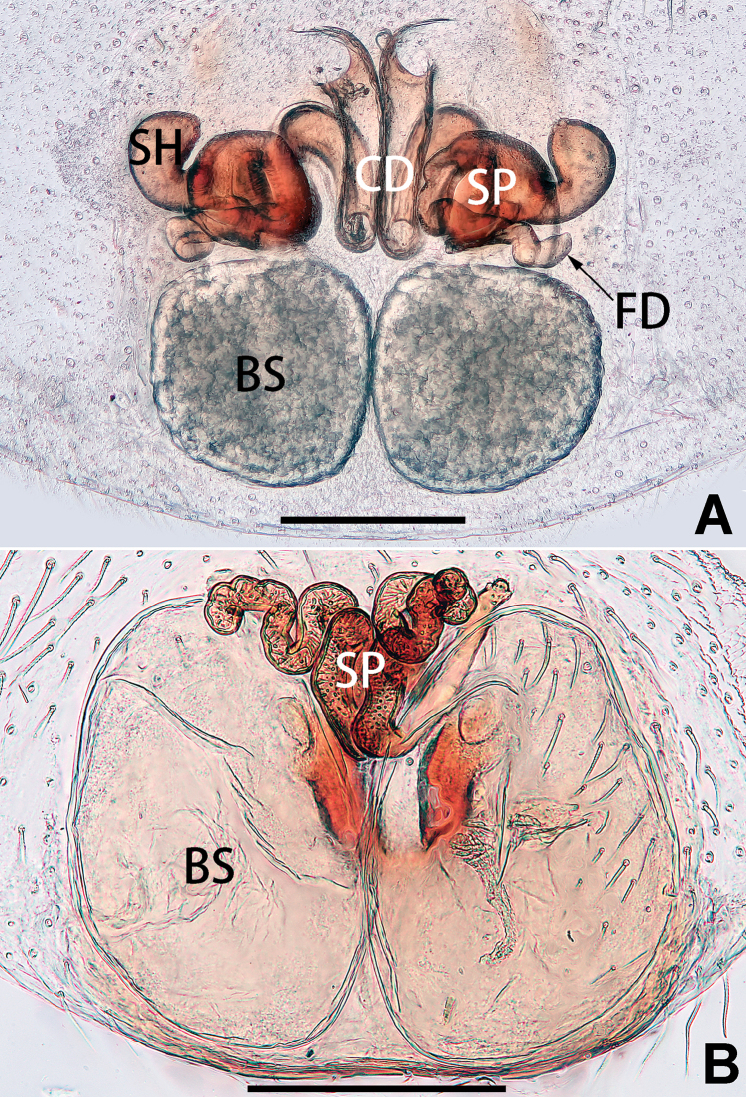
*Clubiona* spp. of the *C. corticalis* group, vulva, cleared, dorsal view **A***C. kurosawai***B***C. pollicaris*. Abbreviations: BS = bursa; CD = copulatory duct; FD = fertilisation duct; SH = spermathecal head; SP = spermatheca. Scale bars: 0.2 mm.

**Figure 93.**
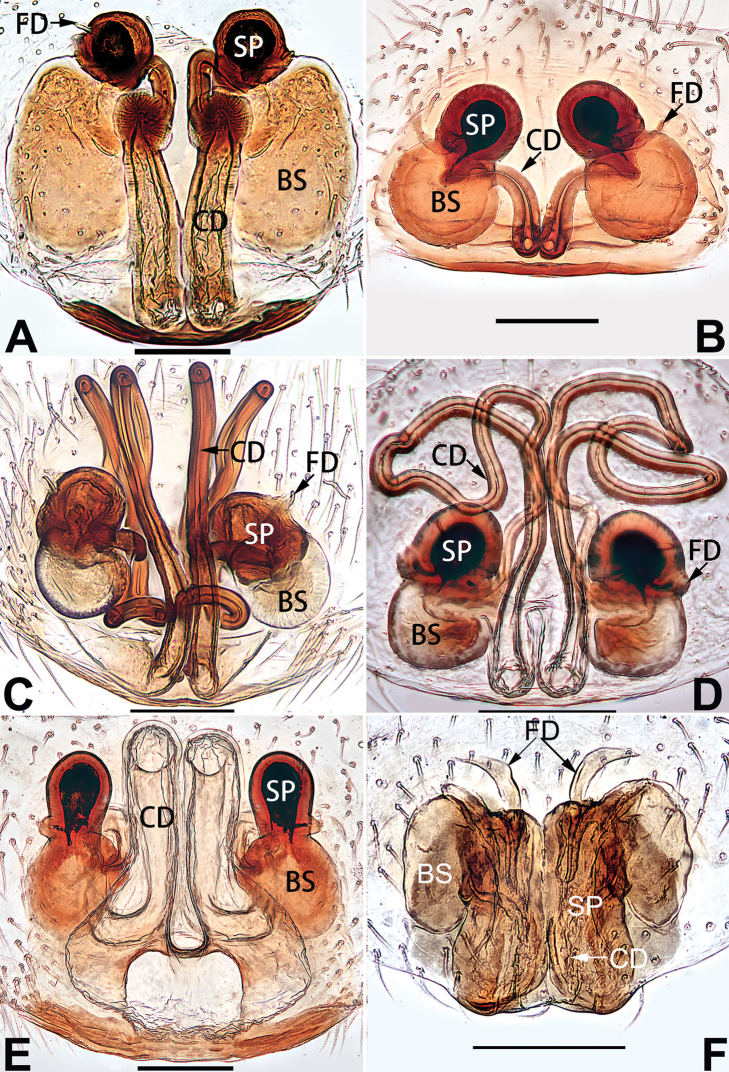
*Clubiona* spp. of the *C. ternatensis* group (**A–E**) and the *C. trivialis* group (**F**), vulva, cleared, dorsal view **A***C. mii* sp. nov., holotype **B***C. subkuu***C***C. theoblicki***D***C. tongi***E***C. zhengi***F***C. bicornis*. Abbreviations: BS = bursa; CD = copulatory duct; FD = fertilisation duct; SP = spermatheca. Scale bars: 0.1 mm.

**Figure 94.**
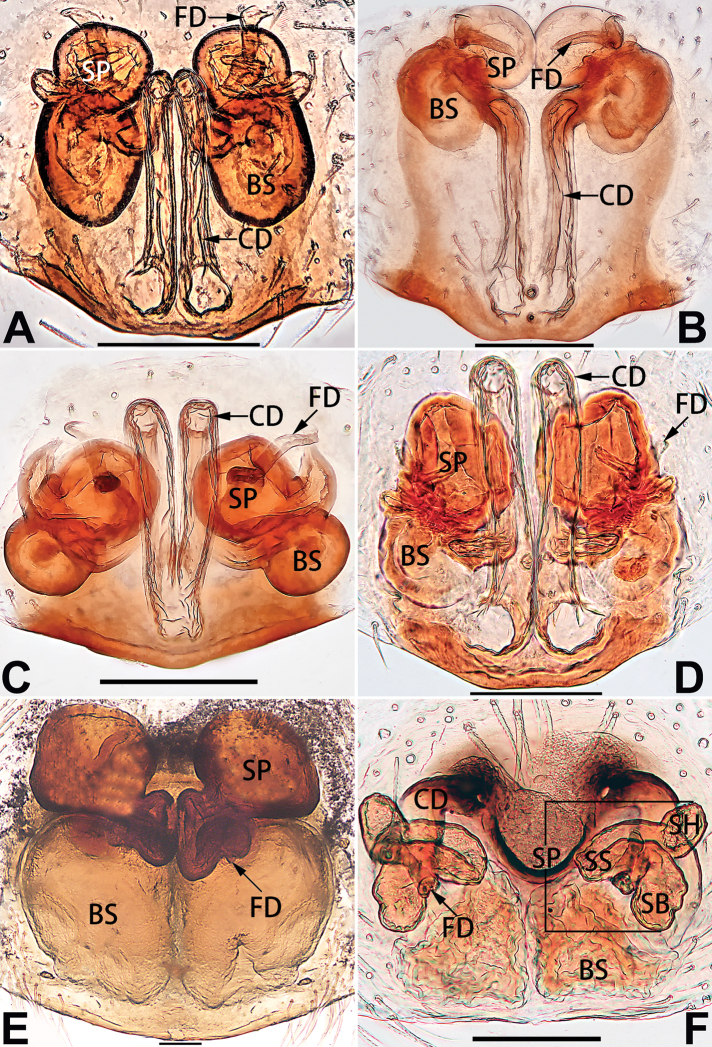
*Clubiona* spp. of the *C. trivialis* group (**A–D**) and the *C. filicata* group (**E, F**), vulva, cleared, dorsal view **A***C. cheni***B***C. menglun* sp. nov., holotype **C***C. subasrevida***D***C. subquebecana***E***C. banna* sp. nov., paratype **F***C. melanosticta*. Abbreviations: BS = bursa; CD = copulatory duct; FD = fertilisation duct; SB = spermathecal base; SH = spermathecal head; SP = spermatheca; SS = spermathecal stalk. Scale bars: 0.1 mm.

**Figure 95.**
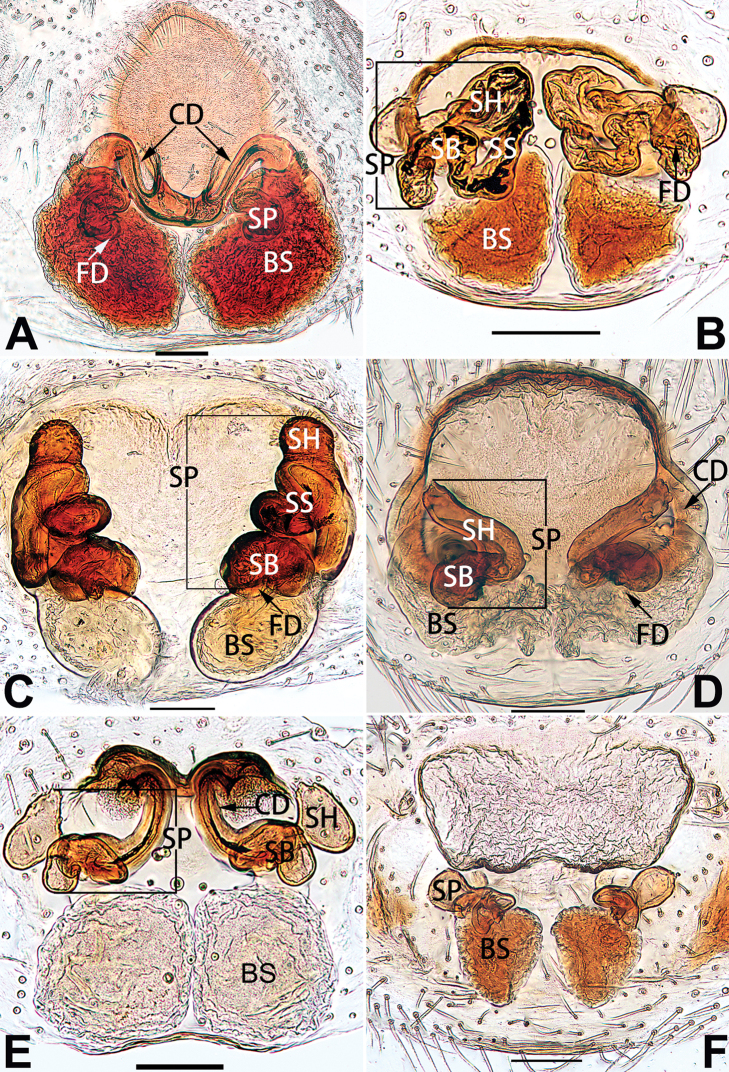
*Clubiona* spp. of the *C. filicata* group, vulva, cleared, dorsal view **A***C. circulata***B***C. reichlini***C***C. grucollaris***D***C. lala***E***C. suthepica***F***C. yueya*. Abbreviations: BS = bursa; CD = copulatory duct; FD = fertilisation duct; SB = spermathecal base; SH = spermathecal head; SP = spermatheca; SS = spermathecal stalk. Scale bars: 0.1 mm.

**Figure 96.**
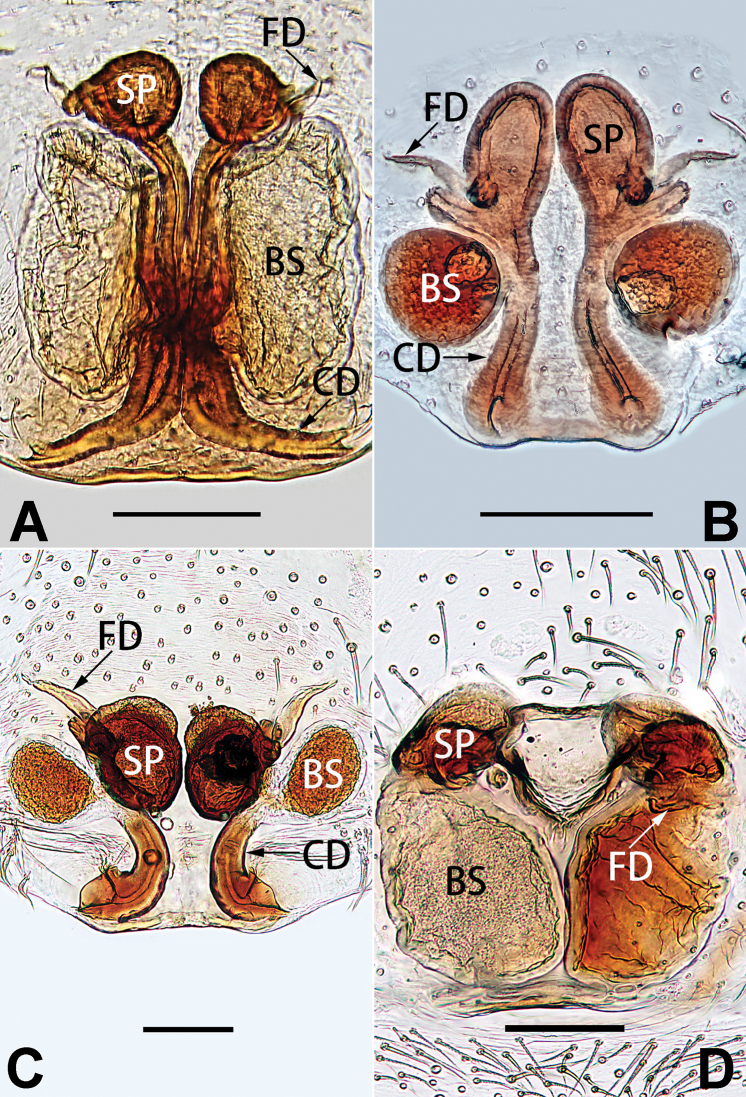
*Clubiona* spp., vulva, cleared, dorsal view **A***C. yaoi***B***C. jiandan***C***C. wangchengi* sp. nov., holotype **D***C. shuangsi* sp. nov., female paratype. Abbreviations: BS = bursa; CD = copulatory duct; FD = fertilisation duct; SP = spermatheca. Scale bars: 0.1 mm.

*Clubiona wangchengi* sp. nov. resembles *C. subkuu* by the similar habitus: wide head, not much narrower than the carapace, and yellowish body (Fig. 52F, G; Yu and Li 2019a: 164, fig. 10G, H) but is consistently separable by the epigyne. The epigynes of both species share the similarly globular spermathecae and bursae and short and curved copulatory ducts which ascend obliquely but differ in the following: (1) the epigynal plate without ridges in *C. wangchengi* sp. nov. (Figs 52A–C; 80C, 88C) (vs. with blade-shaped ridges in *C. subkuu*; Figs 77B, 85B); (2) bursae located on the lateral sides of the spermathecae in *C. wangchengi* sp. nov. (Figs 52D, E; 96C) (vs. bursae located posterior to the spermathecae in *C. subkuu*; Fig. 93B).

##### Remarks.

*Clubiona wangchengi* sp. nov. resembles some members of the *C. ternatensis* group by the wide head and the general shape of the vulva but can be distinguished from these species by the absence of epigynal ridges. Because all *C. ternatensis* group species have epigynal ridges (or hoods, or folds), there remains considerable uncertainty about placing this new species in the *ternatensis group*. In addition, this new species resembles some species of *Pteroneta* Deeleman-Reinhold, 2001, which is most similar to the *C. ternatensis* group in genital morphology. This new species can be separated from all known members of the genus *Pteroneta* by its unpatterned yellow body (*Pteroneta* has a pale green body, ventrally with lazulite blue spots). Despite the similarity of the general shape of the vulva in *C. wangchengi* sp. nov. and species of the *ternatensis group* and the *Pteroneta*, it is currently impossible to discern any obvious derived features (i.e., epigynal ridges and pale green body) that could indicate placement in the *Clubiona ternatensis* group or the genus *Pteroneta*.

##### Description.

**Female.** Holotype (Fig. 52F, G): Total length 8.25; carapace 3.41 long, 2.24 wide; opisthosoma 4.84 long, 2.89 wide. Carapace orange, slightly darker in front, without distinct pattern, cephalic region slightly narrowed, cervical groove and radial grooves indistinct; tegument smooth, anteriorly clothed with sparse setae. Eyes: AER slightly recurved, PER slightly wider than AER, almost straight in dorsal view. Eye sizes and interdistances: AME 0.17, ALE 0.15, PME 0.15, PLE 0.13, AME–AME 0.08, AME–ALE 0.11, PME–PME 0.39, PME–PLE 0.21, MOQL 0.43, MOQA 0.42, MOQP 0.69. Chelicerae robust and brownish red, with three teeth on promargin and two on retromargin. Sternum yellowish white, 1.72 long, 1.03 wide. Labium and endites coloured as carapace. Legs light yellow, without distinct markings. Leg measurements: I 5.63 (1.64, 2.45, 0.97, 0.56), II 6.00 (1.76, 2.60, 1.06, 0.58), III – (–, 2.83, 2.36, –), IV – (1.73, 1.36, 0.46, –). Abdomen elongate, oval, uniformly cream coloured, dorsum with a narrow, heart-shaped mark and two pairs of conspicuous muscle depressions; venter medially with two longitudinal dotted lines.

Epigyne (Figs 52A–E, 80C, 88C, 96C). Epigynal plate ca. 1.5 × wider than long, with spermathecae, bursae and ducts prominent through tegument. Copulatory openings large, located on the window (or chitinous structure) which is at the postero-lateral portion of the epigynal plate. Copulatory ducts short and curved, connected to bursae midway between epigastric fold and anterior surface of the spermathecae. Both spermathecae and bursae are globular, the former larger than the latter. Spermathecae sclerotised, close together. Fertilisation ducts acicular, relatively long, nearly equal to spermathecae diameter, located on anterolateral surface of spermathecae. Bursae located on the lateral sides of the spermathecae, separated by ca. two diameters. Bursal surface membranous and wrinkled, inside pigmented and sclerotised.

**Male.** Unknown.

##### Distribution.

Known only from the type locality, Xishuangbanna, Yunnan, China.

## Supplementary Material

XML Treatment for Clubiona

XML Treatment for Clubiona milingae

XML Treatment for Clubiona yaoi

XML Treatment for Clubiona corticalis

XML Treatment for Clubiona cochlearis

XML Treatment for Clubiona dengpao

XML Treatment for Clubiona didentata

XML Treatment for Clubiona kai

XML Treatment for Clubiona kurosawai

XML Treatment for Clubiona moralis

XML Treatment for Clubiona multidentata

XML Treatment for Clubiona parconcinna

XML Treatment for Clubiona pollicaris

XML Treatment for Clubiona rama

XML Treatment for Clubiona subdidentata

XML Treatment for Clubiona submoralis

XML Treatment for Clubiona subrama

XML Treatment for Clubiona subyaginumai

XML Treatment for Clubiona tixing

XML Treatment for Clubiona tiane

XML Treatment for Clubiona xiaoci

XML Treatment for Clubiona xiaokong

XML Treatment for Clubiona yejiei

XML Treatment for Clubiona zhaoi

XML Treatment for Clubiona zhigangi

XML Treatment for Clubiona ternatensis

XML Treatment for Clubiona heteroducta

XML Treatment for Clubiona mii

XML Treatment for Clubiona subkuu

XML Treatment for Clubiona subtongi

XML Treatment for Clubiona theoblicki

XML Treatment for Clubiona tongi

XML Treatment for Clubiona zhengi

XML Treatment for Clubiona japonicola

XML Treatment for Clubiona japonicola

XML Treatment for Clubiona filicata

XML Treatment for Clubiona abnormis

XML Treatment for Clubiona banna

XML Treatment for Clubiona circulata

XML Treatment for Clubiona reichlini

XML Treatment for Clubiona filicata

XML Treatment for Clubiona filoramula

XML Treatment for Clubiona grucollaris

XML Treatment for Clubiona lala

XML Treatment for Clubiona melanosticta

XML Treatment for Clubiona suthepica

XML Treatment for Clubiona yueya

XML Treatment for Clubiona zhanggureni

XML Treatment for Clubiona zhangyongjingi

XML Treatment for Clubiona trivialis

XML Treatment for Clubiona bicornis

XML Treatment for Clubiona cheni

XML Treatment for Clubiona menglun

XML Treatment for Clubiona subasrevida

XML Treatment for Clubiona subquebecana

XML Treatment for Clubiona jiandan

XML Treatment for Clubiona shuangsi

XML Treatment for Clubiona wangchengi
